# Engineering Bodipy‐Based Metal–Organic Frameworks for Efficient Full‐Spectrum Photocatalysis in Amide Synthesis

**DOI:** 10.1002/anie.202505405

**Published:** 2025-04-14

**Authors:** Binhui Liu, Xu Chen, Yuhao Yang, Mohammad Reza Alizadeh Kiapi, Dhruv Menon, Qianyi Zhao, Guozan Yuan, Luke L. Keenan, David Fairen‐Jimenez, Qingchun Xia

**Affiliations:** ^1^ Henan Key Laboratory of Boron Chemistry and Advanced Energy Materials School of Chemistry and Chemical Engineering Henan Normal University Xinxiang Henan 453007 China; ^2^ Department of Chemical Engineering & Biotechnology University of Cambridge Philippa Fawcett Drive Cambridge CB3 0AS UK; ^3^ School of Chemistry and Chemical Engineering Anhui University of Technology Ma'anshan Anhui 243032 China; ^4^ Diamond Light Source Ltd. Harwell Science and Innovation Campus Chilton Didcot OX11 0DE UK

**Keywords:** Bodipy, Heterogenous photocatalysis, Metal–organic framework, Post‐synthesis modification, Visible light

## Abstract

Developing photocatalysts that can efficiently utilize the full solar spectrum is a crucial step toward transforming sustainable energy solutions. Due to their light absorption limitations, most photo‐responsive metal–organic frameworks (MOFs) are constrained to the ultraviolet (UV) and blue light regions. Expanding their absorption to encompass the entire solar spectrum would unlock their full potential, greatly enhancing efficiency and applicability. Here, we report the design and synthesis of a series of highly stable boron‐dipyrromethene (bodipy)‐based MOFs (BMOFs) by reacting dicarboxyl‐functionalized bodipy ligands with Zr‐oxo clusters. Leveraging the acidity of the methyl groups on the bodipy backbone, we expanded the conjugation system through a solid‐state condensation reaction with various aldehydes, achieving full‐color absorption, thereby extending the band edge into the near‐infrared (NIR) and infrared (IR) regions. These BMOFs demonstrated exceptional reactivity and recyclability in heterogeneous photocatalytic activities, including C─H bond activation of saturated aza‐heterocycles and C─N bond cleavage of *N*,*N*‐dimethylanilines to produce amides under visible light. Our findings highlight the transformative potential of BMOFs in photocatalysis, marking a significant leap forward in the design of advanced photocatalytic materials with tunable properties.

## Graphical abstract

Three highly stable bodipy‐based metal–organic frameworks (BMOFs) were synthesized by treating dicarboxyl‐functionalized bodipy ligands with Zr‐oxo clusters. Featuring the distinct bodipy scaffold in the framework, these BMOFs displayed high reactivity in heterogenous photocatalytic C─H bond activation of *N*,*N*‐dimethylanilines to access amides under visible light.

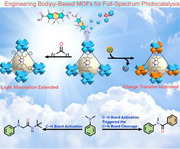

## Introduction

Solar energy is a near‐limitless source of energy. Amidst the rapidly growing demand for clean energy, there is a significant interest in designing highly efficient photocatalysts, which can be tailored to maximize light absorption and energy transfer, meet various requirements, and maintain structural integrity under various harsh conditions.^[^ ^1^, ^2^ ^]^ Meta–organic frameworks (MOFs) are promising materials with extensive applications, which have captured growing interest in visible‐light‐driven photocatalysis, distinguishing themselves from conventional photocatalysts.^[^ ^3^, ^4^, ^5^ ^]^ In MOF photocatalysts, the ligands absorb light, and the photogenerated charges are subsequently transferred to the metal‐oxo clusters to facilitate redox reactions. This process leads to the generation of a charge separation state, endowing MOF photocatalysts with semiconductor behavior.^[^ ^6^, ^7^ ^]^

Benefiting from the modular nature of MOFs, the optical response, electronic valence, and energy level alignment of MOF photocatalysts can be precisely regulated by the selection of photoactive ligands and metal‐oxo clusters.^[^ ^8^, ^9^, ^10^ ^]^ In terms of the ligand, π‐conjugation systems can be extended via post‐synthetic modifications (PSM).^[^ ^11^ ^]^ Such an extension can subsequently tune the optical responsiveness of MOF photocatalysts to cover the entire visible region and further stretch into the near‐infrared (NIR) and infrared regions (IR). This massively improves the solar energy capture capabilities and photocatalytic activity.^[^ ^12^, ^13^, ^14^, ^15^ ^]^ For the metal‐oxo cluster, introducing alternate exogenous metal ions to decorate the intrinsic cluster has been recognized as another effective strategy to improve photocatalytic performance.^[^ ^16^, ^17^, ^18^ ^]^ These exogenous metal ions can act as a charge sink or pump to trap the photogenerated charge across a junction to steer the unidirectional electron flow, thereby inhibiting electron–hole recombination and improving photocatalytic activities.^[^ ^19^, ^20^, ^21^ ^]^ Consequently, engineering either the ligand or the metal‐oxo cluster, which leads to a predictable change in the semiconductor behaviour of MOF photocatalysts, is considered an effective methodology for enhancing photocatalytic activity.^[^ ^22^, ^23^ ^]^

Boron‐dipyrromethenes (bodipy) are a class of versatile and robust photoactive monomers that have received great attention in the context of photocatalysis.^[^ ^24^, ^25^ ^]^ Typically, unsubstituted bodipy monomers absorb around 500 nm, and there are several strategies for tailoring absorption toward the NIR and IR regions to meet specific needs. One particularly effective method involves the condensation reaction of acidic methyl groups found in methyl‐substituted bodipy with selected aromatic aldehydes.^[^ ^26^, ^27^ ^]^ Such condensation reactions are synthetically straightforward and lead to the extension of π‐conjugation in the bodipy backbone, enabling easy tuning of the absorption band of the bodipy backbone within the 500–900 nm range. To date, bodipy‐based MOFs (BMOFs) with fixed absorption behavior have been successfully fabricated for photocatalysis.^[^ ^28^, ^29^, ^30^, ^31^, ^32^, ^33^, ^34^ ^]^ Despite these achievements, it is necessary to rationally design more versatile systems with tunable absorbance rather than creating specific BMOFs from scratch for each desired application.

**Scheme 1:**
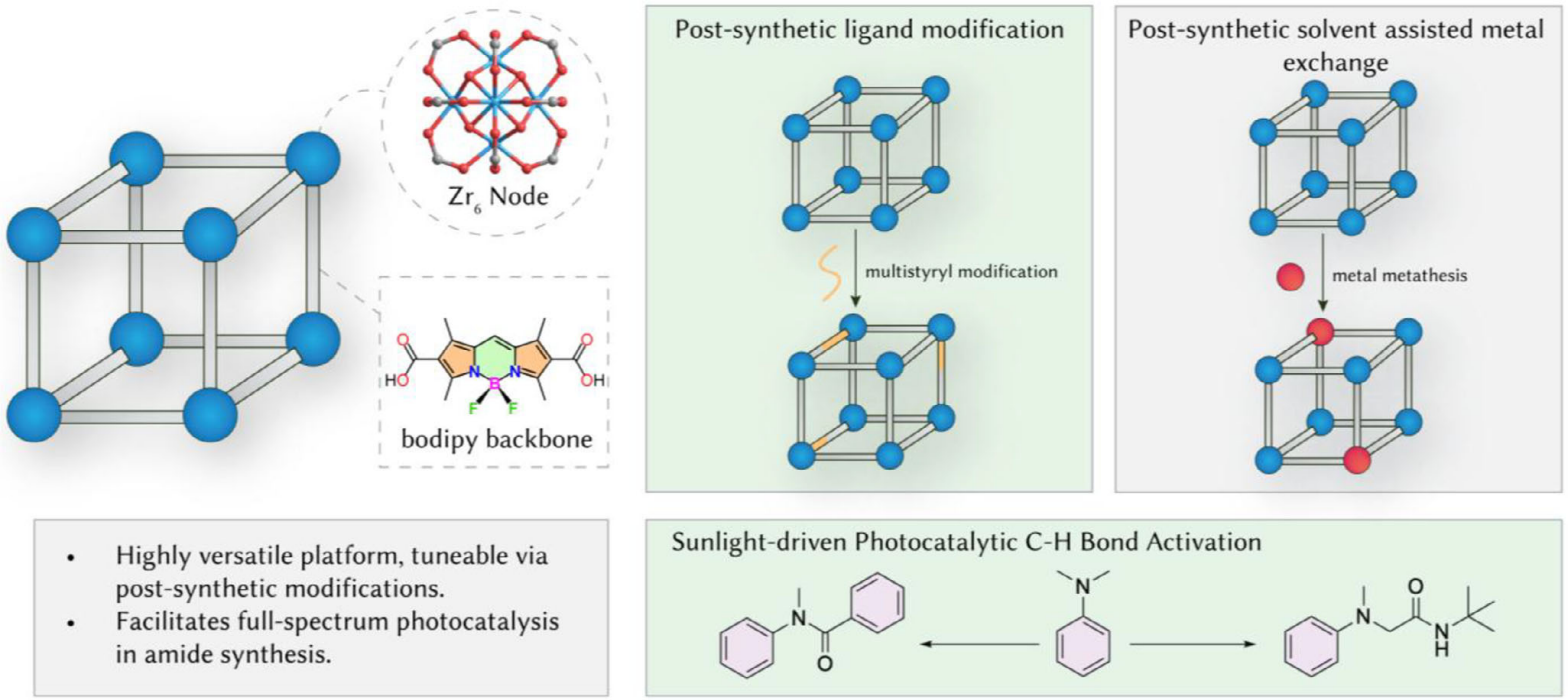
Zr‐BMOFs as dual‐modifiable platforms for efficient full‐spectrum photocatalysis in C─H bond activation for amide synthesis.

Here, we report the synthesis of three highly stable and tunable Zr‐based BMOFs (**1^Zr^**, **2^Zr^**, and **3^Zr^**) by treating dicarboxyl‐functionalized bodipy ligands with Zr‐oxo clusters. These BMOFs provide a dual platform for tailoring their absorption behavior and catalytic activities. By leveraging the acidity of the methyl groups at the 1,7‐ and 3,5‐ positions of the bodipy backbone in **1^Zr^**, the π‐conjugation of the bodipy backbone could be extended via the solid‐state condensation reaction with various aldehydes, resulting in full‐color absorption with the band edge even extending into the NIR region. Moreover, the Zr‐oxo cluster could be decorated by partially replacing Zr ions with exogenous metal ions like Sc^3+^, Ti^4+^, V^4+^, and Sn^4+^ via metal metathesis. Notably, the well‐aligned bodipy scaffold within the channel endows our BMOFs with photocatalytic activities, enabling the direct C(sp^3^)−H carbamoylation of saturated aza‐heterocycles and the dealkylation/acylation of *N*,*N*‐dimethylanilines. This process facilitates the synthesis of α‐amino amides under visible light irradiation. Our strategy demonstrates the potential for the utilization of BMOFs for heterogeneous photocatalysis, and, more importantly, provides a feasible, facile, and straightforward route for designing bodipy‐containing photocatalysts with a desired absorption range (Scheme 1).

## Results and Discussion

### Synthesis and Characterization of 1^Zr^, 2^Zr^, and 3^Zr^

**Figure 1:**
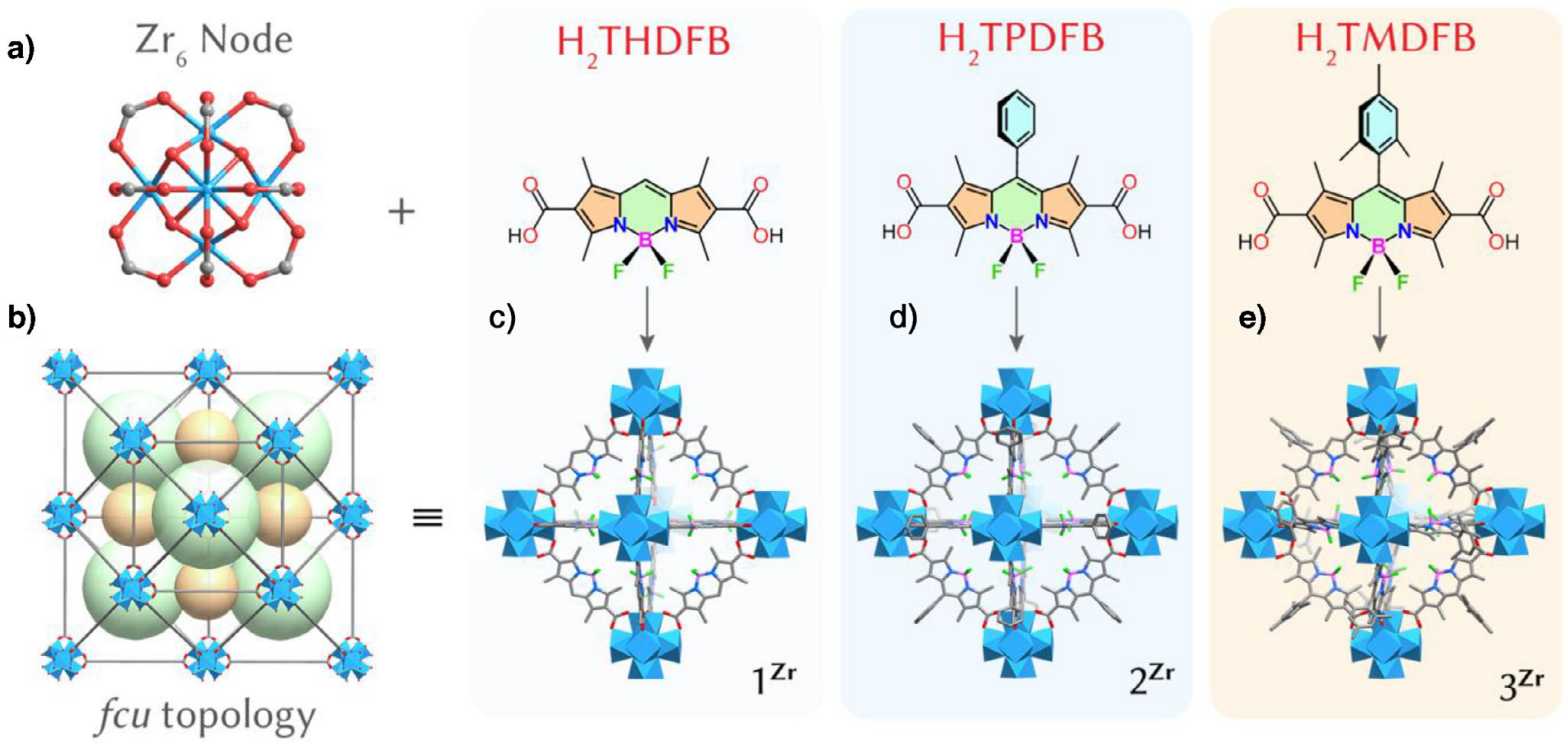
Schematic illustration of the synthesis of **1^Zr^**, **2^Zr^**, and **3^Zr^**. a) The building blocks – Zr_6_ node, H_2_THDFB, H_2_TPDFB, and H_2_TMDFB. b) The *fcu* topology. c–e) Structure of the cages in **1^Zr^**, **2^Zr^**, and **3^Zr^**, respectively. Zr_6_ nodes, blue polyhedra; O, red; B, pink; F, green; C, gray.

The bodipy ligand H_2_TPDFB was synthesized following a previously reported method.^[^ ^34^ ^]^ The new ligands, H_2_THDFB and H_2_TMDFB, were prepared using a similar process, with overall yields of 56% and 17%, respectively (Schemes S1 and S2). Solvothermal reactions between ZrCl_4_ and H_2_THDFB, H_2_TPDFB, or H_2_TMDFB in DMF, with formic acid (FA) or acetic acid (HAc) as modulating agents, yielded orange octahedral crystals of [Zr_6_O_4_(OH)_4_(THDFB)_6_] (**1^Zr^**), [Zr_6_O_4_(OH)_4_(TPDFB)_6_] (**2^Zr^**), and [Zr_6_O_4_(OH)_4_(TMDFB)_6_] (**3^Zr^**) (Figure 1). They were characterized by Fourier transform infrared (FT‐IR) analysis (Figure S38).

Single‐crystal X‐ray diffraction (SC‐XRD) analysis revealed that **1^Zr^**, **2^Zr^**, and **3^Zr^** were isostructural, crystallizing in the cubic space group *Fm‐3 m*.^[^ ^35^ ^]^ Each framework featured a Zr_6_O_4_(OH)_4_ cluster, with triangular faces capped by four *μ*_3_‐O and four *μ*_3_‐OH groups. Each Zr_6_O_4_(OH)_4_ cluster was linked by 12 bidentate carboxylate groups from 12 ligands, forming a three‐dimensional (3D) network with a UiO‐type *fcu* topology (Figure 1b). For the bodipy ligand, the central six‐membered ring aligned in a manner that was nearly coplanar with the adjacent pyrrole rings, indicating strong π‐electron delocalization within the indacene plane. The phenyl and trimethylphenyl groups at the *meso*‐position in **2^Zr^** and **3^Zr^** were almost perpendicular to the indacene plane. The framework structure of **1^Zr^** involved two kinds of cages with ∼12 and 14 Å inner diameters for the small tetrahedral and large octahedral pores, respectively (Figure 1b,c). Both cages were accessible by ∼9.5 Å triangular windows (Figure 1b). PLATON calculations show that **1^Zr^**, **2^Zr^**, and **3^Zr^** had about 47%, 24.5%, and 13.2% of the total volume available for guest inclusion, respectively.^[^ ^36^ ^]^

**Figure 2:**
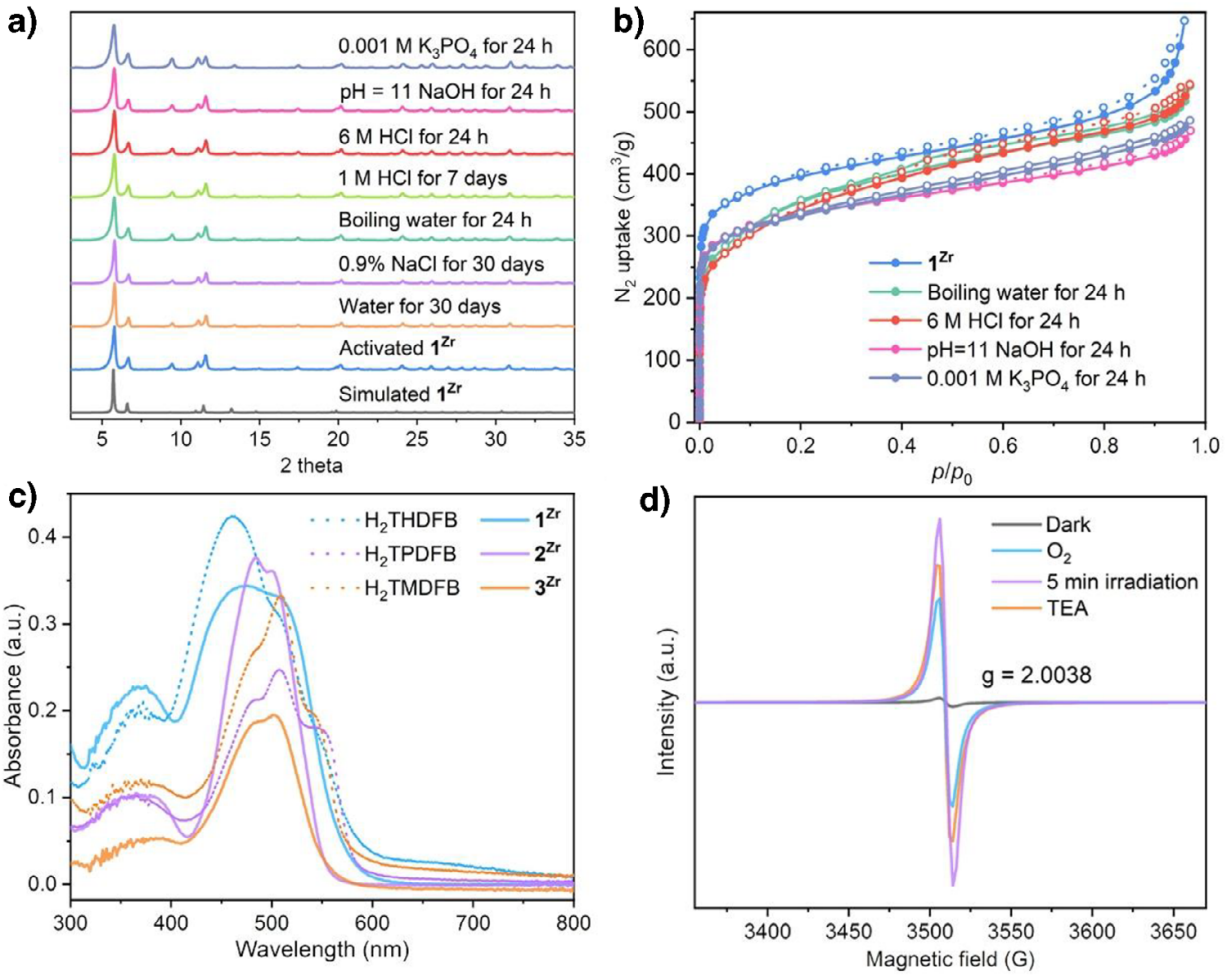
a,b) PXRD patterns and N_2_ adsorption measurements of **1^Zr^** after various treatments. c) UV–vis diffuse reflectance of **1^Zr^**, **2^Zr^**, **3^Zr^**, and their corresponding ligands. d) In situ EPR signal of **1^Zr^** under different conditions.

The phase purity of **1^Zr^**, **2^Zr^**, and **3^Zr^** was confirmed by an excellent match between experimental and simulated PXRD patterns (Figure 2a and Figure S1). N_2_ adsorption isotherms showed Type I adsorption behavior with the Brunauer–Emmett–Teller (BET) areas, calculated using BETSI^[^ ^37^ ^]^ of 1466, 906, and 761 m^2^ g^−1^ (Figure 2b and Figure S2), respectively. Thermogravimetric analysis (TGA) results showed that the included guest molecules within the frameworks could be readily removed from 80 to 250 °C (Figure S3). Variable temperature PXRD (VT‐XRD) experiments confirmed that all three BMOFs maintained their crystallinity up to 300 °C in the open air (Figure S4). PXRD and 77 K N_2_ adsorption analysis further displayed their excellent chemical stability by soaking MOF samples in boiling water, 6 M HCl, NaOH solution (pH = 11), K_3_PO_4_ solution (0.001 M), and PSB (0.1 M, pH = 7.4) for over 24 h. Additionally, the solid‐state UV–vis and PL spectral analyses showed no peak shifts or disappearances after these treatments (Figures S5 and S6). The recovery of the three counterparts displayed little weight loss after these harsh treatments. The resistance of photobleaching was then evaluated by the continuous irradiation of the 300 W Xe lamp for 24 h. As expected, the solid‐state UV–vis absorption and PXRD pattern of the recoveries remained unchanged (Figure S7), suggesting robust photostability for photocatalysis.

Solid‐state UV–vis absorption spectra of **1^Zr^**, **2^Zr^**, and **3^Zr^** showed that all three BMOFs featured strong absorption bands in the region of 400–600 nm, similar to their bodipy ligand counterparts (Figure 2c). The band gaps, estimated from the Tauc plot, were determined to be 2.25, 2.32, and 2.30 eV for **1^Zr^**, **2^Zr^,** and **3^Zr^**, respectively (Figure S8), which almost aligned well with the DFT calculation of 2.34 eV (see Section 7.5 in Supporting Information). Mott–Schottky measurements indicated that they were all n‐type semiconductors, with flat bands of −0.55, −0.59, and −0.55 V versus Ag/AgCl (Figure S9). Considering the bottom of the conduction band (LUMO) in n‐type semiconductors is about 0.2 V above the flat band,^[^ ^34^ ^]^ therefore, the LUMO levels for **1^Zr^**, **2^Zr,^** and **3^Zr^** were located at about −0.35, −0.39, and −0.35 V versus NHE, and their corresponding valence bands (HOMO) were then calculated to be 1.86, 1.93, and 1.95 V versus NHE, respectively.

Steady‐state photoluminescence emission spectra and time‐resolved fluorescence spectra (TRFL) were then employed to further investigate the photogenerated charge separation in these three BMOFs. As shown in Figure S10, with an excitation wavelength of 500 nm, the bodipy ester monomer exhibited a strong orange luminescence with a maximum emission at 607 nm. Significantly, a drastic intensity decrease was observed when the bodipy scaffold was fabricated into the networks. We attributed such a decrease to the effective inhibition of electron–hole recombination within these frameworks. This hypothesis was further supported by TRFL. The average fluorescence lifetimes of **1^Zr^**, **2^Zr^**, and **3^Zr^** were 1.21, 1.04, and 1.04 ns, which were shorter than their corresponding ester monomers of 2.77, 1.84, and 1.80 ns (Figure S11). Additionally, the fluorescence quantum yields decreased markedly from 22.2%, 21.7%, and 21.3% for the ester monomers to 3.9%, 2.1%, and 4.1% for the BMOFs. These findings verified the nonradiative electron transfer from the bodipy backbone to the Zr‐oxo cluster,^[^ ^7^ ^]^ and such electron transfer was further evaluated by in situ electron paramagnetic resonance (EPR).

As shown in Figure 2d, **1^Zr^** after irradiation by visible light for 5 min in N_2_ atmosphere, displays a typical EPR signal (g = 2.0038), confirming the existence of charge separation within the framework.^[^ ^38^ ^]^ Once the air was introduced, the intensity of the signal was reduced, and the signal then rose immediately when TEA was added. In contrast, no EPR signal was detected in the control experiment with the bodipy ester monomer, suggesting insufficient charge separation and the rapid recombination of photoexcited charges. These results from EPR measurements and fluorescence spectra confirmed the effective photoexcited electron transfer from the bodipy backbone to the Zr‐oxo cluster, leading to the reduction of Zr^4+^ to Zr^3+^.^[^ ^7^ ^]^

### Modifying the Bodipy Backbone to Extend the Conjugation System

**Figure 3:**
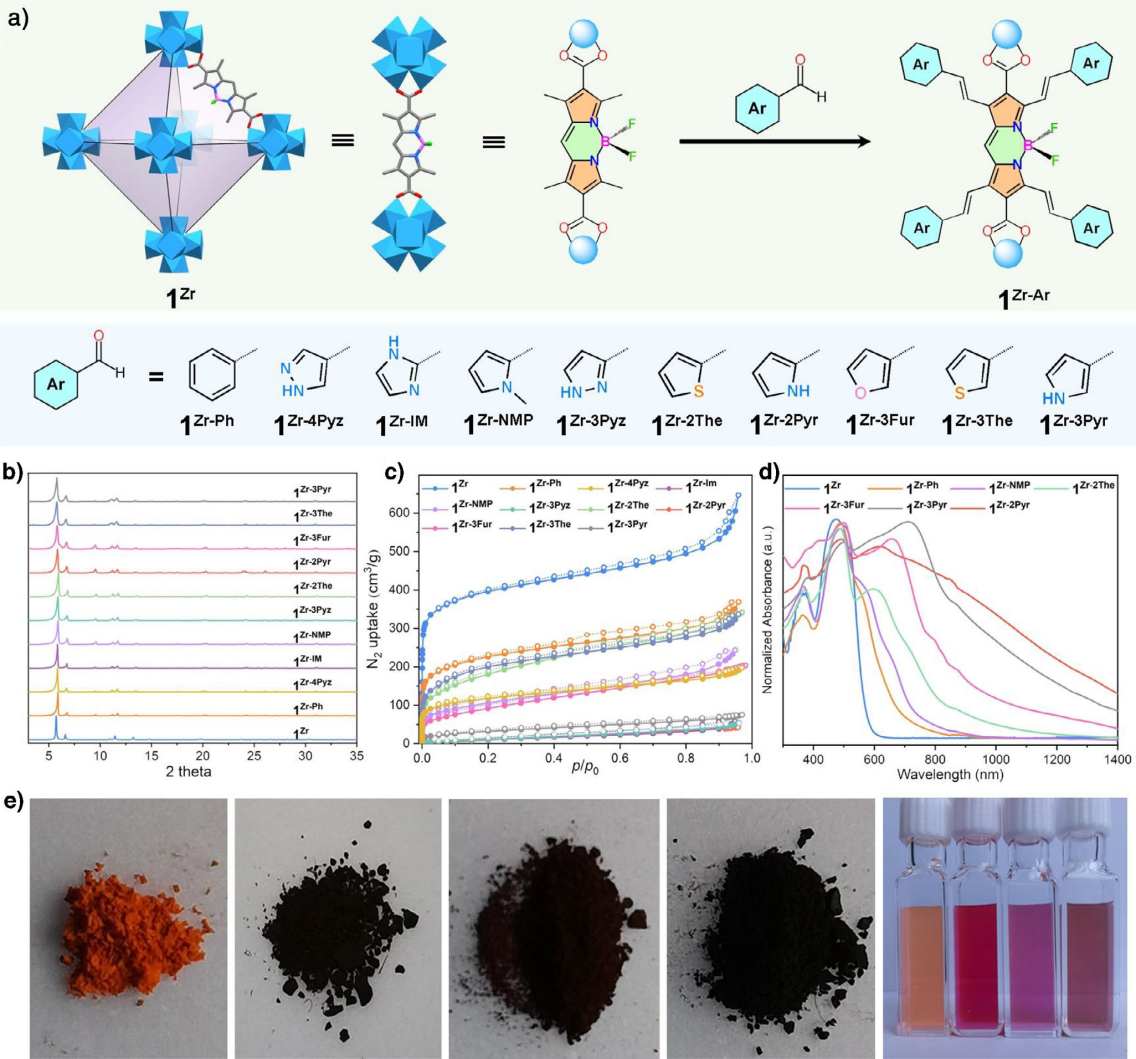
a) Schematic illustration of the modification of the bodipy backbone with various aldehydes to extend the conjugation system. b) PXRD patterns of **1^Zr^** and **1^Zr‐Ar^**. c) N_2_ adsorption measurements of **1^Zr^** and **1^Zr‐Ar^** at 77 K. d) UV–vis spectra of **1^Zr^** and **1^Zr‐Ar^**. e) The optical photographs of **1^Zr^**, **1^Zr‐Ph^**, **1^Zr‐NMP^**, **1^Zr‐2Pyr^**, and the related aqueous suspensions.

Bodipy monomers offer versatile modifications that extend their optical absorption to cover the entire visible spectrum.^[^ ^25^ ^]^ A successful approach involves the utilization of the acidic methyl groups to extend the conjugation system of the backbone.^[^ ^39^, ^40^ ^]^ Given the excellent stability of **1^Zr^** and the acidity of the four methyl groups in the bodipy scaffold, we next introduced the styryl groups into the bodipy scaffold in solid state, so as to achieve the absorption covering the whole visible light region (Figure 3a).

We chose benzaldehyde for the first design. When heating it with **1^Zr^** in the presence of acetic acid and piperidine in toluene, a black powder—termed **1^Zr‐Ph^**—was produced in a quantitative yield. PXRD confirmed that the crystallinity was still maintained (Figure 3b). N_2_ adsorption isotherms reveal a lower uptake, with the BET areas decreasing from 1466 to 809 m^2^ g^−1^ (Figure 3c). Such a decrease in the porosity suggested the successful incorporation of bulky phenyl groups into the backbone and partial occupation of internal porosity by these groups. Since this reaction tends to provide a mixture of mono‐, di‐, tri‐, and tetraphenyl derivatives, we assumed that **1^Zr‐Ph^** might contain a mixture of mono‐, di‐, tri‐, tetra‐, and unsubstituted bodipy scaffolds. Efforts to quantify the amount of functionalized bodipy backbones in **1^Zr‐Ph^** using NMR spectroscopy of the digested sample failed due to the overlapping ^1^H, ^11^B, and ^19^F NMR signals.

In addition to benzaldehyde, we extended our strategy to 2‐formylimidazole (Im), 3‐formylpyrazole (3‐Pyz), 2‐pyrrolecarboxaldehyde (2‐Pyr), 3‐furaldehyde (3‐Fur), N‐methylpyrrole‐2‐carboxaldehyde (NMP), 4‐formylpyrazole (4‐Pyz), 2‐thenaldehyde (2‐The), 3‐thenaldehyde (3‐The), and 3‐pyrrolecarboxaldehyde (3‐Pyr) (Figure 3a and Figures S12–S16). The resulting modified BMOFs were denoted as **1^Zr‐Im^**, **1^Zr‐3Pyz^**, **1^Zr‐2Pyr^**, **1^Zr‐3Pyr^**, **1^Zr‐3Fur^**, **1^Zr‐NMP^**, **1^Zr‐4Pyz^**, **1^Zr‐2The^**, and **1^Zr‐3The^**. PXRD patterns confirmed that all materials maintained their crystallinity after post‐modification (Figure 3b). The BET areas of the modified BMOFs – **1^Zr‐Im^**, **1^Zr‐3Pyz^**, **1^Zr‐2Pyr^**, **1^Zr‐3Pyr^**, **1^Zr‐3Fur^**, **1^Zr‐NMP^**, **1^Zr‐4Pyz^**, **1^Zr‐2The^**, and **1^Zr‐3The^** – were 47, 57, 62, 97, 273, 373, 405, 639, and 634 m^2^ g^−1^, respectively (Figure 3c and Figure S17). Figure S23 shows the ^1^H, ^11^B, and ^19^F NMR signals for all digested BMOFs. Despite several attempts, we were unable to obtain a purified functionalized linker due to the formation of a mixture of mono‐, di‐, tri‐, and tetraphenyl derivatives.

This decrease in the BET area was anticipated due to the introduction of the styryl entities that subsequently block the porosity of the framework. As an illustrative example, we assembled **1^Zr^** from its building blocks by incorporating 3‐Pyr to one branch of the bodipy backbone using a topology‐guided automated algorithm (Figure S55).^[^ ^41^ ^]^ The structure of the assembled framework was optimized first using a force‐field‐based method. Then, GCMC simulations were performed to calculate the N_2_ adsorption isotherm at 77 K (Figure S56 and Section 7 in Supporting Information for details). The simulations were used to calculate the BET area using BETSI,^[^ ^37^ ^]^ revealing an area of 184 m^2^ g^−1^—which was significantly lower than that of the parent framework **1^Zr^**. In practice, since it is likely that more than one branch of the ligand can be modified, the simulation results corroborate our initial hypothesis on reduced pore volume and, therefore, BET area.

Figure S18 shows the TGA curves, Figure 3d and Figure S19a show the UV–vis–IR diffuse reflectance spectra of these multistyryl‐modified BMOFs, demonstrating that the presence of styryl entities led to a bathochromic shift in the absorption edge. Specifically, these multistyryl‐modified BMOFs exhibited two distinct absorption bands with an intrinsic peak of the unmodified bodipy skeleton at ∼500 nm and a new peak emerging in the range of 500–800 nm. With an increase in the electron‐donating capability of the heterocycle, the spectra extended from yellow to red and even to NIR light. Such scenarios were particularly pronounced in the cases of **1^Zr‐3Fur^**, **1^Zr‐3Pyr^**, and **1^Zr‐2Pyr^**, which exhibited a broad, strong absorption in the NIR region. Moreover, the spectral extension was visually corroborated in the optical images of the modified BMOFs, where the corresponding color changed from orange to black (Figure 3e).

We subsequently calculated the optical band gaps based on the Kubelka–Munk formula, which revealed the band gaps of 1.18, 1.41, 1.60, 1.70, 1.77, 1.86, and 1.97 eV for **1^Zr‐2Pyr^**, **1^Zr‐3Pyr^**, **1^Zr‐3Fur^**, **1^Zr‐2The^**, **1^Zr‐3Pyz^**, **1^Zr‐NMP^**, and **1^Zr‐Ph^** (Figure S19b). These results indicated that the band gap could be precisely tailored by the selection of aldehydes. For example, when changing the 3‐pyorrlyethenyl group in **1^Zr‐3Pyr^** to 2‐pyorrlyethenyl in **1^Zr‐2Pyr^**, the band gap was significantly narrowed from 1.41 to 1.18 eV. Interestingly, two similar pyrrolylethenyl‐modified **1^Zr‐2Pyr^** and **1^Zr‐NMP^** elicited changes in the absorption spectra, whereas the absorption of **1^Zr‐NMP^** exhibited a blue shift to ∼120 nm as compared to **1^Zr‐2Pyr^**. Further, introducing an additional N atom at any position of the pyrrole heterocycle, such as the imidazolyethenyl‐modified **1^Zr‐Im^** and pyrazolyethenyl‐modified **1^Zr‐3Pyz^**, resulted in ∼140 nm blue shift relative to **1^Zr‐3Pyr^**. Mott–Schottky measurements showed that the character of the n‐type semiconductor was well preserved, with LUMO levels determined to be −0.43, −0.36, −0.34, −0.47, −0.50, −0.41, and −0.30 V versus NHE, and subsequently, the HOMO levels were calculated to be 0.75, 1.05, 1.26, 1.23, 1.27, 1.45, and 1.67 V versus NHE for **1^Zr‐2Pyr^**, **1^Zr‐3Pyr^**, **1^Zr‐3Fur^**, **1^Zr‐2The^**, **1^Zr‐3Pyz^**, **1^Zr‐NMP^**, and **1^Zr‐Ph^**, respectively (Figure S20).

Light‐absorbing materials covering the entire visible light spectrum, especially those with absorption beyond 800 nm, have attracted considerable attention due to their potential applications in optoelectronics and biology‐related fields.^[^ ^42^, ^43^ ^]^ Numerous pioneering studies have focused on the synthesis of these materials, including organic molecules,^[^ ^44^ ^]^ inorganic semiconductors,^[^ ^45^ ^]^ amorphous porous organic polymers,^[^ ^46^, ^47^ ^]^ covalent organic frameworks (COFs),^[^ ^48^, ^49^, ^50^ ^]^ and MOFs.^[^ ^38^, ^51^ ^]^ However, only a few examples have achieved full‐color absorption, and most of them either require complex synthesis procedures or easily suffer from photobleaching under light irradiation. These challenges lead to high costs and performance degradation in practical applications. In contrast, our **1^Zr‐Ar^** can conveniently tune the absorption edge by a rational selection of aldehydes with different electron‐donating capabilities, thereby achieving full‐color absorption with the absorption edge even extending into the NIR and IR regions. Unlike the complex methods required to extend the π‐conjugation system in organic molecules and MOF/COF building blocks, we offer a simple and direct strategy to adjust the absorption range.

### Modulation of the Metal Cluster via Post‐Synthetic Solvent‐Assisted Metal Exchange

**Figure 4:**
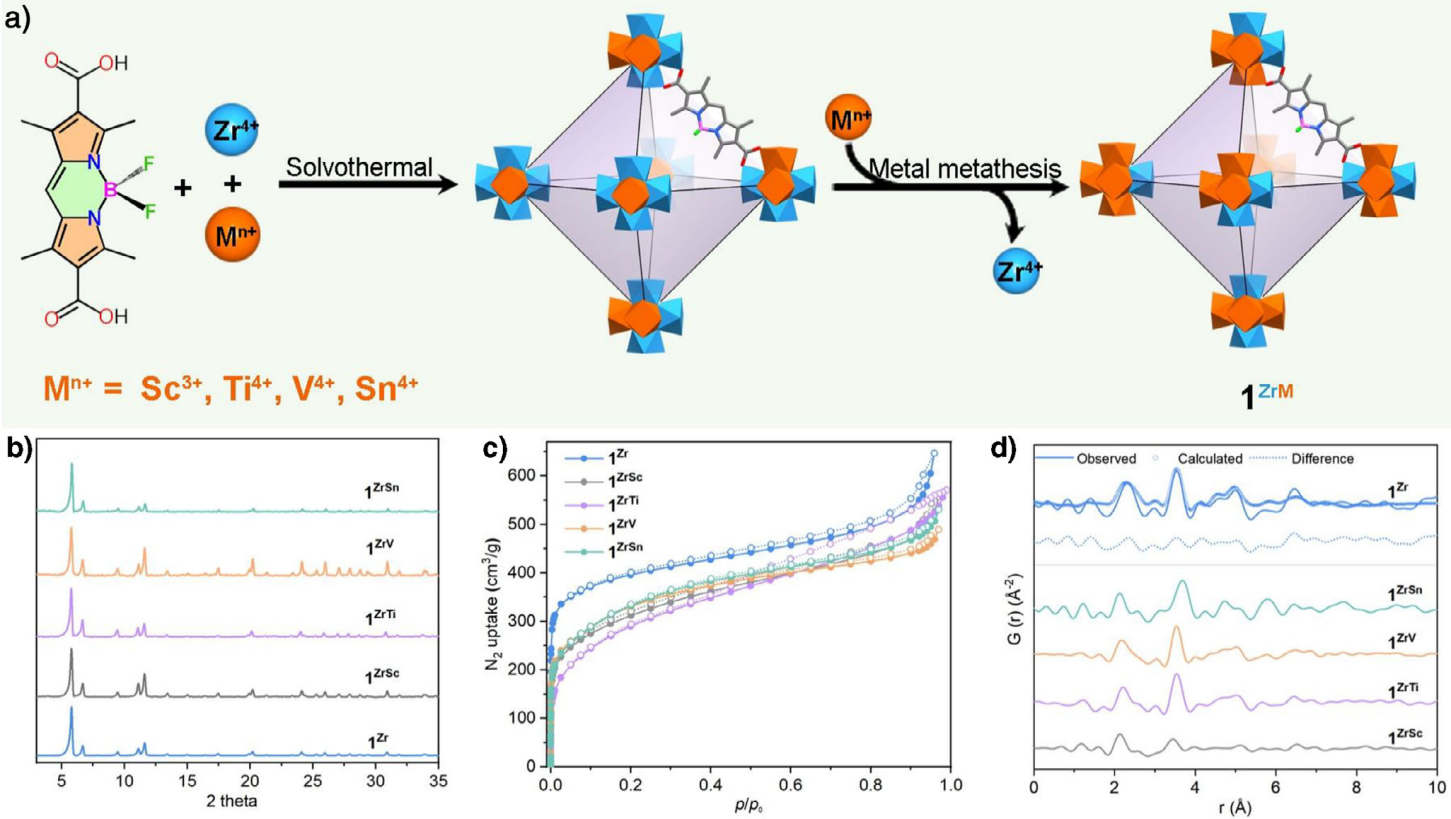
a) Mixed‐metal BMOFs synthesis strategy. b) PXRD patterns and c) 77 K N_2_ adsorption isotherms of **1^ZrM^**. d) Low‐*r* region of the PDFs measured for **1^Zr^**, **1^ZrTi^**, **1^ZrSc^**, and **1^ZrSn^**. PDF simulation from the CIF of **1^Zr^**.

Partial substitution of the MOF intrinsic metal ions with exogenous metal ions has been recognized as another attractive approach to improve photocatalytic performance.^[^ ^18^ ^]^ In this context, to achieve mixed metal ions in our BMOFs, we synthesized four polymetallic variants: **1^ZrSc^**, **1^ZrTi^**, **1^ZrV^**, and **1^ZrSn^**, through the combination of direct synthesis and post‐synthetic solvent‐assisted metal exchange (PSME) (Figure 4a). Initially, four binary BMOFs (**1^ZrSc^**, **1^ZrTi^**, **1^ZrV^**, and **1^ZrSn^**) were prepared by reacting ZrCl_4_ with metal chlorides (ScCl_3_, TiCl_4_, VCl_4_, or SnCl_4_) in a DMF solution containing H_2_THDFB and modulators (FA or acetic acid) at 80 °C for 24 h. ICP‐MS showed that molar ratios of **B**/**Zr**/**M** in these binary frameworks were 1:0.87:0.13 for **1^ZrSn^**, 1:0.67:0.33 for **1^ZrSc^**, 1:0.63:0.37 for **1^ZrTi^**, and 1:0.70:0.30 for **1^ZrV^**. Notably, the metal ratios showed minimal sensitivity to changes in the initial feed ratios. For instance, varying the feed ratio of ZrCl_4_/TiCl_4_ from 2:1 to 1:1 and 2:3 did not change the composition of **1^ZrTi^**. However, an excessive amount of TiCl_4_ led to a decrease in yield and impacted phase purity.

Subsequently, to further increase the content of exogenous ions within **1^ZrM^**, we performed PSME on the pre‐synthesized binary BMOFs by immersing **1^ZrM^** in a DMF solution of the corresponding metal chloride (Figure 4a). Optimal conditions were repeating this process for 10 cycles with solution changes every 12 h. During this process, acetic acid played a crucial role in preventing the hydrolysis of metal ions. Figure S24 displays the kinetic results of the PSME monitored by ICP‐MS. The molar percentage of Zr ions decreased after 10 solution exchanges, with ca. 83%, 87%, 50%, and 46% of Zr ions replaced by Sc, Ti, V, and Sn ions, respectively. The final molar ratios of **B/Zr/M** in the frameworks were 1:0.17:0.83 for **1^ZrSc^**, 1:0.13:0.87 for **1^ZrTi^**, 1:0.50:0.50 for **1^ZrV^,** and 1:0.54:0.46 for **1^ZrSn^**, suggesting that up to 87% of Zr ions in **1^Zr^** could be replaced, and the exogenous metal ions in the resulting binary **1^ZrM^** could be controlled from 17%−87% by controlling the exchange times from 1 to 10. The chemical formulae of the final **1^ZrM^** were (Zr_1‐_ *_x_* M*_x_*)_6_O_4_(OH)_4_(**L1**)_6_), *x* = 0.83 for M = Sc, *x* = 0.87 for M = Ti, *x* = 0.50 for M = V, and *x* = 0.46 for M = Sn, respectively, where *x* represented the average number of the exogenous metal ions per metal‐oxo cluster.

PXRD patterns of these four **1^ZrM^** aligned well with **1^Zr^** (Figure 4b). N_2_ adsorption isotherms showed slight changes within four **1^ZrM^**, with the calculated BET areas ranging from 1023 to 1076 m^2^ g^−1^ (Figure 4c). SEM analysis revealed that the partial metal replacements did not affect the crystal morphology (Figure S27). SEM‐EDS mapping demonstrated a uniform distribution of Zr and relevant metal within a whole crystal, indicating a homogeneous crystalline framework rather than physical mixtures of homoleptic series. PXRD and TGA measurements indicated similar thermal stability compared to **1^Zr^** (Figures S25 and S28). Moreover, the partial replacement of Zr^4+^ in the metal cluster by Sc^3+^, Ti^4+^, V^4+^, or Sn^4+^ did not significantly influence the water and chemical stability. Solid‐state UV–vis diffuse reflectance spectra of the four **1^ZrM^** showed similar adsorption and possessed the feature of the π–π* transition of the bodipy backbone in the region of 350–600 nm with the maxima absorption (λ_max_) at 511 nm (Figure S29a). The band gaps were 2.30 ± 0.03 eV (Figure S29b). Mott–Schottky measurements revealed that they were all n‐type semiconductors with the LUMO levels at −0.36, −0.39, −0.47, and −0.41 V versus NHE for **1^ZrSc^**, **1^ZrTi^**, **1^ZrV^**, and **1^ZrSn^**. The HOMO levels were then calculated to be 1.96, 1.88, 1.83, and 1.86 V versus NHE, respectively (Figure S30). XPS measurements suggested that the valence state of Sc, Ti, V, and Sn was preserved in the related **1^ZrM^** (Figure S33). Figure S31 shows the solid‐state PL spectra of **1^ZrM^,** and Figure S32 shows the corresponding fluorescence lifetimes.

We then performed X‐ray absorption spectroscopy (XAS) at the Ti, Sc, and Sn K‐edges in **1^ZrTi^**, **1^ZrSc^**, and **1^ZrSn^**. Extended X‐ray absorption fine structure (EXAFS) showed that the Ti–O peak of **1^ZrTi^** was located at ∼1.6 Å, suggesting Ti occupied the Zr position in the pristine **1^Zr^**. The EXAFS fitting result displayed that **1^ZrTi^** could be well fitted by using both Ti–O and Ti–Ti (or Ti–Zr) shells with average lengths of ∼2.07 and ∼3.48 Å (Figure S34 and Table S2), respectively. It should be noted that the average Ti–O and Ti–Ti (or Ti–Zr) distances were close to the Zr–O and Zr–Zr distances in **1^Zr^**. In terms of **1^ZrSc^** and **1^ZrSn^**, a model containing two substituted metal centers at opposite ends of the Zr_6_ cluster was found to best fit the EXAFS data (Figures S35 and S36, see Supplemental Information Section S5 for details). We also resorted to atomic pair distribution function (PDF) analysis to provide atomic‐level structural information. As shown in Figure 4d, these four binary MOFs displayed a strong similarity to the PDF features of **1^Zr^**. The main peaks at 2.2, 3.5, and 5.0 Å corresponded to the M–O and M–M distances inside one metal cluster. This indicated that the metal clusters still maintained good integrity in our binary MOFs. We believed that any site or amount of Zr atoms in each Zr_6_ cluster could be replaced (see the DFT analysis in Supporting Information, Section 7.5), resulting in a random distribution of metal clusters with one to six Zr atoms replaced alongside unsubstituted Zr clusters throughout the whole framework. However, at this time, the precise positions and quantities of the exogenous atoms within each cluster are still not accurately resolved and will be continuously addressed in the future by focusing on the synthesis of these binary single crystals for SC‐XRD.

### Heterogeneous Photocatalysis

Considering the attractive optical characteristics of BMOFs, which could be finely tailored to absorb light that covers the full solar spectrum, we further explored their heterogeneous photocatalytic performances under visible light.^[^ ^6^, ^52^, ^53^ ^]^ α‐Amino amides and their derivatives are crucial structural motifs that widely exist in clinical drugs, functional proteins, and complex natural products.^[^ ^54^ ^]^ Among various available synthetic methodologies, direct C(sp^3^)–H carbamoylation via photocatalysis has emerged as a promising approach for synthesizing α‐amino amides and their derivatives.^[^ ^55^ ^]^

To begin with, **1^Zr^** was first chosen as the model photocatalyst. A mixture of 1.0 equiv of 1‐phenylpyrrolidine **6a**, 1.2 equiv of *tert*‐butyl isocyanide **7a**, and 1.0 equiv of FA in chloroform was irradiated by a 5 W white LED lamp at room temperature in air. With 1.0 mol% loading of **1^Zr^**, the reaction yielded the target amide **8a** with 73% yield in 8 h. Moreover, this amidation reaction also proved feasible under natural sunlight. For example, product **8a** was obtained with a 57% conversion after exposure to direct sunlight from 9:00 am to 5:00 pm. It should be noted that this sunlight‐driven reaction was weather‐dependent, achieving a maximum conversion of 67% on a sunny day and dropping to 38% on a cloudy day (Figures S39a and S40a).

**Figure 5:**
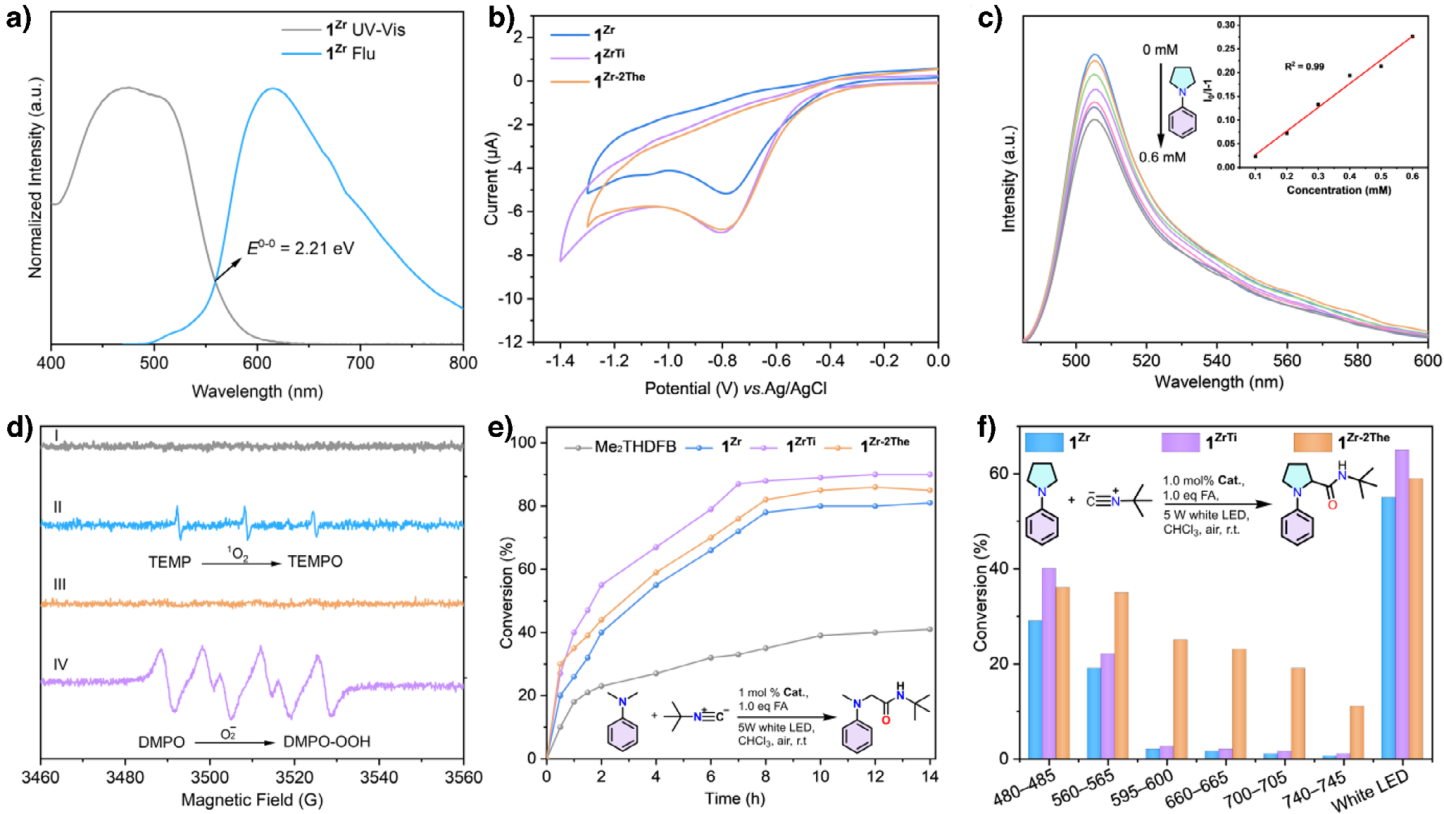
a) Normalized absorption (black line) and emission spectra (blue line) of **1^Zr^**, excited at 500 nm. b) Solid‐state CV of **1^Zr^**, **1^ZrTi^**, and **1^Zr‐2The^** with a scan rate of 10 mV s^−1^ in the range of −1.5–0 V. c) Fluorescence quenching experiment of **1^Zr^** (10 µmol L^−1^) by increasing concentration of 1‐phenylpyrrolidine **6a** in chloroform; the inset represents the Stern–Volmer plot of **1^Zr^** versus the concentration of **6a**. d) ESR spectra of **1^Zr^** particles suspended in pure MeOH (I, III) and a MeOH solution of substrate **6a** (II, IV) in the presence of TEMP/DMPO under irradiation for 2 min. e) Kinetic curves provided by **1^Zr^**, **1^ZrTi^**, **1^Zr‐2The^** and Me_2_THDFB. The mass loading of **1^Zr‐2The^** was the same as **1^Zr^**. f) **8a** promoted by monochromatic and white lights within 4 h. The mass loading of **1^Zr‐2The^** was also the same as **1^Zr^**.

Control experiments revealed that light, photocatalyst, FA, and O_2_ were all indispensable for this amidation transformation. Electron‐spin resonance (ESR) experiments revealed that O_2_ was activated into O_2_^•−^ and ^1^O_2_, with no ^•^OH signal detected in this photocatalytic system (Figure 5d). Solid‐state electrochemical measurement of **1^Zr^** displayed a redox potential of −0.59 V versus NHE that corresponded to the **1^Zr^**/**1^Zr^** ^−^ couple (Figure 5b). Excited state energy *E* ^0−0^ of **1^Zr^**, determined from the intersection of the absorption and fluorescence spectra at 560 nm, was calculated to be 2.21 eV (Figure 5a). Additionally, the oxidation potential *E*_ox_ of **6a** was measured to be 0.77 V (Figure S41). Therefore, the free energy change for the electron transfer from **6a** to the excited state **1^Zr^** * was calculated to be −0.85 eV, confirming that this electron transfer process was thermodynamically feasible. The fluorescence titration experiment, with a quenching constant *K* = 499.1 M^−1^, further confirmed electron transfer from **6a** to **1^Zr^** * within this photocatalytic system (Figure 5c).

Based on the results obtained and relevant literature,^[^ ^34^, ^55^ ^]^ we proposed a plausible reaction mechanism. As shown in Figure S43, under visible light irradiation, the bodipy scaffold in **1^Zr^** acted as a light‐harvesting antenna, absorbing visible light and transitioning to its excited state. The excited electron was then transferred to the Zr‐4d orbital via ligand‐to‐metal charge transfer (LMCT). This process facilitated the reduction of O_2_ to O_2_^•–^, leaving the hole on the bodipy scaffold to oxidize 1‐phenylpyrrolidine **6a** to the aminyl cation radical **6a** ^+•^. The superoxide radical anion O_2_^•–^ then seized a proton from HCOOH to form the ^•^O_2_H, which further oxidized the active species **6a** ^+•^ to give the iminium cation **6a** ^+^ intermediate, along with the generation of H_2_O_2_. The iminium cation **6a** ^+^ intermediate was subsequently attacked by isocyanide to form a nitrilium ion intermediate, which was further hydrolyzed by water to afford the amide **8a**. Additionally, we confirmed that the presence of the ^1^O_2_ was also responsible for this photooxidation, as the bodipy unit was a competent chromophore for the photogeneration of ^1^O_2_ (Figure 5d and Figure S45). The reactant **6a** could also be oxidized by ^1^O_2_ to the iminium cation **6a** ^+^. This proposed mechanism was further supported by the homogeneous reaction, in which **8a** could be synthesized in 35% conversion with the presence of the Me_2_THDFB (Figure 5e).

We then evaluated the generality of **1^Zr^** by examining the substrate scope of saturated aza‐heterocycles **6b–s** and isocyanides **7a–f**. As shown in Table 1, *N*‐aryl pyrrolidines bearing electron‐rich (**6b, 6d, 6e, 6** **g, 6** **h**) or electron‐deficient (**6c, 6f, 6i–l**) substituents on the *para*, *meta*, and *ortho* positions of the aromatic ring were amenable to this cross‐coupling protocol, and the photocatalyst **1^Zr^** provided the corresponding amino amides (**8a–l**) in 68%–87% yields. Furthermore, the substrates **6m–o** bearing two substituents also proceeded smoothly to afford **8m–o** in 69%–81% yields. More excitingly, the effect of the aza‐heterocycle moiety seemed not to influence this carbamoylation process. For example, the six‐, seven‐membered cyclic amine (**6p **− **r**) substrates, and even the ring‐opening substrate *N*,*N*‐dimethylaniline (**6s**), could be used to include an amide bond to obtain the targeted *α*‐amino amides in high yields (**8p** 71%, **8q** 76%, **8r** 78%, and **8s** 75%).

**Table 1:** Direct C(sp^3^)−H carbamoylation of saturated aza‐heterocycles photocatalyzed by **1^Zr^**.^a)^, ^b)^

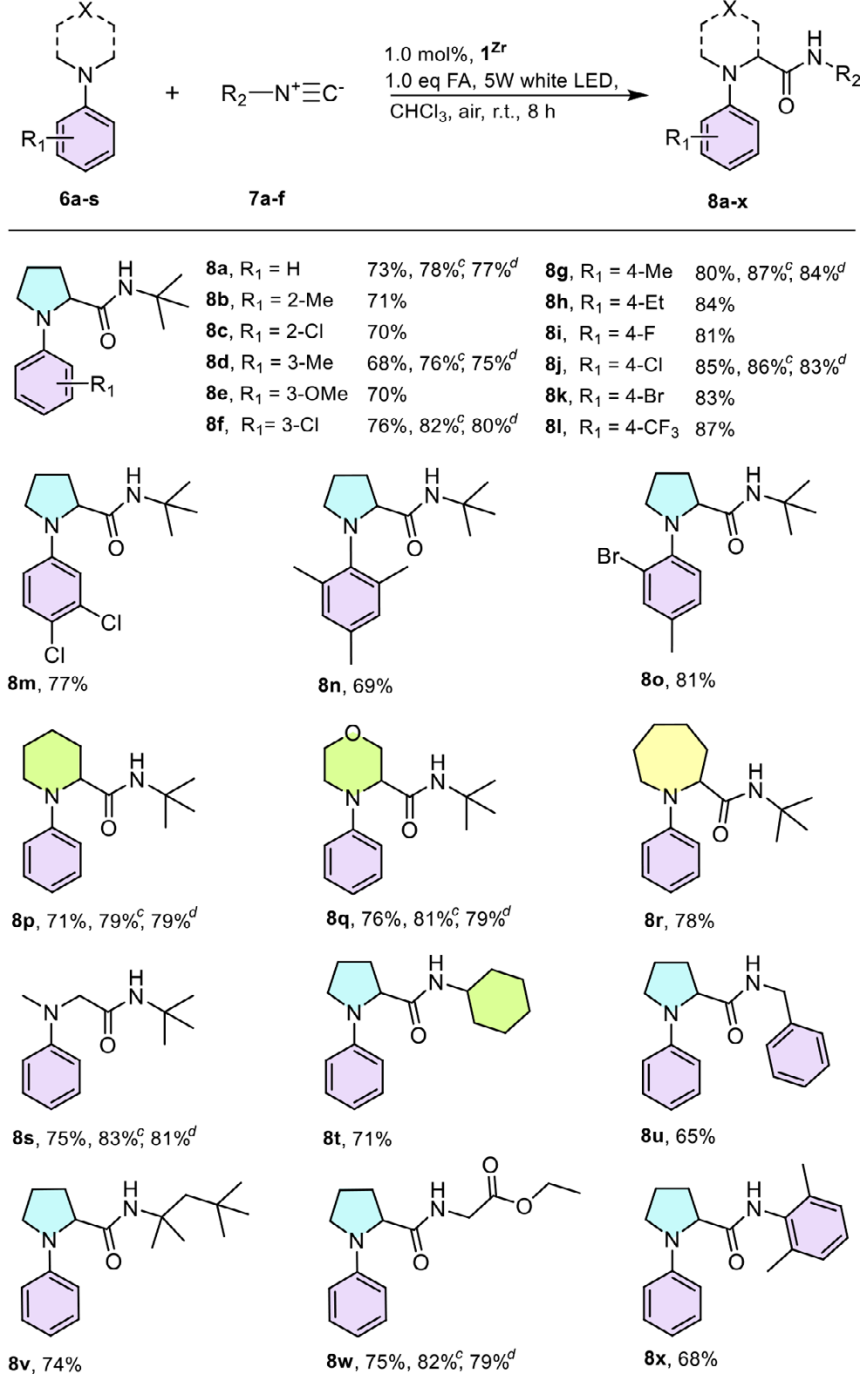

^a)^ Reaction conditions: aza‐heterocycles **6** (0.5 mmol), isocyanides **7** (0.6 mmol), formic acid (FA, 0.5 mmol), **1^Zr^** (1.0 mol% based on **6**), CHCl_3_ (1 mL), 5 W white LED lamp as the light source, room temperature in air for 8 h. The photocatalyst loading was based on the bodipy unit in **1^Zr^**.

^b)^ Isolated yield.

^c)^ Catalyzed by **1^ZrTi^**.

^d)^ Catalyzed by the combination of **1^ZrTi^** and **1^Zr‐The^**.

Other BMOFs, including **2^Zr^**, **3^Zr^**, the multistyryl‐modified **1^Zr‐Ar^**, and the metal‐exchanged **1^ZrM^**, were evaluated for this transformation. With **6a** as a model substrate, the conversions to **8a** provided by **2^Zr^** and **3^Zr^** were 77% and 78%, respectively, which are nearly identical to the 78% conversion observed with **1^Zr^** (Table S7), suggesting the substituent at the *meso*‐position bodipy backbone has minimal impact on the photocatalytic performance. Similarly, the performance of these ten multistyryl‐modified BMOFs exhibited minor differences. Among them, **1^Zr‐2The^** and **1^Zr‐4Pyz^** achieved optimal conversions of up to 87%. **1^Zr‐NMP^**, **1^Zr‐3The^**, **1^Zr‐Im^**, and **1^Zr‐3Pyz^** exhibited lower performance. The electron‐rich heterocycle‐modified BMOFs, such as **1^Zr‐2Pyr^**, **1^Zr‐3Pyr^**, and **1^Zr‐3Fur^**, showed slightly lower activity compared to their electron‐deficient counterparts like **1^Zr‐Im^**, **1^Zr‐3Pyz^**, and **1^Zr‐4Pyz^**. More specifically, the conversions afforded by **1^Zr‐Im^** and **1^Zr‐3Pyz^** were 86% and 85%, respectively, while **1^Zr‐2Pyr^**, **1^Zr‐3pyr^**, and **1^Zr‐3Fur^** provided 80%, 81%, and 80%, respectively. In the cases of the metal‐exchanged **1^ZrM^**, the binary **1^ZrTi^** and **1^ZrSn^** exhibited improved photocatalytic efficiencies, especially for **1^ZrTi^**, with conversion as high as 89%. In addition, we found that when the Ti in **1^ZrTi^** reached 53%—after one PSME treatment—the conversion could reach up to 89%, and further increasing Ti had little effect on the improvement (Table S8).

Given the excellent chemical stability and photostability of **1^Zr^**, **1^Zr‐2The^**, and **1^ZrTi^** (Figures S7, S22, S25, S26, and S37), we then selected three representatives for a more detailed investigation. The reaction kinetics for the conversion of **6a** to **8a** were first investigated and displayed in Figure 5e. Under identical conditions, **1^ZrTi^** and **1^Zr‐2The^** exhibited faster initial reaction rates than **1^Zr^**, greatly surpassing the homogenous Me_2_THDFB. Specifically, the conversions of **8a** over 8 h period were 88%, 82%, and 78% for **1^ZrTi^**, **1^Zr‐2The^**, and **1^Zr^**. In contrast, only 35% of conversion was obtained for Me_2_THDFB. To further study the effect of light wavelength on photocatalysis, we utilized monochromatic LED lamps at wavelengths of 480 (blue), 560 (green), 600 (orange), 660 (red), 700 (crimson), and 740 (red‐purple) nm. As shown in Figure 5f, the light wavelength significantly influenced the reaction efficacy. When using blue and green lights for 4 h, **8a** was produced in 41% and 22% by **1^ZrTi^**, 29% and 19% by **1^Zr^**, and 36% and 35% by **1^Zr‐2The^**. As the wavelength increased, the conversions decreased linearly, with almost no product detected by **1^Zr^** and **1^ZrTi^** upon irradiation over 660 nm. In contrast, **1^Zr‐2The^** maintained conversion rates of 25%, 23%, 19%, and 11% under the wavelengths of 600, 660, 700, and 740 nm, respectively. These results highlighted that extending the π‐conjugation in the bodipy core was crucial for improving photocatalytic performance across different light wavelengths.

**1^ZrTi^** achieved the highest conversions under the white LED light. We attributed it to the presence of the Ti ions, which could facilitate more favorable charge transfer from the excited bodipy scaffold to the Ti site, forming Ti^3+^ within the metal cluster.^[^ ^10^, ^17^ ^]^ This hypothesis was supported by in situ EPR spectroscopy, where **1^ZrTi^** showed an additional signal at g = 1.935 (Figure S46), and this signal was not detected in the pristine counterpart **1^Zr^** (Figure 2d), suggesting the presence of paramagnetic Ti^3+^ species in **1^ZrTi^**.^[^ ^10^, ^56^, ^57^, ^58^ ^]^ Due to the electronic state overlap between Zr and Ti atoms, the resulting Ti^3+^ acted as a mediator to donate the electron to Zr^4+^ to form the photocatalytically active species Zr^3+^.^[^ ^17^, ^18^ ^]^ As such, the presence of the trapping electron state (Ti^3+^) in **1^ZrTi^** allowed for more efficient charge transfer, thus enhancing the photocatalytic efficiency in the visible light region.

The cleavage of the C─N bond has attracted a great deal of attention in organic synthesis and biological metabolic processes. Among various C─N bond cleavage methods, the C─H bond activation‐triggered C─N bond cleavage is of significant synthetic interest since it constitutes another good strategy to deliver the amides.^[^ ^15^ ^]^ Considering that the aforementioned tertiary amine (e.g., 1‐phenylpyrrolidine **6a**) could be oxidized to the iminium cation intermediate, we envisioned that the resulting iminium cation intermediate would be prone to hydrolyze to form the secondary amine and further react with acyl chloride to afford the desired amide. To demonstrate the broader capabilities of our photocatalysts, we continued to showcase the challenging unreactive C─N bond activation of *N*,*N*‐dimethylanilines promoted by visible light.

We performed this amide synthesis using *N*, *N*‐dimethylaniline (**9a**) and benzoyl chloride (**10a**) as model substrates, with **1^Zr^** as the photocatalyst. After rigorous optimization of reaction conditions, we found that this photocatalytic transformation achieved a 45% conversion in THF under the irradiation of a 5 W white LED lamp in the air. This conversion could be further improved to 73% by increasing the reaction temperature to 45 °C with the presence of pentafluoronitrobenzene (PFNB) and KHCO_3_. Control experiments confirmed that light, photocatalyst, PFNB, and KHCO_3_ were all necessary for this transformation.

With the optimal reaction condition established, the scope of this reaction was evaluated using various *N*,*N*‐dimethylaniline and acyl chloride combinations. We first examined the generality and functional group tolerance of the aniline moiety. As shown in Table 2, our bodipy‐based light‐driven system effectively coupled a wide range of *N*, *N*‐dimethylanilines (**9a–k**), giving the corresponding amide products in 70%–82% yields. Next, the scope of acyl chlorides was further explored. A variety of aroyl chlorides bearing electron‐rich (**10a–f**) or electron‐deficient (**10g–k**) substituents on the *para*, *meta*, and *ortho* positions of benzene ring smoothly reacted with *N*,*N*‐dimethylaniline **9a** to deliver the corresponding amides with moderate to high yields (63%–86%). Additionally, heteroaromatic acyl chlorides (**10o**, **10p**), 2‐naphthyl chloride (**10q**), olefin‐derived acyl chlorides (**10r**, **10s**, and **10t**), and even sulfonyl chlorides (**10u**, **10v**, and **10w**) were tolerant to this *N*‐amidative demethylation. However, aliphatic chlorides and acid anhydrides were unsuitable for this transformation, as no amides were detected. Sunlight was also feasible for this transformation, achieving a 46% conversion in the synthesis of **11aa** (Figures S39b and S40b). The heterogeneity of our bodipy‐based photocatalyst was then evaluated. **1^Zr^** could be recovered by centrifugation and reused for at least five runs without any decrease in the activity (Figure S49). PXRD patterns, solid‐state UV–vis and PL spectra of the recovered sample remained consistent with those of the freshly prepared counterpart (Figures S50 and S52), with a BET area of 917 m^2^ g^−1^ for **1^Zr^** (Figure S51). ICP‐MS analysis of the filtrate revealed almost no leaching of Zr ions (<0.05%).

**Table 2:** Photocatalytic Dealkylation/Acylation of Tertiary Amines to Access Amides.^a)^, ^b)^

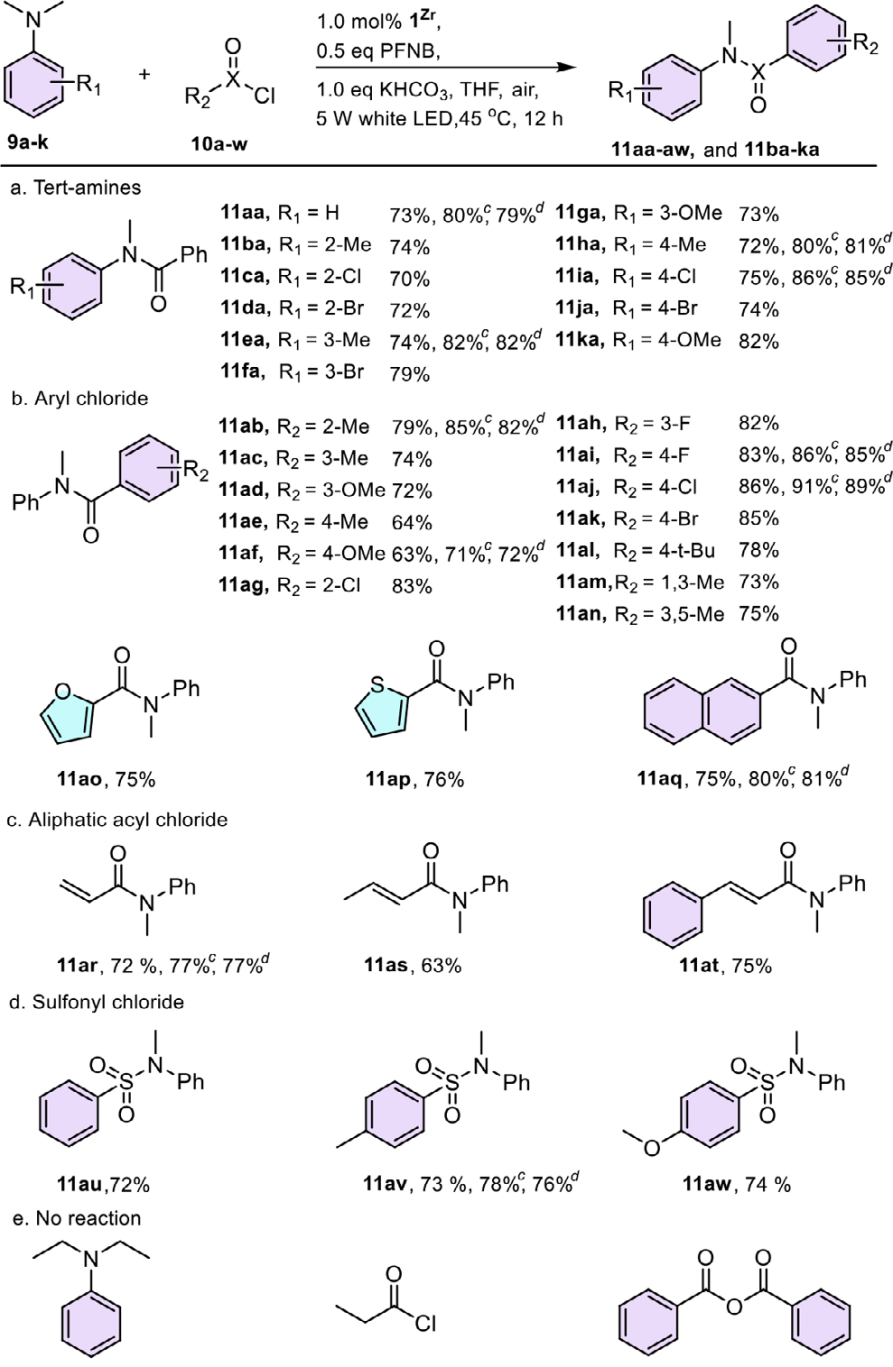

^a)^ Reaction conditions: *N*,*N*‐dimethylanilines **9** (0.5 mmol), acyl chlorides **10** (0.6 mmol), KHCO_3_ (0.6 mmol), **1^Zr^** (1.0 mol% based on **9**), PFNB (0.5 equiv.), THF (1 mL), 5 W white LED lamp as the light source, 45 °C in air for 8 h. The photocatalyst loading was based on the bodipy unit in **1^Zr^**.

^b)^ Isolated yield.

^c)^ Catalyzed by **1^ZrTi^**.

^d)^ Catalyzed by the combination of **1^ZrTi^** and **1^Zr‐The^**.

Several strategies have been made to investigate the generation of reactive iminium cation **6a** ^+^ and/or **9a** ^+^. However, most of them either relied heavily on strong oxidants such as KMnO_4_,^[^ ^59^ ^]^ or were restricted to specific oxidant combinations like I_2_/TEMPO and Fe^2+^/TBHP.^[^ ^60^, ^61^, ^62^ ^]^ Although there were reports of using photocatalysis, they were limited to non‐recyclable photocatalysts such as Rose Bengal or CzIPN photocatalysts.^[^ ^63^, ^64^ ^]^ Additionally, we evaluated our photocatalyst against some well‐known solid photocatalysts, including UiO‐66,^[^ ^65^ ^]^ UiO‐66‐NH_2_,^[^ ^66^ ^]^ MIL‐125,^[^ ^58^ ^]^ MIL‐125‐NH_2_,^[^ ^67^ ^]^ PCN‐224/MOF‐545,^[^ ^68^, ^69^ ^]^ NU‐1000,^[^ ^70^ ^]^ and porphyrin COFs DhaTph,^[^ ^71^ ^]^ in synthesizing amides **8a** and **11aa**. As shown in Figure S53, although UiO/MIL catalysts displayed lower conversions for both **8a** and **11aa**, PCN‐224/MOF‐545 and NU‐1000 achieved moderate conversions of 67% and 42% for **8a**, and 61% and 35% for **11aa**, respectively. Despite some efficacy, these were less effective compared to our bodipy photocatalyst, particularly under natural sunlight.

## Conclusions

We reported the design and synthesis of three highly stable UiO‐type, Zr‐based BMOFs (**1^Zr^**, **2^Zr^**, and **3^Zr^**) using dicarboxyl‐functionalized bodipy ligands. By taking advantage of the acidity of the methyl groups on the bodipy backbone, the condensation reaction of **1^Zr^** with a range of aldehydes in solid state led to an absorption covering the whole visible light range, and with the band‐edge absorption extending into the NIR region. Beyond this, the Zr^4+^ ions in the metal cluster could be partially exchanged with other exogenous metal ions, including Sc^3+^, Ti^4+^, V^4+^, and Sn^4+^. The periodically aligned bodipy scaffolds within the channels allowed **1^Zr^** to behave as an optimal photocatalyst for both C–H carbamoylation of saturated aza‐heterocycles and dealkylation/acylation of *N*,*N*‐dimethylanilines to provide amides under visible light irradiation. Post‐synthetic modification of **1^Zr‐2The^** and **1^ZrTi^** improved photocatalytic activities. Through this work, we have advanced BMOFs as promising platforms for heterogenous photocatalytic transformations. We anticipate that strategies where both the ligand and the metal cluster are rationally modulated to exert fine control over MOF characteristics will pave a new way for a series of other fundamental applications, including gas storage/separation, catalysis, bioimaging, and even perovskite solar cells.

## Conflict of Interests

The authors declare no conflict of interest.

## Supporting information



Supporting Information

Supporting Information

## Data Availability

The data that support the findings of this study are available from the corresponding author upon reasonable request.
