# Supplementary material for: Engineering Bodipy‐Based Metal–Organic Frameworks for Efficient Full‐Spectrum Photocatalysis in Amide Synthesis
Source: Angew Chem Int Ed Engl. 2025 Apr 14;64(24):e202505405. doi: 10.1002/anie.202505405 (PMC12144902; doi:10.1002/anie.202505405)
Supplement: Supplementary file 1 — Supporting Information [file ANIE-64-e202505405-s002.pdf]

Supporting Information for:

Engineering Bodipy-Based Metal−Organic Frameworks for Efficient Full-Spectrum Photocatalysis in Amide Synthesis

Binhui Liu,[1,§] Xu Chen,[2,§] Yuhao Yang,[1] Mohammad Reza Alizadeh Kiapi,[2] Dhruv Menon,[2] Qianyi Zhao,[1] Guozan Yuan,[3,*] Luke L. Keenan,[4] David Fairen-Jimenez,[2,*] and Qingchun Xia,[1,*]

[1]Henan Key Laboratory of Boron Chemistry and Advanced Energy Materials, School of Chemistry and Chemical Engineering, Henan Normal University, Xinxiang, Henan 453007, China.

[2]The Adsorption & Advanced Materials Laboratory (A[2]ML), Department of Chemical Engineering & Biotechnology, University of Cambridge, Philippa Fawcett Drive, Cambridge CB3 0AS, UK.

[3]School of Chemistry and Chemical Engineering, Anhui University of Technology, Ma'anshan, Anhui 243032, China.

[4]Diamond Light Source Ltd., Harwell Science and Innovation Campus, Chilton, Didcot OX11 0DE, UK.

[*]e-mails: guozan@ahut.edu.cn; df334@cam.ac.uk; xiaqingchun@htu.edu.cn

[§]B.L and X.C. contributed equally.

Table of Contents

1 Material and general procedures S2
2 Synthesis S2
3 Experimental procedure for heterogeneous photocatalysis. S7
4 Mott−Schottky plot measurements S8
5 X-ray absorption fine structure (XAFS) spectroscopy S9
6 Atomic pair distribution function (PDF) analysis S10
7 Characterization S12
8 Modelling Details S39
9 NMR S47
10 BET areas calculation using BETSI S104
11 References S127

1 Materials and general procedures

All reagents and solvents used in these studies are commercially available and used without further purification.

Single-Crystal X-ray Diffraction. Single-crystal XRD data for $\mathbf{1^{Zr}}$, $\mathbf{2^{Zr,}}$ and $\mathbf{3^{Zr}}$ were collected on a SuperNova X-ray diffractometer with Cu-Kα radiation (λ = 1.54178 Å) at 150 K. We have collected several datasets for $\mathbf{1^{Zr}}$, $\mathbf{2^{Zr}}$ and $\mathbf{3^{Zr}}$ using Cu-Kα radiation. Among the several datasets, the best data set was used for structure solution and refinement. The empirical absorption correction was applied using the SADABS program (G. M. Sheldrick, SADABS, program for empirical absorption correction of area detector data; University of Göttingen, Göttingen, Germany, 1996).

The structure was solved by direct methods with SHELXS-2018 and refined with SHELXL-2018 using OLEX 1.2. In the structure, all the non-hydrogen atoms except guest molecules were refined by full-matrix least-squares techniques with anisotropic displacement parameters, and the hydrogen atoms were geometrically fixed at the calculated positions attached to their parent atoms and treated as riding atoms. Contributions to scattering due to these highly disordered solvent molecules were removed using the *SQUEEZE* routine of *PLATON*; We had tried our best to get the ideal single crystals of $\mathbf{2^{Zr}}$ and $\mathbf{3^{Zr}}$, and even the crystals were diffracted by synchrotron radiation. However, the data sets were found to be collected poorly, especially at high angles. Among these data sets for the two BMOFs, we chose the best structure solution and refinement, but there was still one A-level alert problem in both $\mathbf{2^{Zr}}$ and $\mathbf{3^{Zr}}$. The A-level alert problem, in which the diffraction was non-existent much beyond 0.95Å, might be caused by the size of the crystal or the bed quality of the crystal.

The structure was then refined again using the data generated. Crystal data and details of the data collection are given in **Table S1**. CCDC 2321983−2321985 contained the supplementary crystallographic data for this paper. These data can be obtained free of charge from the Cambridge Crystallographic Data Centre via www.ccdc.cam.ac.uk/data request/cif

2 Synthesis

2.1 Synthesis of bodipy ligands

2.1.1 Synthesis of H_2THDFB

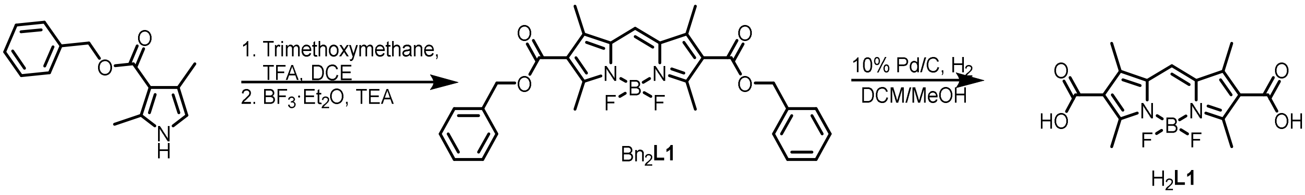

Scheme S1. Synthesis of H_2THDFB

Benzyl 2,4-dimethyl-1*H*-pyrrole-3-carboxylate (4.6 g, 20.0 mmol) and trimethoxymethane (1.06 g, 10 mmol) were dissolved in 1,2-dichloroethane (50 mL). Trifluoroacetic acid (0.1 mL) was added

under an Ar atmosphere, and the solution was stirred at room temperature overnight. When TLC monitoring (silica; CH_2Cl_2) showed complete consumption of the pyrrolic ester, the reaction mixture was concentrated under a vacuum. The acquired crude dipyrromethene was dissolved in toluene and triethylamine (17 mL). $BF_3{\cdot}OEt_2$ (15 mL) was added dropwise under an Ar atmosphere, and the solution was stirred at room temperature for 1 hr. Then EA was added, and the mixture was washed with brine, dried over Na_2SO_4, filtered, and evaporated. The crude product was purified by column chromatography over silica gel (CH_2Cl_2) and recrystallised from CH_2Cl_2 to afford Bn2**L1** as orange crystals (0.98 g, yield 15.5 %).

^{1}H NMR (400 MHz, $CDCl_3$): δ: 2.51 (s, 6H), 2.82 (s, 6H), 5.32 (s, 4H), 7.33-7.36 (m, 2H), 7.38-7.43 (m, 9H).

^{13}C NMR (400 MHz, $CDCl_3$): δ: 12.18, 15.17, 66.17, 120.64, 122.71, 128.26, 128.31, 128.67, 129.47, 133.05, 135.98, 146.17, 161.41, 158.18, 163.82.

^{11}B NMR (400 MHz, $CDCl_3$): 0.82 (t, $J_{B\text{-}F}$ = 62 Hz, 1B)

^{19}F NMR (400 MHz, $CDCl_3$): −143.23 (dd, $J_{F\text{-}B}$ = 24 Hz, $J_{F\text{-}B}$ = 48 Hz, 2F)

Bn2**L1** (0.52 g, 1 mmol) was dissolved in CH_2Cl_2/methanol, 2:1 (20 mL). After adding a small amount of 10% Pd-C, the mixture was stirred under an H_2 atmosphere for 3 hr. When TLC monitoring (silica; CH_2Cl_2-0.1% (v/v) AcOH) showed that the formation of the product was complete, DMF (50 mL) was added to the solution, and the Pd-C was filtered off. Evaporation of the filtrate gave H2**L1** as a red solid. Recrystallisation from CH_2Cl_2 afforded the pure product as red crystals (279 mg, yield 83%).

^{1}H NMR (400 MHz, $CDCl_3$): δ: 2.54 (s, 6H), 2.70 (s, 6H), 8.18 (s, 1H), 12.72 (s, 2H).

^{13}C NMR (400 MHz, $CDCl_3$): δ: 12.24, 15.04, 121.47, 126.75, 133.07, 146.93, 160.14, 165.37.

^{11}B NMR (400 MHz, $CDCl_3$): 0.72 (t, $J_{B\text{-}F}$ = 68 Hz, 1B)

^{19}F NMR (400 MHz, $CDCl_3$): −141.25 (dd, $J_{F\text{-}B}$ = 20 Hz, $J_{F\text{-}B}$ = 44 Hz, 2F)

2.1.2 Synthesis of H2TPDFB

The ligand **H2TPDFB** was synthesized according to the literature.[1]

2.1.3 Synthesis of H2TMDFB

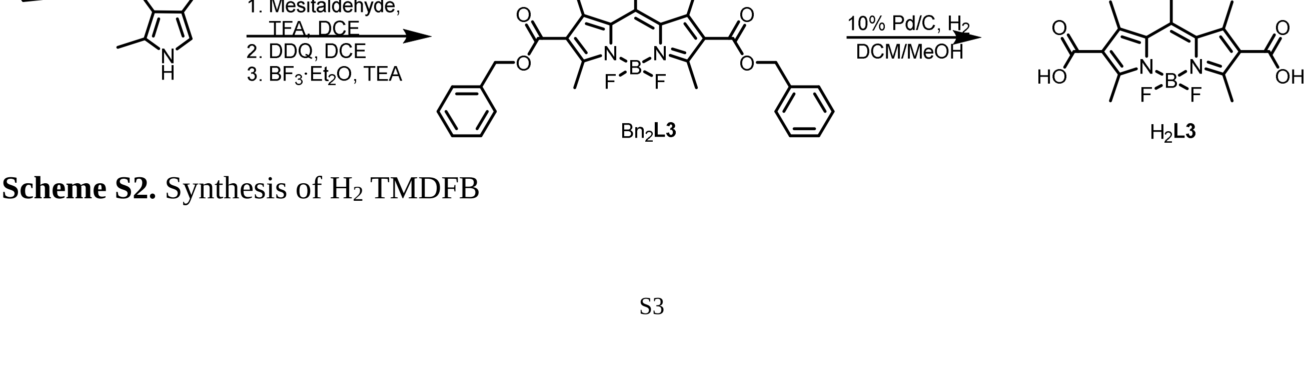

Scheme S2. Synthesis of H2 TMDFB

Benzyl 2,4-dimethyl-1*H*-pyrrole-3-carboxylate (4.6 g, 20.0 mmol) and mesitaldehyde (1.48 g, 10 mmol) were dissolved in 1,2-dichloroethane (50 mL). Trifluoroacetic acid (0.1 mL) was added under an Ar atmosphere, and the solution was stirred at room temperature overnight. When TLC monitoring (silica; CH_2Cl_2) showed complete consumption of the aldehyde, a solution of DDQ (2.27 g, 10 mmol) in 1,2-dichloroethane was added, and after 30 min of stirring, the reaction was washed with brine three times, dried over Na_2SO_4, filtered, and evaporated. The remaining red solid was purified on a filtration column (alumina). Acquired crude dipyrromethene was dissolved in toluene and triethylamine (17 mL). $BF_3{\cdot}OEt_2$ (15 mL) was added dropwise under an Ar atmosphere, and the solution was stirred at room temperature for 1 h. Then, AcOEt was added, and the mixture was washed with brine, dried over Na_2SO_4, filtered, and evaporated. The crude product was purified by column chromatography over silica gel (CH_2Cl_2) and recrystallized from CH_2Cl_2 to afford Bn_2**L3** as orange crystals (0.98 g, yield 15.5 %).

^{1}H NMR (400 MHz, $CDCl_3$): δ: 1.69 (s, 6H), 2.06 (s, 6H), 2.35 (s, 3H), 2.83 (s, 6H), 5.72 (s, 4H) 6.98 (s, 2H), 7.32-7.39 (m, 10H).

^{13}C NMR (400 MHz, $CDCl_3$): δ: 11.39, 14.13, 18.45, 20.21, 65.16, 120.82, 127.24, 127.32, 127.58, 128.49, 129.47, 129.62, 133.55, 134.84, 138.50, 145.55, 146.13, 158.46, 168.10.

^{11}B NMR (400 MHz, $CDCl_3$): 0.71 (t, $J_{\text{B-F}}$ = 64, 1B)

^{19}F NMR (400 MHz, $CDCl_3$): −142.97 (dd, $J_{\text{F-B}}$ = 20 Hz, $J_{\text{F-B}}$ = 48 Hz, 2F)

Elemental analysis: Calculated for $C_{38}H_{37}BF_2N_2O_4$: C, 71.93; H, 5.88; N, 4.41. Found: C, 71.04; H, 6.24; N, 4.12.

Bn_2**L3** (0.63 g, 1 mmol) was dissolved in CH_2Cl_2/methanol, 2:1 (20 mL). After adding a small amount of 10% Pd-C, the mixture was stirred under a H_2 atmosphere for 3 hr. When TLC monitoring (silica; CH_2Cl_2-0.1% (v/v) AcOH) showed that the formation of the product was complete, DMF (50 mL) was added to the solution, and the Pd-C was filtered off. Evaporation of the filtrate gave H_2**L3** as a red solid. Recrystallization from CH_2Cl_2 afforded the pure product as red crystals (377 mg, yield 83%).

^{1}H NMR (400 MHz, DMSO-*d*$_6$) δ: 1.64 (s, 6H), 2.02 (s, 6H), 2.34 (s, 3H), 2.73 (s, 6H), 7.12 (s, 2H), 12.82 (s, 1H).

^{13}C NMR (600 MHz, DMSO-*d*$_6$) δ: 12.28, 15.15, 19.41, 21.26, 123.20, 129.82, 130.37, 134.61, 139.59, 146.43, 158.87, 165.43.

^{11}B NMR (400 MHz, DMSO-*d*$_6$) δ: 0.14 (t, $J_{\text{B-F}}$ = 64, 1B)

^{19}F NMR (400 MHz, DMSO-*d*$_6$) δ: −140.97 (dd, $J_{\text{F-B}}$ = 20 Hz, $J_{\text{F-B}}$ = 48 Hz, 2F)

Elemental analysis: Calculated for $C_{24}H_{25}BF_2N_2O_4$: C, 63.45; H, 5.55; N, 6.41. Found: C, 62.11; H, 5.83; N, 5.93.

2.2 Synthesis of BMOFs

2.2.1 Synthesis of 1^{Zr}, 2^{Zr}, and 3^{Zr}

Synthesis of 1^{Zr}: A mixture of $ZrCl_4$ (113 mg, 0.485 mmol), H_2THDFB (200 mg, 0.485 mmol), DMF (60 mL), HAc (10 mL) was sealed in a 100 mL vial with a screw cap and heated at 120 °C for 24 h. The orange precipitates were collected, washed with DMF and acetone, and dried in air. Yield: 220 mg, 87%. The products can be best formulated as $[Zr_6O_4(OH)_4$ $(THDFB)_6]\cdot G$ (G represents the disordered guest molecules occupied in the frameworks) based on microanalysis, TGA, elemental analysis, IR, and single-crystal diffraction. Elemental analysis and IR for 1^{Zr}: Anal (%). Calculated for $C_{90}H_{78}O_{32}N_{12}B_6F_{12}Zr_6$: C, 40.34; H, 2.93; N, 6.27. Found: C, 41.97; H, 4.56; N, 7.83. IR (KBr, cm^{-1}): 3364 (w), 2928 (w), 1664 (m), 1612 (m), 1577 (s), 1505 (s), 1437 (s), 1414 (s), 1381 (s), 1352 (s), 1245 (s), 1177 (s), 1092 (w), 1063 (w), 1030 (m), 933 (w), 769 (m), 744 (w), 663 (s), 592 (s), 471 (s).

Synthesis of 2^{Zr}: The synthesis of 2^{Zr} was the same as that of 1^{Zr} except that the ligand was exchanged with H_2TPDFB in 83% yield. The products can be best formulated as $[Zr_6O_4(OH)_4$ $(TPDFB)_6]\cdot G$ based on microanalysis, TGA, elemental analysis, IR, and single-crystal diffraction. Elemental analysis and IR for 2^{Zr}: Anal (%). Calculated for $C_{126}H_{106}O_{32}N_{12}B_6F_{12}Zr_6$: C, 48.19; H, 3.40; N, 5.35. Found: C, 47.97; H, 3.15; N, 5.83. IR (KBr, cm^{-1}): 3399 (w), 1709 (w), 1665 (w), 1575 (w), 1540 (w), 1504 (m), 1344 (s), 1314 (m), 1192 (s), 1147 (s), 1073 (m), 1025 (m), 797 (m), 723 (m), 702 (m), 666 (s), 598 (s), 470 (s).

Synthesis of 3^{Zr}: The synthesis of 3^{Zr} was the same as 1^{Zr} except that the ligand was exchanged with H_2TMDFB in a 78% yield. The products can be best formulated as $[Zr_6O_4(OH)_4$ $(TMDFB)_6]\cdot G$ based on microanalysis, TGA, elemental analysis, IR, and single-crystal diffraction. Elemental analysis and IR for 3^{Zr}: Anal (%). Calculated for $C_{144}H_{154}O_{32}N_{12}B_6F_{12}Zr_6$: C, 50.79; H, 4.56; N, 4.94. Found: C, 48.85; H, 5.67; N, 3.86. IR (KBr, cm^{-1}): 3303 (w), 2916 (w), 2850 (w), 1661 (w), 1580 (w), 1531 (w), 1502 (m), 1430 (s), 1394 (s), 1378 (m), 1343 (s), 1310 (s), 1187 (s), 1144 (s), 1073 (m), 1024 (m), 799 (m), 777 (m), 702 (m), 663 (s), 598 (s), 477 (s).

2.2.2 Synthesis of the multistyryl modified BMOFs

1^{Zr} (100 mg, 0.037 mmol) and 1H-pyrrole-3-carbaldehyde (**3-Pyr**, 425 mg, 4.50 mmol) were added to a 100 mL round-bottomed flask containing 20 mL toluene, and to this solution was added piperidine (0.4 mL) and acetic acid (0.4 mL). The mixture was heated under reflux for at least 12 h. After cooling to room temperature, the black powder $1^{Zr\text{-}3Pyr}$ was collected by filtration, washed with DMF and acetone, and dried at 80 °C under vacuum to afford the black free-flowing powder with almost quantitative yield. Other multistyryl modified BMOFs $1^{Zr\text{-}2Pyr}$, $1^{Zr\text{-}3The}$, $1^{Zr\text{-}2The}$, $1^{Zr\text{-}3Fur}$, $1^{Zr\text{-}NMP}$, $1^{Zr\text{-}3Pyz}$, $1^{Zr\text{-}4Pyz}$, $1^{Zr\text{-}Im}$, and $1^{Zr\text{-}Ph}$ were synthesized the same as $1^{Zr\text{-}3Pyr}$ except that the 1H-pyrrole-3-carbaldehyde (**3-Pyr**) was exchanged with 2-pyrrolecarboxaldehyde (**2-Pyr**), 3-thenaldehyde (**3-The**), 2-thenaldehyde (**2-The**), 3-furaldehyde (**3-Fur**), N-methylpyrrole-2-carboxaldehyde (**NMP**), 3-formylpyrazole (**3-Pyz**), 4-formylpyrazole (**4-Pyz**), 2-formylimidazole (**Im**), and benzaldehyde (**Ph**), respectively.

IR for $1^{Zr\text{-}3Pyr}$: 3281 (w), 2944 (w), 2859 (w), 1581 (m),1410 (m),1363 (m), 1239 (m), 1101 (m), 1022

(m), 649 (s), 587 (s), 472 (s).

IR for **1**$^{Zr\text{-}2Pyr}$: 3403 (w), 2929 (w), 2849 (w), 1556 (m), 1506 (m), 1357 (m), 1236 (m), 1162 (m), 1031 (m), 742 (m), 646 (s), 587 (s), 460 (s).

IR for **1**$^{Zr\text{-}3The}$: 3341 (w), 2952 (w), 2865 (w), 1581 (m), 1500 (m), 1410 (m), 1239 (m),1164 (m), 1025 (m), 798 (m), 649 (s), 587 (m), 468 (s).

IR for **1**$^{Zr\text{-}2The}$: 3359 (w), 2916 (w), 2847 (w), 1574 (m), 1409 (m),1363 (m), 1236 (m), 1059 (m), 795 (m), 642 (s), 586 (m), 459 (s).

IR for **1**$^{Zr\text{-}3Fur}$: 3358 (w), 2931 (w), 2857 (w), 1574 (m),1509 (m), 1410 (m), 1236 (m), 1167 (s), 1019 (m), 795 (m), 649 (s), 587 (s), 469 (s).

IR for **1**$^{Zr\text{-}NMP}$: 3385 (w), 2935 (w), 2878 (w), 1574 (m), 1409 (m), 1236 (m), 1164 (m), 1059 (m), 1021 (m), 795 (m), 642 (s), 587 (m), 459 (s).

IR for **1**$^{Zr\text{-}3Pyz}$: 3433 (w), 2945 (s), 2862 (w), 1639 (s), 1606 (s), 1556 (s), 1469 (m), 1378 (m), 1337 (s), 1229 (m), 1200 (m), 1175 (m), 1134 (m), 1026 (w), 930 (w), 794 (w), 711 (m), 574 (m).

IR for **1**$^{Zr\text{-}4Pyz}$: 3251 (w), 2934 (w), 2842 (w), 1587 (m), 1410 (m), 1236 (m), 1158 (m), 823 (m), 776 (s), 649 (s), 590 (m), 466(s).

IR for **1**$^{Zr\text{-}Im}$: 3231 (w), 2935 (m), 2841 (w), 1574 (m), 1409 (m), 1363 (m), 1236 (m), 795 (m), 642 (s), 586 (m), 459 (s).

IR for **1**$^{Zr\text{-}Ph}$: 3393 (w), 2947 (w), 2867 (w), 1577 (m), 1406 (m), 1363 (m), 1232 (m), 1161 (m), 1028 (m), 795 (m), 642 (s), 590 (m), 459 (s).

2.2.3 Synthesis of binary BMOFs

A mixture of $ZrCl_4$ (113 mg, 0.485 mmol), metal chloride (65 mg, 0.485 mmol), H_2THDFB (200 mg, 0.485 mmol), DMF (60 mL), HAc (10 mL) was sealed in a 100 mL vial with a screw cap and heated at 120 °C for 24 h. The orange precipitates were collected, and then immersed in 25 mL anhydrous DMF solution of metal chloride (8 mmol/L) at 80 °C. After 12 h, the exchanged MOFs were thoroughly washed with DMF (10 mL × 3) and re-immersed in the related metal chloride as mentioned above solution for another 12 h. The corresponding binary BMOFs **1**^{ZrM} (**1**^{ZrSc}, **1**^{ZrTi}, **1**^{ZrV}, and **1**^{ZrSn}) were afforded after ten exchange cycles and collected, washed with DMF and acetone, and dried under vacuum at room temperature as the powder-form. These six binary BMOFs can be best formulated as $[(Zr_{1\text{-}x}M_x)_6O_4(OH)_4$ (THDFB)$_6$] based on EA, TGA, IR, and ICP-MS, where *x* represents the average number of the corresponding metal irons per SBU.

1^{ZrSc} can be formulated as $[(Zr_{0.17}Sc_{0.83})_6O_4(OH)_4$ (THDFB)$_6$]. Elemental analysis, ICP-MS, and IR measurements for **1**^{ZrSc}: Anal (%). Calculated for $C_{90}H_{78}O_{32}N_{12}B_6F_{12}Zr_{1.02}Sc_{4.98}$: C, 44.13; H, 3.31; N, 6.86. Found: C, 41.85; H, 3.17; N, 6.66. ICP-MS measurement indicated the molar ratio of B:Zr:Sc is 1:0.17:0.83. IR (KBr, cm^{-1}): 3336 (s), 2975 (w), 2930 (w), 2882 (w), 1650 (s), 1568 (s), 1495 (m), 1443 (m), 1406 (s), 1382 (s), 1346 (s), 1240 (s), 1166 (s), 1089 (s), 1048 (s), 876 (w), 793 (m), 739 (w), 658 (m), 589 (s), 463 (s).

1^{ZrTi} can be formulated as $[(Zr_{0.13}Ti_{0.87})_6O_4(OH)_4$ $(THDFB)_6]$. Elemental analysis, ICP-MS, and IR measurements for 1^{ZrTi}: Anal (%). $C_{90}H_{78}O_{32}N_{12}B_6F_{12}Zr_{0.78}Ti_{5.22}$: C, 44.06; H, 3.20; N, 6.85. Found: C, 42.85; H, 3.02; N, 5.97. ICP-MS measurement indicated the molar ratio of B:Zr:Ti is 1:0.13:0.87. IR (KBr, cm^{-1}): 3291 (w), 2929 (w), 1658 (m), 1572 (m), 1498 (m), 1442 (m), 1409 (s), 1385 (s), 1356 (s), 1239 (s), 1166 (s), 1096 (w), 1056 (w), 1028 (s), 938 (w), 881 (w), 792 (m), 739 (m), 657 (s), 584 (s), 475 (s).

1^{ZrV} can be best formulated as $[(Zr_{0.5}V_{0.5})_6O_4(OH)_4$ $(THDFB)_6]$. Elemental analysis, ICP-MS, and IR measurements for 1^{ZrV}: Anal (%). Calculated for $C_{90}H_{78}O_{32}N_{12}B_6F_{12}Zr_3V_3$: C, 42.24; H, 3.07; N, 6.57. Found: C, 40.67; H, 3.53; N, 6.22. ICP-MS measurement indicated the molar ratio of B:Zr:V is 1:0.50:0.50. IR (KBr, cm^{-1}): 3392 (w), 2935 (w), 1664 (m), 1605 (m), 1580 (m), 1506 (m), 1436 (s), 1413 (s), 1362 (s), 1237 (s), 1170 (s), 1063 (w), 1023 (m), 934 (w), 802 (m), 746 (w), 658 (s), 588 (s), 466 (s).

1^{ZrSn} can be best formulated as $[(Zr_{0.54}Sn_{0.46})_6O_4(OH)_4$ $(THDFB)_6]$. Elemental analysis, ICP-MS, and IR measurements for 1^{ZrSn}: Anal (%). Calculated for $C_{90}H_{78}O_{32}N_{12}B_6F_{12}Zr_{3.24}Sn_{2.76}$: C, 39.23; H, 2.85; N, 6.10. Found: C, 39.54; H, 3.71; N, 5.97. ICP-MS measurement indicated the molar ratio of B:Zr:Sn is 1:0.54:0.46. IR (KBr, cm^{-1}): 3327 (w), 2968 (w), 2925 (w), 2875 (w), 1661 (m), 1576 (m), 1502 (m), 1436 (s), 1409 (s), 1379 (s), 1357 (s), 1236 (s), 1168 (s), 1132 (w), 1086 (m), 1047 (s), 1020 (s), 940 (w), 875 (m), 795 (m), 745 (w), 661 (s), 591 (s), 471 (s).

3 Experimental procedure for heterogeneous photocatalysis.

Prior to catalysis, all the BMOFs were exchanged with chloroform for two days and then activated under vacuum at room temperature for 4 h to remove the guest solvents.

3.1 The photocatalytic α-C(sp^3)−H carbamoylation of saturated aza-heterocycles to access amides

To a flame-dried Schlenk pressure tube was added activated photocatalyst (1.0 mol%), formic acid (0.5 mmol), tertiary arylamine (0.5 mmol), isocyanide (0.6 mmol), and $CHCl_3$ (1 mL) under air condition. The mixture was stirred under irradiation by a 5 W white LED lamp at room temperature for 2 h. After that, the mixture was centrifuged at 9000 rpm for 5 min; the supernatant was concentrated under a vacuum and then purified by column chromatography on silica gel (EtOAc/ petroleum ether, 1/4, v/v) to afford the desired products. The conversion was calculated according to the ^1H NMR of the reaction solutions. The photocatalyst loading was calculated based on the bodipy unit in each counterpart.

Thermodynamic calculation

The determined oxidation potential of E_{ox} (**6a**) = 0.57 V, E_{red} (1^{Zr}) = –0.79 V, and E^{0-0} = 2.21 eV (read from the cross-point of the absorption and luminescence spectra at 557 nm, the negative free energy change (ΔG = –0.85 eV) calculated by using the Rehm-Weller Equation (1).

$\Delta G = E_{ox} - E_{red} - E^{0-0}$ (1)

3.2 The photocatalytic dealkylation/acylation of tertiary amines to access amides

To a flame-dried Schlenk pressure tube was added activated photocatalyst (1.0 mol%), aniline (0.5 mmol, 1.0 equiv.), benzoyl chloride (0.6 mmol, 1.2 equiv.), PFNB (0.25 mmol, 0.5 equiv.), $KHCO_3$ (50.0 mg, 0.5 mmol, 1.0 equiv.), and THF (1 mL) under air condition. The mixture was stirred under irradiation by a 5 W white LED lamp at room temperature. After completion of the reaction, as checked by TLC, the mixture was centrifuged at 9000 rpm for 5 min; the supernatant was concentrated under a vacuum and then purified by column chromatography on silica gel (EtOAc/petroleum ether) to afford the desired products. The conversion was calculated according to the ^{1}H NMR of the reaction solutions.

3.3 The recycle experiments (using the photocatalytic carbamoylation of saturated aza-heterocycles by $\mathbf{1^{Zr}}$ as an example)

To a suspension of $\mathbf{1^{Zr}}$ (1.0 mol%) in $CHCl_3$ (1.0 mL), formic acid (0.5 mmol), tertiary arylamine (0.5 mmol), and *iso*cyanide (0.6 mmol) were added. The reaction was conducted under irradiation by a 5 W white LED lamp at room temperature under air conditions for 2 h. After that, the mixture was centrifuged at 9000 rpm for 5 min; the precipitate was washed with $CHCl_3$ 3 times, sonicated for 10 min, and dried under pressure. Then, used for the next run, the supernatant was concentrated under vacuum. The concentrate was analyzed by ^{1}H NMR to give the conversion.

3.4 Catalyst leaching measurement by UV/vis spectra and ICP-MS (using the photocatalytic carbamoylation of saturated aza-heterocycles by $\mathbf{1^{Zr}}$ as an example)

To a suspension of $\mathbf{1^{Zr}}$ (1.0 mol%) in $CHCl_3$ (1.0 mL), formic acid (0.5 mmol), tertiary arylamine (0.5 mmol), and *iso*cyanide (0.6 mmol) were added. The reaction was conducted under irradiation by a 5 W white LED lamp at room temperature under air conditions for 2 h. After that, the mixture was centrifuged at 9000 rpm for 5 min; the supernatant was analyzed by UV/vis spectra. After that, the supernatant was concentrated under vacuum and used for ICP-MS analysis.

4 Mott−Schottky plot measurements

The BMOFs (2 mg) were dispersed into 10 μL 5 wt% Nafion, and 2 mL ethanol mixed solution and the working electrode was prepared by dropping the suspension (20 μL) onto the surface of the glassy carbon electrode. Mott−Schottky plots of the sample were measured on an electrochemical workstation (Autolab) in a standard three-electrode system with the photocatalyst-coated glassy carbon (Φ = 3 cm) as working electrode, Pt plate as counter electrode, and Ag/AgCl as reference electrode at frequencies of 500, 1000, and 1500 Hz, respectively. A 0.1 M Na_2SO_4 solution after deoxidation was used as electrolyte.

5 X-ray absorption fine structure (XAFS) spectroscopy

The X-ray absorption spectroscopy (XAS) data were recorded on beamline 14W1 at the Shanghai Synchrotron Radiation Facility (SSRF). The electron beam energy was 3.5 GeV with a stored current of approximately 200 mA in top-up operation. A fixed-exit double crystal Si <111> monochromator was used for the incident energy selection. XAS data of the samples were acquired in transmission mode using two ionization chambers filled with nitrogen. Standard procedures were followed to analyze the XAS data using the Demeter software package (J. Synchrotron. Radiat. 2005, 12, 537-541). The backscattering amplitude and phase shift were calculated with the program FEFF9 (Phys. Chem. Chem. Phys. 2010, 12 (21), 5503-5513). The XAS data were calibrated, averaged, pre-edge background subtracted, and post-edge normalized using the Athena program. Theoretical fittings for the EXAFS data were executed using the Artemis program. The Fourier transformation of the k^2-weighted EXAFS oscillations from *k*-space to *r*-space was performed over a range of 3.0−10.0 Å^{-1}. A window of 1.0−3.8 Å in *r*-space was applied to fit EXAFS data. The value of amplitude reduction factor S_0^2 was fixed at 0.75 during the fitting for the samples. Other structural parameters, such as coordination numbers (*N*), bond distance (*R*), Debye-Waller factor (σ^2), and inner potential shift (ΔE_0), were obtained from the fitting.

5.1 XAFS analysis of $\mathbf{1}^{\mathbf{ZrTi}}$

We performed X-ray absorption spectroscopy (XAS) at the Ti *K*-edge to investigate the Ti coordination environment. X-ray absorption near edge structure (XANES) data (Figure S34a) shows $\mathbf{1}^{\mathbf{ZrTi}}$ has the same absorption edge position (~4984eV) as TiO_2, revealing the same oxidation state of Ti. It is worth noting that the pre-edge peak (~4971eV) of $\mathbf{1}^{\mathbf{ZrTi}}$ has a different shape from that of TiO_2, revealing that the Ti^{4+} in $\mathbf{1}^{\mathbf{ZrTi}}$ has a different coordination environment from TiO_2. The extended X-ray absorption fine structure (EXAFS) data (Figure S34b) shows that TiO_2 exhibits Ti-O and Ti-Ti backscatter peaks at ~1.5 Å and ~2.5 Å, respectively. In contrast, the Ti-O peak of $\mathbf{1}^{\mathbf{ZrTi}}$ is located at ~1.6 Å, revealing that the $\mathbf{1}^{\mathbf{ZrTi}}$ has a longer average Ti-O bond length than TiO_2. Due to the longer bond length of Zr-O in $\mathbf{1}^{\mathbf{Zr}}$ than Ti-O in TiO_2, EXAFS data suggests that Ti in $\mathbf{1}^{\mathbf{ZrTi}}$ occupies the Zr position in $\mathbf{1}^{\mathbf{Zr}}$. The EXAFS fitting result (Figure S34c) shows that the $\mathbf{1}^{\mathbf{ZrTi}}$ can be well fitted by using both Ti-O and Ti-Ti (or Ti-Zr) shells with average lengths of ~ 2.07 Å and ~ 3.48 Å (**Table S2**), respectively. It should be noted that the average Ti-O and Ti-Ti (or Ti-Zr) distances are close to the Zr-O and Zr-Zr distances in $\mathbf{1}^{\mathbf{Zr}}$, respectively. It confirms that $\mathbf{1}^{\mathbf{ZrTi}}$ is derived by partially replacing Zr in $\mathbf{1}^{\mathbf{Zr}}$ with Ti.

5.2 XAFS analysis of $\mathbf{1}^{\mathbf{ZrSc}}$ and $\mathbf{1}^{\mathbf{ZrSn}}$

$\mathbf{1}^{\mathbf{ZrSc}}$ and $\mathbf{1}^{\mathbf{ZrSn}}$ (**Tables S3-S4**) model containing two substituted metal centres at opposite ends of the UiO typical cluster (derived from DFT substitution and geometric optimisation) was found to best fit the EXAFS data (Figures S35-S36). Models were also fitted from 1/6 up to 4/6 substituted Sc and Sn in the cluster. For $\mathbf{1}^{\mathbf{ZrSn}}$ the EXAFS data was a good fit to a model, with slightly shifted Sn positions from the corresponding substituted Zr positions in the cluster. There are two Sn-Zr distances of 3.61 and 3.77 Å with coordination number 2.1 (2) for each. The four carboxylate groups that are coordinated to the Sn atoms have a Sn-C distance of 3.16 Å with more varying Sn-O distances reflecting the asymmetry of the Sn atomic positions. For $\mathbf{1}^{\mathbf{ZrSc}}$ the model fitting yielded a symmetric cluster with all Sc-Zr distances equal at 3.37 Å and coordination number 3.4 (4). The four carboxylate groups coordinated to the Sc atoms have Sc-C distances of 3.21 Å and seven oxygen atoms of which are in the range 1.94 to 2.10 Å and two more Sc-O at a distance of 2.60 Å, consistent with the location of the Sc atoms at

the extreme ends of the M-O cluster. The fitting uncertainties are higher than $\mathbf{1}^{\mathbf{ZrSn}}$ and so this does not preclude the Sc being in a shifted position than the substituted Zr would have been located in as is the case in $\mathbf{1}^{\mathbf{ZrSn}}$.

6 Atomic pair distribution function (PDF) analysis

The atomic pair distribution function (PDF) data was measured at the BL13SSW line station of Shanghai Light Source. During the experiment, a lead plate with a small hole in the center was positioned in front of the sample container to minimize stray light interference. The energy employed was 50 keV (λ=0.248 Å), and an energy calibration at this value was achieved using tungsten absorption edges. To protect the detector, a beam aperture with a size of ~1 mm × 1 mm was also employed to block the direct beam. The binary BMOFs were affixed to the sample rack using two layers of Kapton tape, and each measurement was controlled within 90s. Additionally, we further measured the data of an empty container with two layers of Kapton tape as background for subsequent subtraction. Fit 2D software was applied to calibrate the scattering patterns with CeO_2 standard sample and then integrate the 2D patterns into 1D profiles to establish a relationship between intensity and 2θ. PDFgetX2 software was employed to subtract the background from the 1D patterns, followed by performing Fourier transform to compute G(*r*) patterns. The resulted PDF profiles were refined with the relevant structure models calculated from the CIF of $\mathbf{1}^{\mathbf{Zr}}$ by using PDFgui software. For the calculation from the CIF of $\mathbf{1}^{\mathbf{Zr}}$, import the CIF file of $\mathbf{1}^{\mathbf{Zr}}$ into the Material Studio program and then reduce the spatial group from Fm-3m to P-1. After removing the disorder in the structure, a new CIF file was generated and then employed for the calculation by PDFgui software. Unfortunately, if the disorder in the CIF file is not removed, the high degree of disorder in the $\mathbf{1}^{\mathbf{Zr}}$ structure would lead to a crash of the PDFgui software, rendering it incapable of performing calculations.

Table S1. Crystal data and structure refinement for **1^{Zr}**, **2^{Zr}**, and **3^{Zr}**

Identification code	**1^{Zr}**	**2^{Zr}**	**3^{Zr}**
Empirical formula	$C_{90}H_{78}B_6F_{12}N_{12}O_{32}Zr_6$	$C_{252}H_{204}B_{12}F_{24}N_{24}O_{64}Zr_{12}$	$C_{288}H_{276}B_{12}F_{24}N_{24}O_{64}Zr_{12}$
Formula weight	2679.66	6272.74	6777.67
Temperature (K)	170.00	170.00	170.00
Radiation	CuKα (λ = 1.54184)	CuKα (λ = 1.54184)	CuKα (λ = 0.71073)
Crystal system	Cubic	Cubic	Cubic
Space group	*Fm-3m*	*Fm-3m*	*Fm-3m*
Unit cell dimensions	a = b = c = 26.79(3) Å<br>$\alpha = \beta = \gamma$ =90	a = b = c = 26.336 Å<br>$\alpha = \beta = \gamma$ =90	a = b = c = 26.242(3) Å<br>$\alpha = \beta = \gamma$ = 90
Volume (Å^3), Z	19220.3(7), 24	18265.4, 2	18071(6), 2
ρ_{calc}g/cm^3	0.926	1.141	1.246
μ/mm^{-1}	3.079	3.312	0.409
F(000)	5344	6304.0	6880.0
θ range for data collection (º)	9.338 to 133.476	5.812 to 117.834	5.148 to 49.946
Index ranges	$-20 \le h \le 29$<br>$-3 \le k \le 31$<br>$-20 \le l \le 25$	$-17 \le h \le 29$<br>$-23 \le k \le 18$<br>$-22 \le l \le 29$	$3 \le h \le 31$<br>$0 \le k \le 22$<br>$0 \le l \le 21$
Reflections collected	3995	7318	1430
Independent reflections	911<br>[R_{int} = 0.0424, R_{sigma} = 0.0308]	720<br>[R_{int} = 0.0732, R_{sigma} = 0.0375]	854<br>[R_{int} = 0.0089, R_{sigma} = 0.0145]
Completeness to theta	99%	100%	99%
Data / restraints / parameters	911/204/89	721/495/217	854/444/246
Goodness-of-fit on F^2	1.072	1.126	1.104
Final R indices [I>2sigma(I)]	R_1 = 0.0983, wR_2 = 0.2518	R_1 = 0.0753, wR_2 = 0.1810	R_1 = 0.0920, wR_2 = 0.2218
R indices (all data)	R_1 = 0.1035, wR_2 = 0.2600	R_1 = 0.0849, wR_2 = 0.2009	R_1 = 0.0963, wR_2 = 0.2282
Largest diff. peak and hole (e.Å^{-3})	1.87/−1.98	1.51/−1.18	0.92/−1.49

7 Characterization

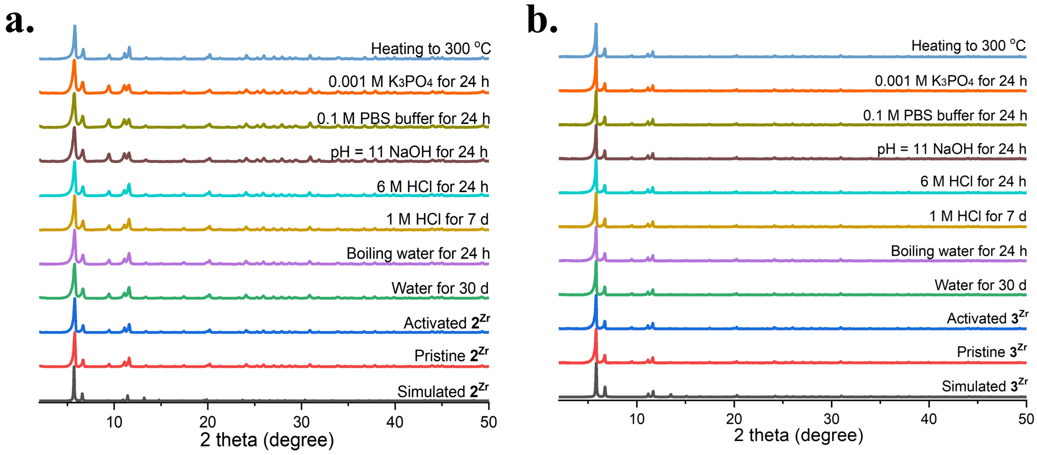

Figure S1. PXRD patterns of **a.** $\mathbf{2^{Zr}}$ and **b.** $\mathbf{3^{Zr}}$ after various treatments

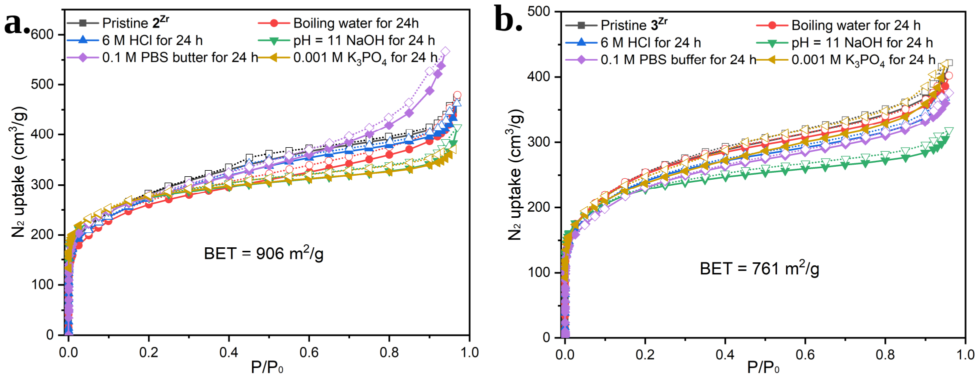

Figure S2. N_2 adsorption isotherms of **a.** $\mathbf{2^{Zr}}$ and **b.** $\mathbf{3^{Zr}}$ after various treatments.

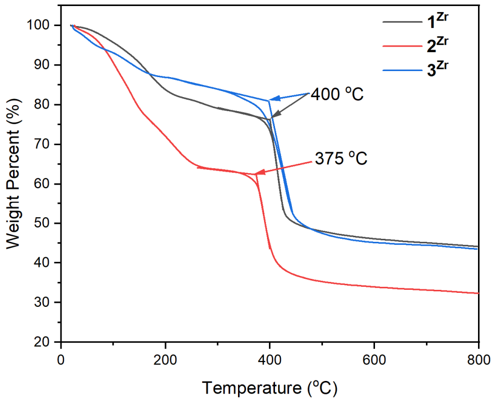

Figure S3. TGA curves.

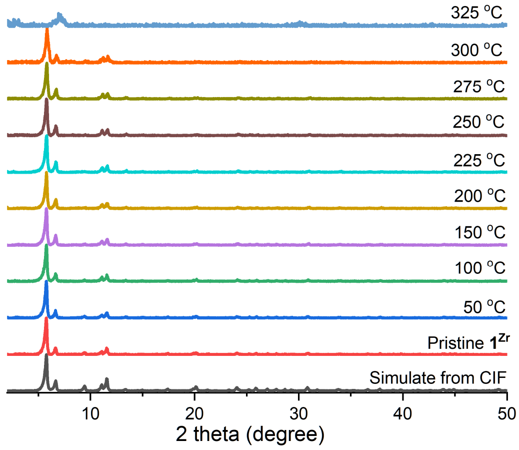

Figure S4. VT-PXRD patterns of **1^{Zr}**.

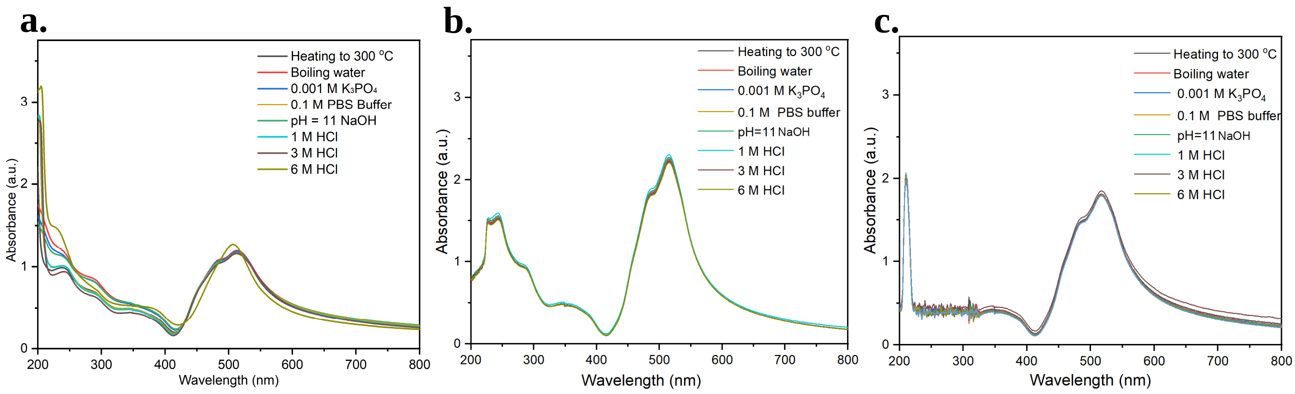

Figure S5. UV−Vis spectra of **a. 1^{Zr}**, **b. 2^{Zr}**, and **c. 3^{Zr}** after various treatments.

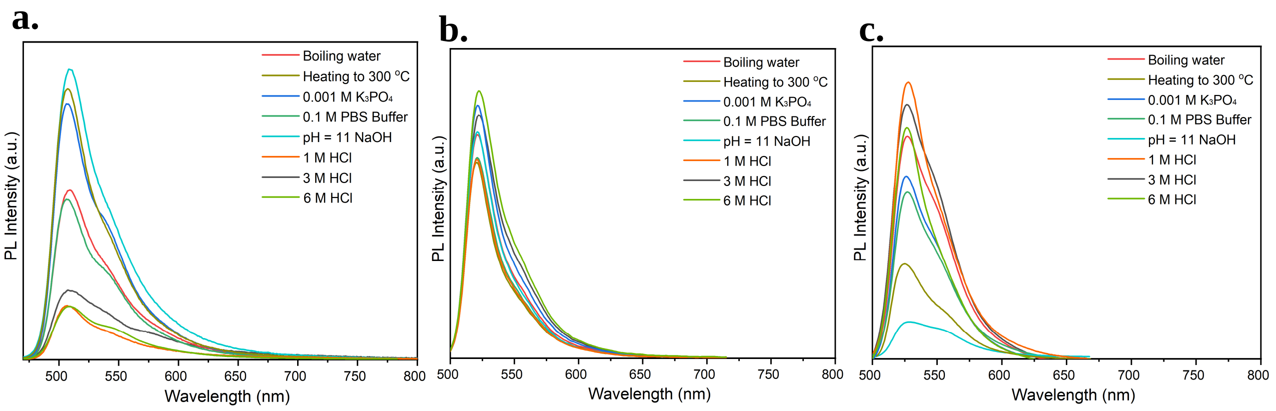

Figure S6. PL spectra of **a. 1^{Zr}**, **b. 2^{Zr}**, and **c. 3^{Zr}** after various treatments.

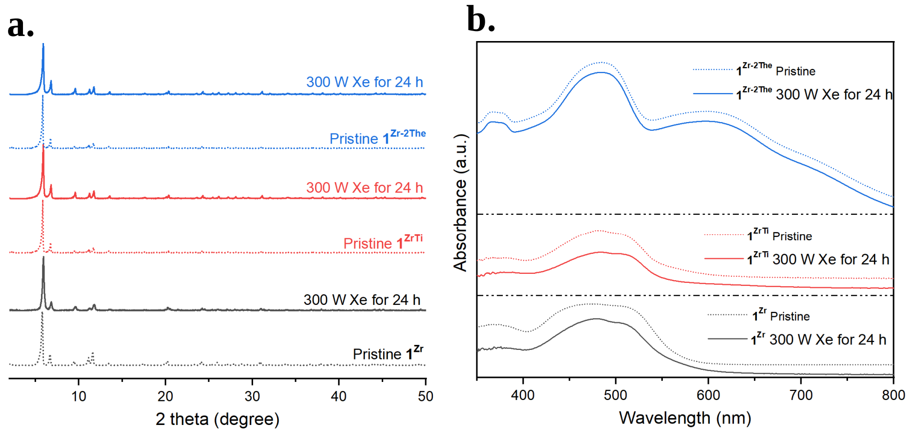

Figure S7. a. PXRD pattern and **b.** Solid-state UV−Vis spectra of $\mathbf{1}^{\mathbf{Zr}}$, $\mathbf{1}^{\mathbf{ZrTi}}$, and $\mathbf{1}^{\mathbf{Zr\text{-}The}}$ after the continuous irradiation with 300W Xe lamp for 24 h.

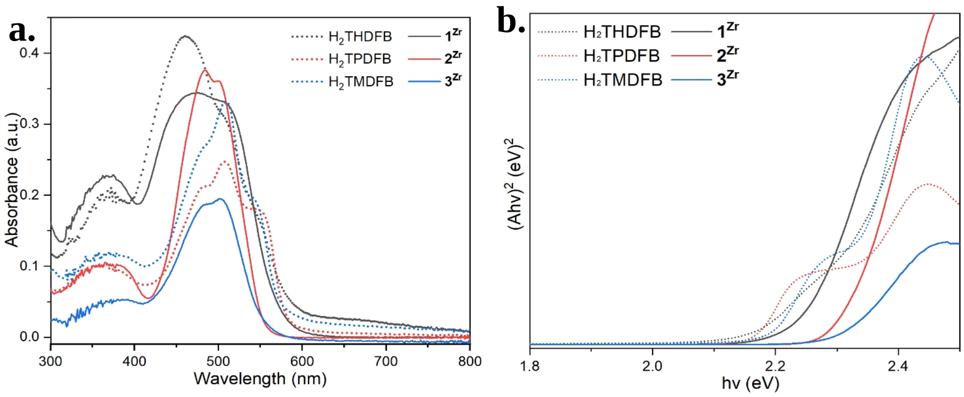

Figure S8. a. Solid-state UV−Vis spectra and **b.** Tauc plots of $\mathbf{1}^{\mathbf{Zr}}$, $\mathbf{2}^{\mathbf{Zr}}$, $\mathbf{3}^{\mathbf{Zr}}$, and their corresponding ligands.

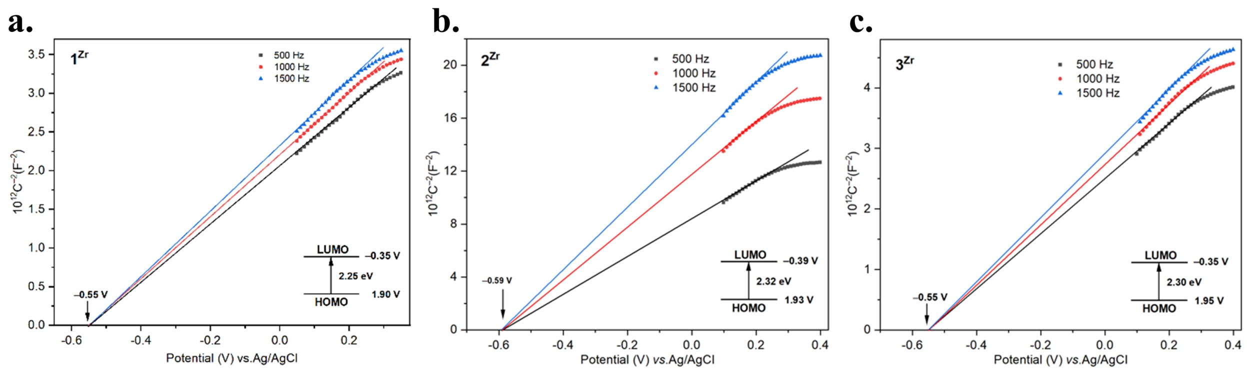

Figure S9. Mott–Schottky plots for **a. 1^{Zr}**, **b. 2^{Zr}**, and **c. 3^{Zr}**.

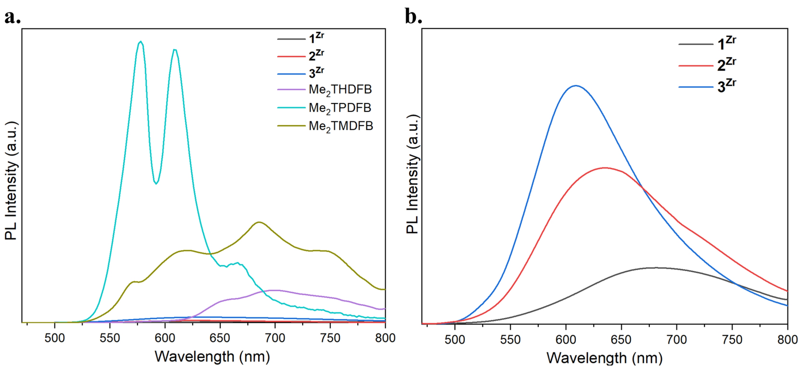

Figure S10. Solid-state PL spectra for **1^{Zr}**, **2^{Zr}**, **3^{Zr}**, and their ester monomers; **a**. full and **b**. zoomed in scale spectrum.

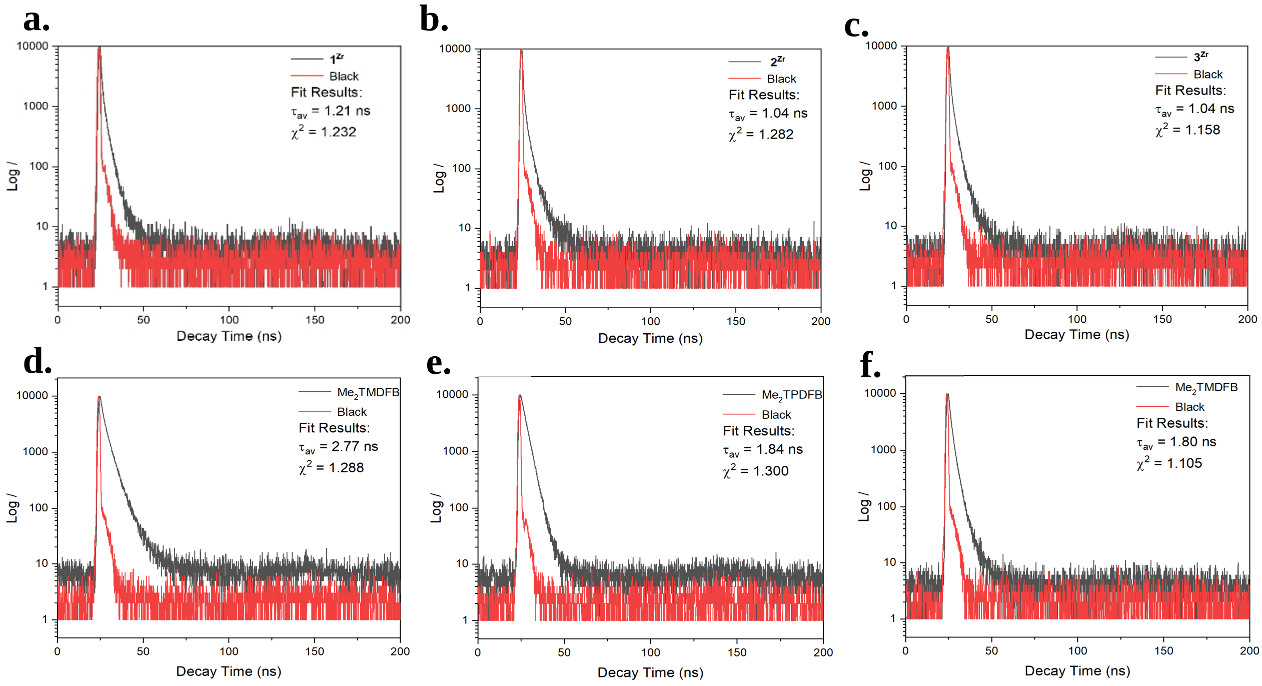

Figure S11. Fluorescence lifetimes of **a. 1^{Zr}**, **b. 2^{Zr}**, **c. 3^{Zr}**, **d.** Me_2THDFB, **e,** Me_2TPDFB, and **f.** Me_2TMDFB.

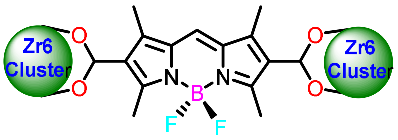

Figure S12. The structure of the unfunctionalized bodipy linker in **1**$^{\mathbf{Zr}}$.

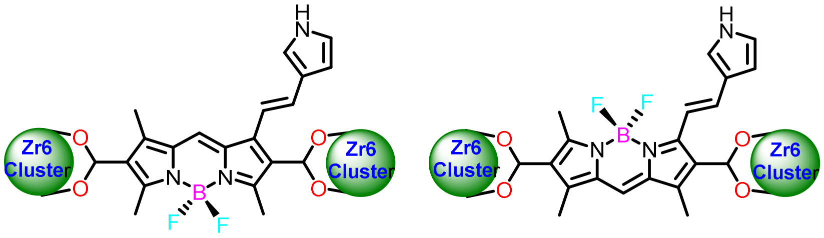

Figure S13. The possible structures of bodipy linker functionalized with one 3-Pyr moiety in **1**$^{\mathbf{Zr\text{-}3Pyr}}$.

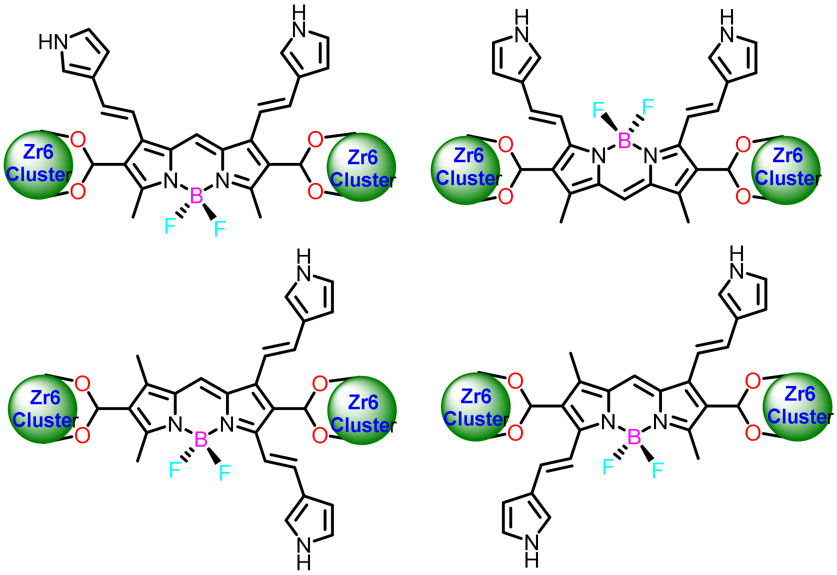

Figure S14. The possible structures of bodipy linker functionalized with two 3-Pyr moieties.

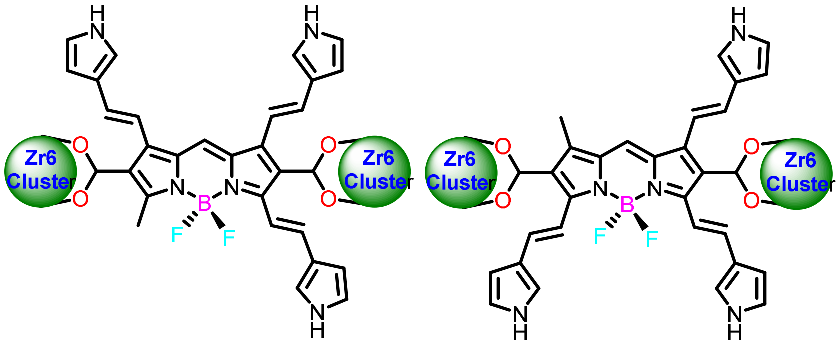

Figure S15. The possible structures of bodipy linker functionalized with three 3-Pyr moieties.

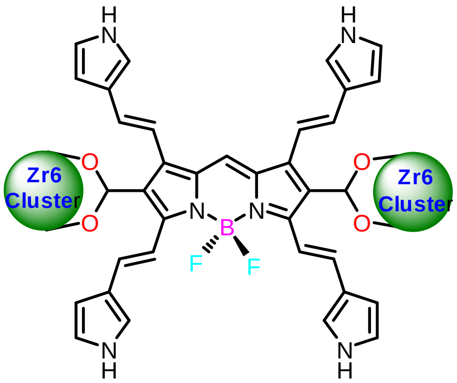

Figure S16. The possible structures of bodipy linker functionalized with four 3-Pyr moieties.

a.

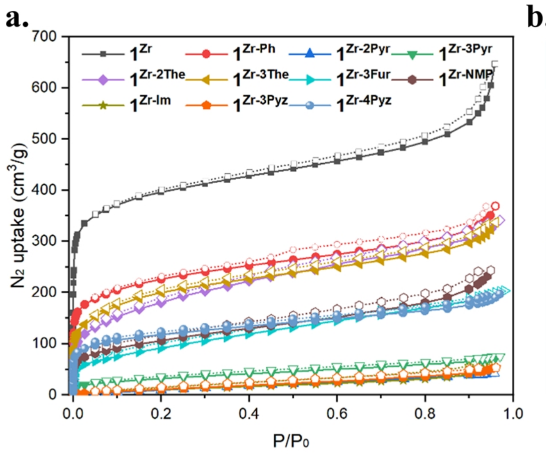

b.

BMOF	BET (m^2/g)	BMOF	BET (m^2/g)
1^{Zr}	1466	$1^{Zr-3Fur}$	273
1^{Zr-Ph}	809	$1^{Zr-3Pyr}$	97
$1^{Zr-3The}$	634	$1^{Zr-2Pyr}$	62
$1^{Zr-2The}$	639	1^{Zr-Im}	47
$1^{Zr-4Pyz}$	405	$1^{Zr-3Pyz}$	57
1^{Zr-NMP}	373		

Figure S17. a. The N_2 adsorption isotherms and **b**. BET areas (calculated using BETSI[2]) of $\mathbf{1}^{\mathbf{Zr-Ar}}$.

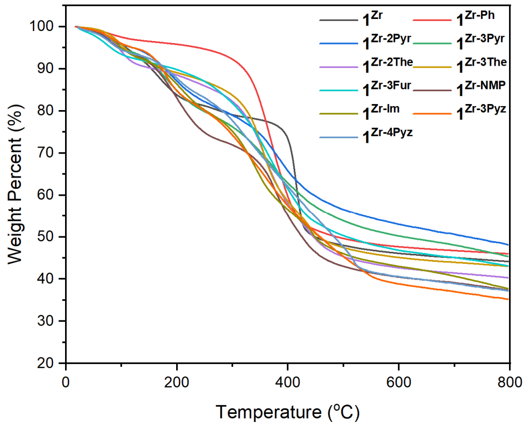

Figure S18. TGA curves of $\mathbf{1}^{\mathbf{Zr-Ar}}$.

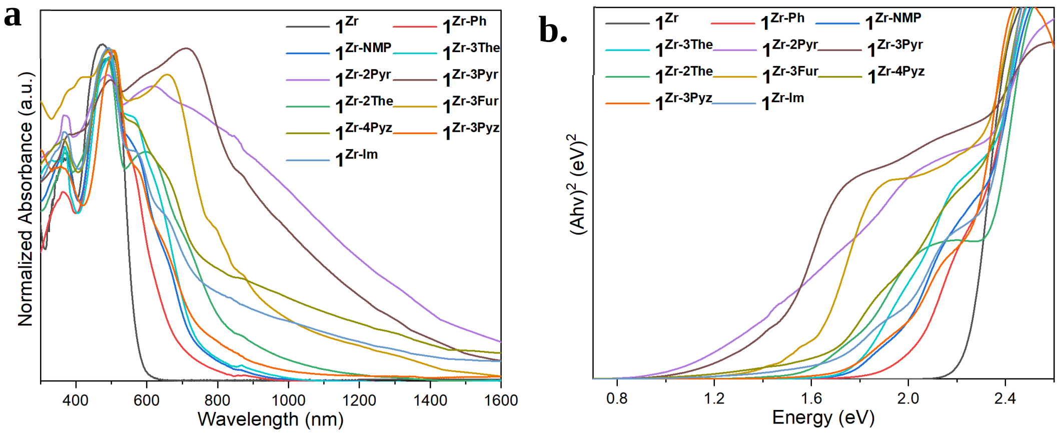

BMOFs	1^{Zr}	$1^{Zr\text{-}Ph}$	$1^{Zr\text{-}NMP}$	$1^{Zr\text{-}3Pyz}$	$1^{Zr\text{-}2The}$	$1^{Zr\text{-}3The}$	$1^{Zr\text{-}Im}$	$1^{Zr\text{-}4Pyz}$	$1^{Zr\text{-}3Fur}$	$1^{Zr\text{-}3Pyr}$	$1^{Zr\text{-}2Pyr}$
B. G. (eV)	2.21	1.97	1.86	1.77	1.70	1.65	1.65	1.62	1.60	1.41	1.18

Figure S19 a. Solid-state UV–Vis spectra and **b.** Tauc plots of $\mathbf{1^{Zr\text{-}Ar}}$.

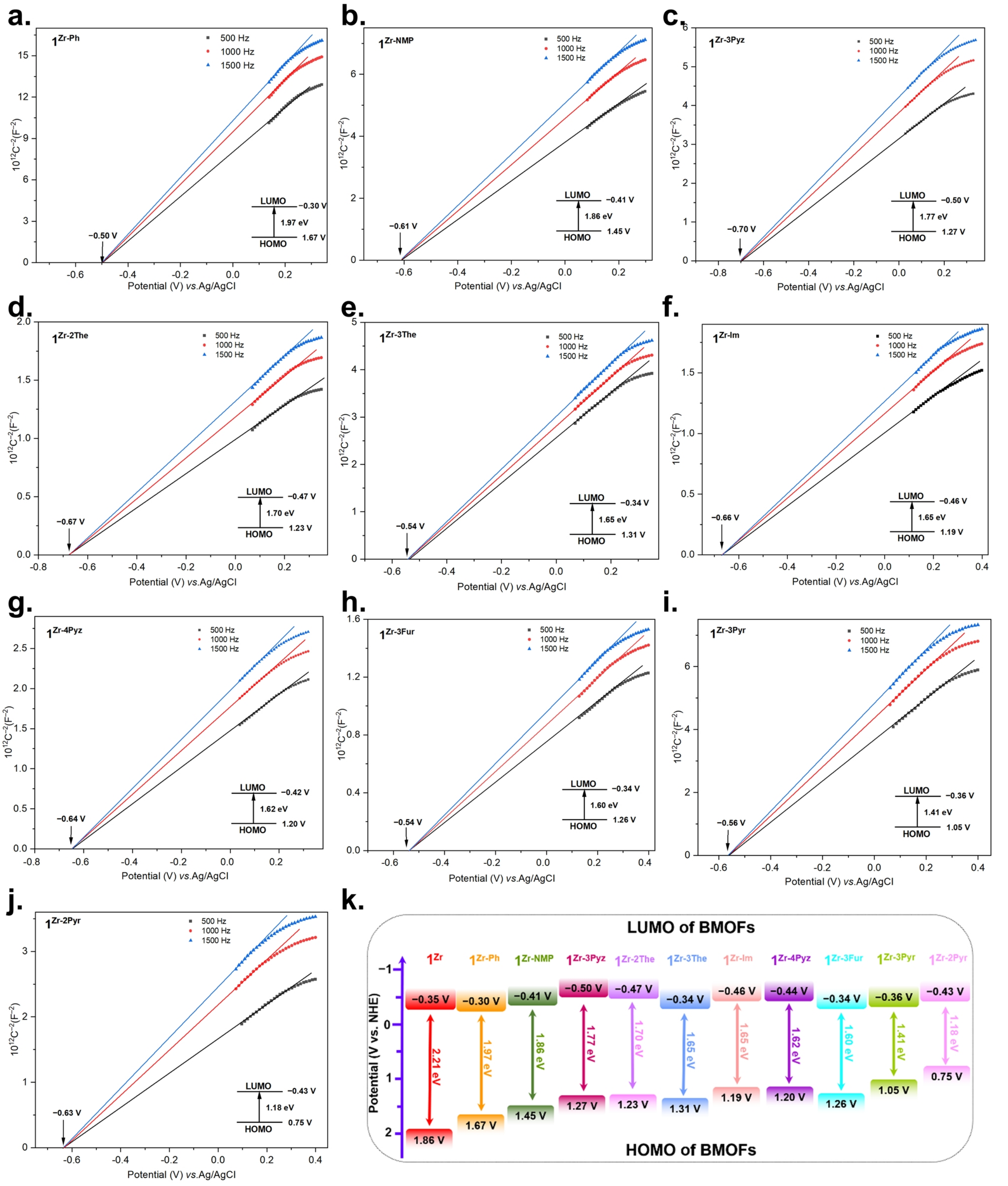

Figure S20. Mott–Schottky plots of **a**. $\mathbf{1^{Zr\text{-}Ph}}$, **b**. $\mathbf{1^{Zr\text{-}NMP}}$, **c**. $\mathbf{1^{Zr\text{-}3Pyz}}$, **d**. $\mathbf{1^{Zr\text{-}2The}}$, **e**. $\mathbf{1^{Zr\text{-}3The}}$, **f**. $\mathbf{1^{Zr\text{-}Im}}$, **g** $\mathbf{1^{Zr\text{-}4Pyz}}$, **h**. $\mathbf{1^{Zr\text{-}3Fur}}$, **i.** $\mathbf{1^{Zr\text{-}3Pyr}}$ **j**. $\mathbf{1^{Zr\text{-}2Pyr}}$. **k**. HOMO-LUMO gap of $\mathbf{1^{Zr\text{-}Ar}}$.

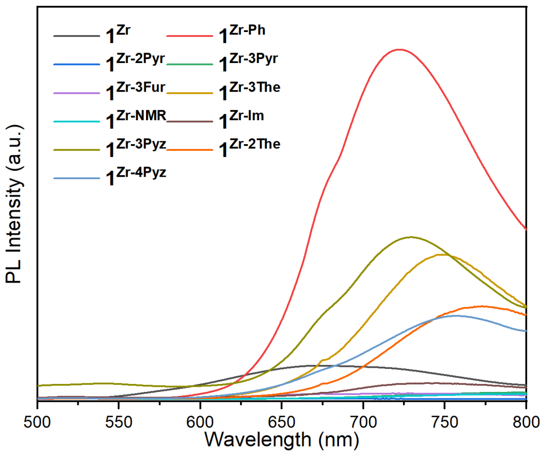

Figure S21. Solid-state PL spectra of $\mathbf{1}^{\mathbf{Zr\text{-}Ar}}$

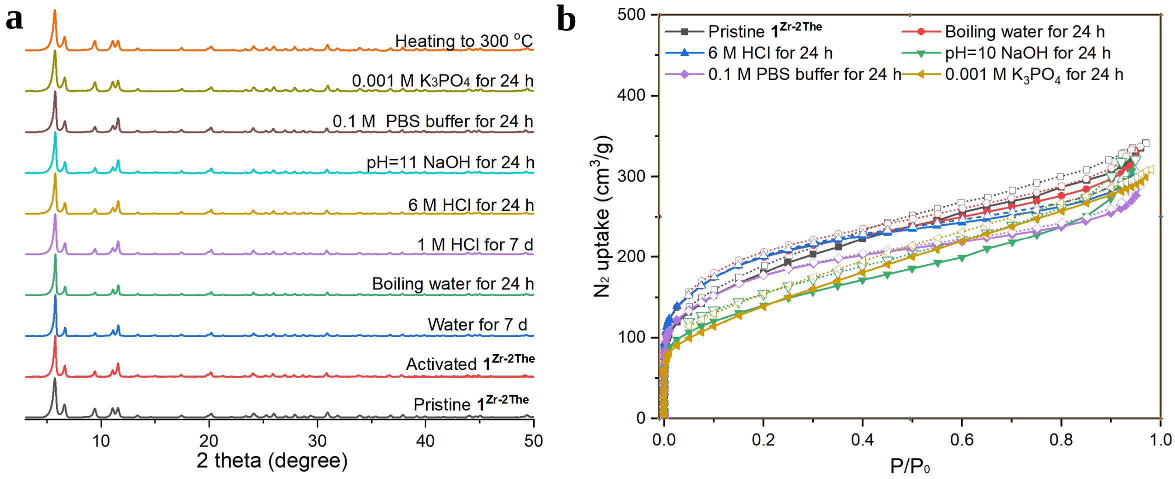

Figure S22 a. PXRD and **b.** BET of $\mathbf{1}^{\mathbf{Zr\text{-}2The}}$ after various treatments.

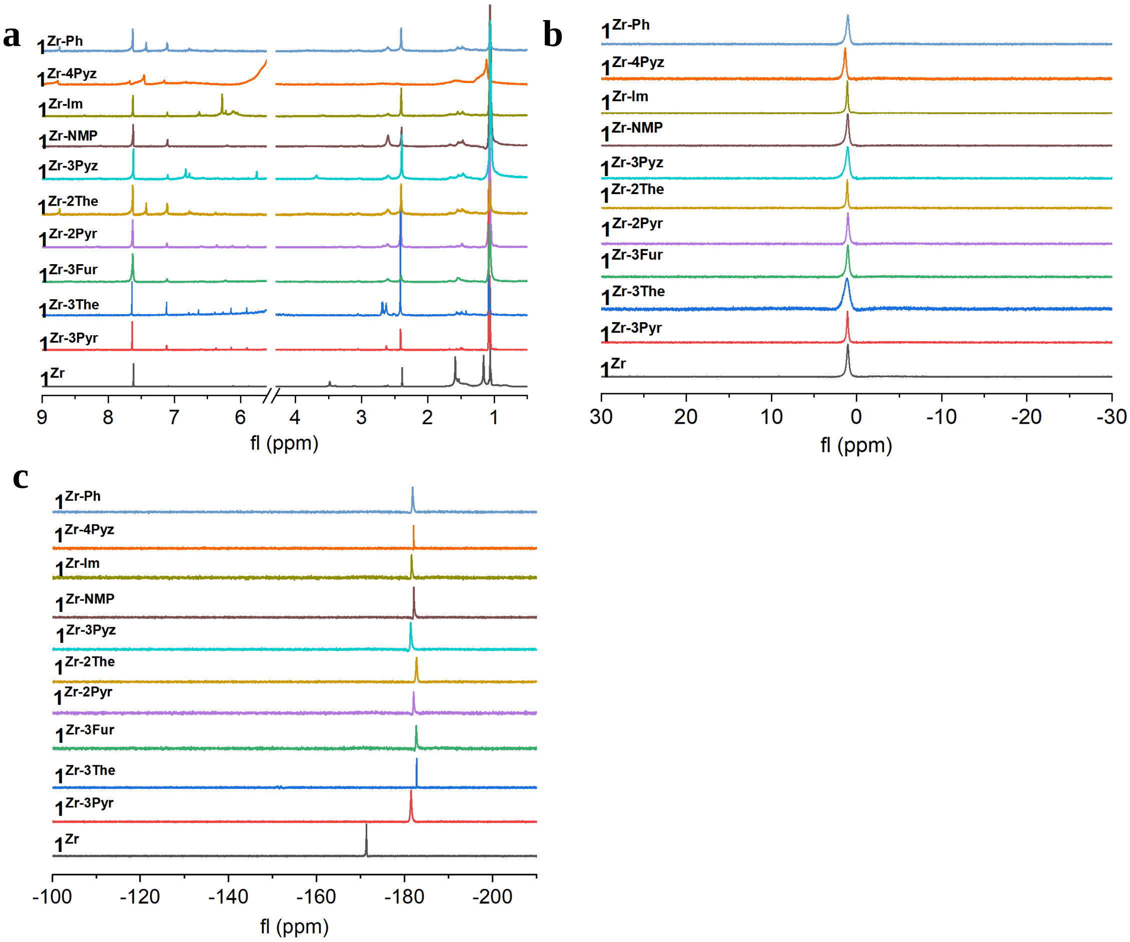

Figure S23 a. ^{1}H, **b.** ^{11}B, and **c.** ^{19}F NMR of $\mathbf{1}^{\mathbf{Zr\text{-}Ar}}$ digested by NaOD/D_2O

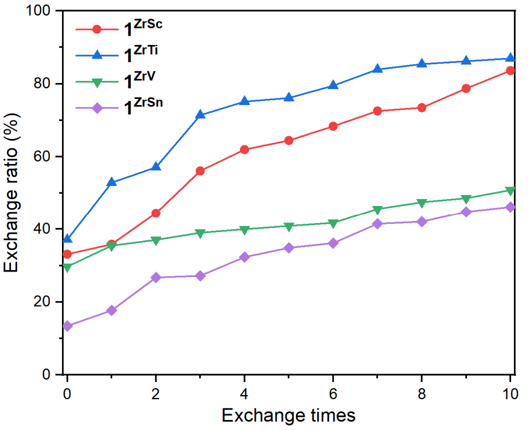

Figure S24. Kinetic results for the PSME process of $\mathbf{1^{ZrM}}$

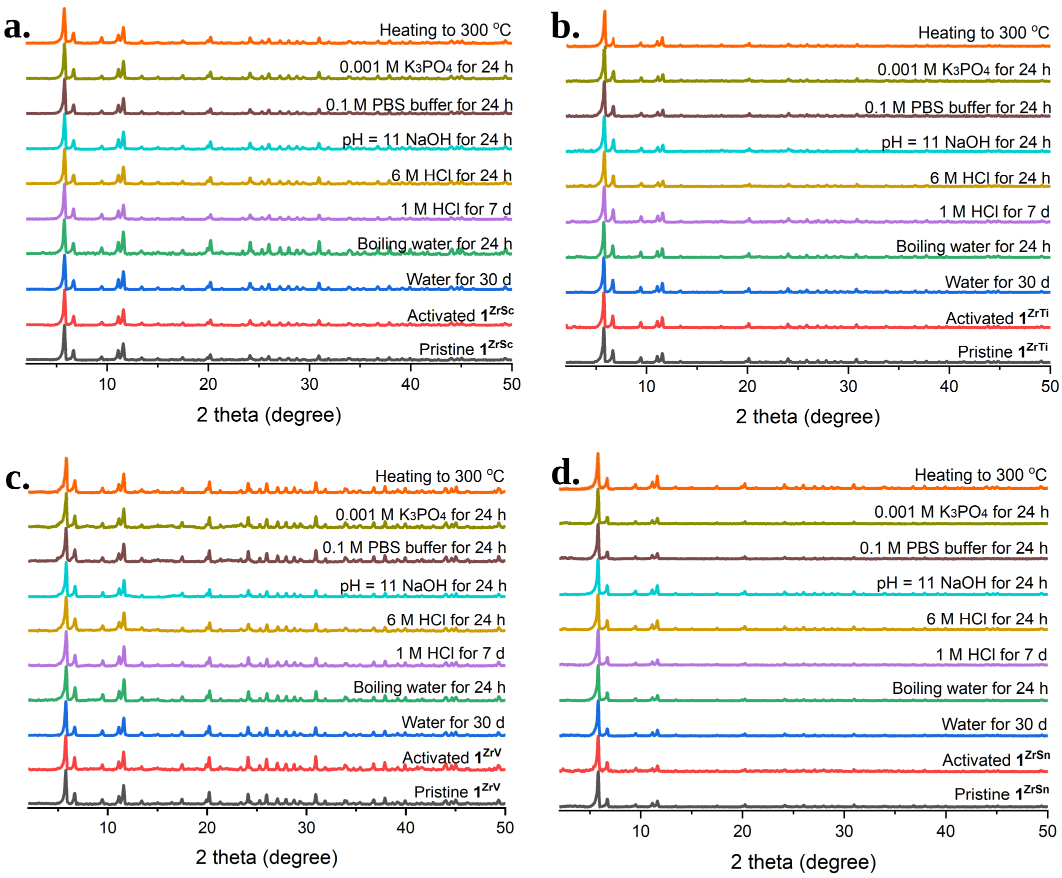

Figure S25. PXRD patterns of $\mathbf{1^{ZrM}}$; **a.** $\mathbf{1^{ZrSc}}$, **b.** $\mathbf{1^{ZrTi}}$, **c.** $\mathbf{1^{ZrV}}$, and **d.** $\mathbf{1^{ZrSn}}$.

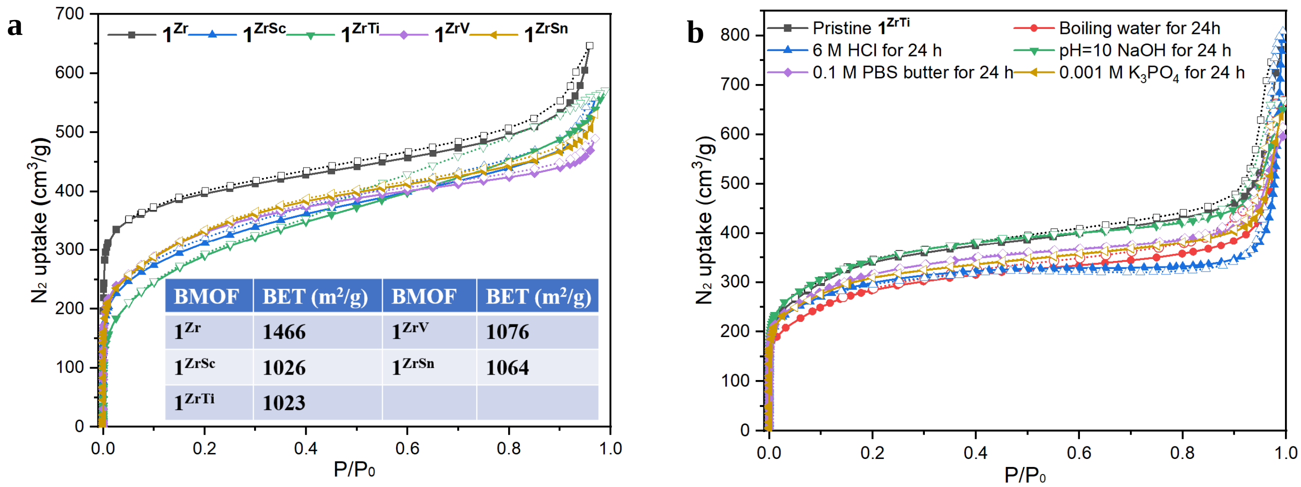

BMOF	BET (m^2/g)	BMOF	BET (m^2/g)
1^{Zr}	1466	1^{ZrV}	1076
1^{ZrSc}	1026	1^{ZrSn}	1064
1^{ZrTi}	1023		

Figure S26. a. The N_2 adsorption isotherms and BET areas (calculated using BETSI)[2] of $\mathbf{1^{ZrM}}$, **b.** The N_2 adsorption isotherms of $\mathbf{1^{ZrTi}}$ after various treatments.

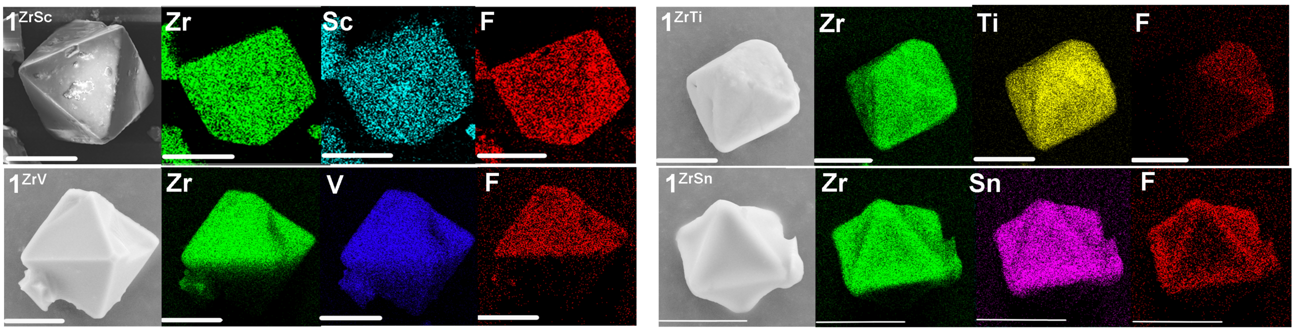

Figure S27. SEM images and EDS mappings of $\mathbf{1^{ZrM}}$

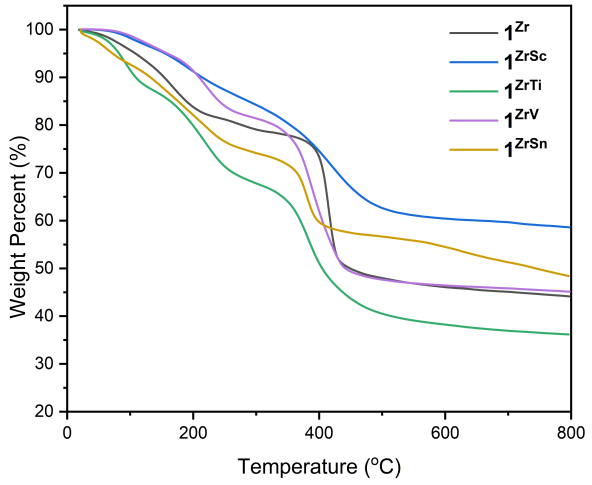

Figure S28. TGA curves of $\mathbf{1^{ZrM}}$

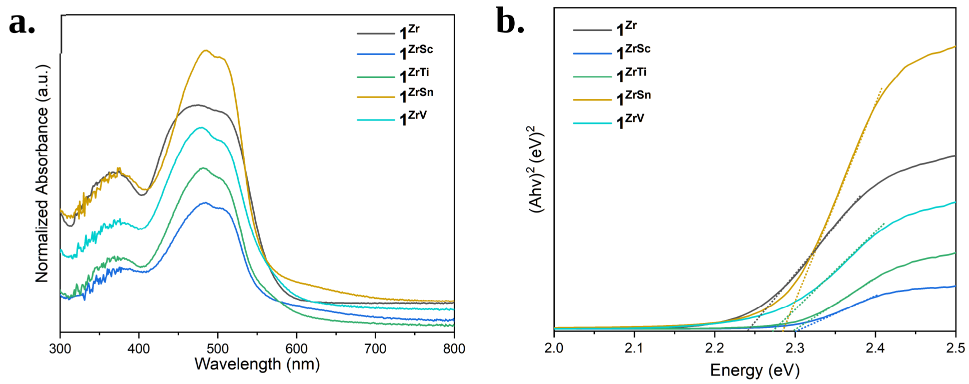

Figure S29. a. Solid-state UV–Vis spectra and **b.** Tauc plots of $\mathbf{1^{ZrM}}$

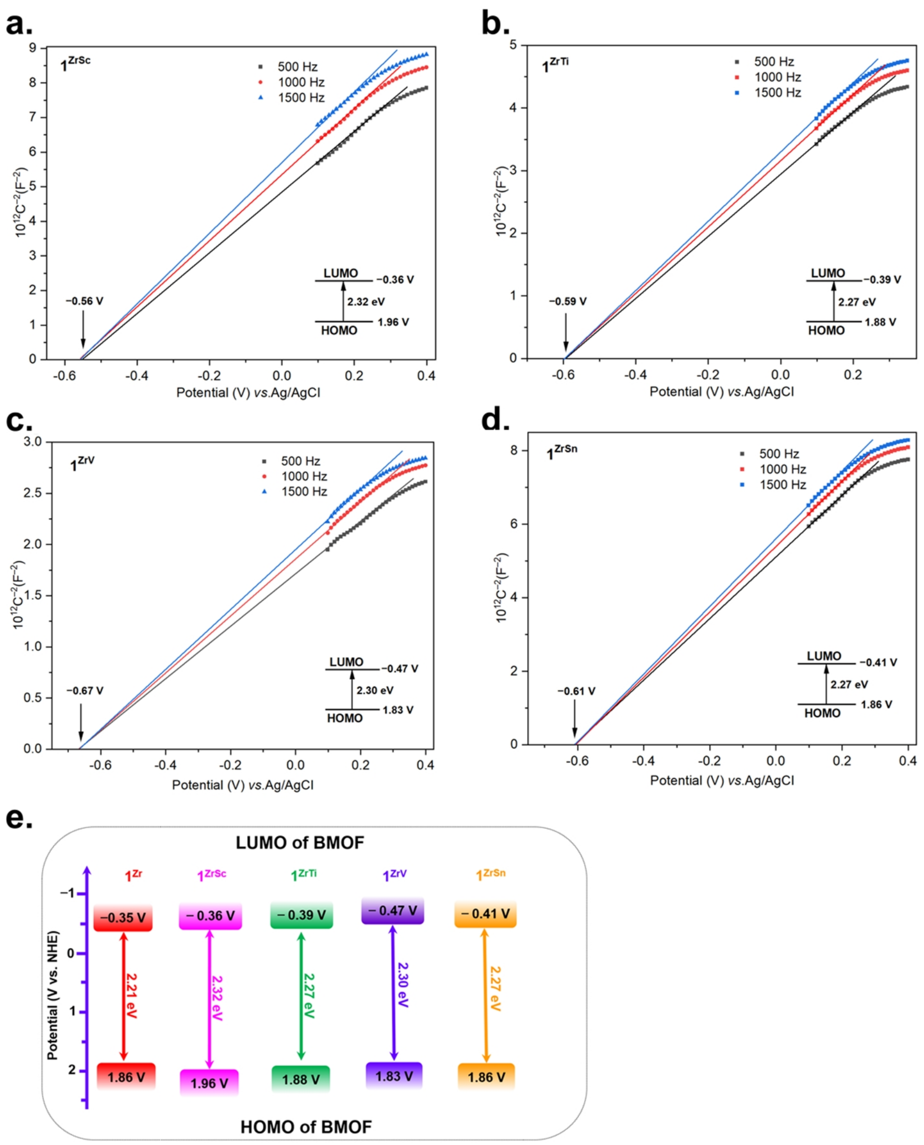

Figure S30. Mott–Schottky plots of **a**. $\mathbf{1^{ZrSc}}$, **b.** $\mathbf{1^{ZrTi}}$, **c**. $\mathbf{1^{ZrV}}$, **d**. $\mathbf{1^{ZrSn}}$, and **e**. HOMO-LUMO gap of $\mathbf{1^{ZrM}}$

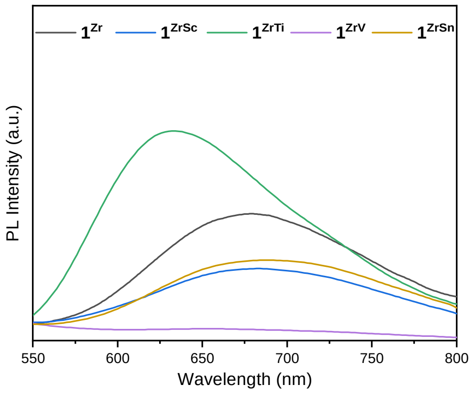

Figure S31. Solid-state PL spectra of **1^{ZrM}**

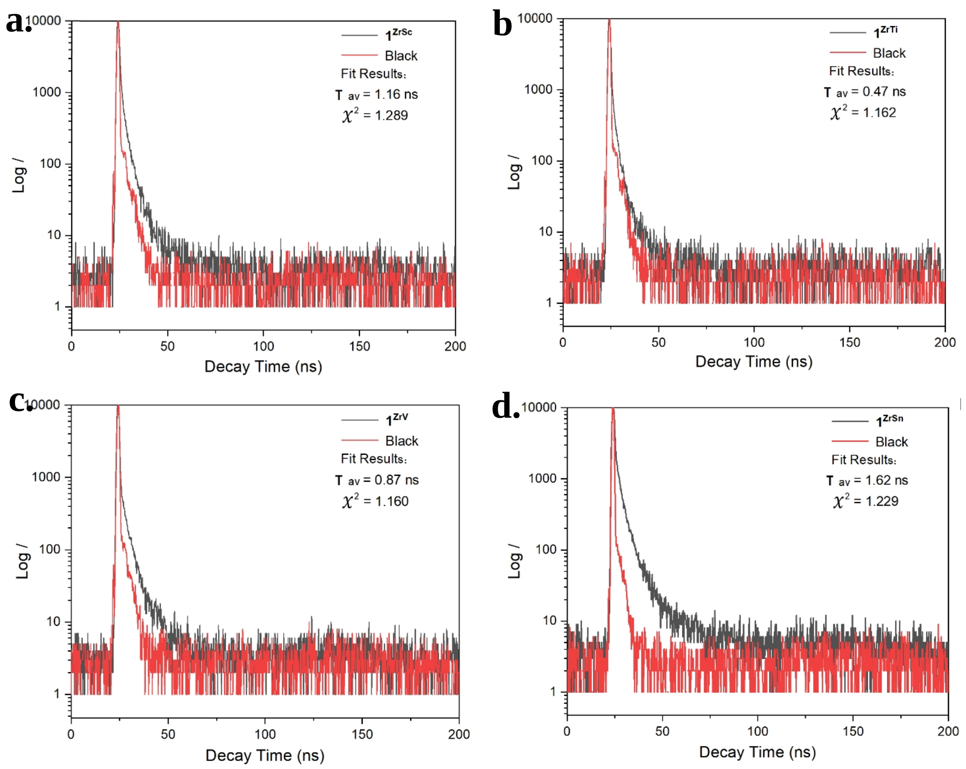

Figure S32. Fluorescence lifetimes of **1^{ZrM}**; **a. 1^{ZrSc}**, **b. 1^{ZrTi}**, **c. 1^{ZrV}**., and **d. 1^{ZrSn}**.

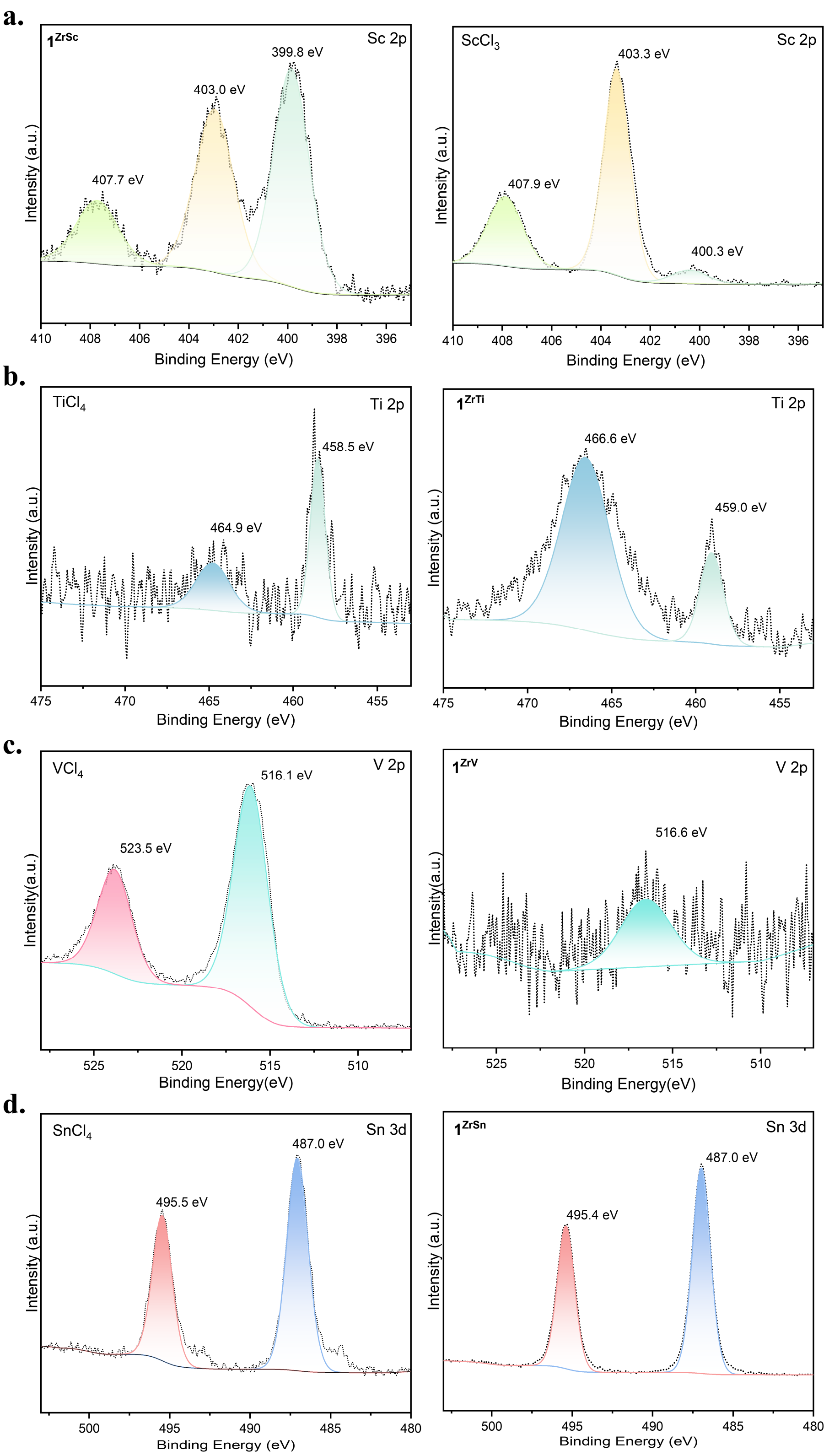

Figure S33. XPS spectra; **a**. $ScCl_3$ and**1**^{ZrSc}, **b**. $TiCl_4$ and **1**^{ZrTi}, **c**. VCl_4 and **1**^{ZrV}, **d.** $SnCl_4$ and **1**^{ZrSn}.

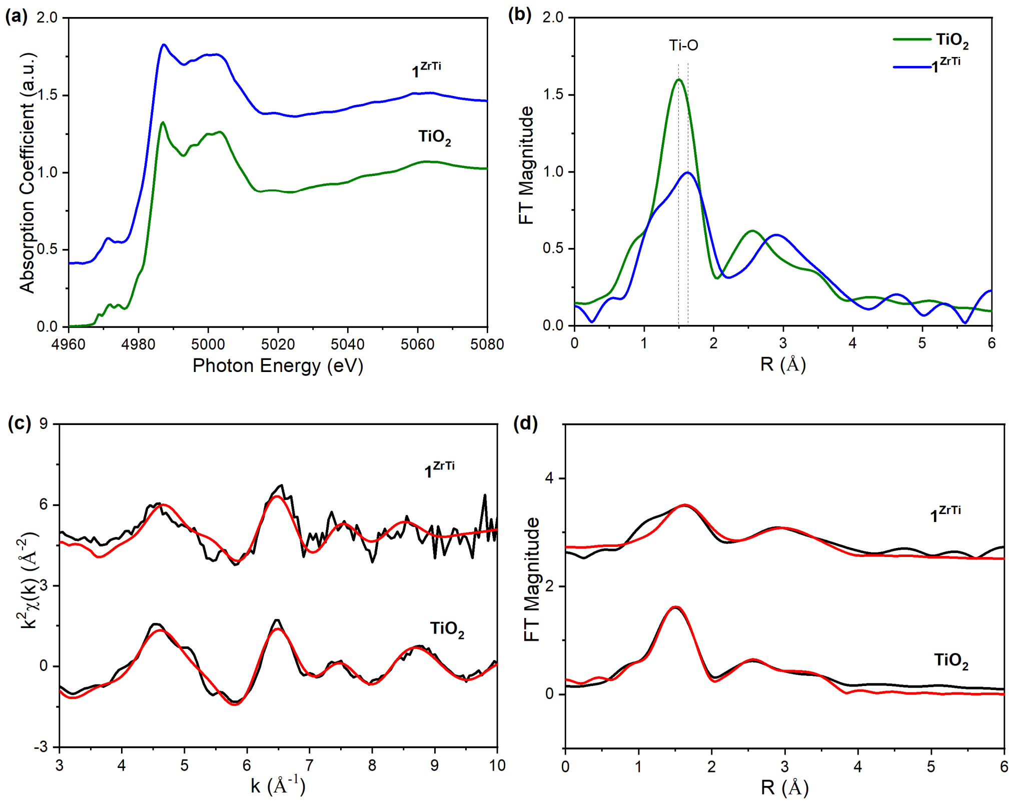

Figure S34. X-ray absorption fine structure (XAFS) spectroscopy of 1^{ZrTi}; **a**. The Ti *K*-edge XANES spectra of TiO_2 and $\mathbf{1^{ZrTi}}$, **b**. The Ti *K*-edge EXAFS data of TiO_2 and $\mathbf{1^{ZrTi}}$, **c.** k^2-weighted Ti *K*-edge experimental $\chi(k)$ data (black) and fit (red) in *k*-space, and **d.** the corresponding Fourier transform for TiO_2 and $\mathbf{1^{ZrTi}}$.

Table S2. Structure parameters of TiO_2 and $\mathbf{1^{ZrTi}}$ derived from the Ti *K*-edge EXAFS fitting results.

Sample	Paths	*N*	*R*(Å)	$\sigma^2(\times 10^{-3}$ Å$^2)$	ΔE_0(eV)
TiO_2	Ti-O	6.0 ± 0.5	1.97 ± 0.02	4.5 ± 1.5	0.7
	Ti-Ti	4.0 ± 0.5	2.05 ± 0.02	6.2 ± 3.0	-8.0
	Ti-Ti	4.0 ± 0.5	3.82 ± 0.02	6.2 ± 3.0	-8.0
$\mathbf{1^{ZrTi}}$	Ti-O	8.2 ± 0.8	1.56 ± 0.02	13.0 ± 4.5	12.0
	Ti-C	3.7 ± 0.4	3.20 ± 0.02	3.5 ± 1.0	10.7
	Ti-Ti(Zr)	3.9 ± 0.5	3.48 ± 0.02	4.5 ± 1.5	4.9

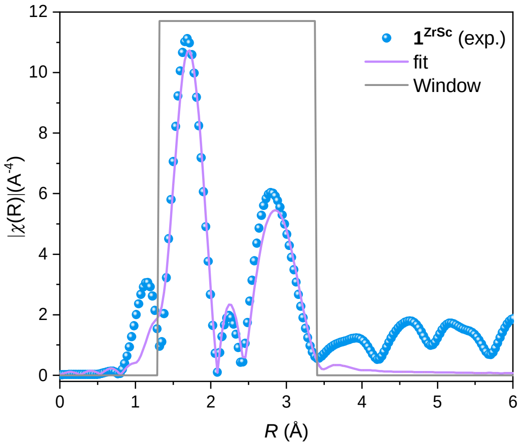

Figure S35. EXAFS fitting curves in *R*-space for $\mathbf{1}^{\mathbf{ZrSc}}$.

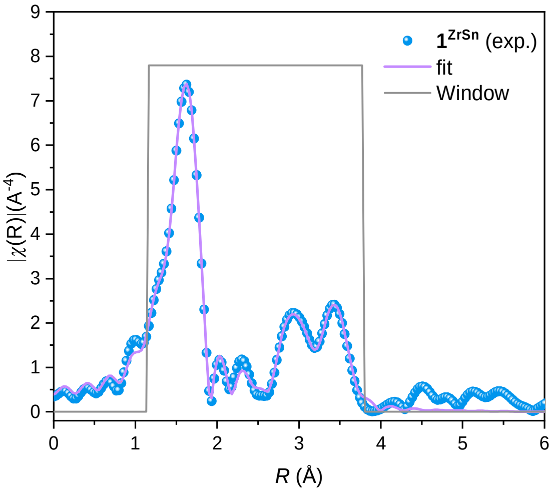

Figure S36. EXAFS fitting curves in *R*-space for $\mathbf{1}^{\mathbf{ZrSn}}$.

Table S3. Structure parameters of $\mathbf{1}^{\mathbf{ZrSn}}$ derived from the Sn *K*-edge EXAFS fitting results.

Sample	Path	N	R (Å)	$\sigma^2 \cdot 10^{-3}$	$S0^2$	ΔE0 (ev)	r-factor	fit range (Å)
$\mathbf{1}^{\mathbf{ZrSn}}$	Sn-O	2.0 (4)	1.908 (6)	27.5 (44)	0.960 (6)	3.94 (7)	0.0011	1.15 – 3.80
	Sn-O	2.0 (1)	2.054 (2)	10.8 (10)				
	Sn-O	3.0 (0.5)	2.038 (0.7)	3.4 (2)				
	Sn-C	4.4 (2)	3.156 (2)	2.3 (4)				
	Sn-O	2.7 (1)	3.312 (2)	0.7 (3)				
	Sn-Zr2	2.1 (2)	3.608 (3)	7.7 (7)				
	SnZr3	2.1(2)	3.767 (2)	4.1 (4)				

Table S4. Structure parameters of $\mathbf{1}^{\mathbf{ZrSc}}$ derived from the Sc *K*-edge EXAFS fitting results.

Sample	path	N [(b)]	R (Å) [(c)]	$\sigma^2 \cdot 10^{-3}$ [(d)]	S0^2 [(a)]	ΔE0 (ev) [(e)]	r-factor	fit range (Å)
$\mathbf{1}^{\mathbf{ZrSc}}$	Sc-O	5.0 (5)	2.097 (4)	1.2 (3)	0.95 (4)	-5.1 (4)	0.016	1.3-3.4
	Sc-O	2.0 (6)	1.935 (29)	13.4 (46)				
	Sc-O	1.9 (4)	2.598 (18)	3.2 (22)				
	Sc-C	4.6 (8)	3.211 (13)	1.4 (13)				
	Sc-Zr2	3.4 (4)	3.367 (8)	6.1 (7)				
	Sc-O	4.3 (9)	3.617 (16)	2.5 (21)				

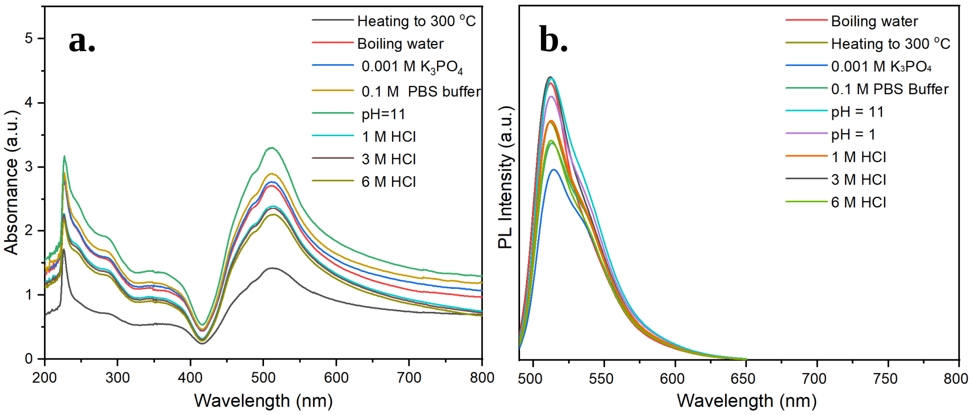

Figure S37. a. UV−Vis and **b.** PL spectra of $\mathbf{1}^{\mathbf{ZrTi}}$ under different conditions

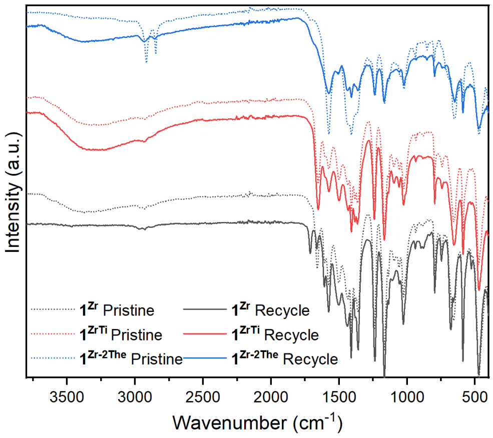

Figure S38. IR spectra of $\mathbf{1}^{\mathbf{Zr}}$, $\mathbf{1}^{\mathbf{Zr\text{-}2The}}$, and $\mathbf{1}^{\mathbf{ZrTi}}$

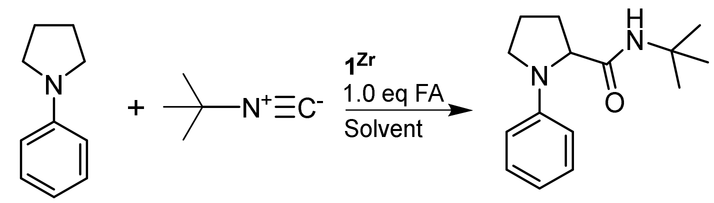

6a 7a 8a

Table S5. Optimization of the photocatalysis (I)

	Solvent	Temp /^{o}C	Cat. Loading	Light (LED/W)	Time/h	Conv. (%)
1	DMSO	r.t	1%	5 W	8	40
2	DMF	r.t	1%	5 W	8	17
3	MeOH	r.t	1%	5 W	8	22
4	MeCN	r.t	1%	5 W	8	16
5	THF	r.t	1%	5 W	8	20
6	DCE	r.t	1%	5 W	8	ND
7	DCM	r.t	1%	5 W	8	30
8	Toluene	r.t	1%	5 W	8	16
9	Dioxane	r.t	1%	5 W	8	ND
10	MTBE	r.t	1%	5 W	8	48
11	DCE	r.t	1%	5 W	8	ND
12	Chloroform	r.t	1%	5 W	8	78
13	Chloroform	r.t	2%	5 W	8	78
14	Chloroform	r.t	0.5%	5 W	8	73
15	Chloroform	r.t	1%	10 W	8	78
16	Chloroform	r.t	1%	5 W	24	78
17	Chloroform	r.t	1%	5 W	4	53

※ The photocatalyst loading was based on the bodipy unit in each counterpart.

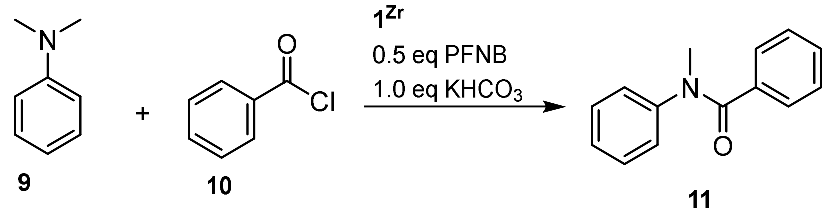

Table S6. Optimization of the photocatalysis (II)

	Solvent	Temp/ ^{o}C	Cat. Loading	Light (LED/W)	$KHCO_3$/ eq	PFNB/ eq	Time/h	Conv. (%)
1	DMSO	r.t	1%	5 W	-----	-----	12	ND
2	DMF	r.t	1%	5 W	-----	-----	12	15
3	MeOH	r.t	1%	5 W	-----	-----	12	8
4	MeCN	r.t	1%	5 W	-----	-----	12	10
5	DCE	r.t	1%	5 W	-----	-----	12	ND
6	DCM	r.t	1%	5 W	-----	-----	12	38
7	Chloroform	r.t	1%	5 W	-----	-----	12	30
8	Toluene	r.t	1%	5 W	-----	-----	12	6
9	DCE	r.t	1%	5 W	-----	-----	12	11
10	THF	r.t	1%	5 W	-----	-----	12	45
11	THF	r.t	1%	5 W	-----	0.5	12	55
12	THF	r.t	1%	5 W	1	0.5	12	61
13	THF	r.t	1%	5 W	1	1	12	58
14	THF	r.t	1%	5 W	2	0.5	12	60
15	THF	r.t	2%	5 W	1	0.5	12	63
16	THF	r.t	1%	10 W	1	0.5	12	59
17	THF	45	1%	5 W	1	0.5	12	73
18	THF	60	1%	5 W	1	0.5	12	74
19	THF	45	1%	5 W	1	0.5	24	75
20	THF	45	1%	5 W	1	0.5	6	57

※ The photocatalyst loading was based on the bodipy unit in each counterpart.

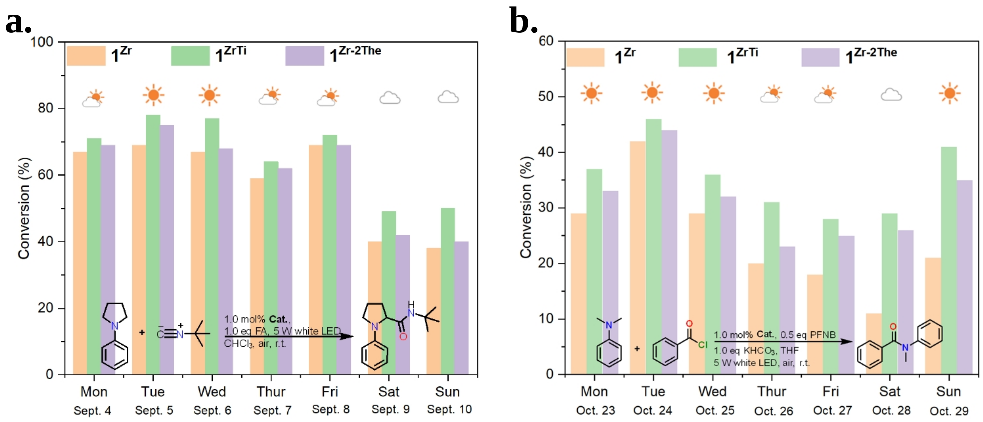

Figure S39. Sunlight-driven the synthesis of **a.** 8a and **b.** 11aa.

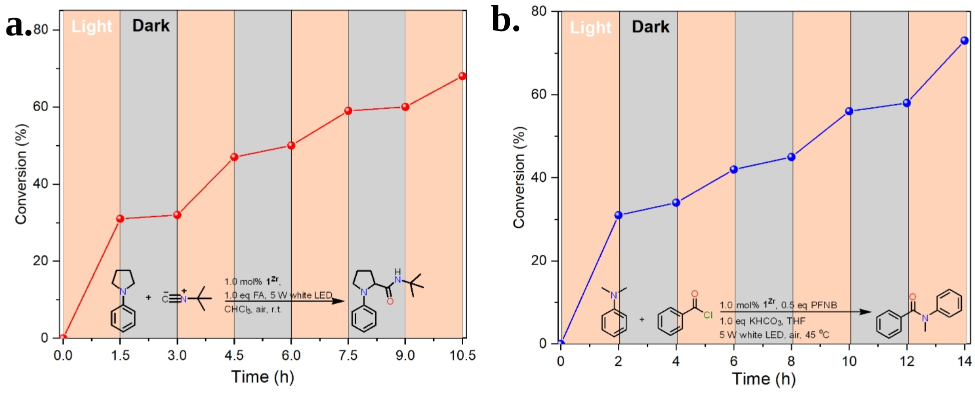

Figure S40. The kinetic curves for the synthesis of **a.** 8a and **b.** 11aa with visible light on/off.

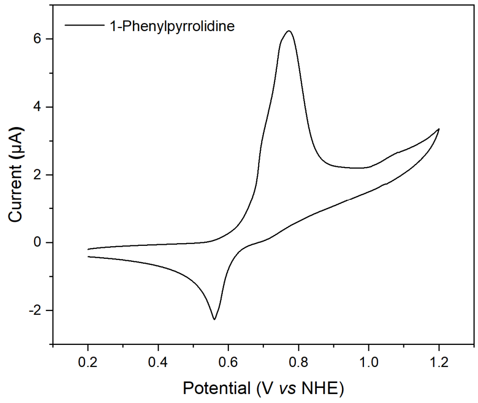

Figure S41. Cyclic voltammetry (CV) curve of 1-phenylpyrrolidine (**6a**).

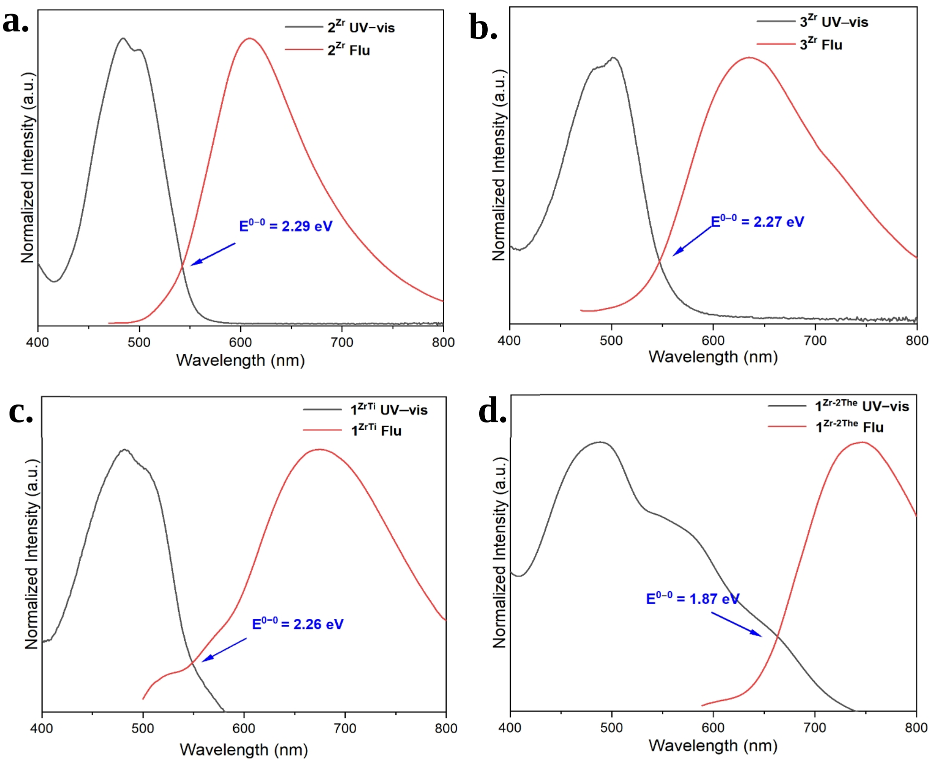

Figure S42. The excited state energy E^{0-0} of **a. 2^{Zr}**, **b. 3^{Zr}**, **c. 1^{ZrTi}**, and **d. $1^{Zr-2The}$**

Table S7. The photocatalytic performance comparison

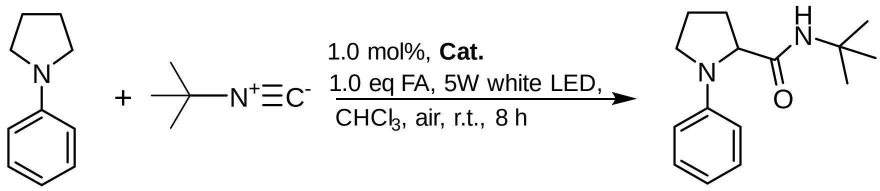

6a **7a** **8a**

Cat.	1^{Zr}	2^{Zr}	3^{Zr}	1^{Zr-Ph}	$1^{Zr-4Pyz}$	1^{Zr-IM}	1^{Zr-NMP}	$1^{Zr-3Pyz}$
Conversion (%)	78	80	78	84	87	86	82	85
Cat.	$1^{Zr-2The}$	$1^{Zr-2Pyr}$	$1^{Zr-3Fur}$	$1^{Zr-3The}$	$1^{Zr-3Pyr}$	1^{ZrSc}	1^{ZrTi}	1^{ZrV}
Conversion (%)	87	80	80	84	81	85	88	80
Cat.	1^{ZrSn}							
Conversion (%)	79							

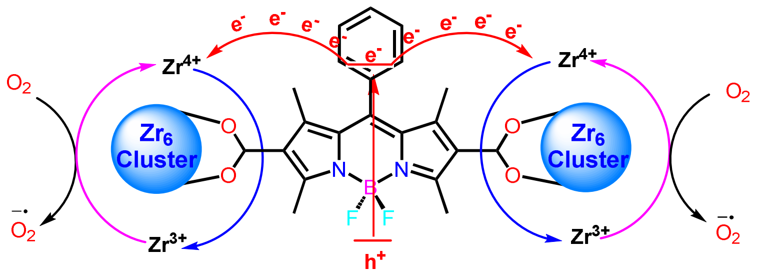

Figure S43. The proposed electron transfer from the bodipy backbone to the Zr-4d orbital

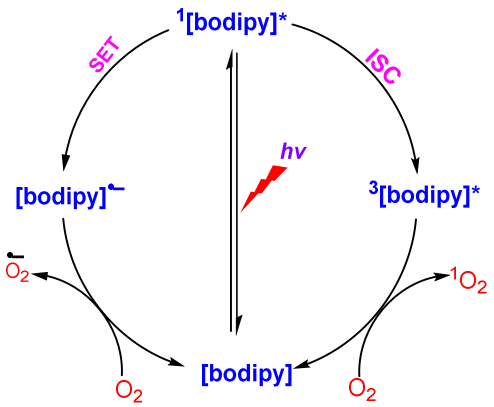

Figure S44. Proposed reaction mechanism by the bodipy monomer. SET = Single-electron transfer; ISC = Intersystem crossing.

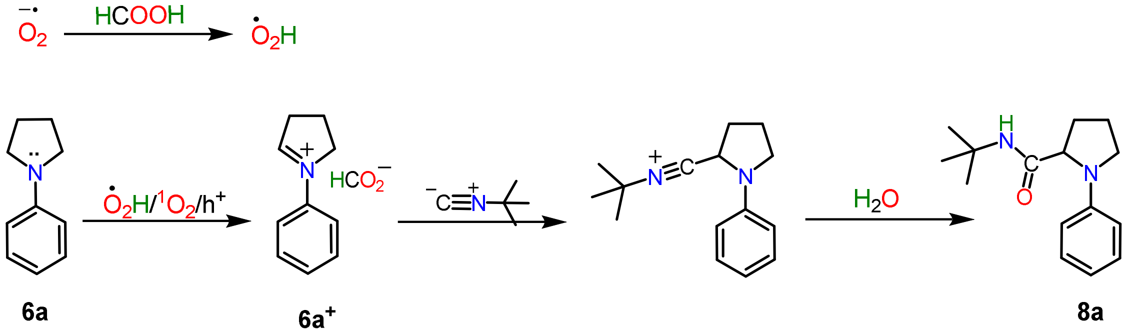

Proposed reaction mechanism of the synthesis of **8a**.

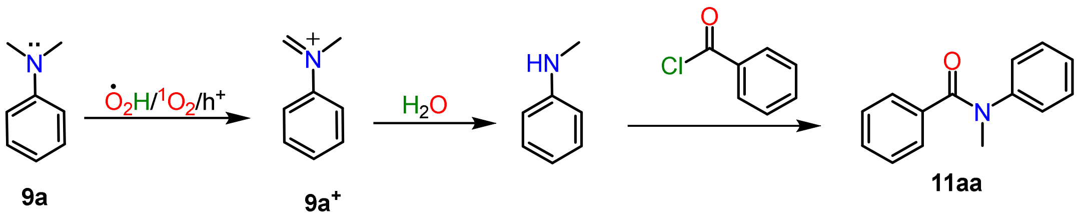

Proposed reaction mechanism of the synthesis of **11aa**.

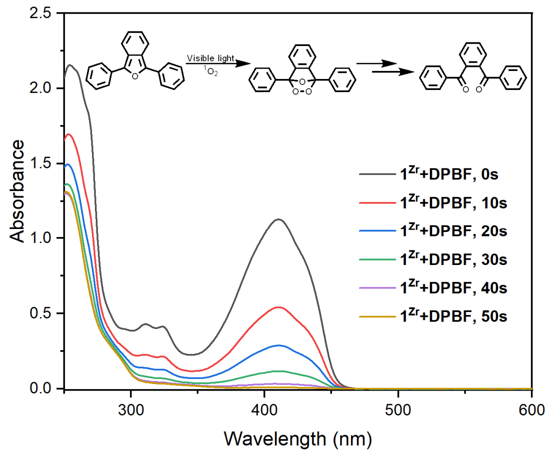

Figure S45. The detection of $^1O_2$ by using 1, 3-diphenylisobenzofuran (DPBF).

Table S8. Comparison of the photocatalytic performance of $\mathbf{1}^{\mathbf{ZrTi}}$ with varying Ti contents

Cat.	$\mathbf{1}^{\mathbf{Zr}}$	$\mathbf{1}^{\mathbf{ZrTi}}$							
Exchange Times	----	0	1	2	3	4	5	6	7
Ti content (%)	0	37	53	57	71	75	76	79	85
Conv. (%)	78	83	89	89	88	89	89	88	89

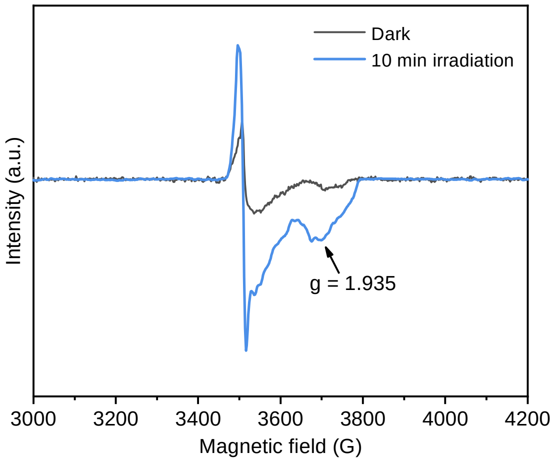

Figure S46. *In situ* EPR signal of $\mathbf{1}^{\mathbf{ZrTi}}$.

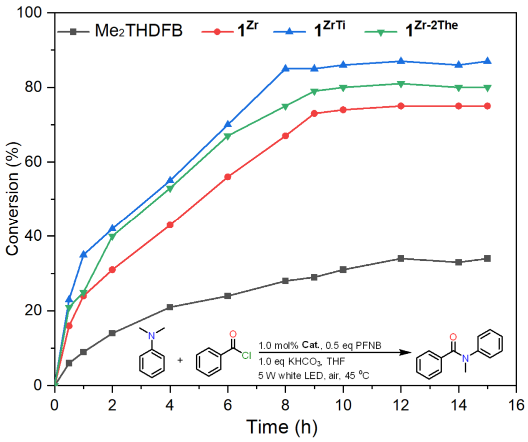

Figure S47. The kinetic curve of the synthesis of **11aa**.

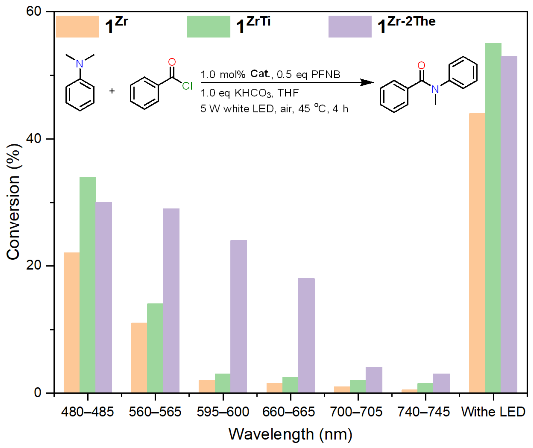

Figure S48. The synthesis of **11aa** promoted by monochromic and white lights within 4 h.

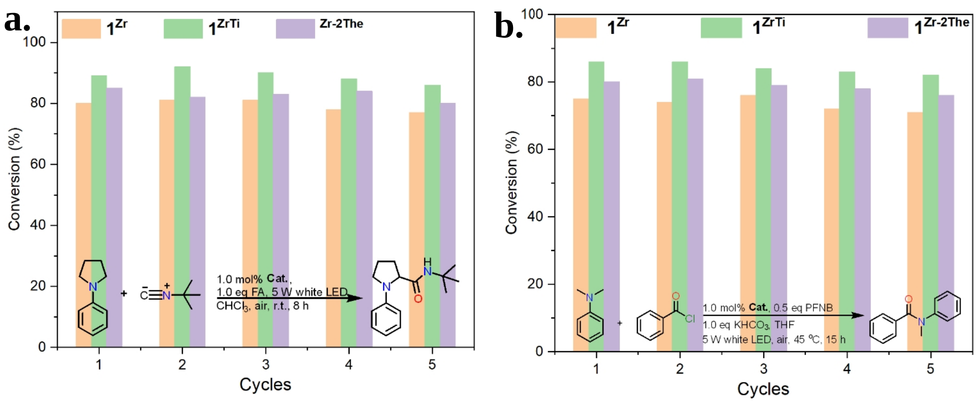

Figure S49. The recycling experiments for the synthesis of **a.** 8a and **b.** 11aa.

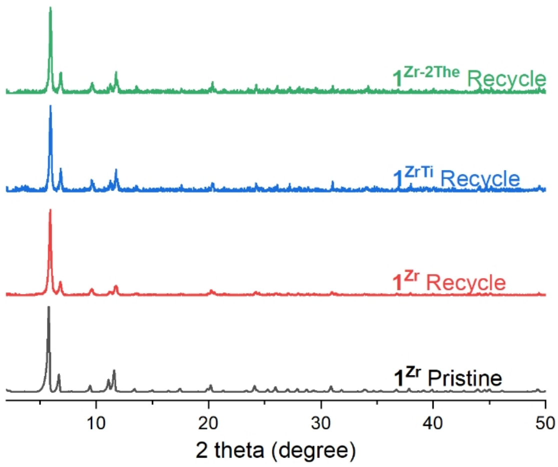

Figure S50. PXRD patterns of recoveries.

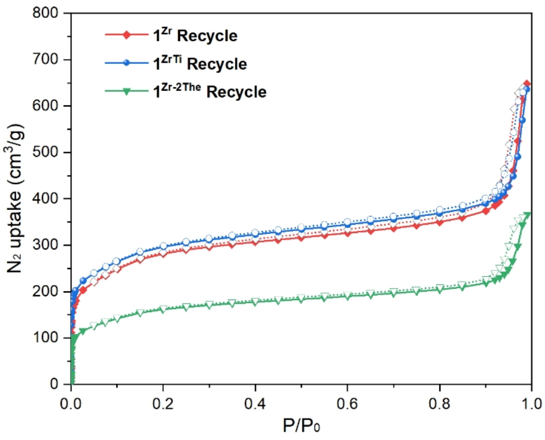

Figure S51. N_2 adsorption isotherms of the recoveries.

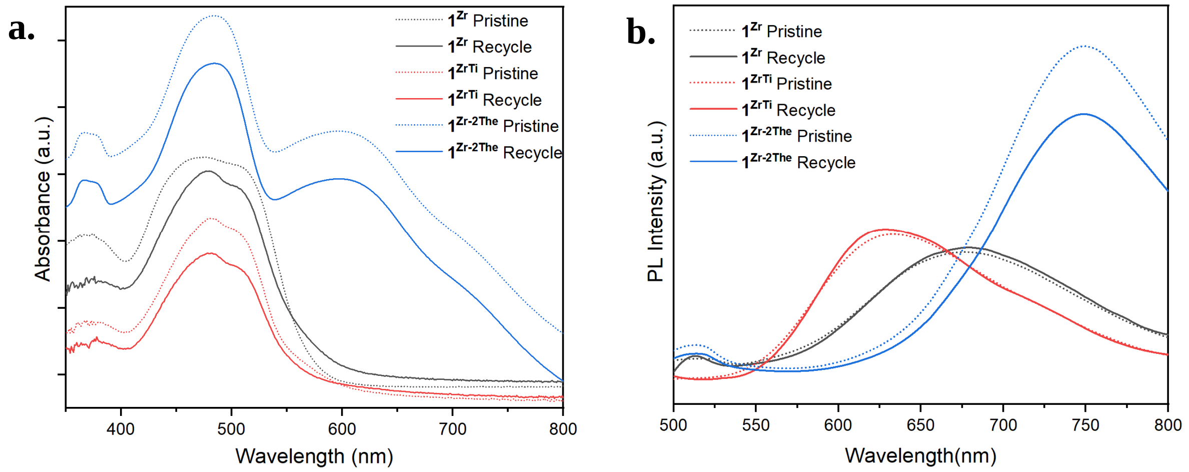

Figure S52. a. UV−Vis and **b.** steady-state PL spectra of the recoveries of $\mathbf{1^{Zr}}$, $\mathbf{1^{ZrTi}}$, and $\mathbf{1^{Zr\text{-}The}}$.

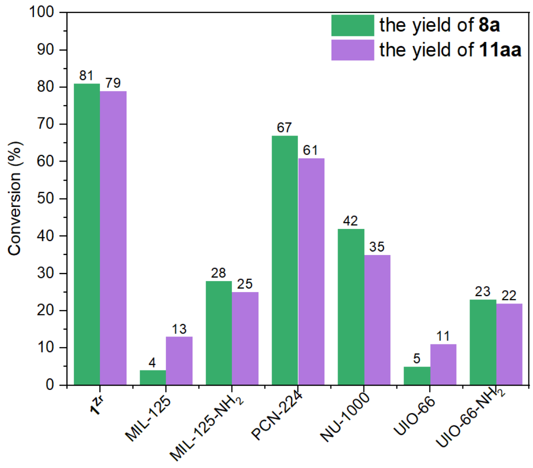

Figure S53. The comparison of photocatalytic activities with other solids.

8 Modelling Details

8.1 Density functional theory (DFT) calculations

Spin-polarized periodic DFT calculations were conducted using the Quantum ESPRESSO (QE) simulation package.[3, 4] The calculations employed the primitive cell of **1**$^{\mathbf{Zr}}$ crystal structure, which consists of 236 atoms, including 6 Zr, 90 C, 32 O, 12 N, 12 F, 6 B, and 78 H, as depicted in **Figure S54**. For geometry optimizations, the PBEsol exchange–correlation functional was utilized,[5] while single-point energy and electronic property calculations were performed using the HSE06 functional,[6] incorporating 25% exact exchange. This methodology, referred to as the HSE06//PBEsol protocol, has been demonstrated to balance accuracy and computational efficiency.[7, 8] The D3BJ dispersion correction scheme is also used to account for dispersion interactions.[9] Core electrons were described using the SSSP pseudopotentials for geometry optimizations and optimized norm-conserving Vanderbilt (ONCV) pseudopotentials for single-point energy calculations,[10, 11] with a kinetic energy cutoff of 800 eV. A 1 × 1 × 1 k-point mesh (i.e., Γ-point sampling of the first Brillouin zone) was applied for both geometry optimizations and single-point calculations. The convergence criterion for self-consistent field (SCF) iterations was set to 10e-5 eV.

Both atomic positions and cell parameters were allowed to relax during geometry optimization, using a force convergence criterion of 0.01 eV/Å for each atom. Isolated O_2, H_2, N_2, and F_2 molecules were modeled within 20 × 20 × 20 Å^3 simulation cells. For these molecules, as well as graphite, bulk boron, and the metals Zr, Sc, Sn, V, and Ti, k-point mesh samplings of 5 × 5 × 5, 5 × 5 × 5, 5 × 5 × 5, 5 × 5 × 5, 20 × 20 × 7, 3 × 3 × 3, 8 × 8 × 5, 8 × 8 × 5, 6 × 6 × 6, 8 × 8 × 8, and 9 × 9 × 5, respectively, were applied. The HSE06//PBEsol protocol was used for all calculations to determine the energies. The crystallographic information file (CIF) for bulk boron used in the calculations was sourced from.[12] Visualization and graphics were generated using Materials Studio (7.0).[13]

For formation energy calculations, we followed the workflow outlined in.[14] The formation energy of a substitutional or doped metal impurity in charge state q, as a function of the electron chemical potential or Fermi energy (E_F), is given by:[15]

$$E_{form}^{Doped}\left(E_f, q\right) = E_{total}^{Doped} - E_{total}^{pristine} + n(\mu_{Zr} - \mu_M) + q(E_F + E_V) + \Delta E_{correction}^{q} \quad (7.1)$$

where E_{total}^{Doped} is the total energy of the MOF containing the impurity M in charge state q; $E_{total}^{pristine}$ and E_V represent the total energy and the valence band maximum (VBM) energy of the pristine MOF, respectively; μ_{Zr} and μ_M are the chemical potentials of the removed Zr atom(s) and the added impurity atom(s), respectively; and $\Delta E_{correction}^{q}$ is the correction term for charged defects in the finite supercell. The correction term $\Delta E_{correction}^{q}$ was calculated using the method proposed by Freysoldt et al.,[16, 17] implemented via the **sxdefectalign** program.[18]

Chemical potentials represent the energy of the reservoirs with which atoms are exchanged. Their limits are constrained by the formation of crystal or gas phases and vary within a range determined by the formation enthalpy (ΔH_f) of the host MOF, expressed as:

$$\Delta H_f = \frac{1}{N_t}\left[E_{total} - \sum_i n_i \mu_i(R)\right] \quad (7.2)$$

where N_t is the total number of atoms in the MOF unit cell; i represents the constituent elements (Zr, O, C, H, B, F, N); n_i denotes the number of atoms of each species in the unit cell; and $\mu_i(R)$ is the chemical potential of each element in its reference reservoir. The values for $\mu_i(R)$ were calculated as follows: graphite for C, bulk phases for Zr and B, and gas phases for O, H, N, and F. Our calculations yielded a formation enthalpy of ΔH_f = -0.668 eV and a band gap of 2.34 eV for the pristine MOF, which agrees well with the obtained experimental band gap (~2.21 eV). In the context of Eq. 7.1, the chemical potential of Zr (μ_{Zr}) is restricted to a range defined by ΔH_f. When a metal dopant replaces a Zr atom in the MOF structure, creating a Zr deficit, μ_{Zr} was set to its Zr-poor limit (μ_{Zr}=μ_{Zr}(R)−ΔH_f). Conversely, dopants were considered in their metal-rich limit, with their chemical potentials (μ_M) obtained from their respective bulk phases (μ_M=μ_M(bulk)). The calculated valence band maximum (E_V) for pristine $\mathbf{1}^{\mathbf{Zr}}$ is -1.78 eV. The thermodynamic charge transition level, ε(q/q′), represents the Fermi level at which the formation energies of charge states q and q′ are equal. These ε(q/q′) levels are observable in experiments where the final charge state is allowed to fully relax to its equilibrium configuration following the transition.[14, 15]

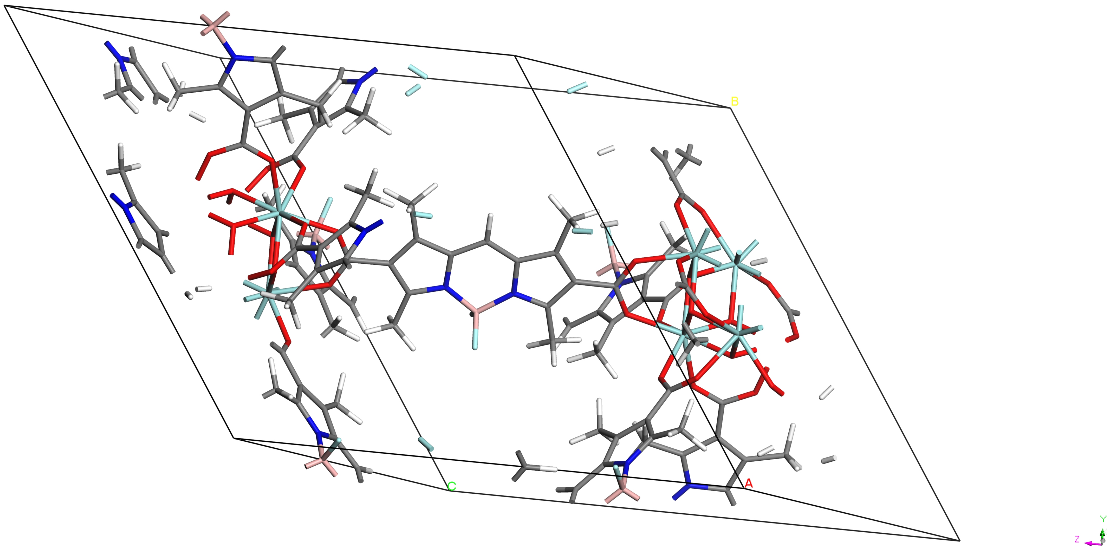

Figure S54. Primitive cell that considered for DFT calculations.

8.2 ToBaCCo protocol for assembly of 3-Pyr modified $\mathbf{1^{Zr}}$

To study the effect of linker post-modification on the BET area, we constructed a model system using the Topologically Based Crystal Constructor (ToBaCCo).[19] ToBaCCo is a computational algorithm that assembles a MOF from its building blocks by placing them on topological blueprints (periodic, abstract nets). The algorithm takes as input a pre-defined edge-transitive net consisting of nodes and edges, along with MOF building blocks as crystallographic information files (CIFs), with the connection points tagged. The building blocks are of two types: ‘nodular’ – corresponding to the metal node – and ‘connecting’ – corresponding to the linker. The nodular building blocks are placed onto the net nodes, while the connecting building blocks are placed onto the net edges. The building blocks are then rotated around their centroid to ensure a proper orientation, by matching the vectors of the building blocks with those of the net.

We designed the H_2THDFB linker in $\mathbf{1^{Zr}}$ with one branch functionalized with **3-Pyr** using Materials Studio (7.0).[13] Following this, the functionalized linker was subject to geometry optimization using molecular mechanics calculations. These calculations were performed using the Forcite module within Materials Studio, using the SMART algorithm, which is a cascade of the steepest descent, adjusted basis set Newton-Raphson, and quasi-Newton methods.[13] Herein, the bonded and short-range non-bonded interactions between the atoms of the ligand were modeled using the Universal Force Field (UFF)[20], while the long-range electrostatic interactions were modeled using a Coloumbic term. In UFF, the bond stretching is described by a harmonic term, angle bending by a three-term Fourier cosine expansion, the torsion and inversion by cosine-Fourier expansion terms, and the van der Waals interactions by the Lennard-Jones potential. A cutoff distance of 18.5 Å was used for interactions during the geometry optimization. The Ewald summation method was used for computing electrostatic interactions, with partial charges derived from the QEq protocol within Forcite. While we appreciate that in experimental scenarios, it is entirely feasible that some linkers have more than one branch functionalized while other linkers remain unfunctionalized, the primary intention here was to use this as a model system to validate our hypothesis on the reduction of the BET area. We thus generated a model MOF using ToBaCCo with only one branch modified. Here, the topology of the bare $\mathbf{1^{Zr}}$ – *fcu* – was used as the blueprint along with CIF files of the 12-connected Zr (Zr_6) and modified H_2THDFB as the building blocks. In doing so, however, the newly assembled MOF, due to the incorporation of the bulky group had occupancy issues pertaining to atomic overlaps, which needed to be manually fixed in Materials Studio[13]. In the case of minor overlaps, these were tackled by tilting one branch with respect to the second branch to accommodate both branches, while in the case of major overlaps, one branch was removed altogether. Once the structure was cleaned – i.e. minor and major overlaps were resolved, it was optimized using the Forcite module using the same force field described above. Following this, the PXRD pattern of the assembled MOF was simulated and compared to the experimentally derived pattern to confirm the validity of the structure. Subsequently, the pore properties of the MOF were

calculated using Zeo++[21] with a helium probe (probe radius = 1.32 Å) as outlined in **Table S9** This structure was subsequently used for 77 K N_2 isotherm calculations.

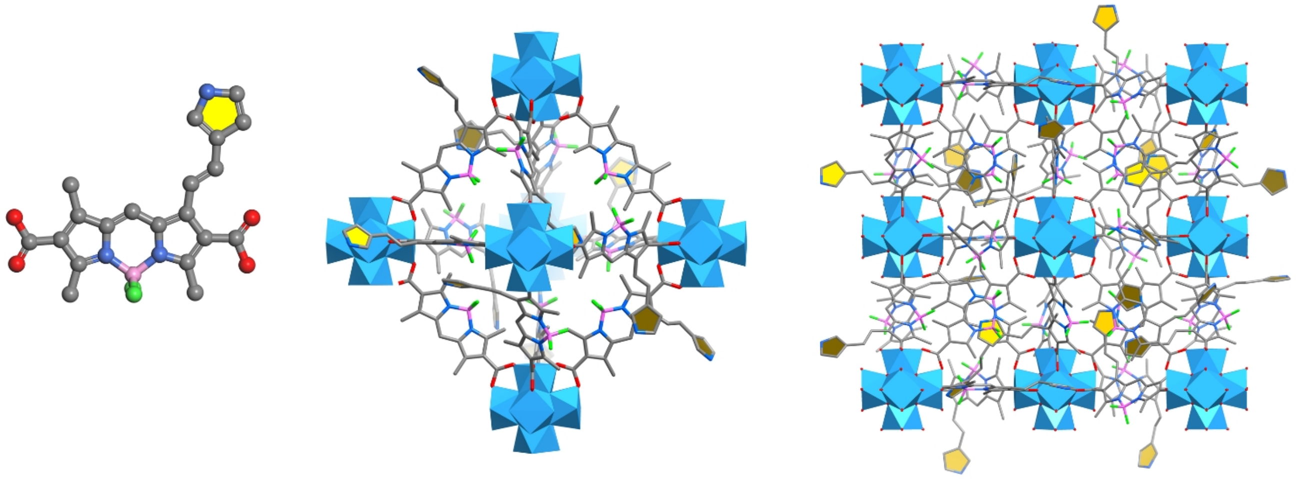

Figure S55. Illustration of **1**^{Zr} with one methyl group of H_2THDFB functionalized with one 3-Pyr (Zr_6 nodes, blue polyhedra; 3-Pyr groups, yellow polyhedra; O, red; B, pink; F, green; C, gray. Hydrogen atoms are omitted for clarity).

8.3 Pore Properties of Modified and Bare 1^{Zr}

All the pore properties were calculated using Zeo++. As expected, the incorporation of the functional group led to an increase in the density and decrease in the Largest Cavity Diameter (LCD) and Pore Limiting Diameter (PLD) of the bare MOF. There was a significant reduction in the accessible surface area and the corresponding volume fraction of the modified MOF.

Table S9. Pore properties of **1**^{Zr} and **1**$^{Zr\text{-}3Pyr}$

Name	Density[2] (g/cm^3)	LCD (Å)	PLD (Å)	Accessible Surface area (m^2/g)	Volume fraction
**1**^{Zr}	1.031	10.573	4.692	2050	0.481
**1**$^{Zr\text{-}3Pyr}$	1.228	19.357	3.501	1341	0.336

8.4 GCMC simulations

To calculate the BET area of **1^Zr^** post-modification, we performed grand canonical Monte Carlo (GCMC) simulations using the RASPA simulation package.[22] For all calculations, the atomic positions of the framework atoms were kept fixed. The non-bonded guest-guest and guest-host interactions were modeled using the Lennard-Jones (LJ) potential with a cutoff of 12.8 Å while the long-range electrostatic interactions were calculated using Ewald summation. The LJ potential parameters for the framework atoms were obtained from a combination of the Dreiding force field and UFF (refer to **Table S10**).[20, 23] The LJ parameters and partial charges for N_2 were obtained from Martin-Calvo et al. (**Table S11**).[24] The partial charges on the framework atoms were assigned using the EQeq protocol.[25] For interactions between atoms of different types, the Lorentz-Berthelot mixing rules were applied. To simulate the single-component adsorption isotherms for N_2 at 77 K, GCMC simulations were carried out at total pressures ranging from 1 – 99992 Pa. These simulations consisted of 10,000 initialization cycles followed by 40,000 production cycles for each point on the isotherm. Each cycle comprises of 'N' Monte Carlo moves – 'N' either being the number of guest molecules at the beginning of the cycle or 20, depending on whichever is lower. Guest insertion/deletion, translation, rotation, and reinsertion were each assigned equal probabilities for every Monte Carlo move.

Table S10. Force Field parameters for framework atoms used in GCMC simulations.

Atom	ε/k_B(K)	σ (Å)
C[1]	47.8562	3.47
N[1]	38.9492	3.26256
O[1]	48.1581	3.03315
H[1]	7.64893	2.84642
F[1]	36.4834	3.0932
B[1]	47.8058	3.58141
Zr[2]	34.7221	2.78317

[1]: Dreiding Force Field; [2]: Universal Force Field

Table S11. Force field parameters and charges for N_2 in the GCMC simulations.

Atom	ε/k_B(K)	σ (Å)	Charge
N_N2	38.298	3.306	-0.405
COM	0	0	0.810

The calculated isotherm has been compared to the experimentally obtained isotherm (**Figure S56**). The corresponding BET area calculated using BETSI[2] (**Figure S80**) was 181 m^2/g.

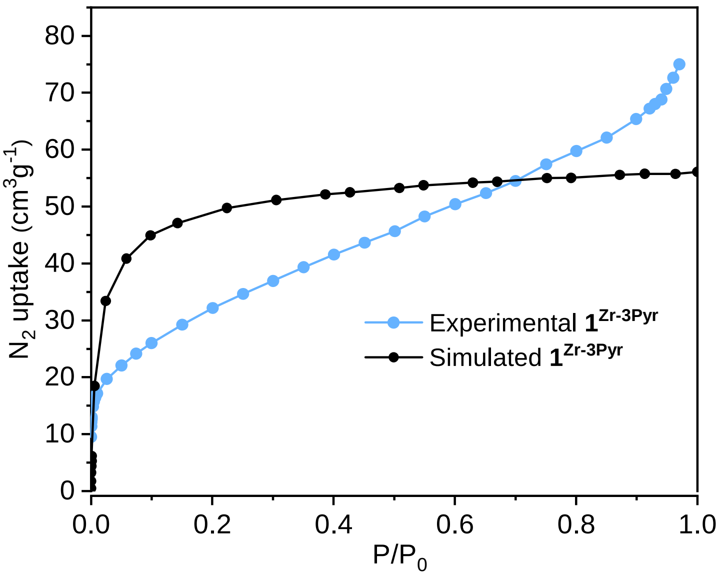

Figure S56. 77 K N_2 isotherms of $\mathbf{1}^{\mathbf{Zr\text{-}3Pyr}}$

8.5 DFT analysis of the formation of metal-exchanged variants.

To further validate our experimental findings on PSME of $\mathbf{1}^{\mathbf{Zr}}$, we conducted density functional theory (DFT) calculations to analyze the possibility of the formation of metal-exchanged variants. Here, we focused on the changes in formation energy associated with introducing exogenous metal ions (M) at Zr sites in $\mathbf{1}^{\mathbf{Zr}}$. Experimental results demonstrated the successful synthesis of metal-exchanged BMOFs ($\mathbf{1}^{\mathbf{ZrM}}$), where the exchanged metals exhibit stoichiometric redox activity while preserving the coordination environment. We investigated single- and double-atom exchange using Sc, Ti, V, and Sn as dopants to calculate the formation energies and identify the corresponding stable charge states of doped BMOFs as a function of Fermi energy (E_F). **Figure S57** illustrates the doping configurations considered. Following geometric relaxation, all dopants retained coordination environment, showing minor distortions compared to the pristine structure. **Table S12** summarizes the cell parameters and volumes for each defect analyzed in this study. In general, greater structural distortion was observed with V and Sc doping. Specifically, $Zr_4V_2^2$ exhibited the largest cell volume reduction (~3.4%), while $Zr_4Sc_2^1$ caused the greatest increase (~1.0%). The cell volume decrease with V dopants results from a ~12% reduction in V−O bond distances with neighboring oxygen atoms and a ~7% reduction in distances between V and neighboring Zr atoms. Conversely, the increase in cell volume with Sc dopants stems from a ~1.4% increase in Sc−O bond distances and a ~0.9% increase in distances between Sc and neighboring Zr atoms.

We further investigated the thermodynamic stability of dopants in their stable charge states. Stability was evaluated using formation energy calculations,[49] as dopants with comparatively high formation energies are unlikely to exist in significant concentrations. **Figure S57** presents the formation energies of metal dopants in $\mathbf{1}^{\mathbf{Zr}}$ as a function of E_F. The E_F ranges from the valence band maximum (VBM) to the conduction band maximum (CBM), corresponding to the band gap of $\mathbf{1}^{\mathbf{Zr}}$. The calculated band gap of $\mathbf{1}^{\mathbf{Zr}}$ is found to be 2.34 eV which aligns well with the experimental value of 2.25 eV. Negative slopes in the formation energy curves indicate stable charge states, while kinks in the curves mark the thermodynamic transition levels, $\varepsilon(q/q')$. V, Sn, and Ti dopants were found to be stable in a neutral charge state throughout the entire E_F range, as these metals typically exhibit the same oxidation state as the host Zr metal. In contrast, Sc dopants exhibited multiple stable charge states across different E_F intervals, reflecting the differing oxidation state of Sc compared to Zr. For Zr_5Sc_1, a neutral charge state was stable up to an E_F of 0.21 eV, while a single-negative charge state was stable at higher Fermi energy levels, indicating acceptor behavior in this region. Interestingly, when doped with two Sc atoms, the results revealed two transition levels: $\varepsilon(0/-1)$ around 0.1 eV and $\varepsilon(-1/-2)$ around 0.5 eV. This suggests that these defects can assume three different stable charge states depending on the Fermi energy. Ti and Sc were identified as the most stable dopants in $\mathbf{1}^{\mathbf{Zr}}$, with lower formation energies, while Sn dopants showed relatively high formation energies, making their presence unlikely at a relevant concentration. It is worth noting that the formation energy increases sharply with the number of doped metals, rendering configurations with more than two dopants unstable and rare, which is consistent with experimental observations.

Table S12. DFT optimized unit cell parameters of metal dopants in $\mathbf{1^{Zr}}$

	a (A)	b (A)	c (A)	alpha (degrees)	beta (degrees)	gamma (degrees)	Cell Volume (A^3)
Zr_6	18.619	18.622	18.630	59.993	59.992	90.010	4566.086
Zr_5Ti_1	18.526	18.534	18.610	59.893	59.831	89.480	4519.800
$Zr_4Ti_2^1$	18.471	18.460	18.569	59.905	59.770	89.327	4480.985
$Zr_4Ti_2^2$	18.513	18.522	18.518	59.736	59.647	89.420	4470.617
Zr_5V_1	18.486	18.539	18.566	59.861	59.792	89.520	4494.257
$Zr_4V_2^1$	18.394	18.404	18.483	59.919	59.842	89.462	4428.839
$Zr_4V_2^2$	18.436	18.457	18.437	59.823	59.514	89.391	4414.981
Zr_5Sn_1	18.557	18.553	18.582	60.286	60.251	90.332	4547.269
$Zr_4Sn_2^1$	18.628	18.627	18.558	59.858	59.794	89.889	4533.694
$Zr_4Sn_2^2$	18.575	18.578	18.581	59.984	60.124	90.047	4539.534
Zr_5Sc_1	18.593	18.610	18.718	59.825	59.775	89.260	4581.157
$Zr_4Sc_2^1$	18.631	18.633	18.772	59.807	59.744	89.158	4610.000
$Zr_4Sc_2^2$	18.695	18.707	18.700	59.667	59.732	89.475	4603.409

Note: $Zr_xM_y^1$ and $Zr_xM_y^2$ indicate two different doping configurations.

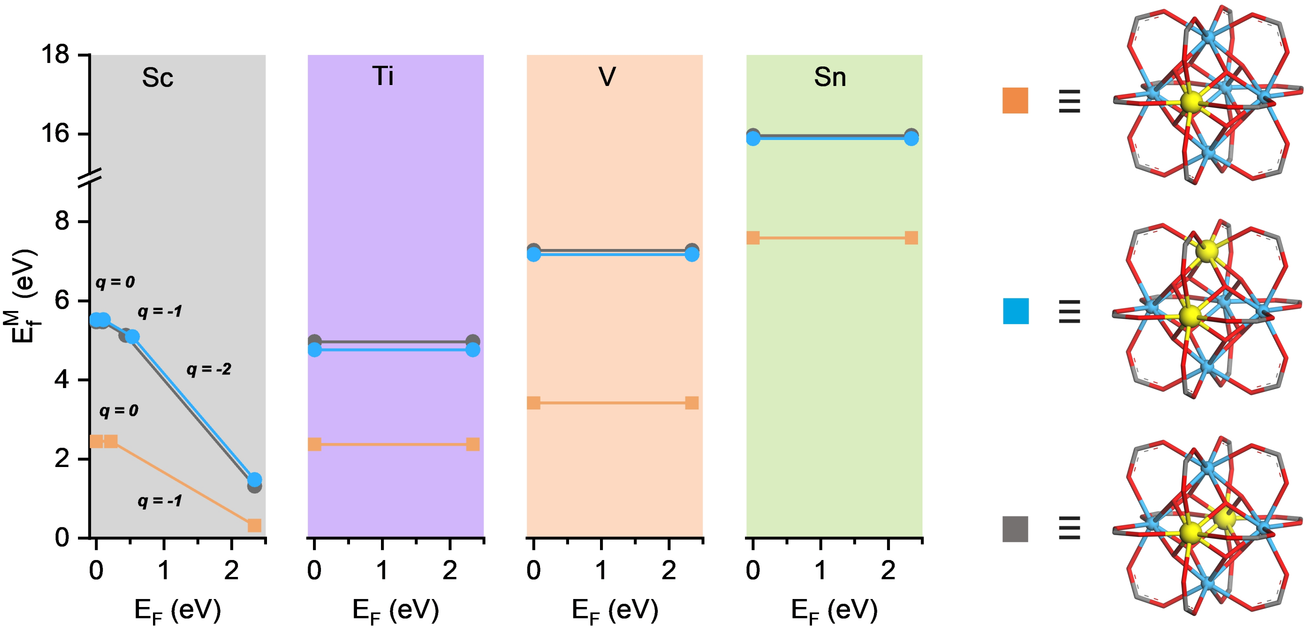

Figure S57. Formation energies of metal dopants in $\mathbf{1^{Zr}}$ under a Zr-poor condition, shown as a function of Fermi energy within the band gap. For Sc, slopes (q) represent stable charge states, and kinks indicate thermodynamic charge transition levels ε(q/q').

9 NMR

9.1 The direct C(sp^3)-H carbamoylation of saturated aza-heterocycles

N-(tert-butyl)-1-phenylpyrrolidine-2-carboxamide

^{1}H NMR (400 MHz, $CDCl_3$) δ 7.26 (dd, *J* = 8.6, 7.4 Hz, 2H), 6.81 (t, *J* = 7.3 Hz, 1H), 6.63 (d, *J* = 8.0 Hz, 2H), 6.33 (s, 1H), 3.86 – 3.81 (m, 1H), 3.62 (ddd, *J* = 9.0, 7.1, 2.0 Hz, 1H), 3.24 – 3.16 (m, 1H), 2.22 (ddd, *J* = 9.4, 7.5, 5.4 Hz, 2H), 2.04 – 1.89 (m, 2H), 1.29 (s, 9H). **^{13}C NMR (101 MHz, $CDCl_3$)** δ 173.05, 147.66, 129.24, 118.10, 113.15, 65.30, 50.71, 49.71, 31.39, 28.64, 24.22. **HRMS (ESI-TOF)**: m/z calculated for $C_{15}H_{23}ON_2$ $[M+H]^+$: 247.1805. Found: 247.1803.

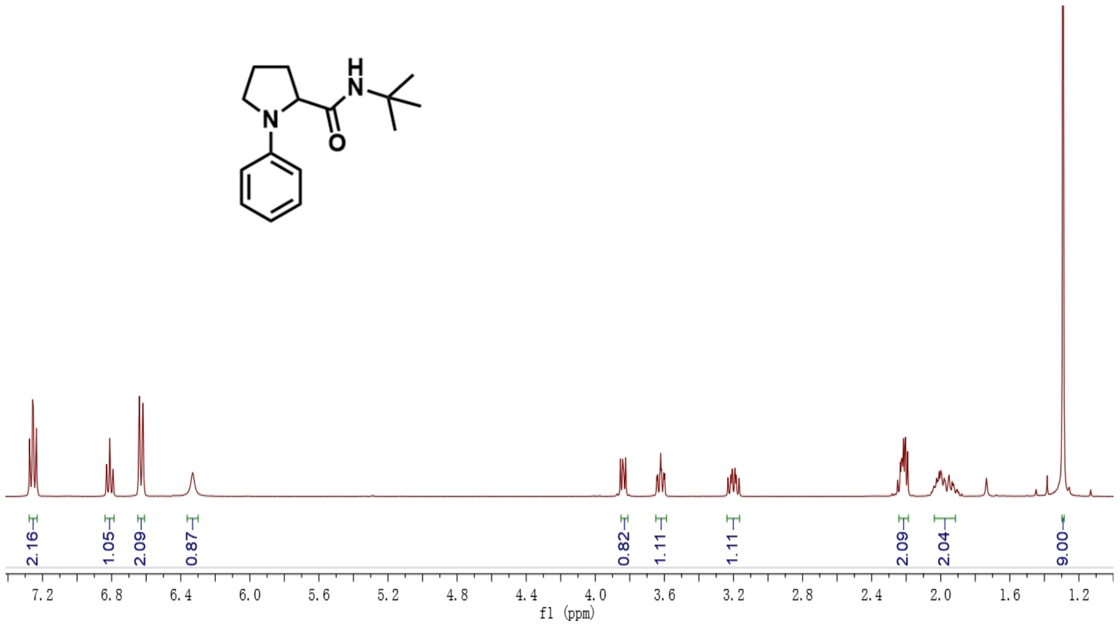

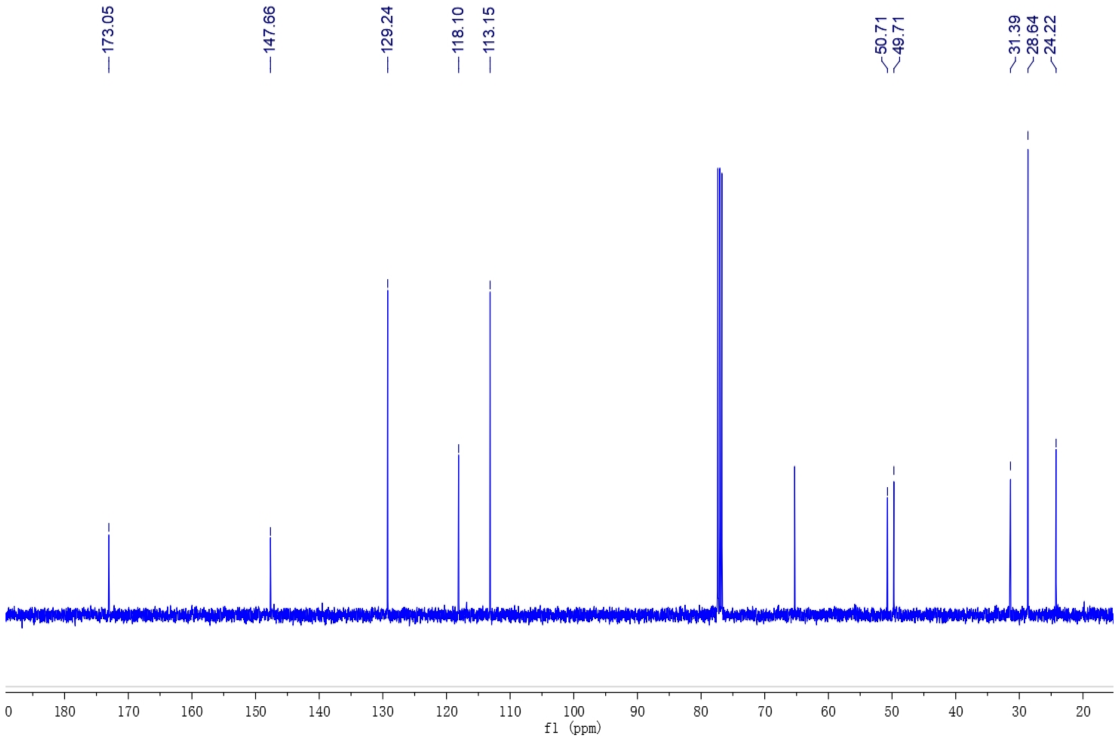

N-(tert-butyl)-1-(o-tolyl)pyrrolidine-2-carboxamide

^{1}H NMR (400 MHz, $CDCl_3$) δ 7.19 – 7.09 (m, 2H), 7.02 – 6.92 (m, 2H), 6.74 (s, 1H), 4.07 (t, *J* = 7.3 Hz, 1H), 3.64 (dt, *J* = 9.4, 6.6 Hz, 1H), 2.84 (dt, *J* = 9.4, 7.1 Hz, 1H), 2.44 – 2.38 (m, 1H), 2.36 (s, 3H), 2.04 – 1.87 (m, 3H), 1.20 (s, 9H). **^{13}C NMR (101 MHz, $CDCl_3$)** δ 172.96, 148.48, 131.59, 131.38, 127.00, 123.20, 118.89, 65.71, 54.63, 50.35, 31.32, 28.67, 24.89, 19.64. **HRMS (ESI-TOF)**: m/z calculated for $C_{16}H_{24}ON_2K$ $[M+K]^+$: 299.1520. Found: 299.1524.

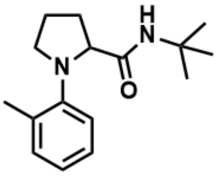

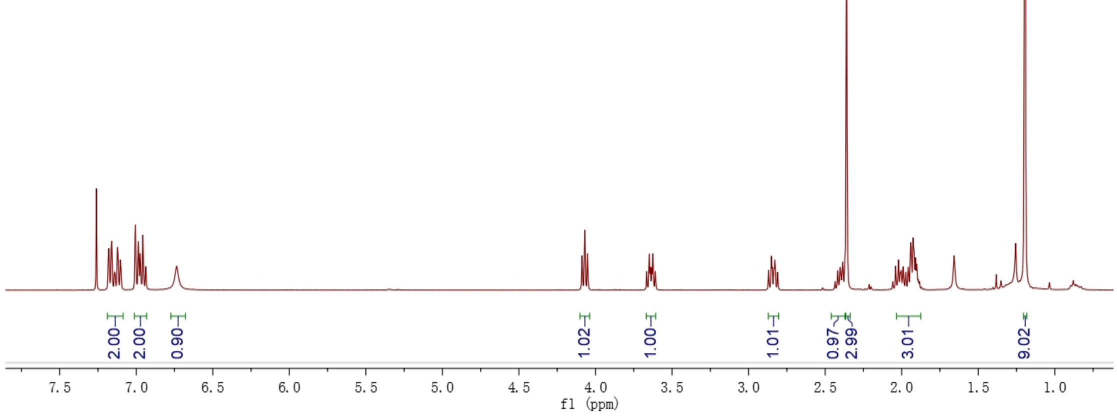

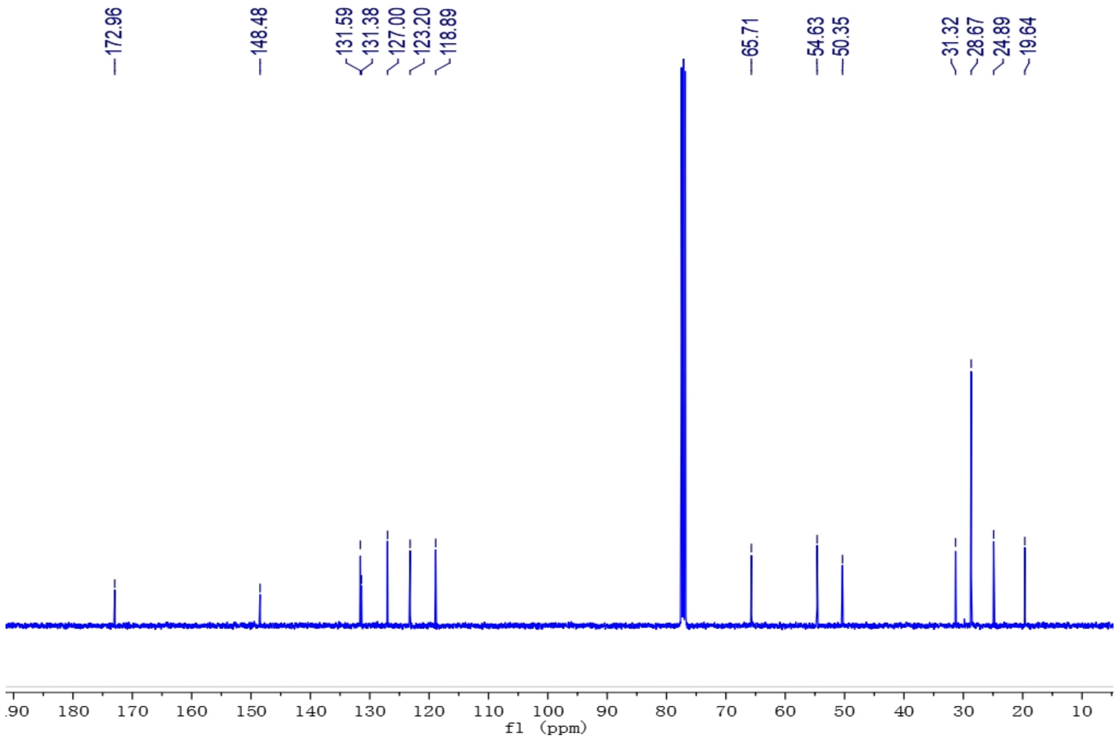

N-(tert-butyl)-1-(2-chlorophenyl)pyrrolidine-2-carboxamide

^{1}H NMR (400 MHz, CDCl$_3$) δ 7.37 (dd, *J* = 7.9, 1.3 Hz, 1H), 7.21 – 7.16 (m, 1H), 7.11 – 7.08 (m, 1H), 7.00 – 6.95 (m, 2H), 4.11 (dd, *J* = 8.0, 6.2 Hz, 1H), 3.88 (dt, *J* = 9.4, 6.3 Hz, 1H), 2.89 (dt, *J* = 9.3, 7.1 Hz, 1H), 2.38 (dt, *J* = 19.6, 7.3 Hz, 1H), 2.06 (td, *J* = 13.1, 6.9 Hz, 1H), 1.94 (dt, *J* = 13.3, 6.5 Hz, 2H), 1.22 (s, 9H). **^{13}C NMR (101 MHz, CDCl$_3$)** δ 172.77, 146.59, 130.74, 128.81, 127.89, 124.26, 121.40, 65.53, 54.39, 50.54, 31.48, 28.69, 25.10. **HRMS (ESI-TOF)**: m/z calculated for $C_{15}H_{22}ClON_2$ $[M+H]^+$: 281.1415. Found: 281.1416.

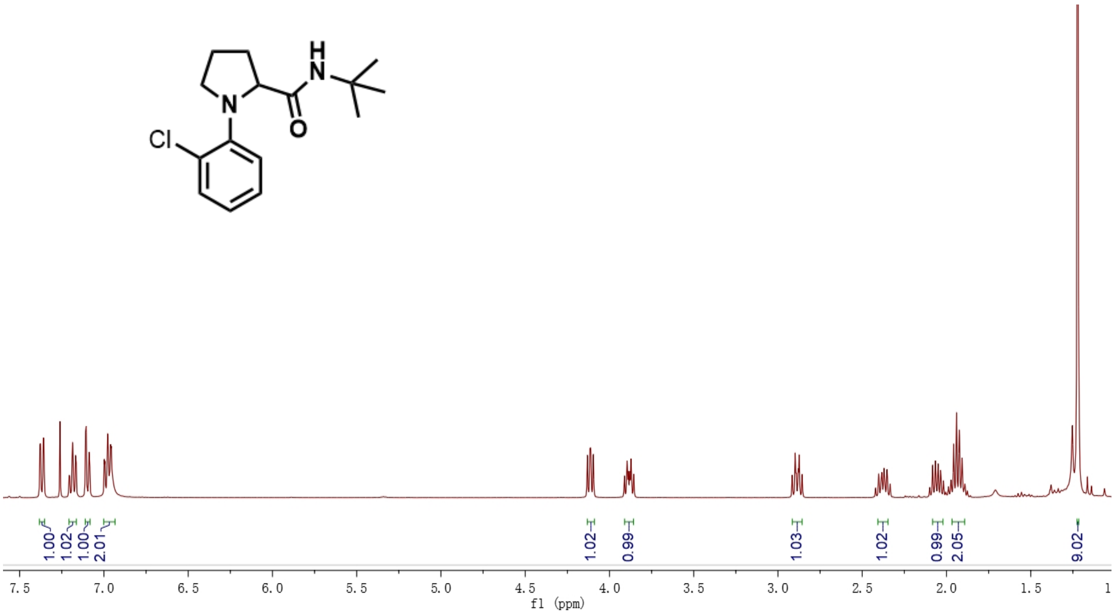

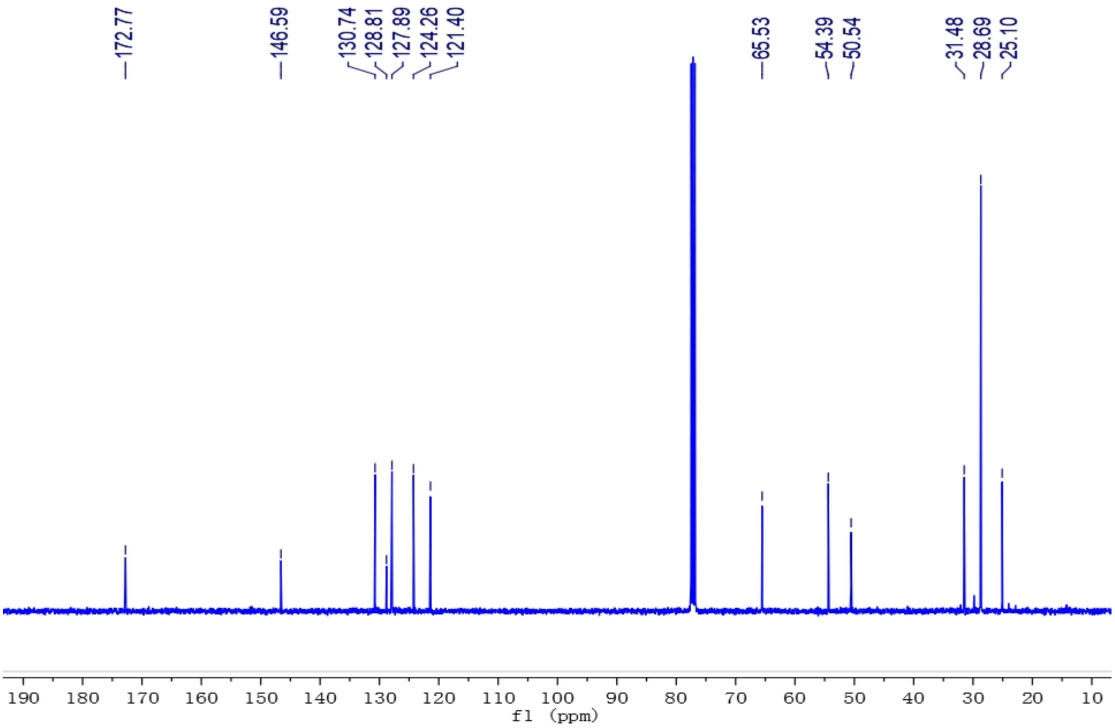

N-(tert-butyl)-1-(m-tolyl)pyrrolidine-2-carboxamide

^{1}H NMR (400 MHz, CDCl$_3$) δ 7.14 (dd, *J* = 10.6, 5.8 Hz, 1H), 6.64 (d, *J* = 7.4 Hz, 1H), 6.45 (d, *J* = 6.0 Hz, 2H), 6.34 (s, 1H), 3.82 (dd, *J* = 12.7, 6.3 Hz, 1H), 3.60 (d, *J* = 7.1 Hz, 1H), 3.19 (td, *J* = 9.7, 6.4 Hz, 1H), 2.31 (s, 3H), 2.26 – 2.16 (m, 2H), 2.06 – 1.87 (m, 2H), 1.29 (s, 9H). **^{13}C NMR (101 MHz, CDCl$_3$)** δ 173.32, 147.88, 139.17, 129.22, 119.16, 114.05, 110.53, 65.44, 50.83, 49.86, 31.49, 28.77, 24.34, 21.89. **HRMS (ESI-TOF)**: m/z calculated for $C_{16}H_{25}ON_2$ [M+H]$^+$: 261.1970. Found: 261.1970.

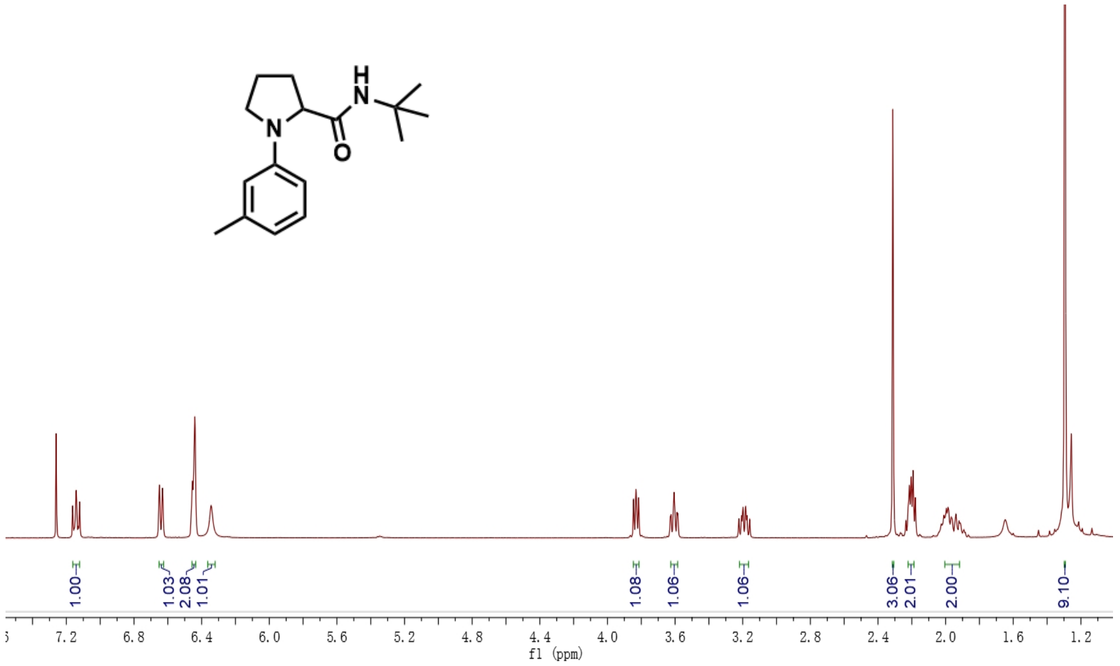

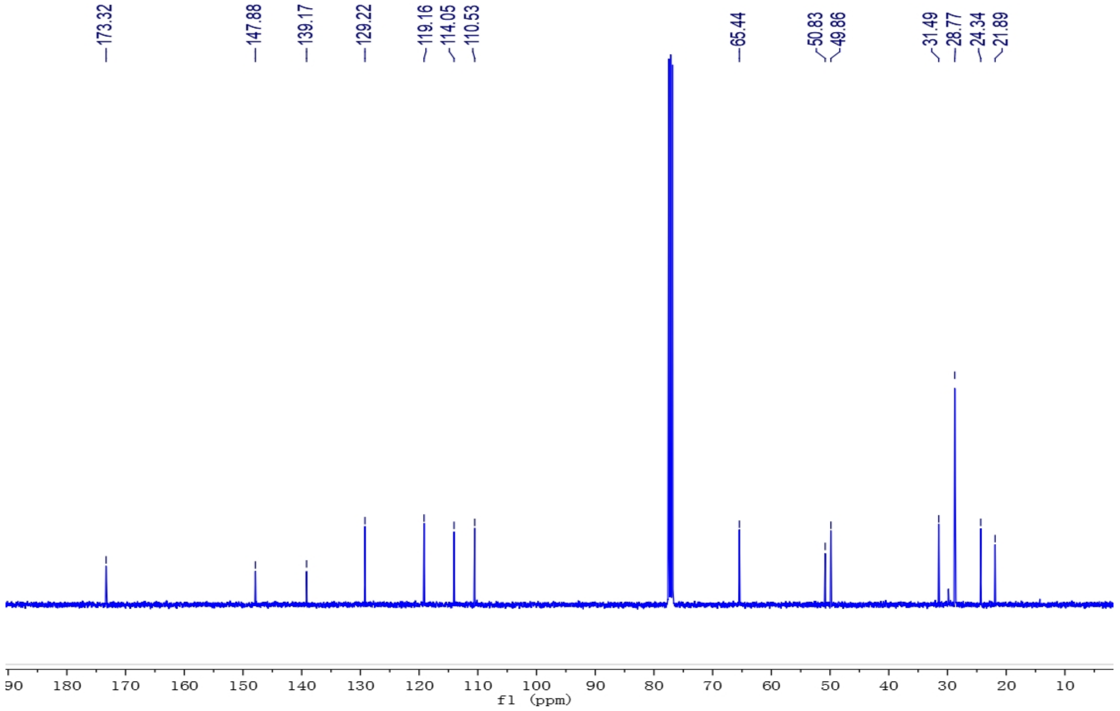

N-(tert-butyl)-1-(3-methoxyphenyl)pyrrolidine-2-carboxamide

^{1}H NMR (400 MHz, $CDCl_3$) δ 7.15 (t, *J* = 8.2 Hz, 1H), 6.40 – 6.36 (m, 1H), 6.29 (s, 1H), 6.23 (d, *J* = 8.2 Hz, 1H), 6.18 (s, 1H), 3.87 – 3.83 (m, 1H), 3.78 (s, 3H), 3.59 (t, *J* = 7.3 Hz, 1H), 3.21 (dt, *J* = 15.9, 8.0 Hz, 1H), 2.25 – 2.17 (m, 2H), 2.03 – 1.88 (m, 2H), 1.30 (s, 9H). **^{13}C NMR (101 MHz, $CDCl_3$)** δ 173.07, 160.82, 149.15, 130.11, 106.24, 103.51, 99.52, 65.39, 55.28, 50.86, 49.88, 31.49, 28.78, 24.30. **HRMS (ESI-TOF)**: m/z calculated for $C_{16}H_{25}O_2N_2$ $[M+H]^+$: 277.1911. Found: 277.1914.

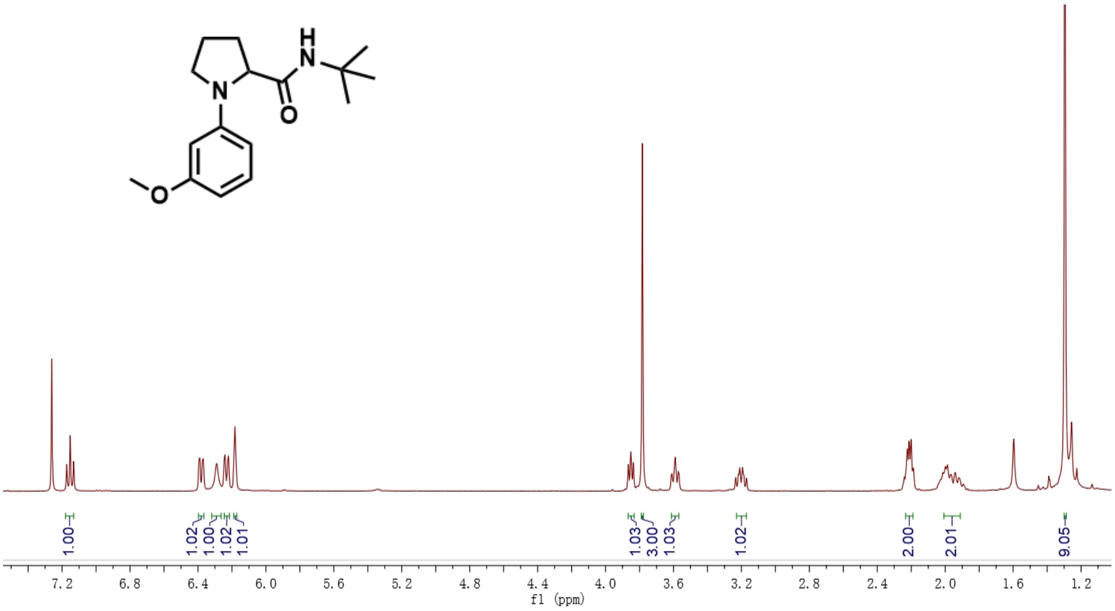

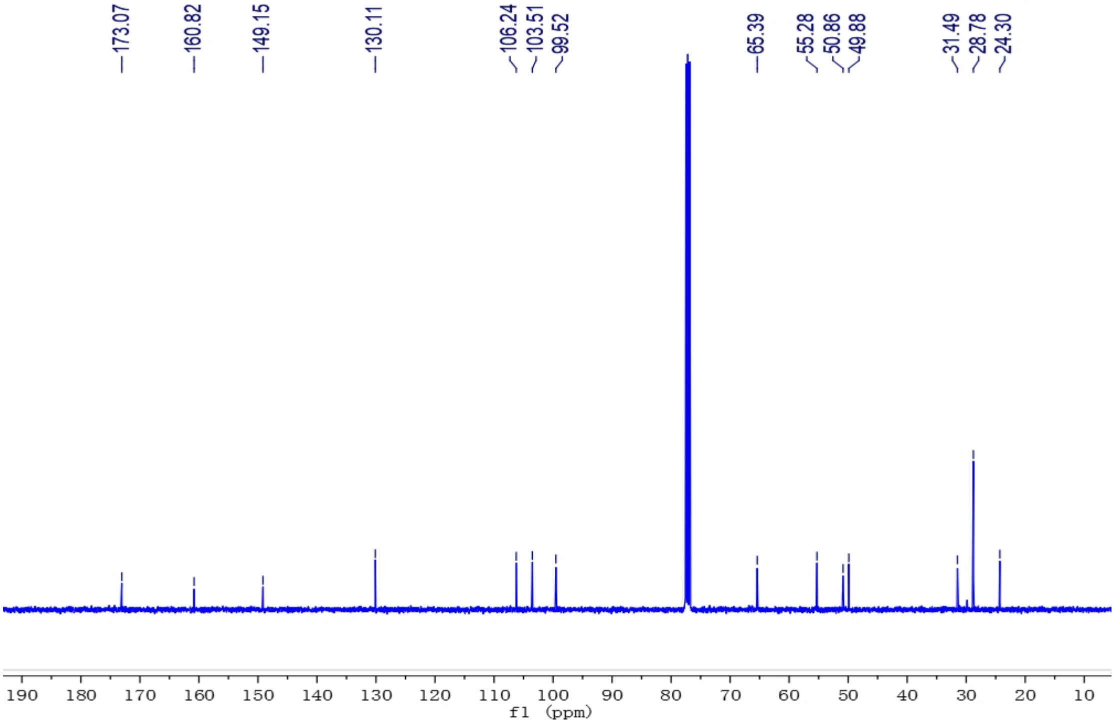

N-(tert-butyl)-1-(3-chlorophenyl)pyrrolidine-2-carboxamide

^{1}H NMR (400 MHz, $CDCl_3$) δ 7.14 (t, *J* = 8.1 Hz, 1H), 6.77 (dd, *J* = 7.9, 1.0 Hz, 1H), 6.61 (t, *J* = 2.0 Hz, 1H), 6.46 (dd, *J* = 8.3, 2.1 Hz, 1H), 6.16 (s, 1H), 3.84 (dd, *J* = 7.4, 4.6 Hz, 1H), 3.60 (td, *J* = 9.3, 4.8 Hz, 1H), 3.20 (td, *J* = 9.6, 6.5 Hz, 1H), 2.34 – 2.16 (m, 2H), 2.06 – 1.89 (m, 2H), 1.29 (s, 9H). **^{13}C NMR (101 MHz, $CDCl_3$)** δ 172.48, 148.66, 135.24, 130.34, 118.12, 113.17, 111.43, 65.19, 51.00, 49.81, 31.49, 28.76, 24.24. **HRMS (ESI-TOF)**: m/z calculated for $C_{15}H_{22}ClON_2$ $[M+H]^+$: 281.1415. Found: 281.1409.

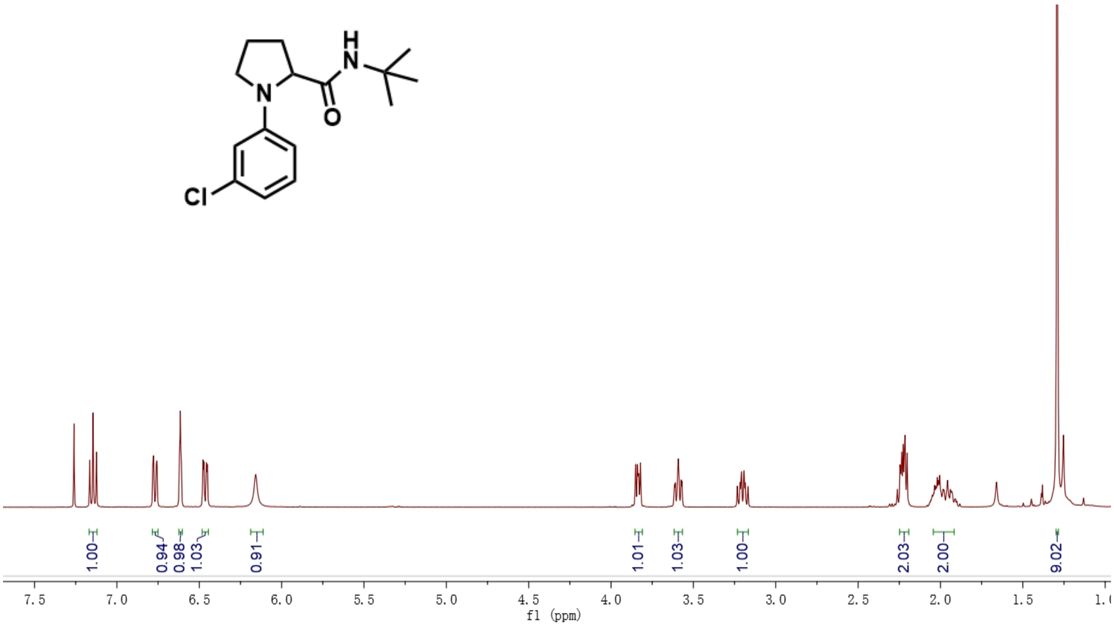

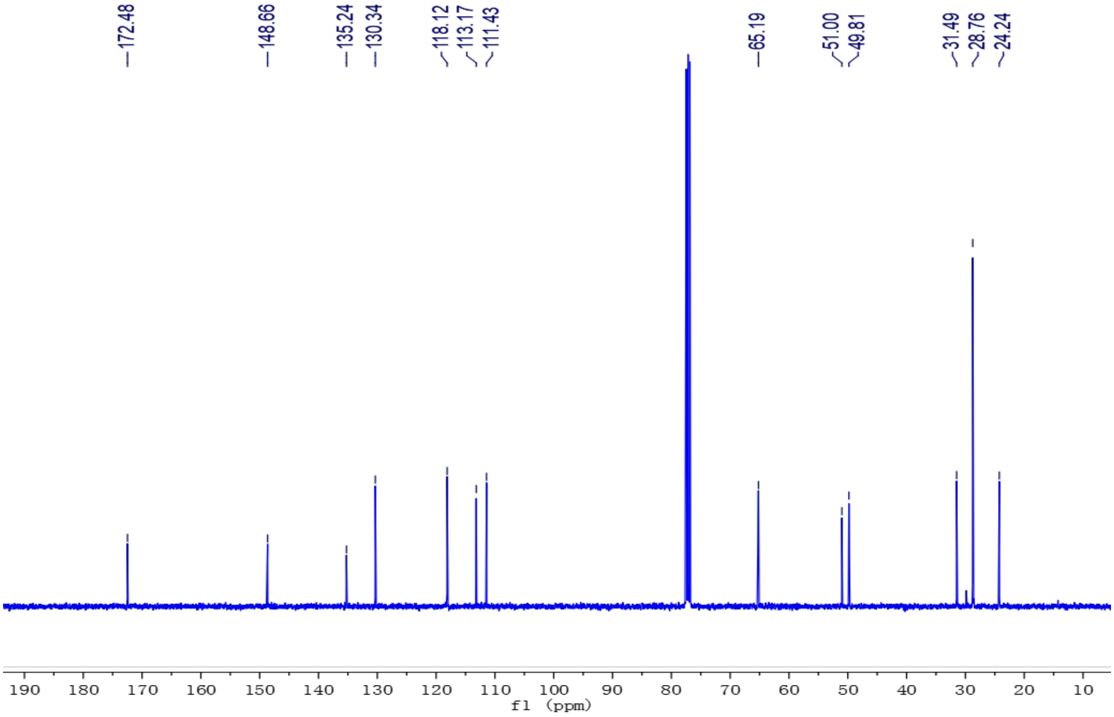

N-(tert-butyl)-1-(p-tolyl)pyrrolidine-2-carboxamide

^{1}H NMR (400 MHz, $CDCl_3$) δ 7.06 (d, *J* = 8.3 Hz, 2H), 6.55 (d, *J* = 8.5 Hz, 2H), 6.41 (s, 1H), 3.78 (dd, *J* = 8.1, 4.1 Hz, 1H), 3.64 – 3.57 (m, 1H), 3.19 – 3.11 (m, 1H), 2.27 (s, 3H), 2.23 – 2.16 (m, 2H), 2.01 – 1.87 (m, 2H), 1.30 (s, 9H). **^{13}C NMR (101 MHz, $CDCl_3$)** δ 173.32, 145.66, 129.73, 127.31, 113.20, 65.52, 50.65, 49.95, 31.38, 28.65, 24.26, 20.30. **HRMS (ESI-TOF)**: m/z calculated for $C_{16}H_{25}ON_2$ [M+H]$^+$: 261.1961. Found: 261.1958.

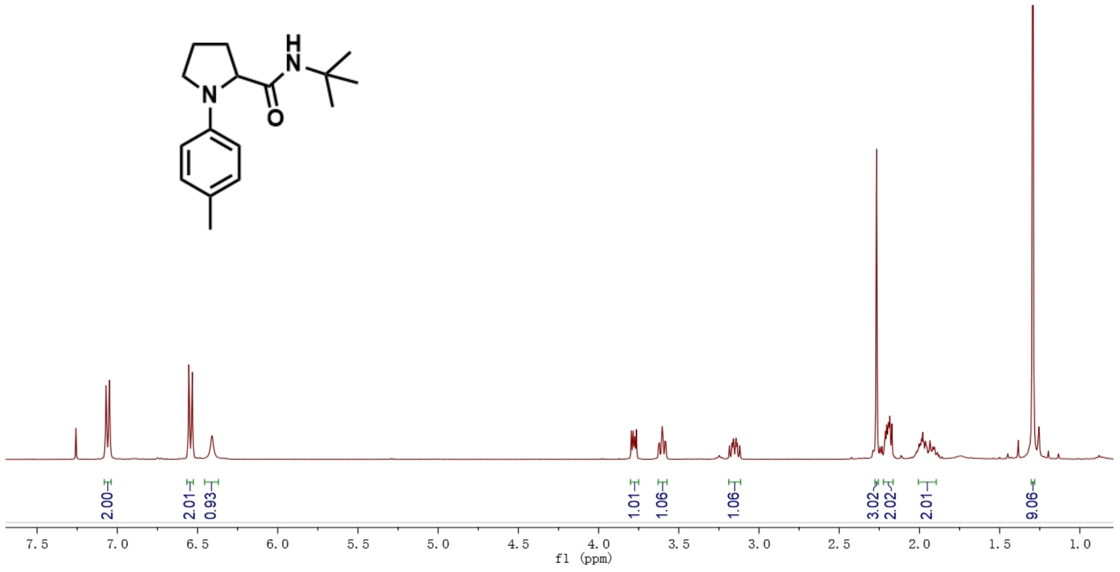

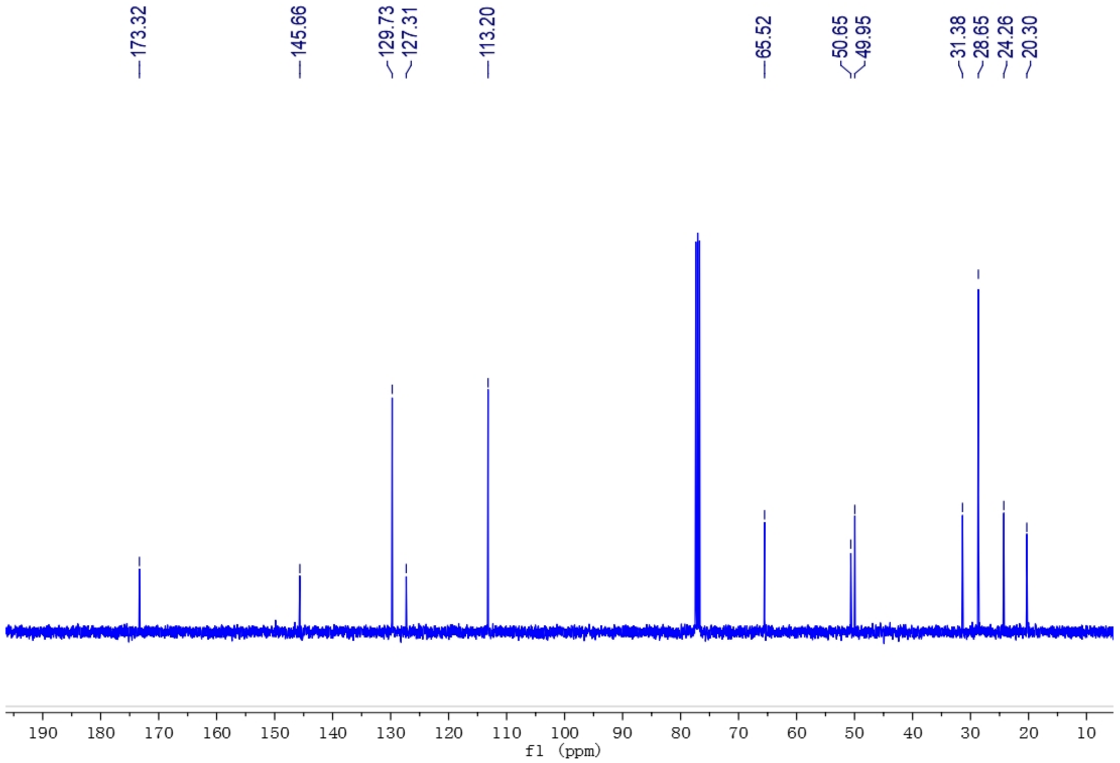

N-(tert-butyl)-1-(4-ethylphenyl)pyrrolidine-2-carboxamide

^{1}H NMR (400 MHz, $CDCl_3$) δ 7.09 (d, J = 8.5 Hz, 2H), 6.57 (d, J = 8.5 Hz, 2H), 6.42 (s, 1H), 3.81 – 3.77 (m, 1H), 3.60 (dd, J = 11.4, 4.4 Hz, 1H), 3.21 – 3.10 (m, 1H), 2.57 (q, J = 7.6 Hz, 2H), 2.25 – 2.14 (m, 2H), 2.04 – 1.89 (m, 2H), 1.30 (s, 9H), 1.21 (t, J = 7.6 Hz, 3H). **^{13}C NMR (101 MHz, $CDCl_3$)** δ 173.45, 146.02, 134.07, 128.68, 113.37, 65.73, 50.79, 50.08, 31.53, 28.79, 27.98, 24.40, 16.01. **HRMS (ESI-TOF)**: m/z calculated for $C_{17}H_{26}ON_2Na$ $[M+Na]^+$: 297.1937. Found: 297.1932.

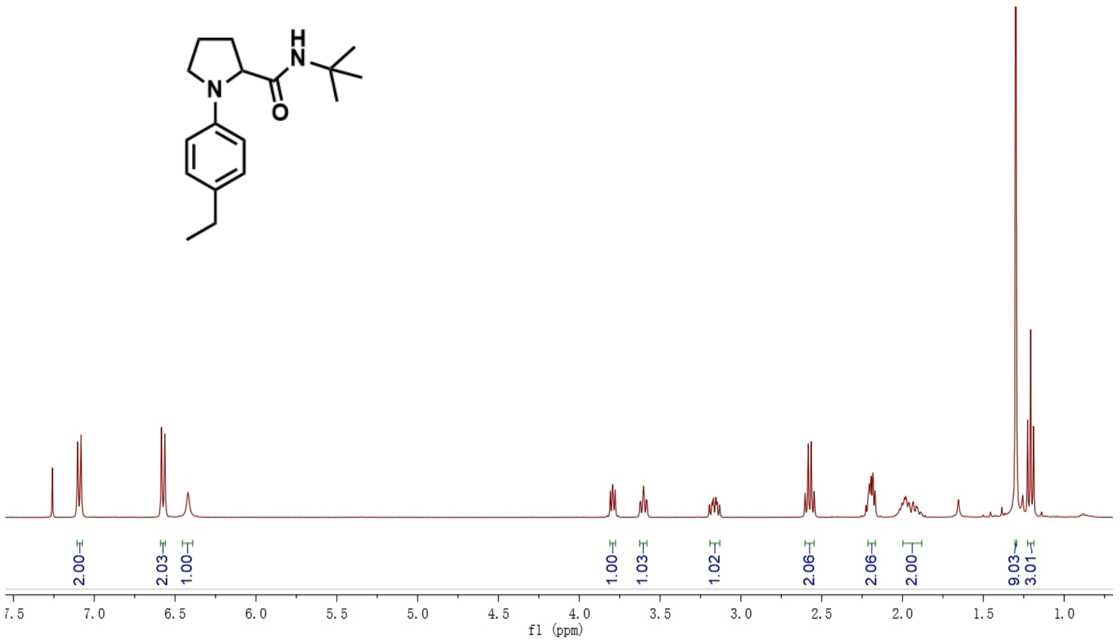

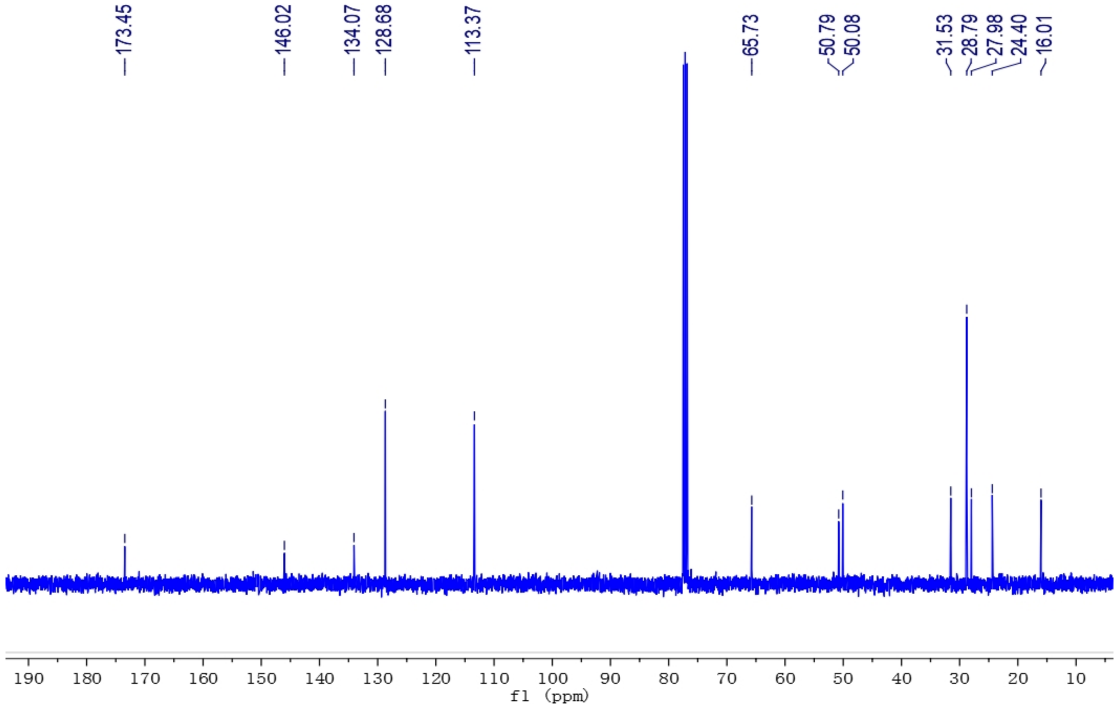

N-(tert-butyl)-1-(4-fluorophenyl)pyrrolidine-2-carboxamide

^{1}H NMR (400 MHz, CDCl$_3$) δ 6.95 (t, *J* = 8.7 Hz, 2H), 6.56 – 6.49 (m, 2H), 6.33 (s, 1H), 3.76 (dd, *J* = 9.1, 3.0 Hz, 1H), 3.66 – 3.54 (m, 1H), 3.15 (td, *J* = 9.4, 6.5 Hz, 1H), 2.30 – 2.15 (m, 2H), 2.07 – 1.89 (m, 2H), 1.29 (s, 9H). **^{13}C NMR (101 MHz, CDCl$_3$)** δ 173.04, 144.30, 115.92, 115.70, 113.96, 113.88, 65.77, 50.84, 50.36, 31.60, 28.77, 24.45. **HRMS (ESI-TOF)**: m/z calculated for $C_{15}H_{22}FON_2$ [M+H]$^+$: 265.1711. Found: 265.1708.

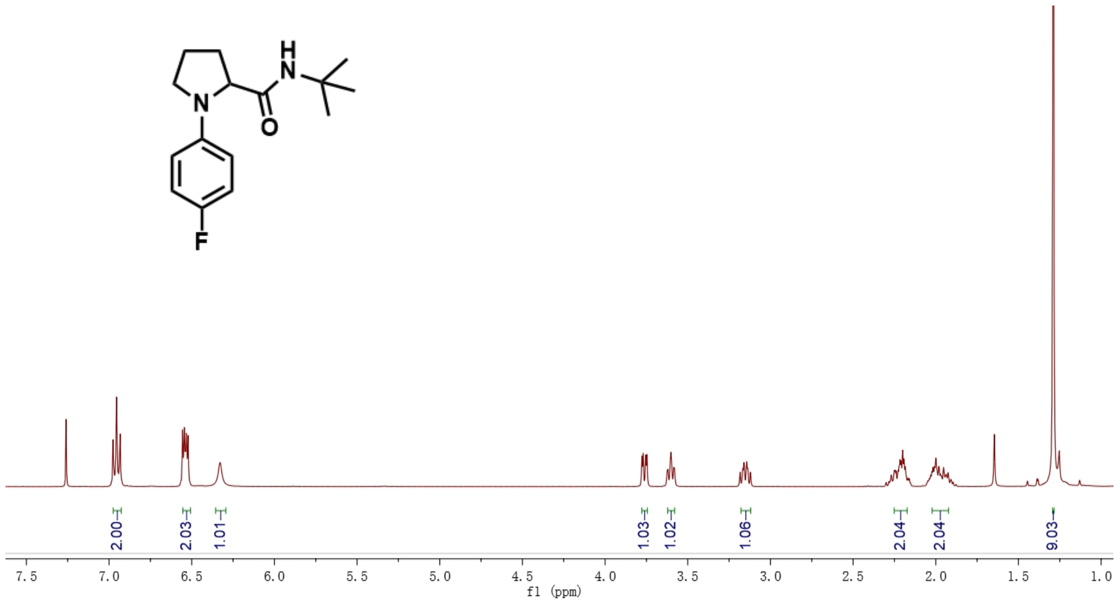

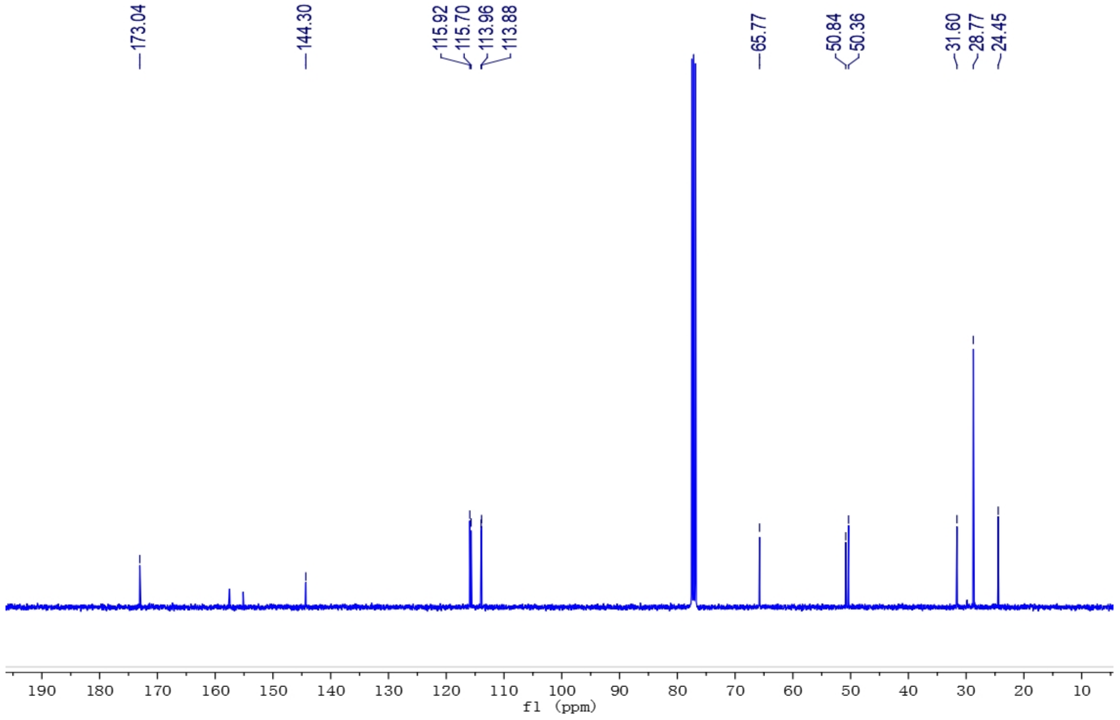

N-(tert-butyl)-1-(4-chlorophenyl)pyrrolidine-2-carboxamide

^{1}H NMR (400 MHz, $CDCl_3$) δ 7.19 (d, *J* = 8.9 Hz, 2H), 6.53 (d, *J* = 8.9 Hz, 2H), 6.21 (s, 1H), 3.80 (dd, *J* = 8.8, 3.3 Hz, 1H), 3.64 – 3.57 (m, 1H), 3.18 (td, *J* = 9.5, 6.5 Hz, 1H), 2.29 – 2.16 (m, 2H), 2.05 – 1.90 (m, 2H), 1.29 (s, 9H). **^{13}C NMR (101 MHz, $CDCl_3$)** δ 172.72, 146.24, 129.21, 123.19, 114.31, 65.39, 50.94, 50.00, 31.55, 28.76, 24.36. **HRMS (ESI-TOF)**: m/z calculated for $C_{15}H_{22}ClON_2$ [M+H]$^+$: 281.1415. Found: 281.1414.

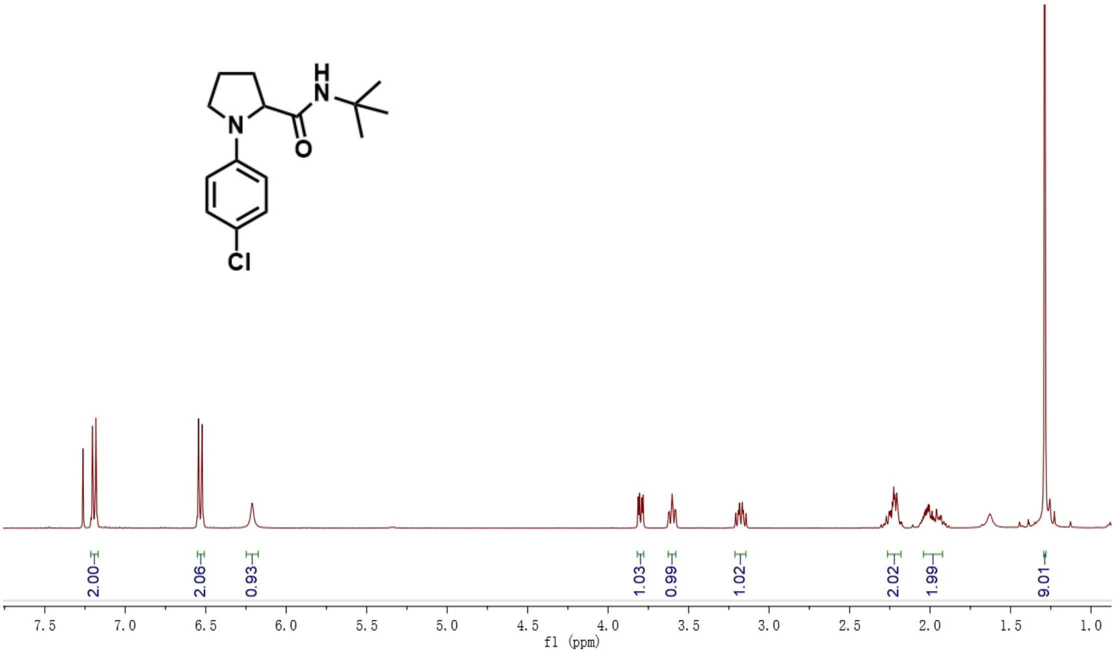

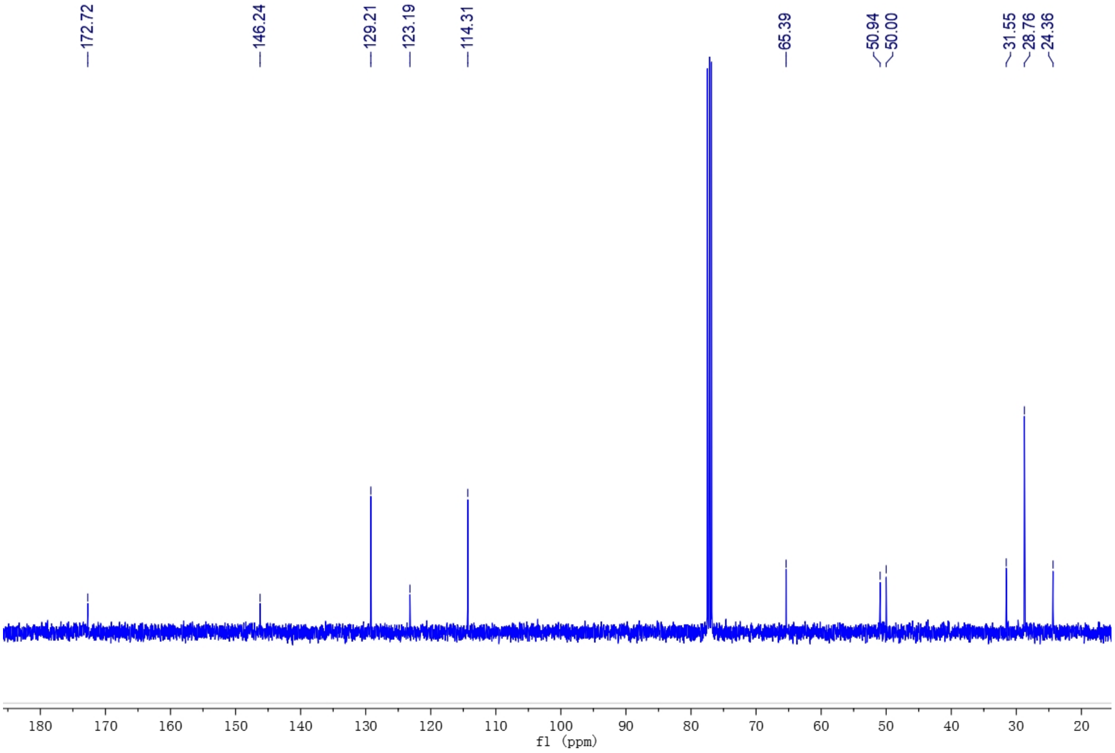

1-(4-bromophenyl)-N-(tert-butyl)pyrrolidine-2-carboxamide

^{1}H NMR (400 MHz, $CDCl_3$) δ 7.35 – 7.30 (m, 2H), 6.52 – 6.44 (m, 2H), 6.19 (s, 1H), 3.79 (dd, *J* = 8.7, 3.4 Hz, 1H), 3.63 – 3.55 (m, 1H), 3.17 (td, *J* = 9.6, 6.4 Hz, 1H), 2.29 – 2.17 (m, 2H), 2.06 – 1.90 (m, 2H), 1.29 (s, 9H). **^{13}C NMR (101 MHz, $CDCl_3$)** δ 172.65, 146.62, 132.09, 114.82, 110.36, 65.33, 50.96, 49.93, 31.55, 28.76, 24.34. **HRMS (ESI-TOF)**: m/z calculated for $C_{15}H_{22}BrON_2$ $[M+H]^+$: 325.0910. Found: 325.0904.

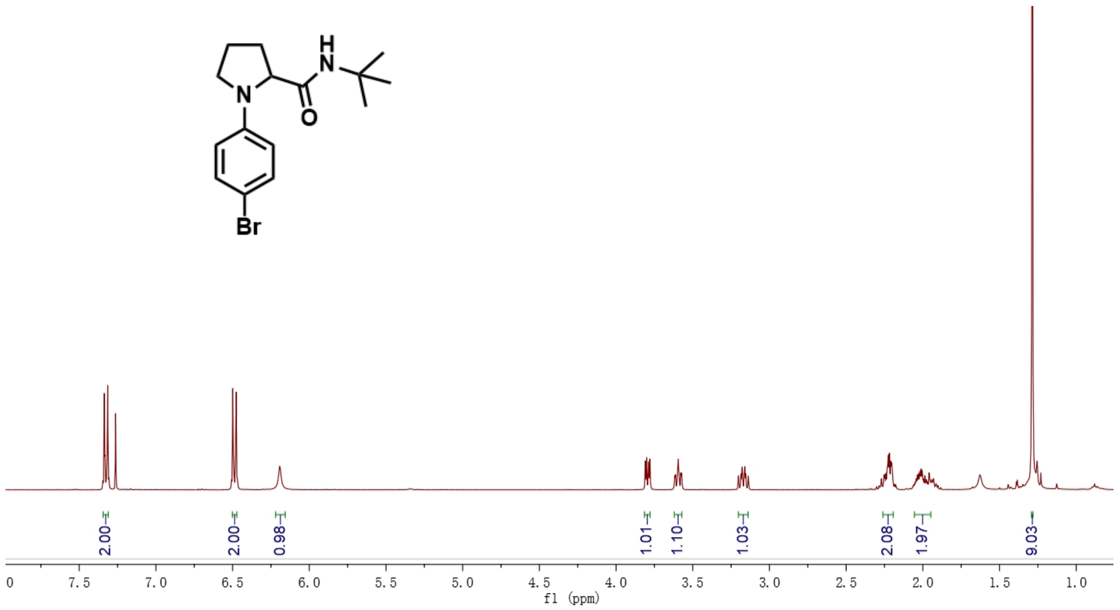

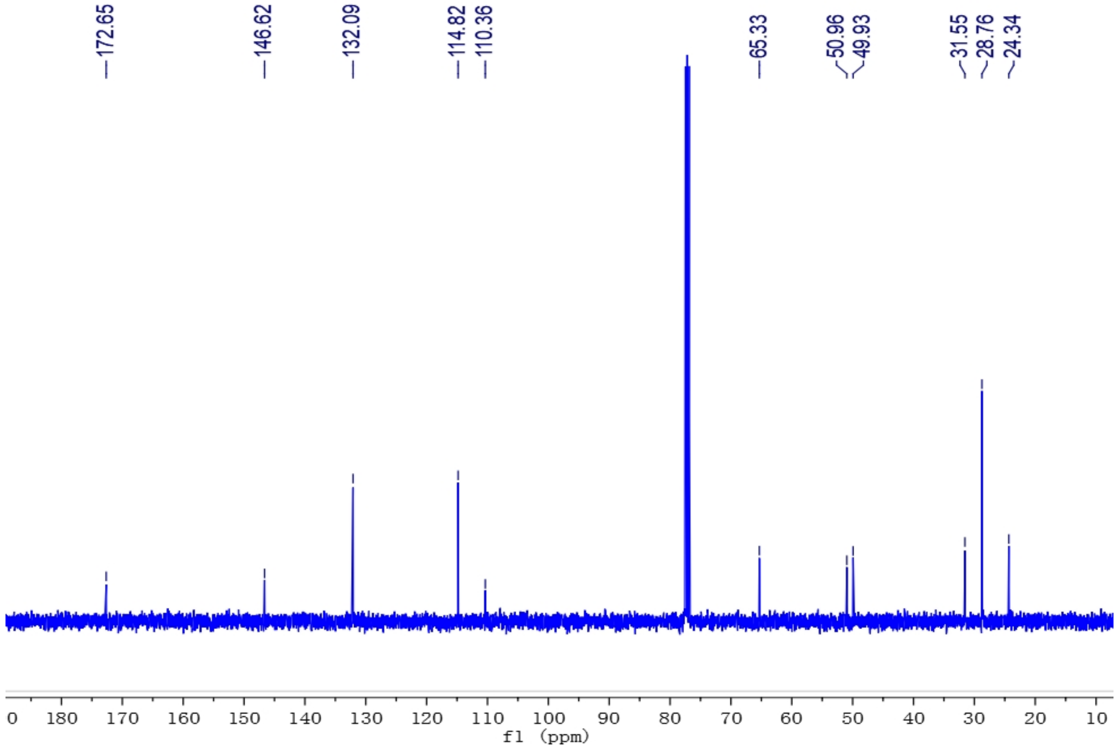

N-(tert-butyl)-1-(4-(trifluoromethyl)phenyl)pyrrolidine-2-carboxamide

^{1}H NMR (400 MHz, CDCl$_3$) δ 7.47 (d, *J* = 8.6 Hz, 2H), 6.63 (d, *J* = 8.6 Hz, 2H), 6.06 (s, 1H), 3.90 (dd, *J* = 12.8, 6.3 Hz, 1H), 3.70 – 3.61 (m, 1H), 3.26 (td, *J* = 9.6, 6.7 Hz, 1H), 2.27 – 2.21 (m, 2H), 2.09 – 1.95 (m, 2H), 1.29 (s, 9H). **^{13}C NMR (101 MHz, CDCl$_3$)** δ 172.20, 149.75, 126.69, 112.60, 65.05, 51.09, 49.69, 31.52, 28.73, 24.22. **HRMS (ESI-TOF)**: m/z calculated for $C_{16}H_{22}F_3ON_2$ $[M+H]^+$: 315.1679. Found: 315.1674.

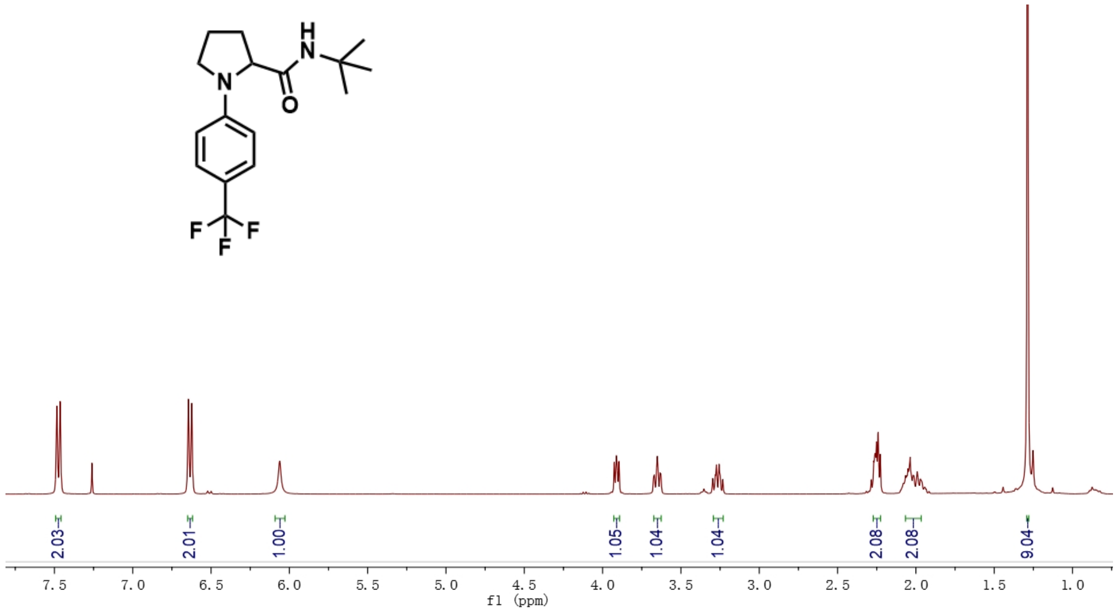

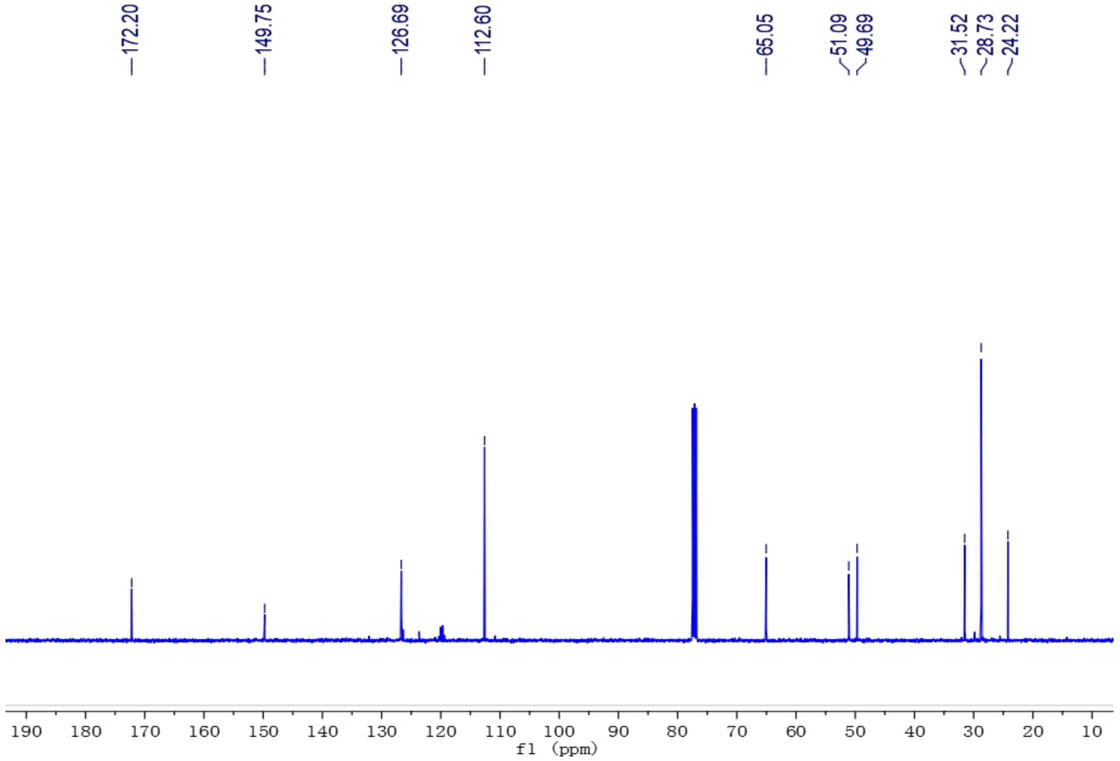

N-(tert-butyl)-1-(3,4-dichlorophenyl)pyrrolidine-2-carboxamide

^{1}H NMR (400 MHz, CDCl$_3$) δ 7.28 (d, J = 1.9 Hz, 1H), 6.71 (d, J = 2.8 Hz, 1H), 6.44 (dd, J = 8.9, 2.8 Hz, 1H), 6.10 (s, 1H), 3.82 (dd, J = 8.6, 3.4 Hz, 1H), 3.63 – 3.57 (m, 1H), 3.20 (td, J = 9.5, 6.6 Hz, 1H), 2.31 – 2.18 (m, 2H), 2.09 – 1.96 (m, 2H), 1.31 (s, 9H). **^{13}C NMR (101 MHz, CDCl$_3$)** δ 172.12, 146.96, 133.10, 130.75, 121.12, 114.60, 112.71, 65.18, 51.09, 49.94, 31.55, 28.75, 24.28. **HRMS (ESI-TOF)**: m/z calculated for $C_{15}H_{20}Cl_2ON_2K$ [M+K]$^+$: 353.0584. Found: 353.0578.

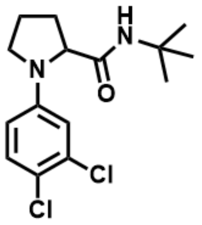

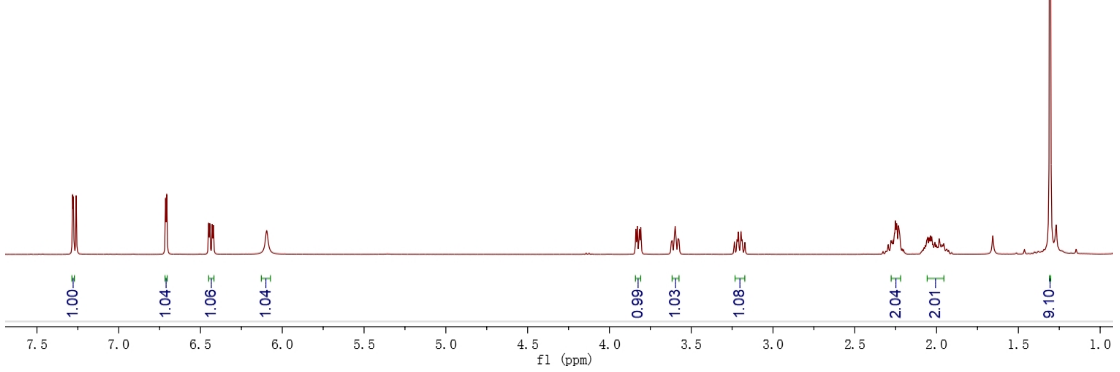

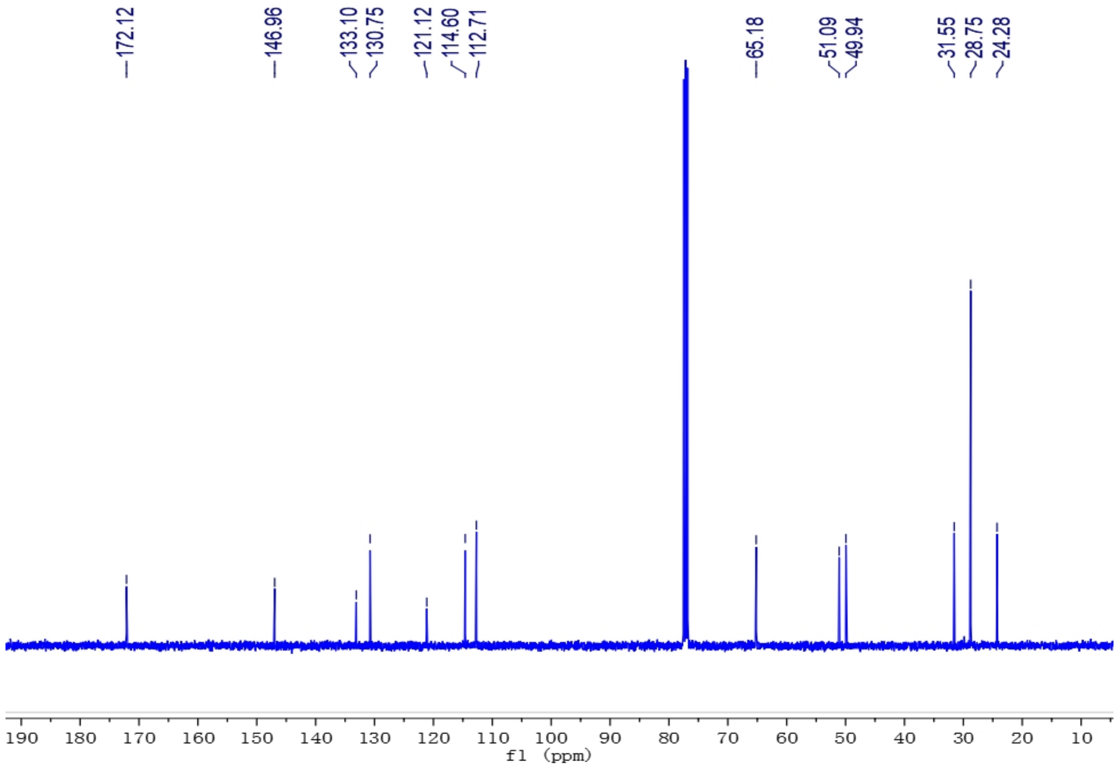

N-(tert-butyl)-1-mesitylpyrrolidine-2-carboxamide

^{1}H NMR (400 MHz, CDCl$_3$) δ 7.07 (s, 1H), 6.85 (s, 2H), 3.94 (dd, J = 9.5, 2.0 Hz, 1H), 3.40 (t, J = 7.3 Hz, 1H), 3.08 – 2.93 (m, 1H), 2.48 – 2.37 (m, 1H), 2.32 (s, 6H), 2.24 (s, 3H), 2.19 – 2.11 (m, 1H), 2.02 – 1.83 (m, 2H), 1.32 (s, 9H). **^{13}C NMR (101 MHz, CDCl$_3$)** δ 175.23, 142.93, 136.09, 135.27, 130.45, 66.40, 54.22, 50.65, 32.30, 28.94, 25.65, 20.74, 19.62. **HRMS (ESI-TOF)**: m/z calculated for $C_{18}H_{28}ON_2K$ [M+K]$^+$: 327.1833. Found: 327.1837.

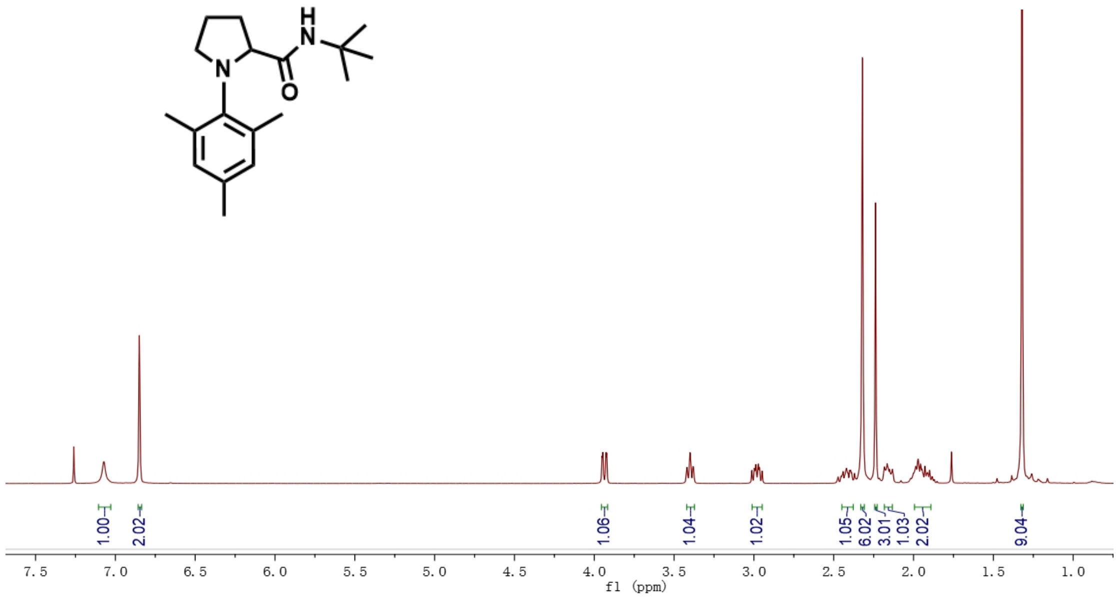

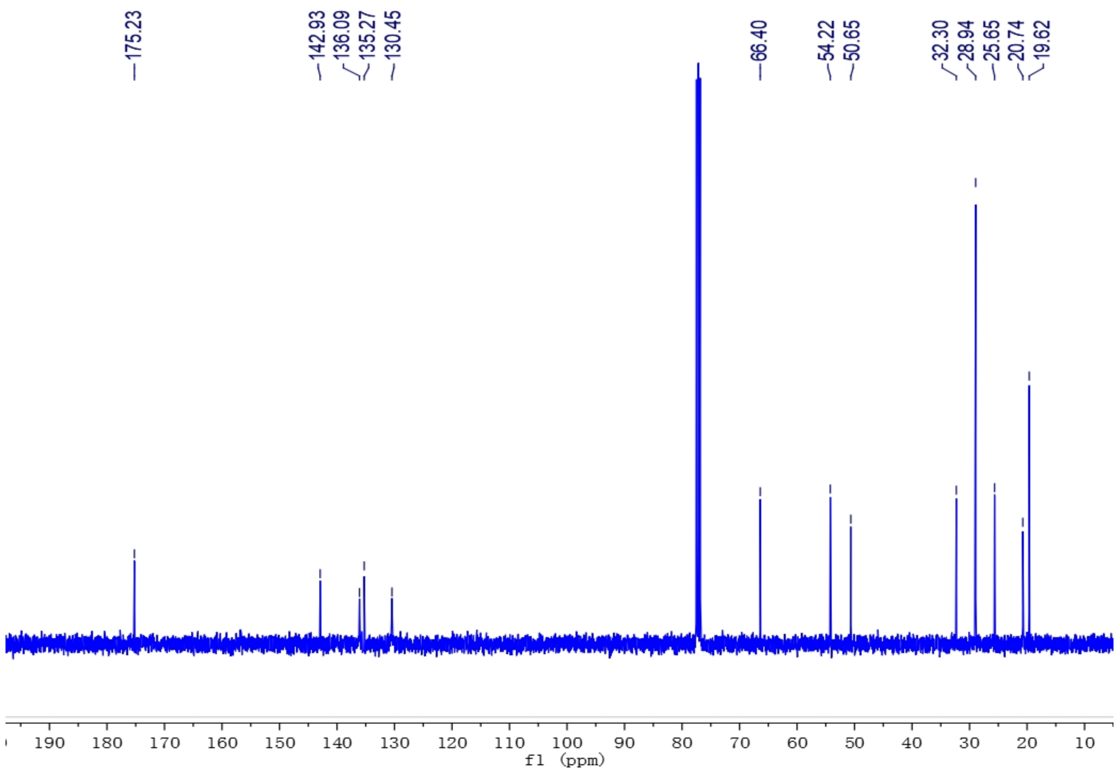

1-(2-bromo-4-methylphenyl)-N-(tert-butyl)pyrrolidine-2-carboxamide

^{1}H NMR (400 MHz, CDCl$_3$) δ 7.39 (s, 1H), 7.23 (s, 1H), 7.04 (s, 2H), 4.05 (dd, *J* = 8.6, 5.5 Hz, 1H), 3.83 (dt, *J* = 9.4, 5.9 Hz, 1H), 2.72 (dd, *J* = 16.7, 7.5 Hz, 1H), 2.35 (ddd, *J* = 20.2, 11.7, 6.3 Hz, 1H), 2.28 (s, 3H), 2.07 (td, *J* = 12.5, 6.5 Hz, 1H), 1.95 – 1.86 (m, 2H), 1.25 (s, 9H). **^{13}C NMR (101 MHz, CDCl$_3$)** δ 173.00, 145.74, 135.58, 133.97, 129.42, 122.46, 120.79, 65.92, 55.30, 50.61, 31.57, 28.81, 25.04, 20.53. **HRMS (ESI-TOF)**: m/z calculated for $C_{16}H_{24}BrON_2$ [M+H]$^+$: 339.1067. Found: 339.1069.

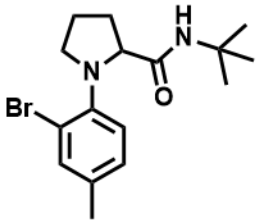

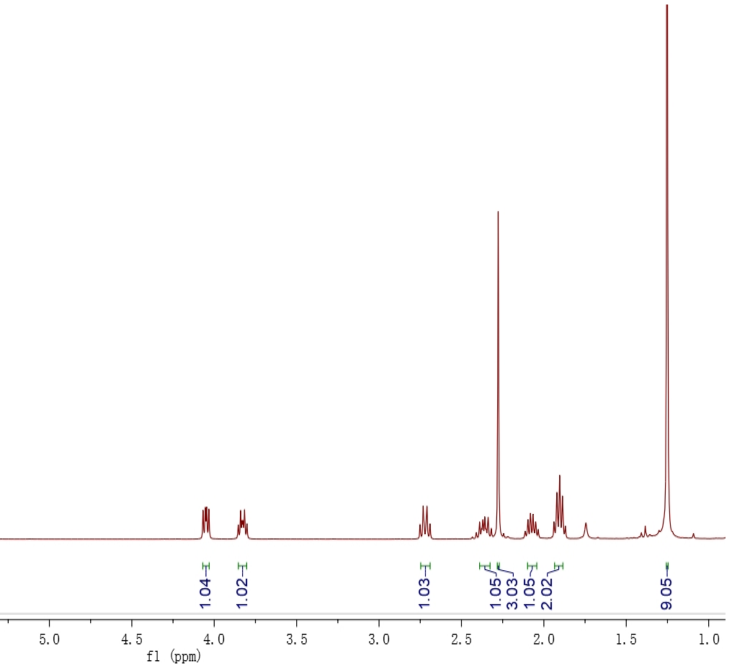

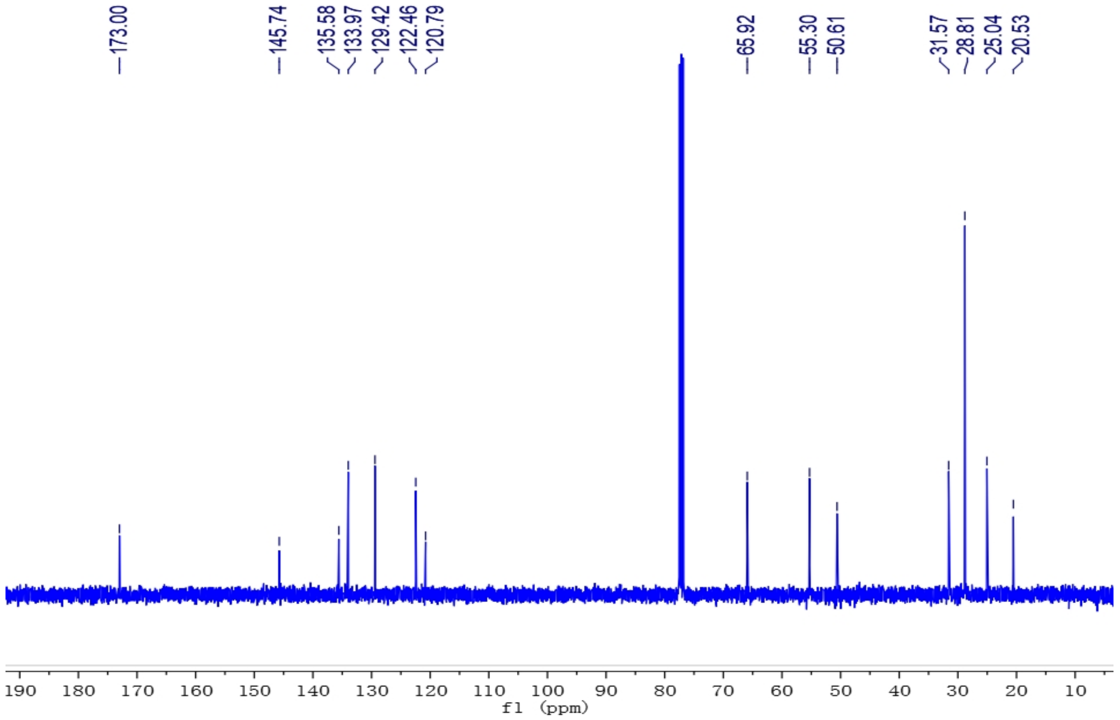

N-(tert-butyl)-2-(methyl(phenyl)amino)acetamide

^{1}H NMR (400 MHz, $CDCl_3$) δ 7.27 (ddd, *J* = 6.4, 4.8, 1.5 Hz, 2H), 6.84 (t, *J* = 7.3 Hz, 1H), 6.74 (d, *J* = 8.0 Hz, 2H), 6.38 (s, 1H), 3.72 (s, 2H), 2.98 (s, 3H), 1.33 (s, 9H). **^{13}C NMR (101 MHz, $CDCl_3$)** δ 169.61, 149.61, 129.39, 118.80, 113.47, 59.96, 51.01, 39.86, 28.80. **HRMS (ESI-TOF)**: m/z calculated for $C_{13}H_{20}ON_2K$ $[M+K]^+$: 259.1207. Found: 259.1208.

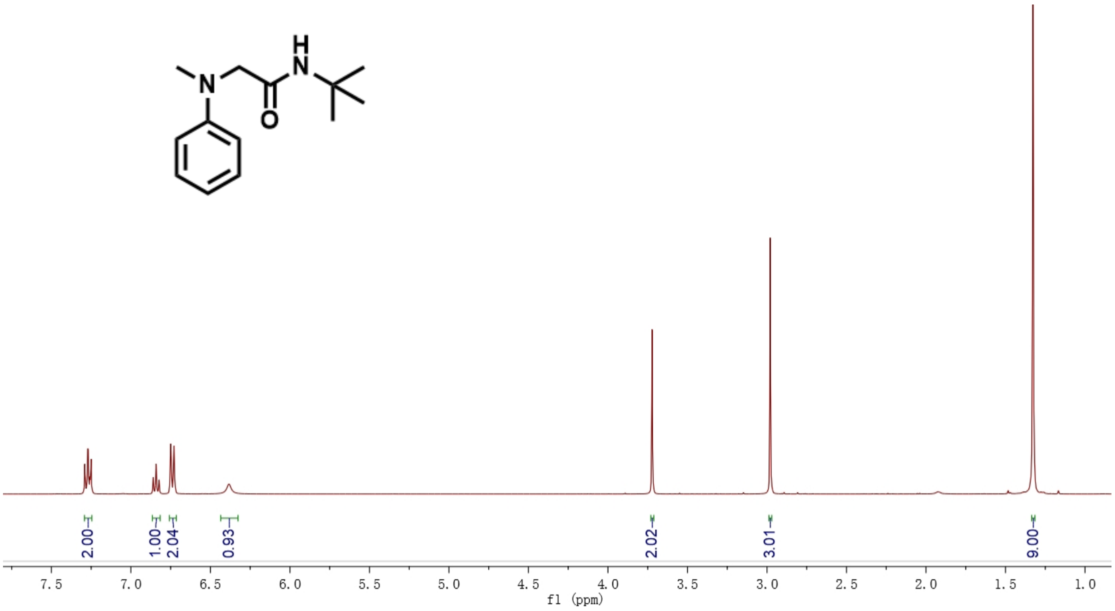

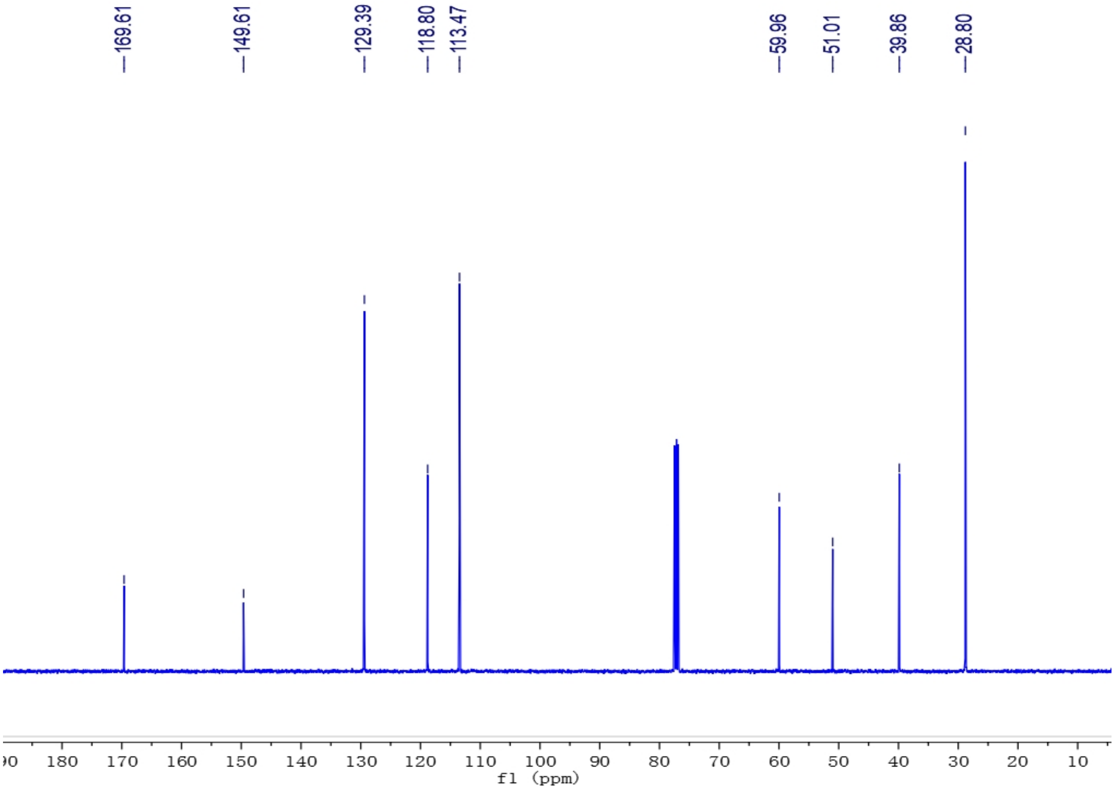

N-(tert-butyl)-1-phenylpiperidine-2-carboxamide

^{1}H NMR (400 MHz, CDCl$_3$) δ 7.27 (dd, *J* = 8.6, 7.4 Hz, 2H), 6.97 (d, *J* = 7.9 Hz, 2H), 6.91 (t, *J* = 7.3 Hz, 1H), 6.32 – 6.12 (m, 1H), 3.87 – 3.83 (m, 1H), 3.31 – 3.24 (m, 1H), 3.18 – 3.11 (m, 1H), 1.99 – 1.87 (m, 2H), 1.72 – 1.63 (m, 3H), 1.57 – 1.47 (m, 1H), 1.17 (s, 9H). **^{13}C NMR (101 MHz, CDCl$_3$)** δ 172.26, 151.36, 129.40, 121.00, 118.38, 63.00, 50.63, 50.32, 28.63, 27.45, 24.69, 22.06. **HRMS (ESI-TOF)**: m/z calculated for $C_{16}H_{24}ON_2Na$ $[M+Na]^+$: 283.1781. Found: 283.1782.

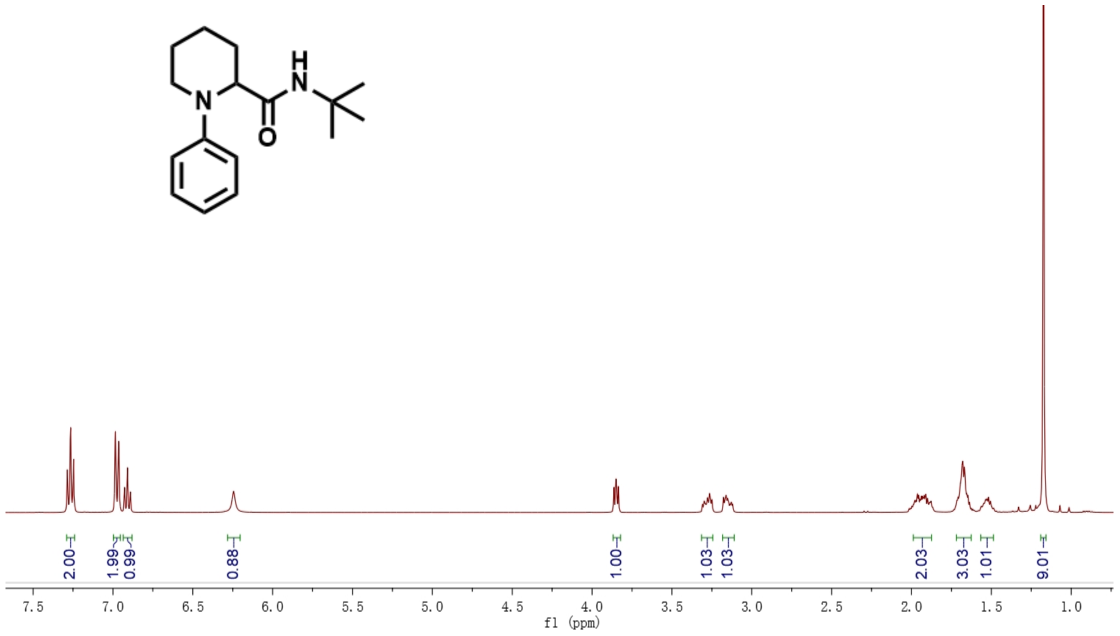

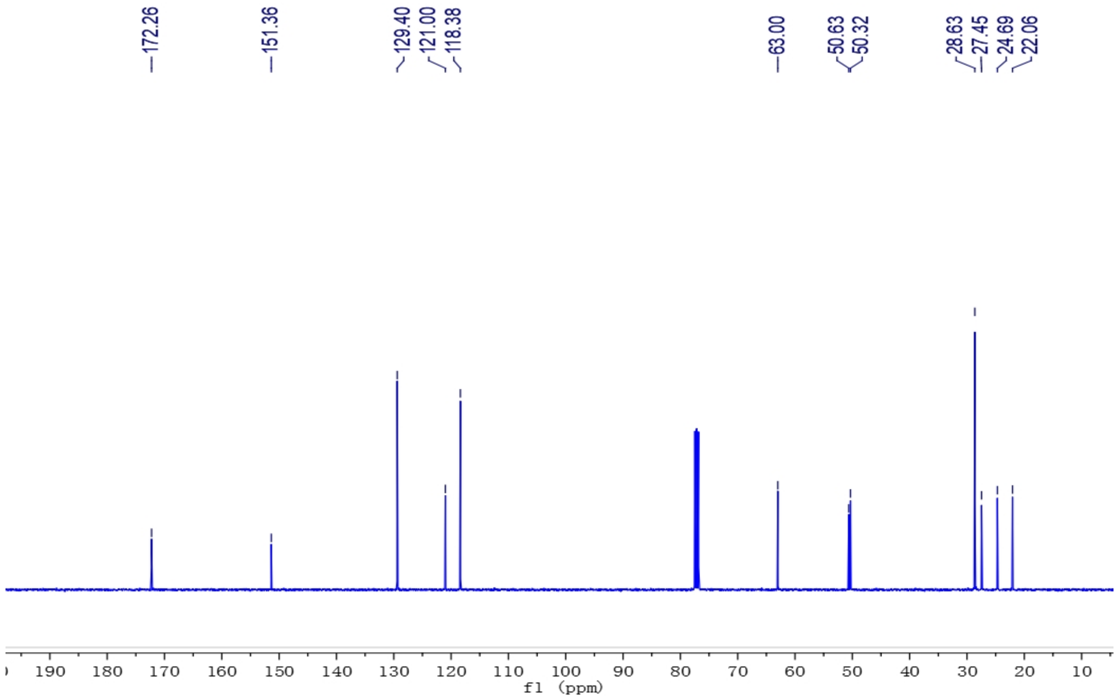

N-(tert-butyl)-4-phenylmorpholine-3-carboxamide

^{1}H NMR (400 MHz, $CDCl_3$) δ 7.25 – 7.19 (m, 2H), 6.93 – 6.89 (m, 3H), 5.97 (s, 1H), 3.98 – 3.91 (m, 1H), 3.92 – 3.82 (m, 1H), 3.81 – 3.76 (m, 3H), 3.33 – 3.24 (m, 1H), 3.13 – 3.01 (m, 1H), 1.06 (s, 9H). **^{13}C NMR (101 MHz, $CDCl_3$)** δ 169.26, 150.14, 129.60, 122.05, 118.24, 68.50, 66.82, 62.28, 50.99, 49.87, 28.57. **HRMS (ESI-TOF)**: m/z calculated for $C_{15}H_{23}O_2N_2$ [M+H]$^{+}$: 263.1754. Found: 263.1758.

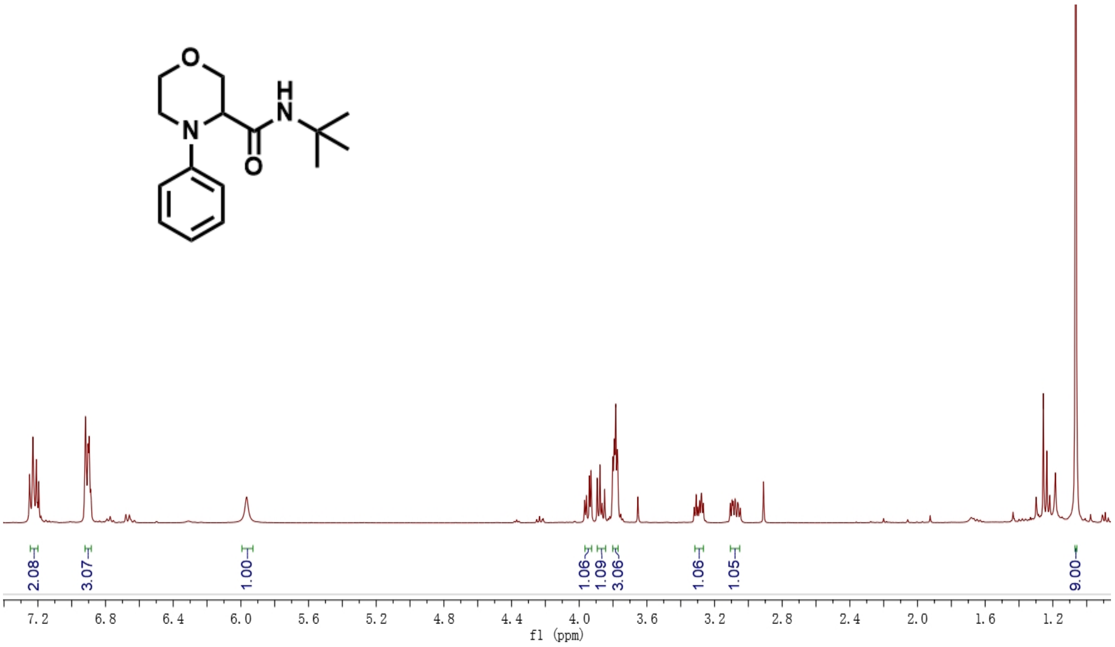

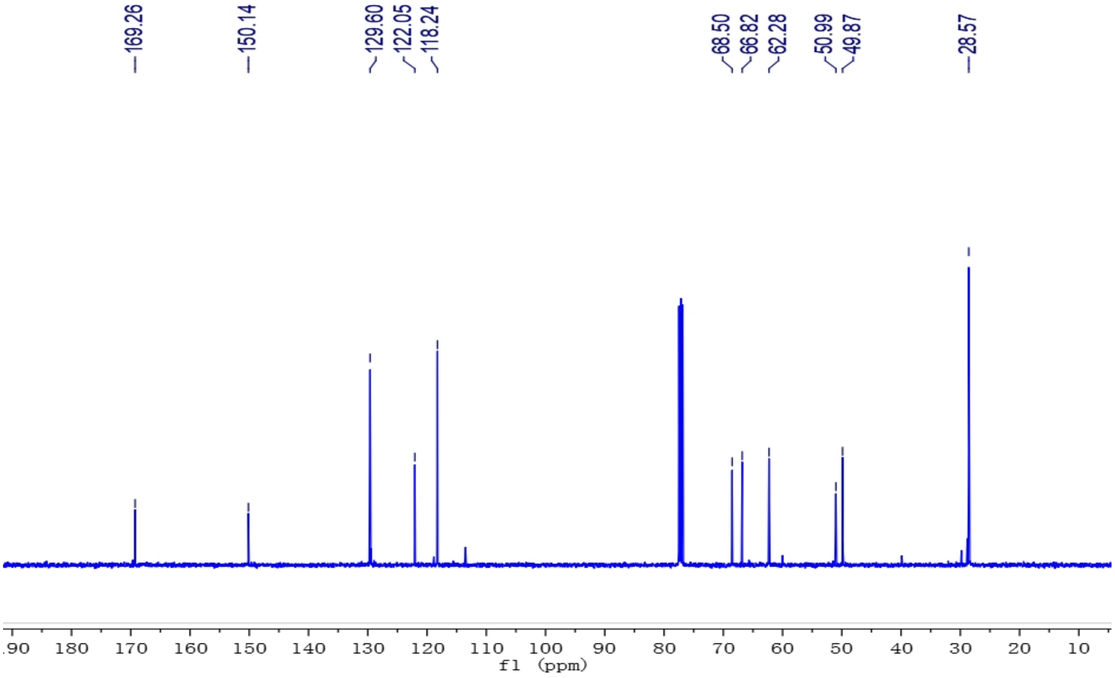

N-(tert-butyl)-1-phenylazepane-2-carboxamide

^{1}H NMR (400 MHz, CDCl$_3$) δ 7.24 (dd, *J* = 8.6, 7.3 Hz, 2H), 6.76 (t, *J* = 7.3 Hz, 1H), 6.69 (d, *J* = 8.3 Hz, 2H), 6.14 (s, 1H), 3.81 (dd, *J* = 11.7, 5.1 Hz, 1H), 3.72 (dd, *J* = 15.5, 4.7 Hz, 1H), 3.36 (dd, *J* = 15.5, 10.0 Hz, 1H), 2.59 – 2.49 (m, 1H), 1.98 – 1.85 (m, 1H), 1.84 – 1.67 (m, 4H), 1.43 – 1.31 (m, 2H), 1.28 (s, 9H). **^{13}C NMR (101 MHz, CDCl$_3$)** δ 172.95, 148.59, 129.40, 117.40, 112.50, 66.46, 50.74, 45.43, 32.22, 29.22, 28.70, 27.91, 26.78. **HRMS (ESI-TOF)**: m/z calculated for $C_{17}H_{27}ON_2$ $[M+H]^+$: 275.2118. Found: 275.2114.

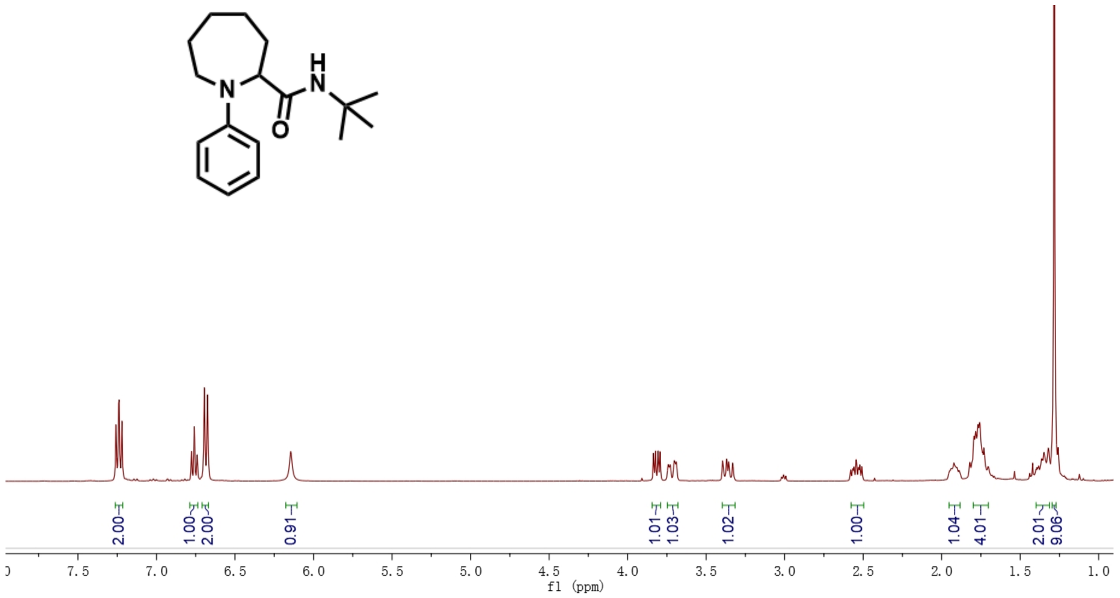

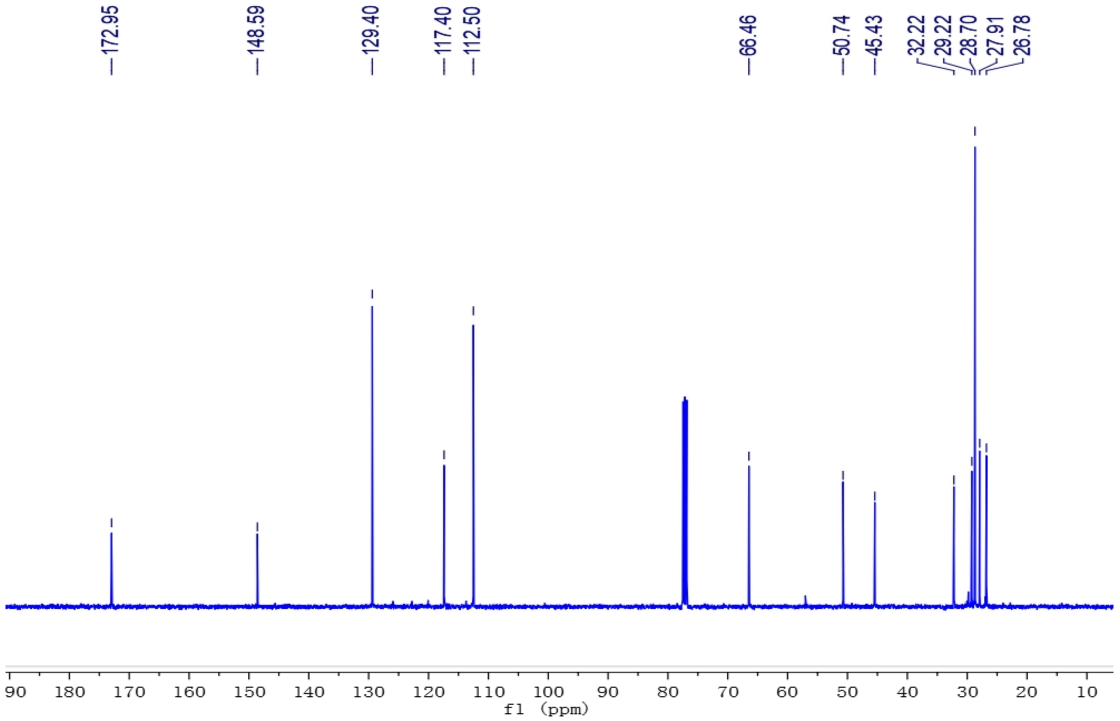

1-phenyl-N-(2,4,4-trimethylpentan-2-yl)pyrrolidine-2-carboxamide

^{1}H NMR (400 MHz, $CDCl_3$) δ 7.30 – 7.18 (m, 2H), 6.80 (t, *J* = 7.3 Hz, 1H), 6.60 (t, *J* = 12.5 Hz, 2H), 6.53 (s, 1H), 3.85 (dt, *J* = 13.0, 6.5 Hz, 1H), 3.65 – 3.54 (m, 1H), 3.20 (td, *J* = 9.6, 6.5 Hz, 1H), 2.22 (dt, *J* = 10.2, 5.7 Hz, 2H), 2.07 – 1.86 (m, 2H), 1.75 – 1.62 (m, 2H), 1.42 (d, *J* = 3.6 Hz, 3H), 1.32 (s, 3H), 0.89 (s, 9H). **^{13}C NMR (101 MHz, $CDCl_3$)** δ 172.59, 147.54, 129.21, 118.08, 113.16, 65.24, 54.76, 53.28, 49.74, 31.55, 31.38, 31.19, 28.45, 24.27. **HRMS (ESI-TOF)**: m/z calculated for $C_{19}H_{31}ON_2$ $[M+H]^+$: 303.2431. Found: 303.2429.

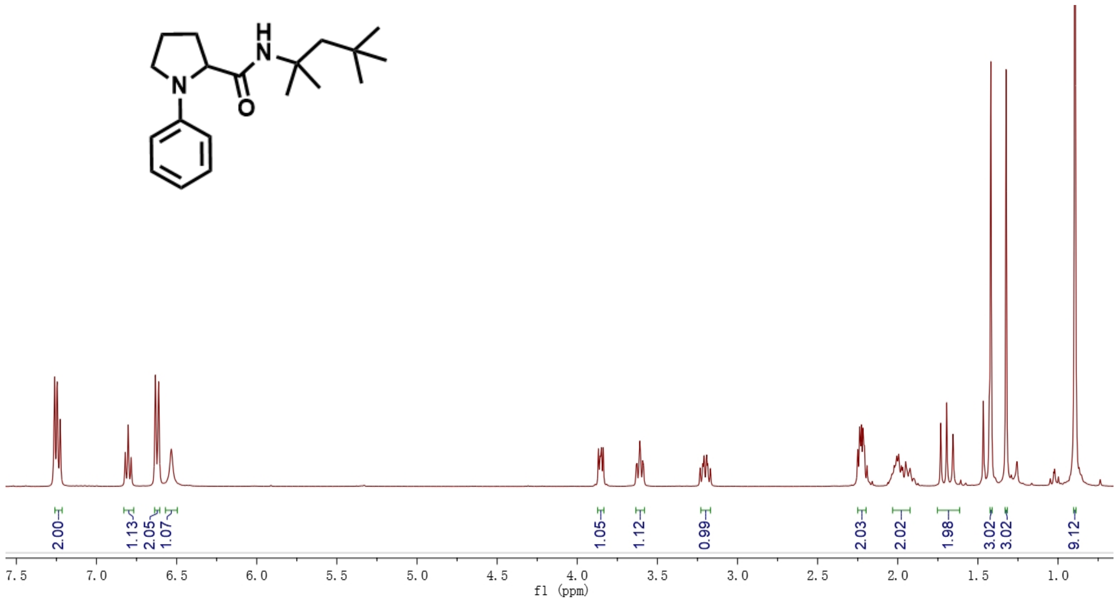

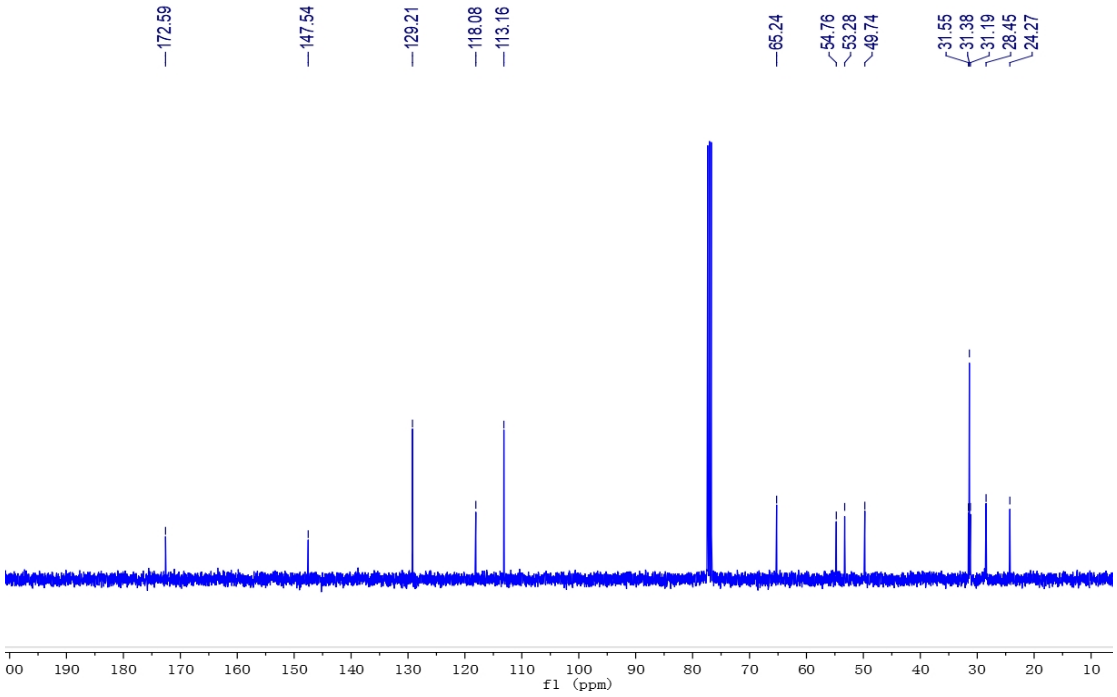

ethyl phenylprolylglycinate

^{1}H NMR (400 MHz, CDCl$_3$) δ 7.26 (t, J = 8.0 Hz, 2H), 7.00 (s, 1H), 6.82 (t, J = 7.3 Hz, 1H), 6.66 (d, J = 8.1 Hz, 2H), 4.23 – 4.12 (m, 3H), 4.05 (dt, J = 13.2, 6.5 Hz, 1H), 3.83 (dd, J = 18.1, 4.9 Hz, 1H), 3.70 – 3.64 (m, 1H), 3.24 (dd, J = 16.9, 8.8 Hz, 1H), 2.32 – 2.19 (m, 2H), 2.12 – 1.96 (m, 2H), 1.24 (t, J = 7.1 Hz, 3H). **^{13}C NMR (101 MHz, CDCl$_3$)** δ 174.48, 169.71, 147.59, 129.47, 118.35, 113.29, 64.43, 61.53, 49.82, 41.14, 31.64, 24.27, 14.23. **HRMS (ESI-TOF)**: m/z calculated for $C_{15}H_{21}O_3N_2$ $[M+H]^+$: 277.1547. Found: 277.1544.

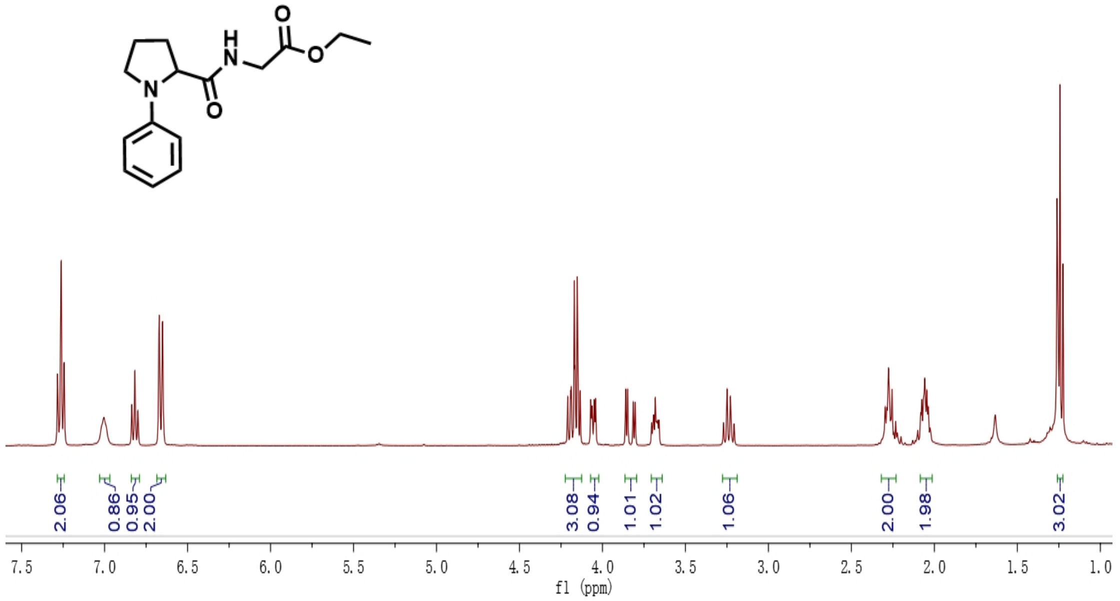

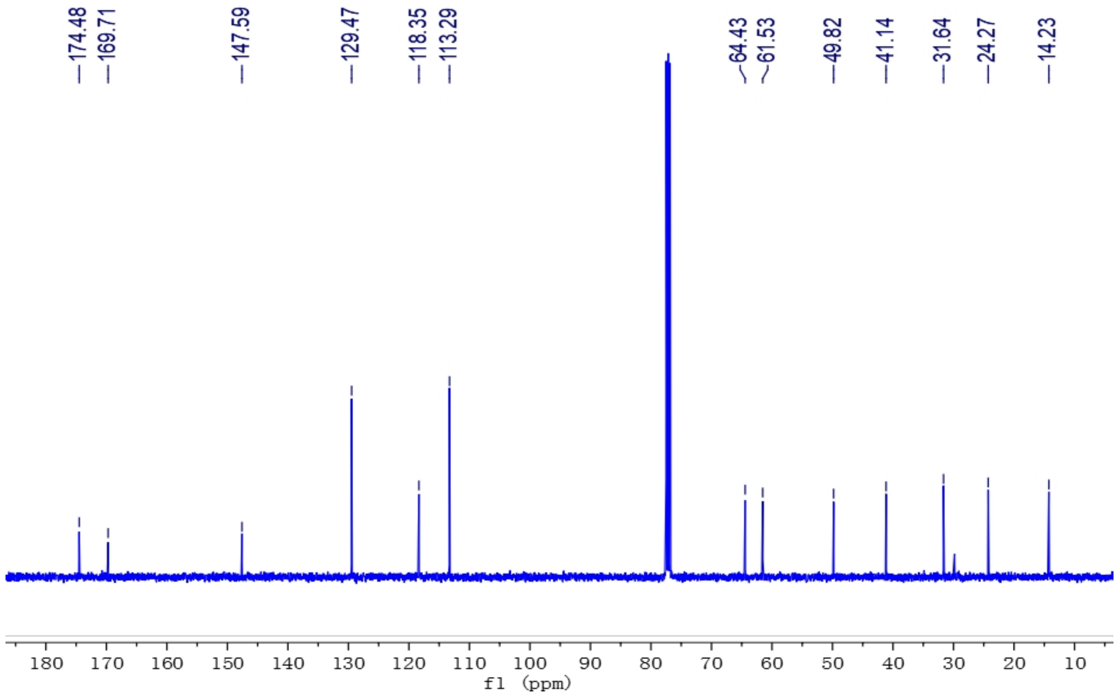

N-cyclohexyl-2-phenylcyclopentane-1-carboxamide

^{1}H NMR (400 MHz, CDCl$_3$) δ 7.30 – 7.17 (m, 2H), 6.81 (t, *J* = 7.3 Hz, 1H), 6.62 (d, *J* = 8.1 Hz, 2H), 6.40 (d, *J* = 8.0 Hz, 1H), 3.95 (dd, *J* = 12.8, 7.2 Hz, 1H), 3.86 – 3.71 (m, 1H), 3.63 (td, *J* = 8.8, 4.5 Hz, 1H), 3.27 – 3.14 (m, 1H), 2.33 – 2.18 (m, 2H), 2.07 – 1.88 (m, 2H), 1.80 (dt, *J* = 19.3, 10.8 Hz, 2H), 1.70 – 1.52 (m, 3H), 1.39 – 1.23 (m, 2H), 1.15 – 0.90 (m, 2H). **^{13}C NMR (101 MHz, CDCl$_3$)** δ 172.76, 147.73, 129.39, 118.23, 113.23, 64.81, 49.77, 47.95, 33.26, 32.99, 31.55, 25.55, 24.99, 24.85, 24.32. **HRMS (ESI-TOF)**: m/z calculated for $C_{17}H_{25}ON_2$ [M+H]$^+$: 273.1961. Found: 273.1963.

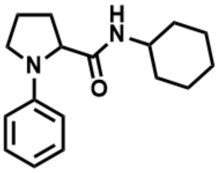

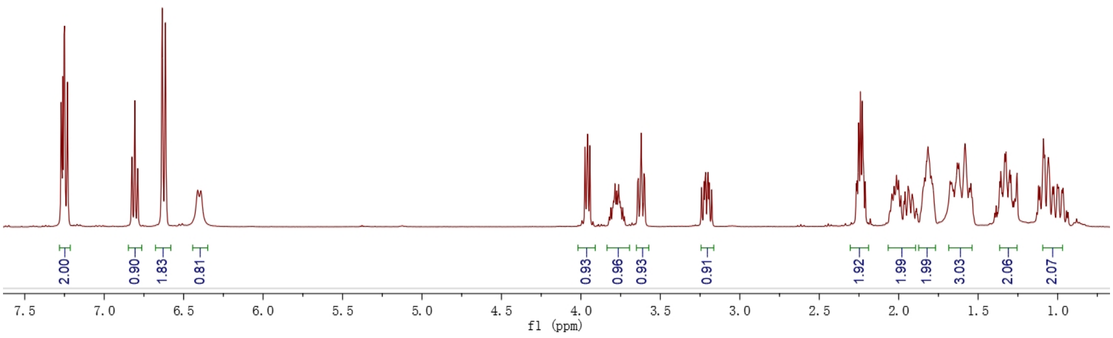

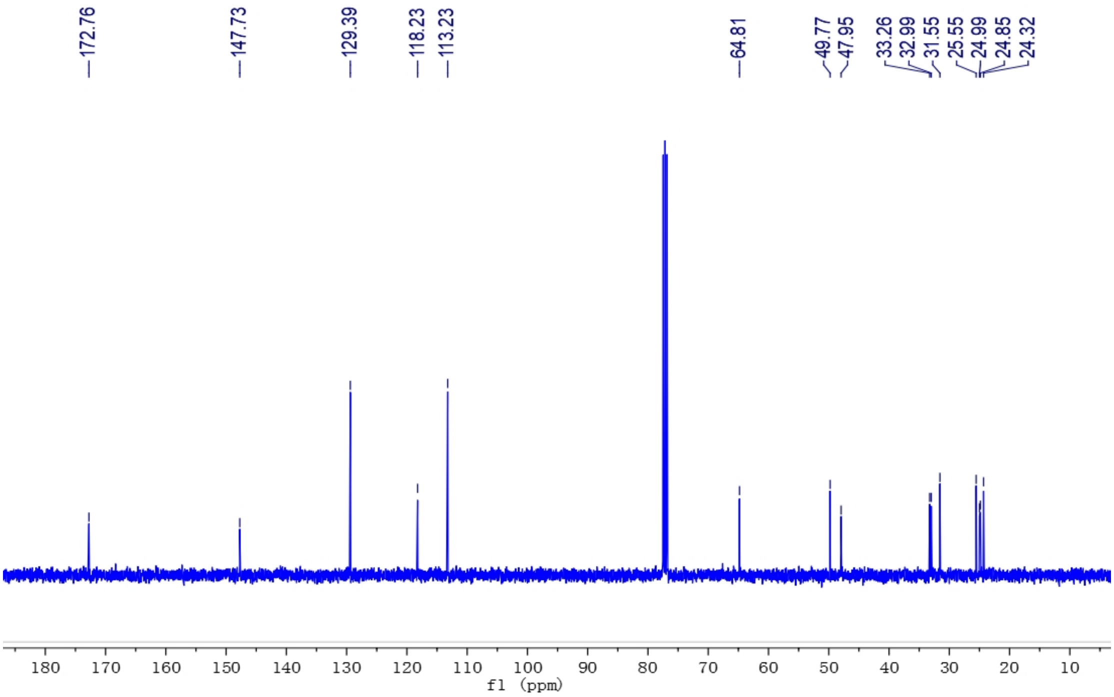

N-benzyl-1-phenylpyrrolidine-2-carboxamide

^{1}H NMR (400 MHz, $CDCl_3$) δ 7.33 – 7.20 (m, 5H), 7.15 (d, *J* = 6.7 Hz, 2H), 6.89 (s, 1H), 6.81 (t, *J* = 7.3 Hz, 1H), 6.64 (d, *J* = 8.0 Hz, 2H), 4.54 (dd, *J* = 15.0, 6.5 Hz, 1H), 4.35 (dd, *J* = 15.0, 5.7 Hz, 1H), 4.07 (dt, *J* = 12.7, 6.3 Hz, 1H), 3.65 – 3.56 (m, 1H), 3.26 – 3.16 (m, 1H), 2.30 (dt, *J* = 10.8, 6.6 Hz, 2H), 2.09 – 1.89 (m, 2H). **^{13}C NMR (101 MHz, $CDCl_3$)** δ 173.95, 147.59, 138.37, 129.47, 128.74, 127.49, 118.36, 113.29, 64.61, 49.81, 43.22, 31.65, 24.42. **HRMS (ESI-TOF)**: m/z calculated for $C_{18}H_{21}ON_2$ [M+H]$^+$: 281.1648. Found: 281.1646.

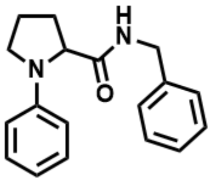

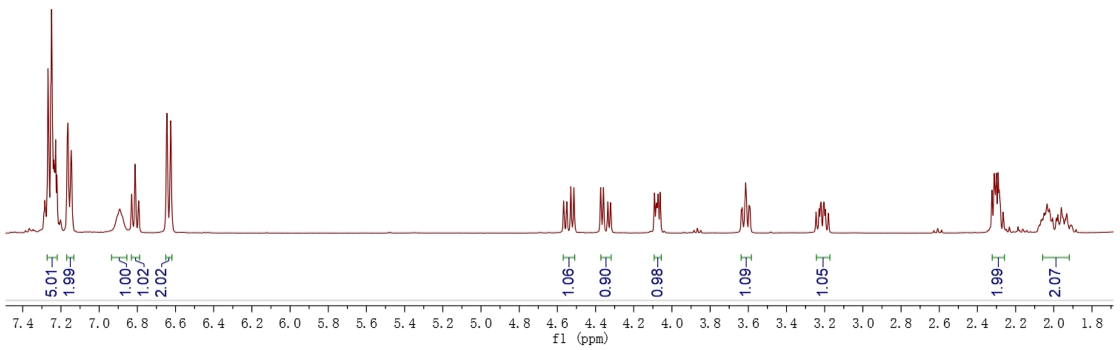

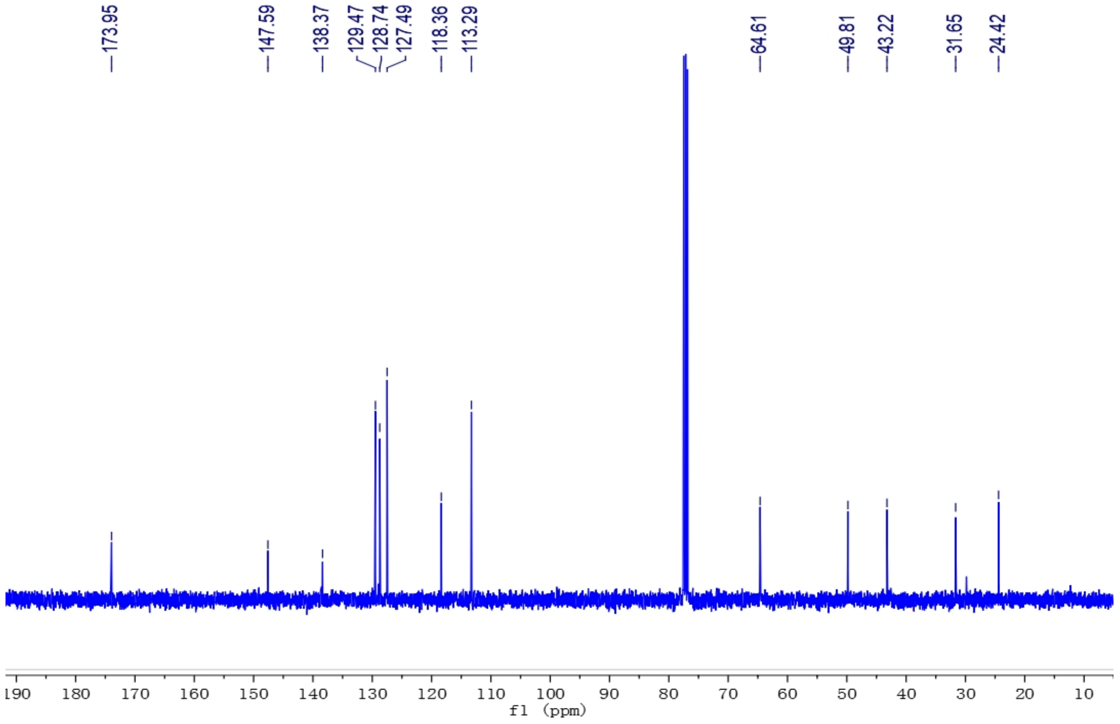

N-(2,6-dimethylphenyl)-1-phenylpyrrolidine-2-carboxamide

^{1}H NMR (400 MHz, $CDCl_3$) δ 7.18 (dd, J = 11.5, 4.7 Hz, 3H), 7.13 – 7.07 (m, 2H), 6.65 (t, J = 7.3 Hz, 1H), 6.54 (d, J = 8.0 Hz, 2H), 5.59 (d, J = 8.3 Hz, 1H), 3.57 – 3.40 (m, 2H), 2.54 – 2.42 (m, 1H), 2.25 – 2.17 (m, 1H), 2.16 (s, 3H), 2.13 (s, 3H), 2.09 – 2.02 (m, 1H), 1.94 (s, 2H). **^{13}C NMR (101 MHz, $CDCl_3$)** δ 176.15, 173.07, 146.62, 137.36, 135.81, 135.42, 129.25, 129.11, 129.00, 116.34, 112.11, 63.42, 48.31, 30.96, 25.56, 22.83, 18.30, 17.88 . **HRMS (ESI-TOF)**: m/z calculated for $C_{19}H_{23}ON_2$ $[M+H]^+$: 295.1805. Found: 295.1804.

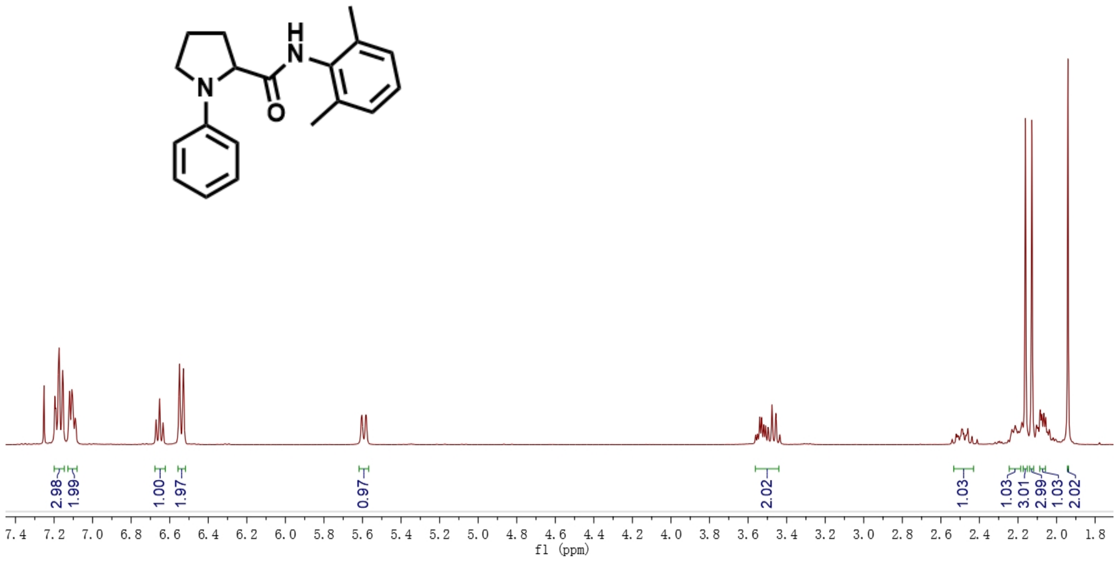

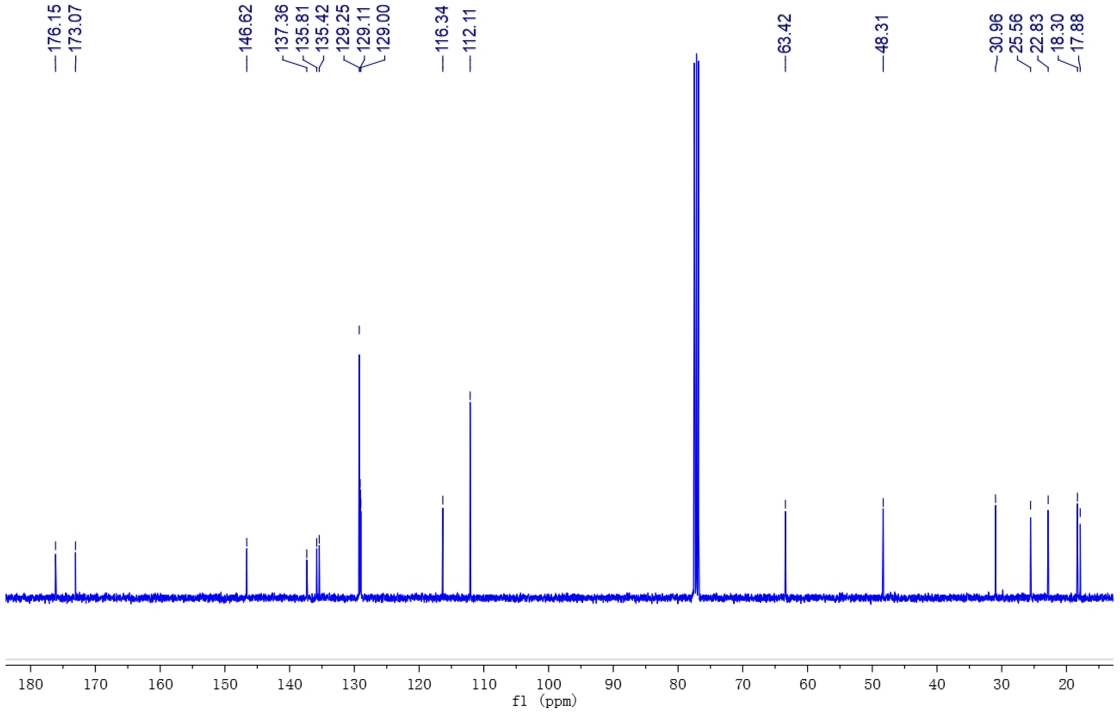

9.2 Dealkylation/Acylation of Tertiary Amines to Access Amides

N-methyl-N-phenylbenzamide

^{1}H NMR (400 MHz, $CDCl_3$) δ 7.21 (s, 2H), 7.14 (s, 3H), 7.08 (s, 3H), 6.97 (s, 2H), 3.43 (s, 3H). **^{13}C NMR (101 MHz, $CDCl_3$)** δ 170.72, 144.93, 135.93, 129.59, 129.15, 128.72, 127.73, 126.92, 126.50, 38.42. **HRMS (ESI-TOF)**: m/z calculated for $C_{14}H_{14}NO$ $[M+H]^+$: 202.1070. Found: 202.1055.

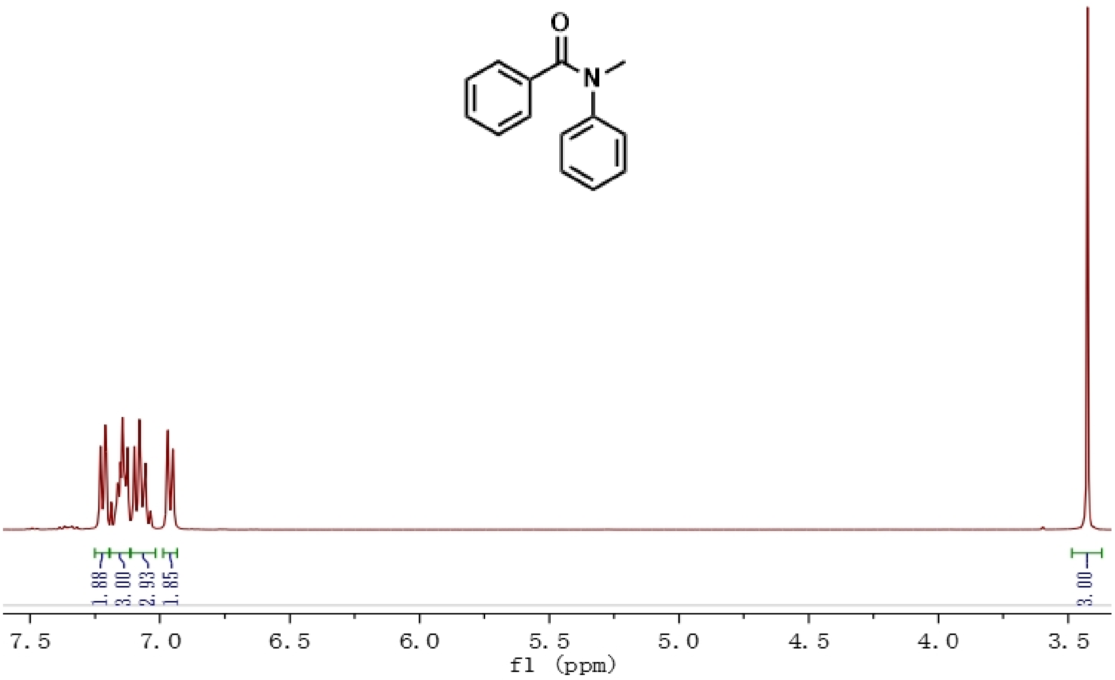

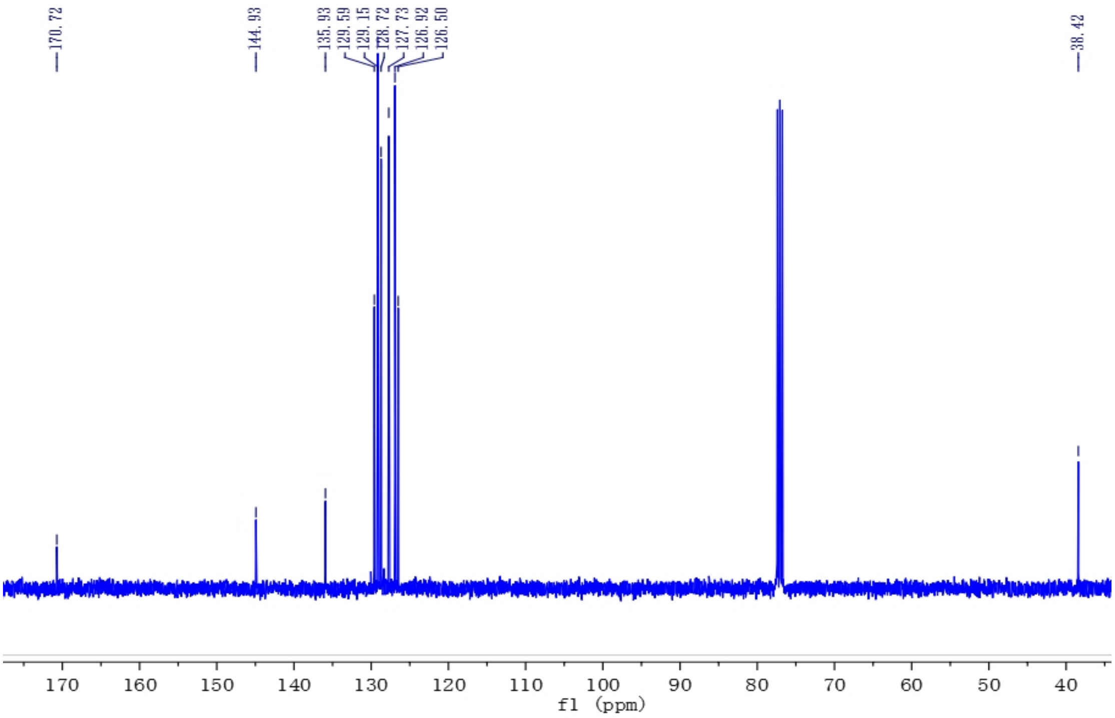

N,2-dimethyl-N-phenylbenzamide

^{1}H NMR (400 MHz, $CDCl_3$) δ 7.25 – 7.14 (m, 1H), 7.15 – 7.05 (m, 2H), 7.04 – 6.85 (m, 6H), 3.55 – 3.25 (m, 3H), 2.26 (s, 3H). **^{13}C NMR (101 MHz, $CDCl_3$)** δ 171.43, 144.02, 138.44, 134.68, 133.74, 130.95, 128.90, 127.46, 126.46, 125.83, 37.56, 21.16, 19.47. **HRMS (ESI-TOF)**: m/z calculated for $C_{15}H_{15}NO$ $[M+H]^+$: 226.1266. Found: 226.1266.

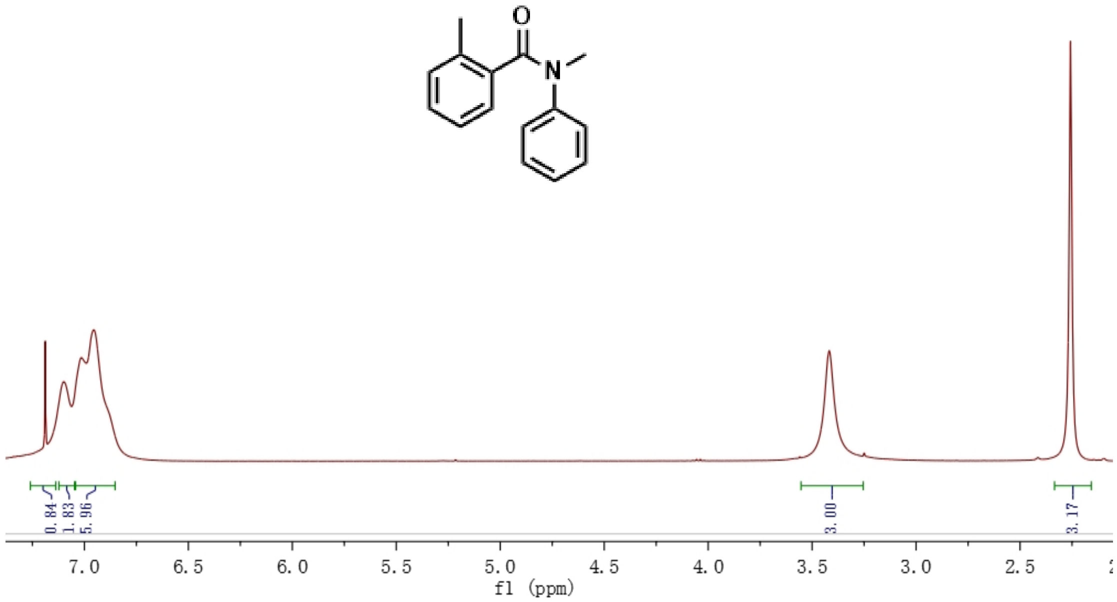

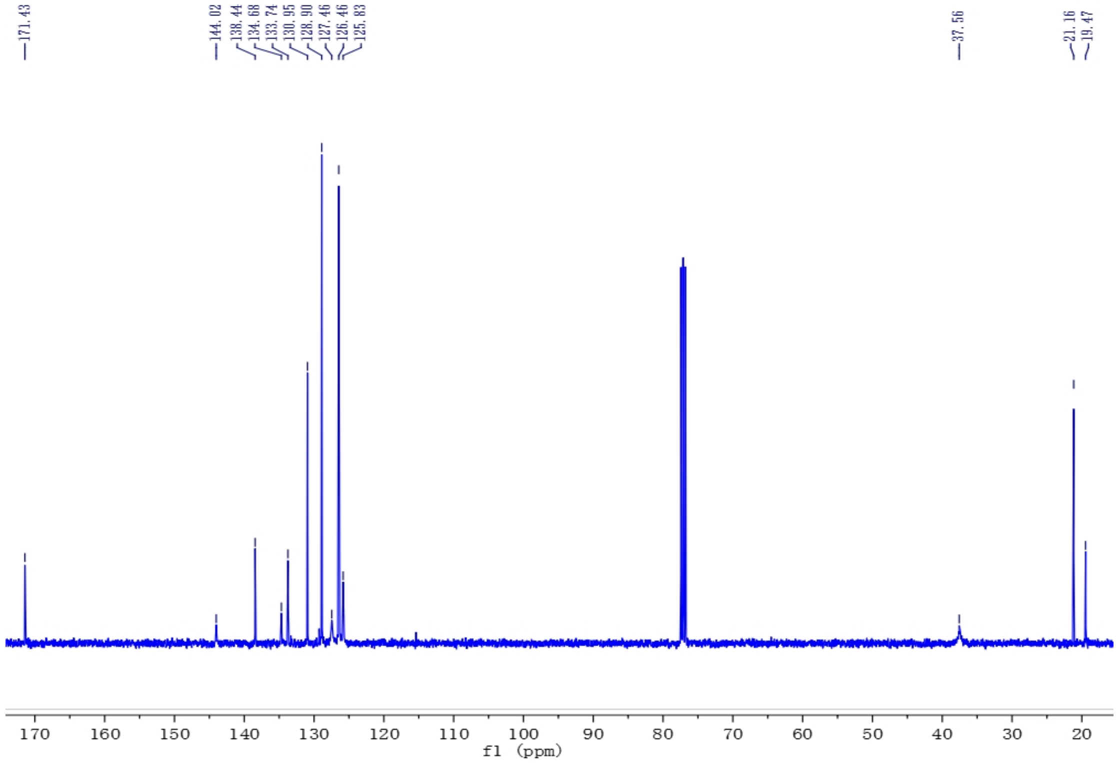

2-Chloro-N-methyl-N-phenylbenzamide

^{1}H NMR (400 MHz, CDCl$_3$) δ 7.27 (s, 1H), 7.21 – 7.15 (m, 2H), 7.14 – 7.06 (m, 2H), 6.99 (dt, J = 13.3, 7.6 Hz, 4H), 3.41 (s, 3H).**^{13}C NMR (101 MHz, CDCl3)** δ 169.18, 144.49, 138.26, 137.76, 133.95, 129.79, 129.44, 129.06, 129.01, 127.00, 126.98, 126.83, 38.50. **HRMS (ESI-TOF)**: m/z calculated for $C_{14}H_{13}ClNO$ [M+H]$^+$: 246.0680. Found: 246.0680.

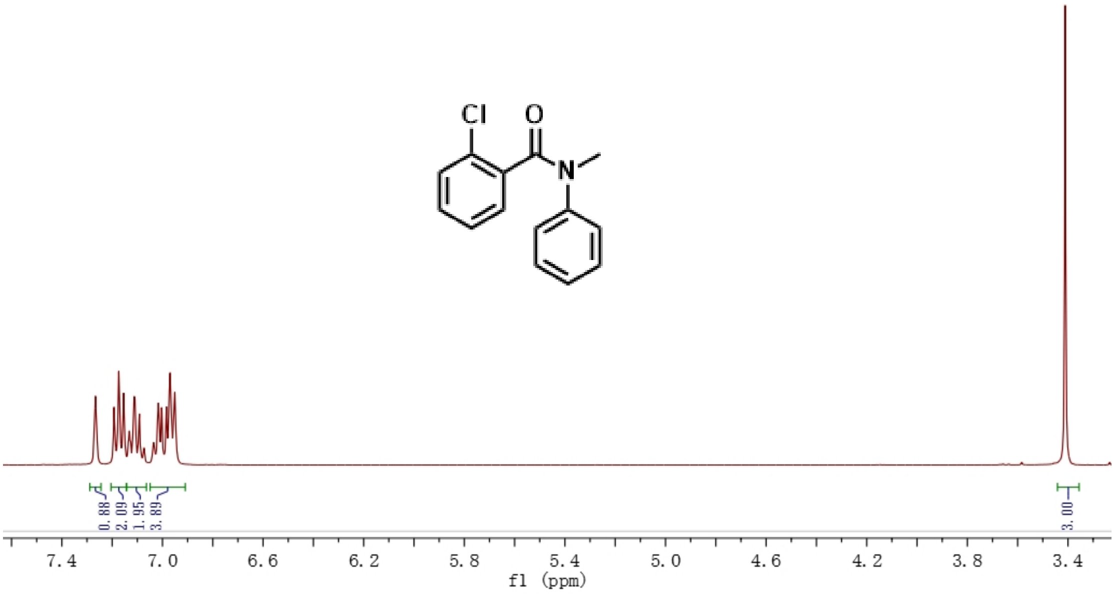

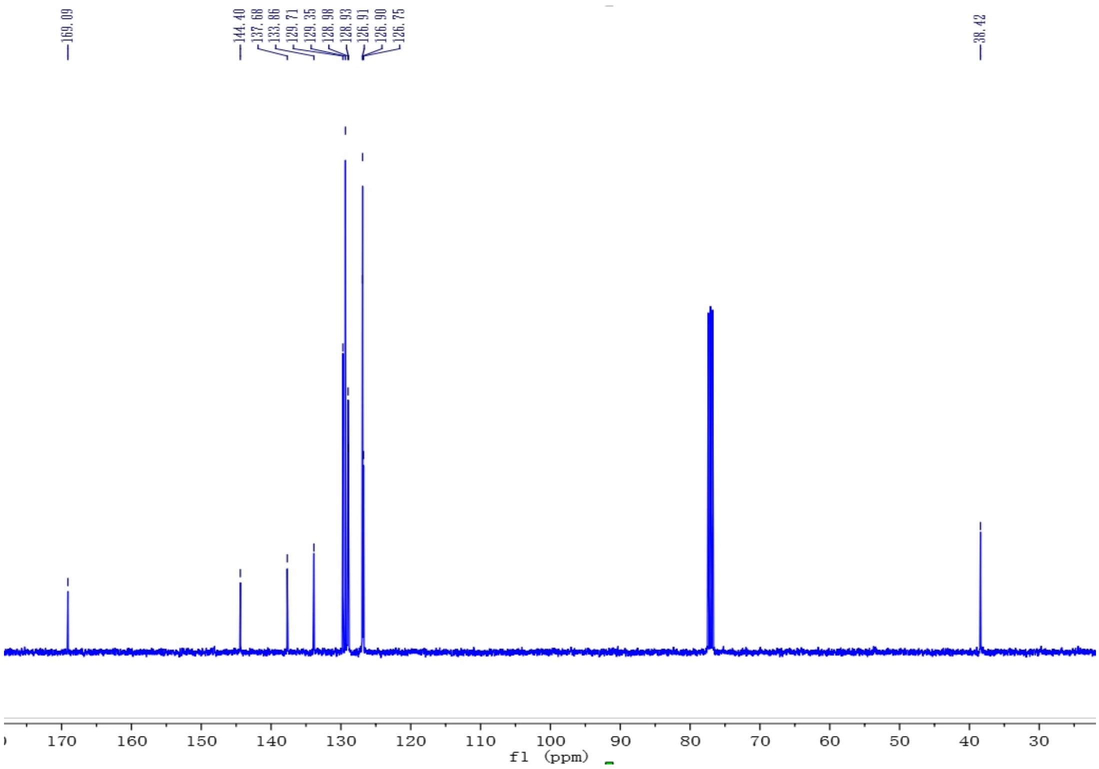

N,3-dimethyl-N-phenylbenzamide

^{1}H NMR (400 MHz, CDCl3) δ 7.20 (dd, J = 10.4, 4.8 Hz, 3H), 7.10 (dd, J = 8.3, 6.4 Hz, 1H), 7.05 – 6.95 (m, 5H), 3.47 (s, 3H), 2.20 (s, 3H). **^{13}C NMR (101 MHz, CDCl3)** δ 170.81, 144.95, 137.50, 135.86, 130.32, 129.41, 129.09, 127.45, 126.85, 126.43, 125.75, 38.37, 21.21. **HRMS (ESI-TOF)**: m/z calculated for $C_{15}H_{15}NO$ [M+H]$^{+}$: 226.1226. Found: 226.1227.

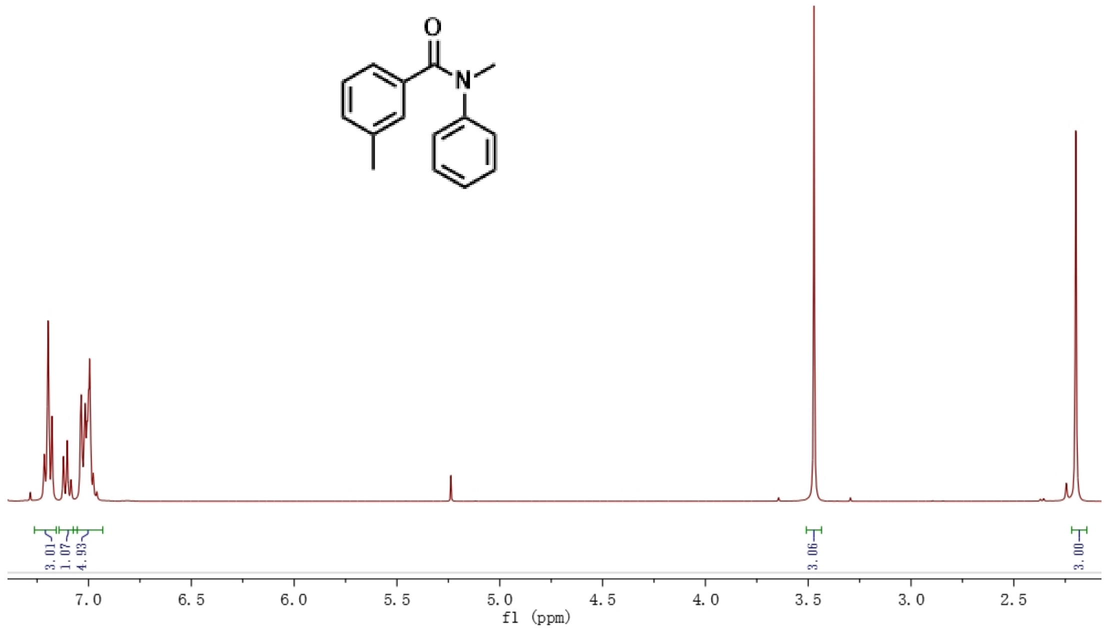

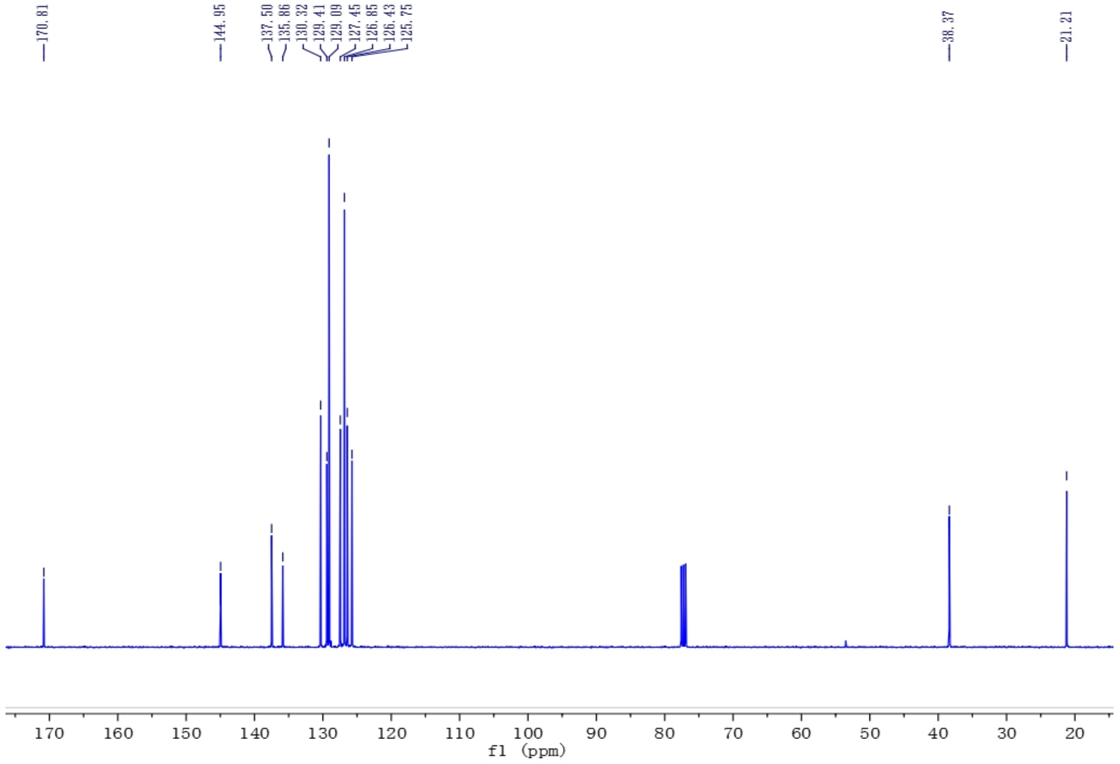

3-methoxy-N-methyl-N-phenylbenzamide

^{1}H NMR (400 MHz, CDCl3) δ 7.29 – 7.21 (m, 2H), 7.19 – 7.10 (m, 1H), 7.09 – 7.00 (m, 3H), 6.89 – 6.81 (m, 2H), 6.81 – 6.73 (m, 1H), 3.65 (s, 3H), 3.50 (s, 3H). **^{13}C NMR (101 MHz, CDCl3)** δ 170.70, 159.22, 145.25, 137.40, 129.47, 129.05, 127.12, 126.83, 121.54, 116.39, 113.98, 55.49, 38.72. **HRMS (ESI-TOF)**: m/z calcd for $C_{15}H_{15}NO_2$ $[M+H]^+$: 242.1176. Found: 242.1178.

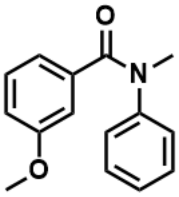

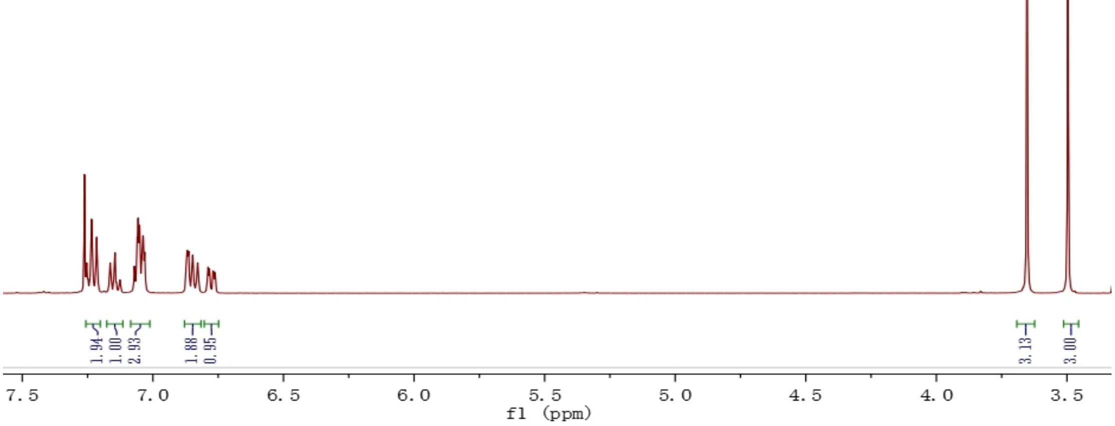

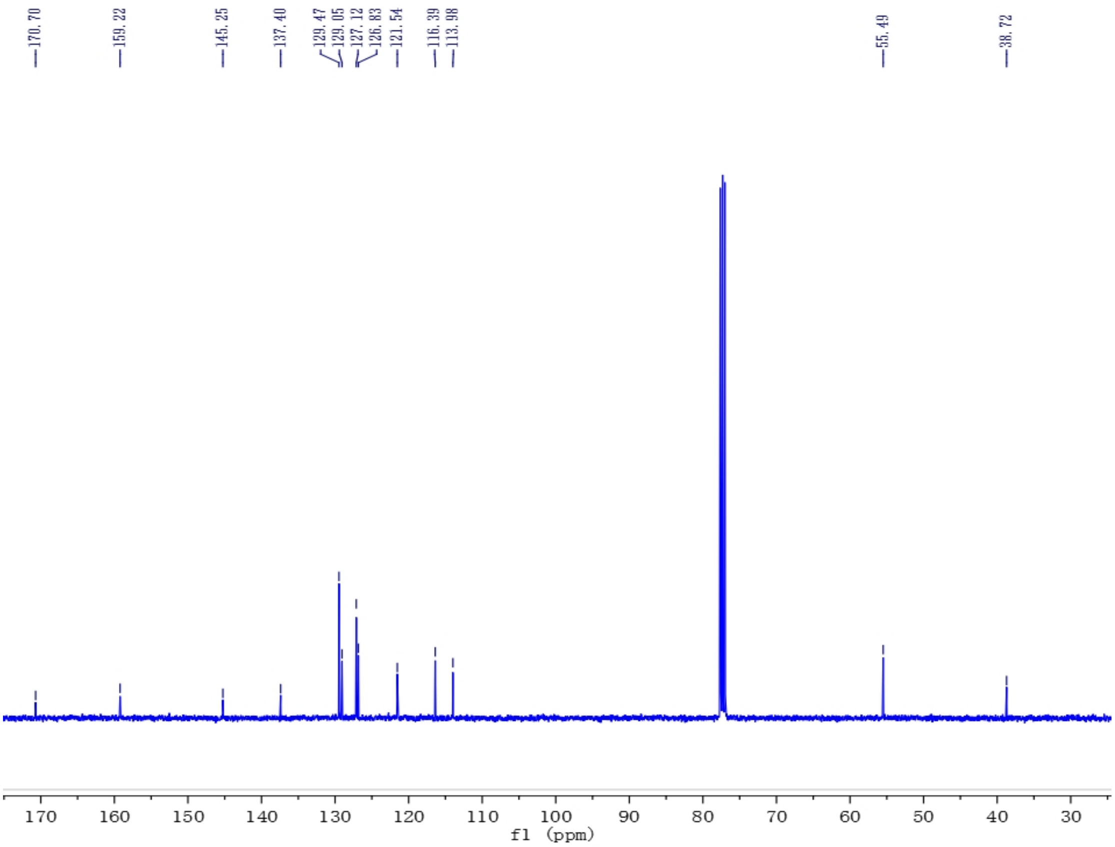

3-fluoro-N-methyl-N-phenylbenzamide

^{1}H NMR (400 MHz, CDCl3) δ 7.17 (dd, J = 13.2, 6.0 Hz, 1H), 7.12 – 7.00 (m, 1H), 6.98 – 6.91 (m, 2H), 6.84 (d, J = 1.8 Hz, 1H), 3.41 (s, 1H). **^{13}C NMR (101 MHz, CDCl3)** δ 169.59, 164.47, 161.98, 144.92, 132.00, 131.17, 131.08, 129.34, 126.93, 126.70, 114.94, 114.73, 38.53. **HRMS (ESI-TOF)**: m/z calculated for $C_{14}H_{12}FNO$ $[M+H]^{+}$: 230.0976. Found: 230.0976.

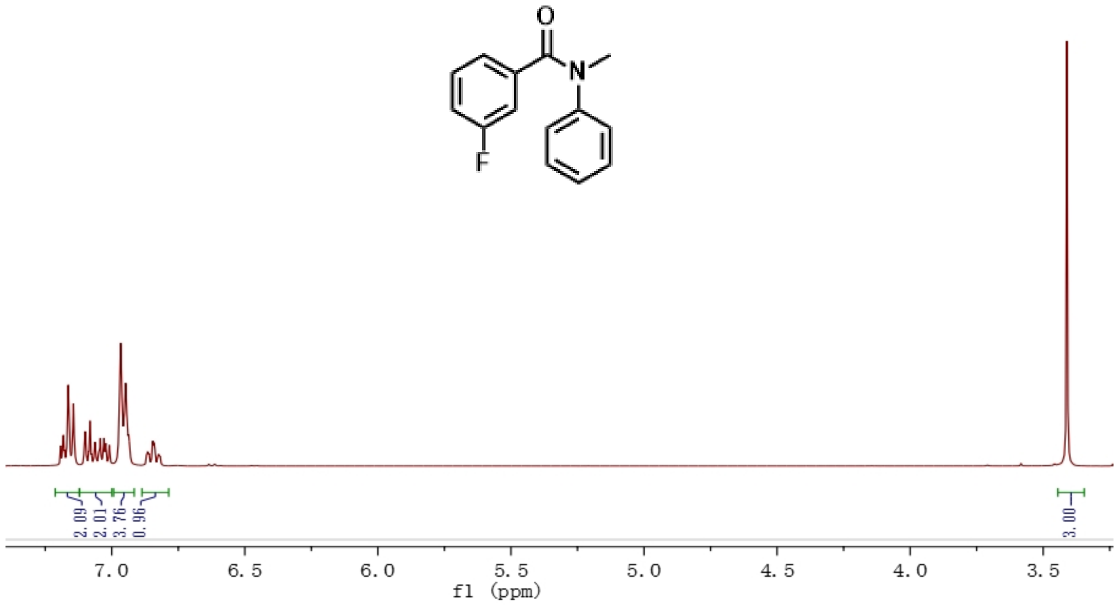

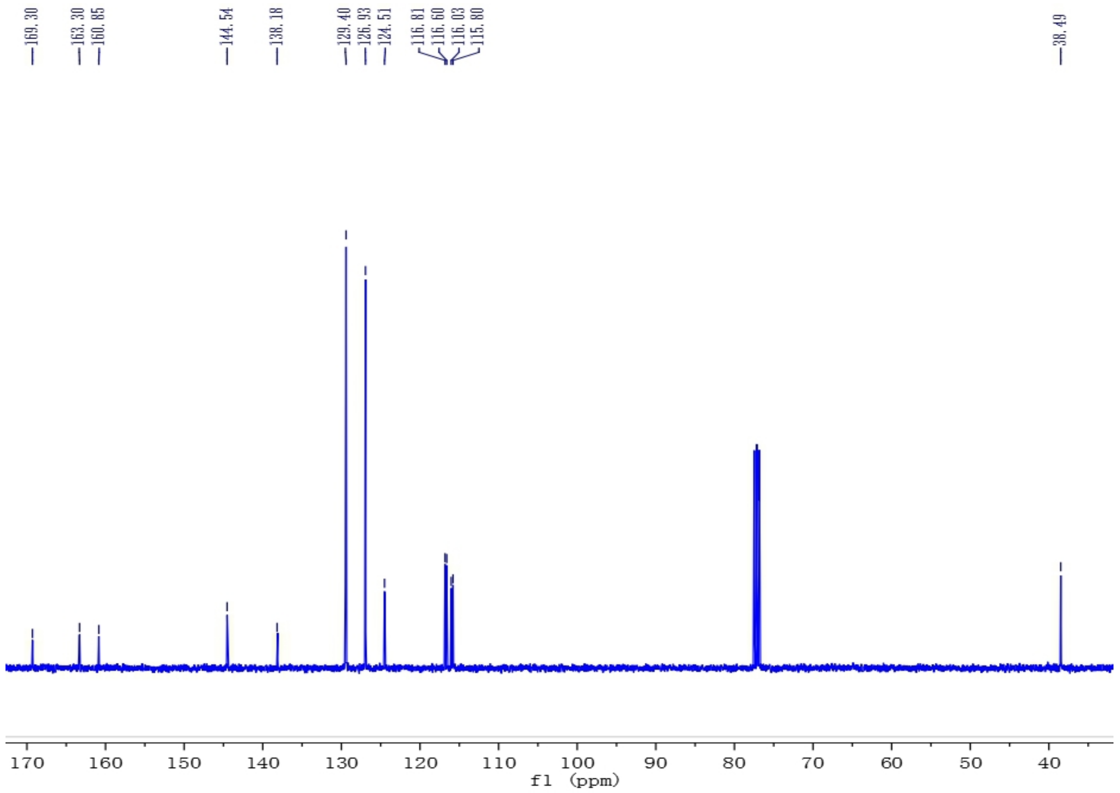

N,4-dimethyl-N-phenylbenzamide

^{1}H NMR (400 MHz, CDCl3) δ 7.11 (dd, J = 22.2, 13.9 Hz, 5H), 7.00 – 6.93 (m, 2H), 6.88 (d, J = 8.0 Hz, 2H), 3.41 (s, 3H), 2.17 (s, 3H). **^{13}C NMR (101 MHz, CDCl3)** δ 170.81, 145.31, 139.90, 133.04, 129.23, 128.99, 128.47, 126.98, 126.44, 38.60, 21.43. **HRMS (ESI-TOF)**: m/z calculated for $C_{15}H_{15}NO$ [M+H]$^+$: 226.1226. Found: 226.1226.

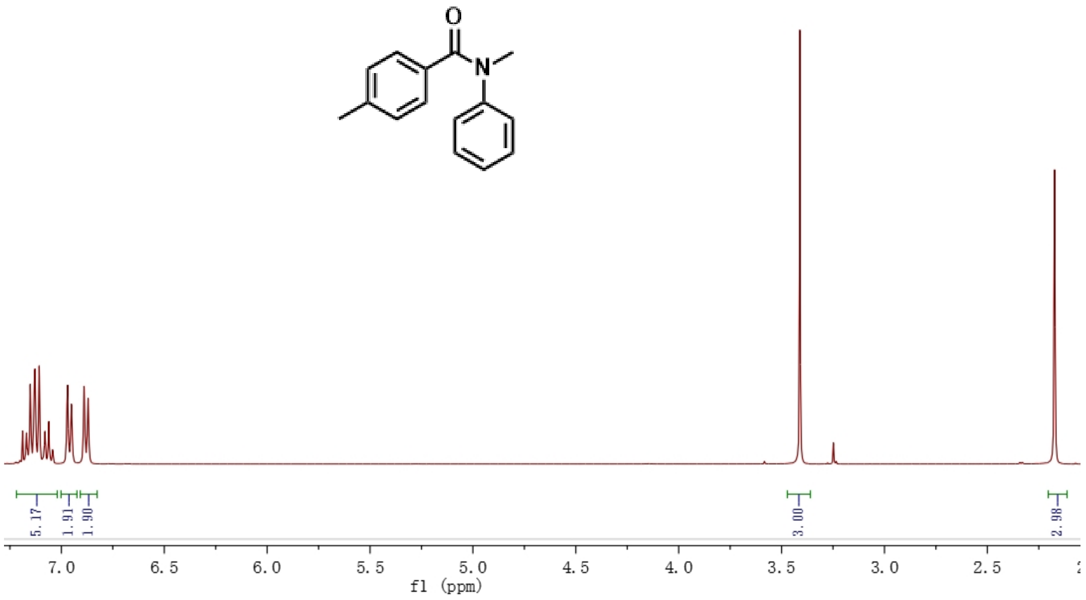

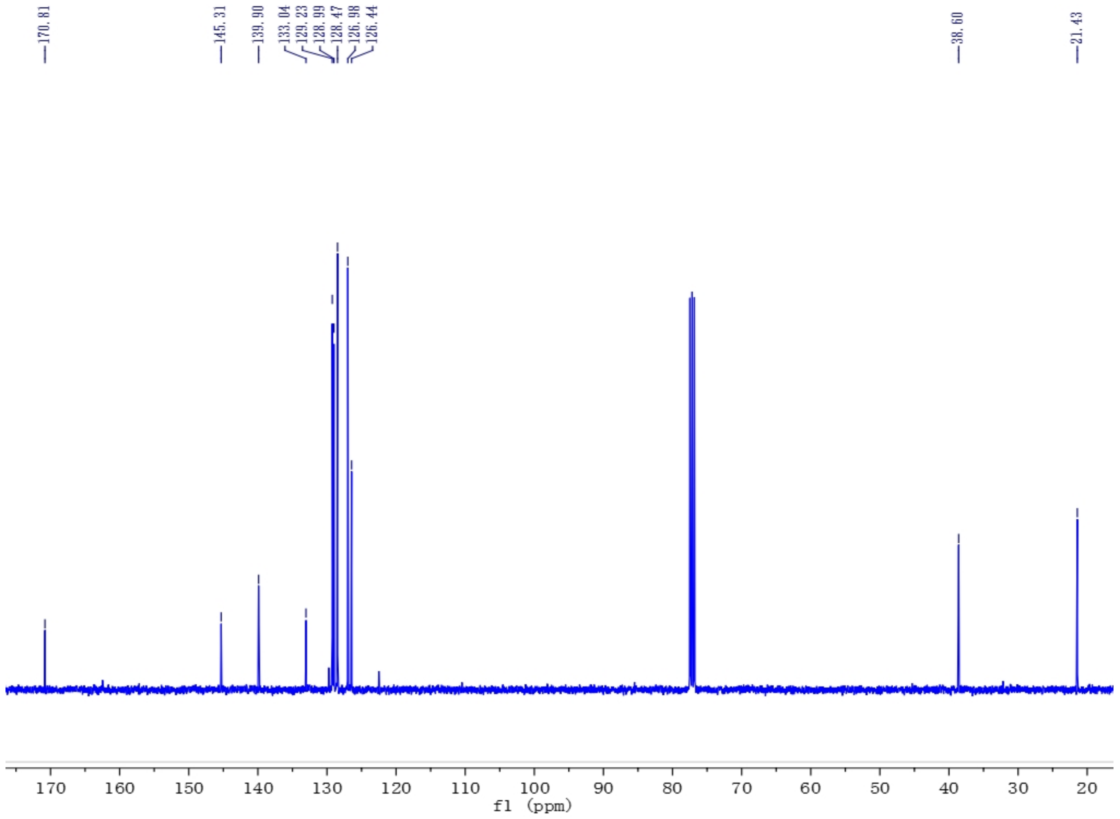

4-methoxy-N-methyl-N-phenylbenzamide

^{1}H NMR (400 MHz, CDCl3) δ 7.29 – 7.19 (m, 4H), 7.14 (t, J = 7.4 Hz, 1H), 7.07 – 7.00 (m, 2H), 6.70 – 6.61 (m, 2H), 3.72 (s, 3H), 3.48 (s, 3H). **^{13}C NMR (101 MHz, CDCl3)** δ 170.28, 160.60, 145.44, 130.89, 129.20, 127.96, 126.85, 126.30, 112.98, 55.17, 38.62. **HRMS (ESI-TOF)**: m/z calcd for $C_{15}H_{15}NO_2$ $[M+H]^+$: 242.1176. Found: 242.1176.

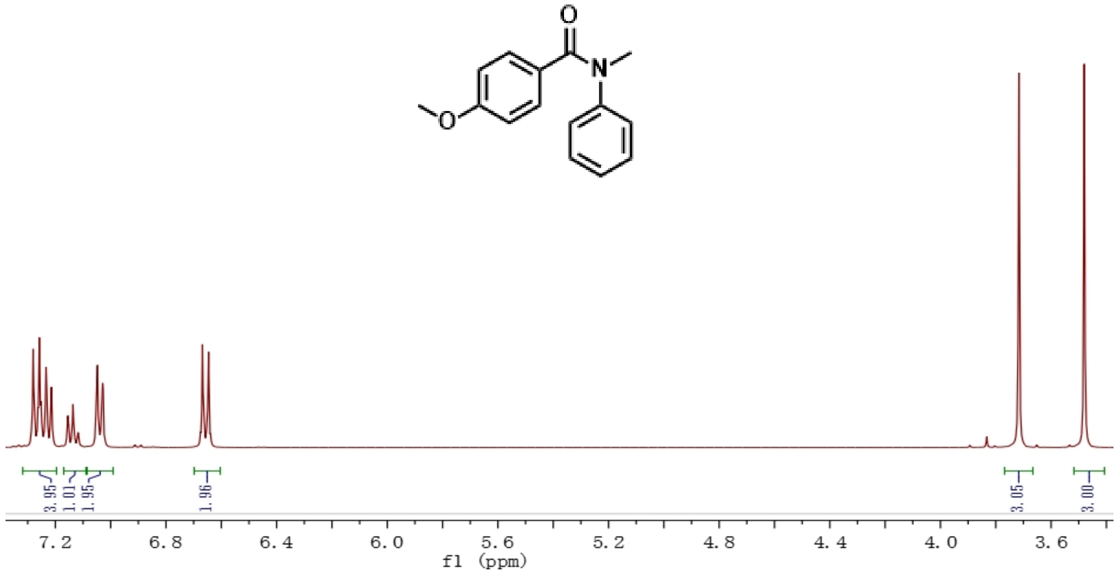

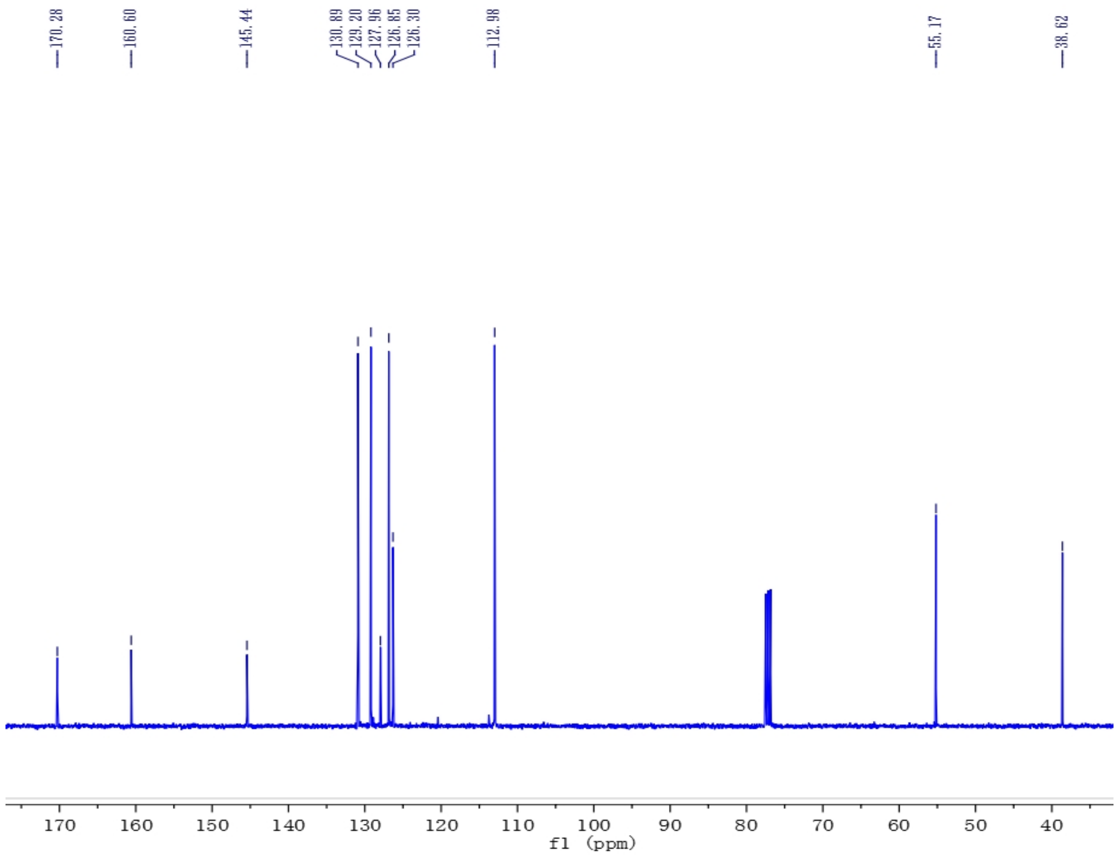

4-fluoro-N-methyl-N-phenylbenzamide

^{1}H NMR (400 MHz, CDCl3) δ 7.25 – 7.19 (m, 4H), 7.15 (t, J = 7.6 Hz, 1H), 7.07 (t, J = 7.4 Hz, 2H), 6.94 (d, J = 7.4 Hz, 2H), 6.75 (t, J = 8.7 Hz, 3H), 3.40 (s, 1H). **^{13}C NMR (101 MHz, CDCl3)** δ 169.59, 164.47, 161.98, 144.92, 132.00, 131.17, 131.08, 129.34, 126.93, 126.70, 114.94, 114.73, 38.53. **HRMS (ESI-TOF)**: m/z calculated for $C_{14}H_{12}FNO$ $[M+H]^{+}$: 230.0976. Found: 230.0976.

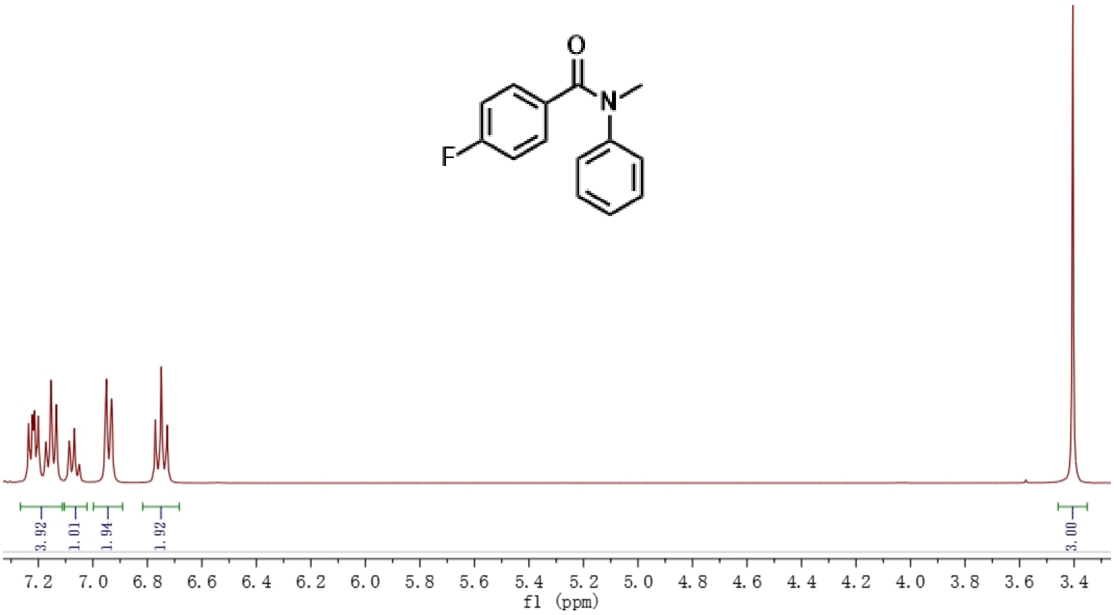

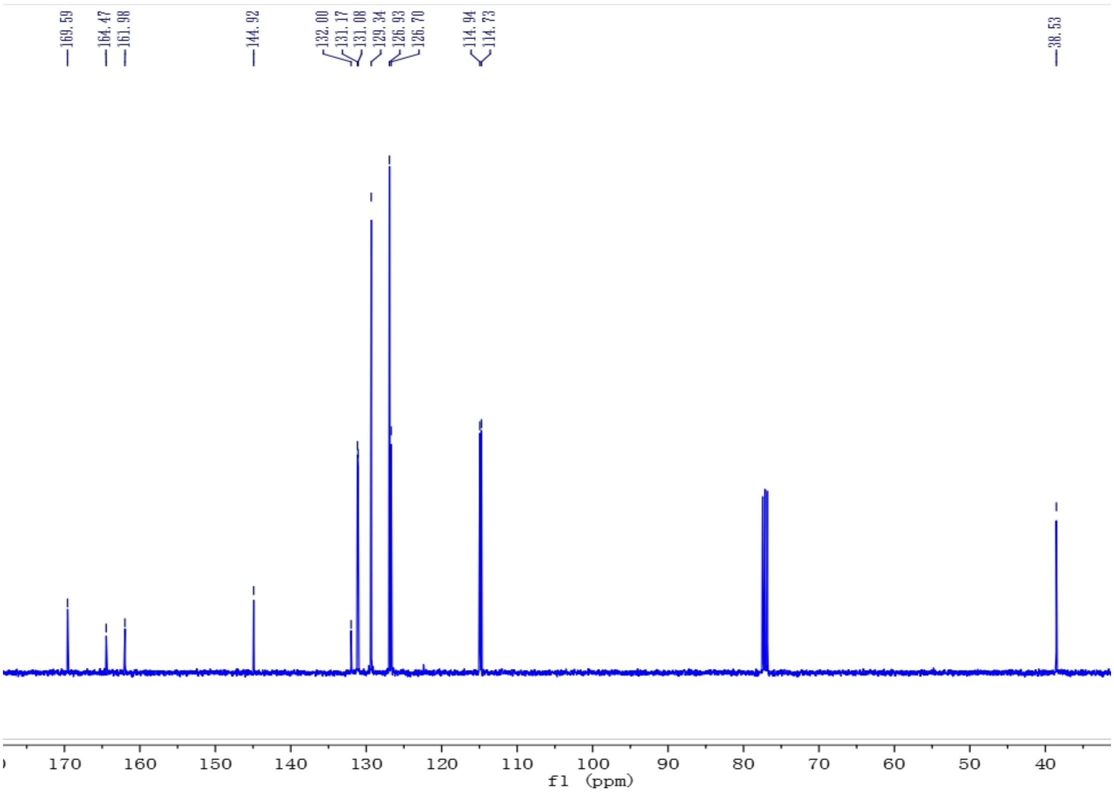

4-chloro-N-methyl-N-phenylbenzamide

^{1}H NMR (400 MHz, CDCl3) δ 7.18 (dd, J = 12.7, 4.9 Hz, 4H), 7.12 – 7.01 (m, 3H), 6.98 – 6.91 (m, 2H), 3.41 (s, 3H).**^{13}C NMR (101 MHz, CDCl3)** δ 169.59, 144.77, 135.80, 134.38, 130.35, 129.46, 128.12, 126.99, 126.89, 38.58. **HRMS (ESI-TOF)**: m/z calculated for $C_{14}H_{12}ClNO$ [M+H]$^{+}$: 246.0680. Found: 246.0680.

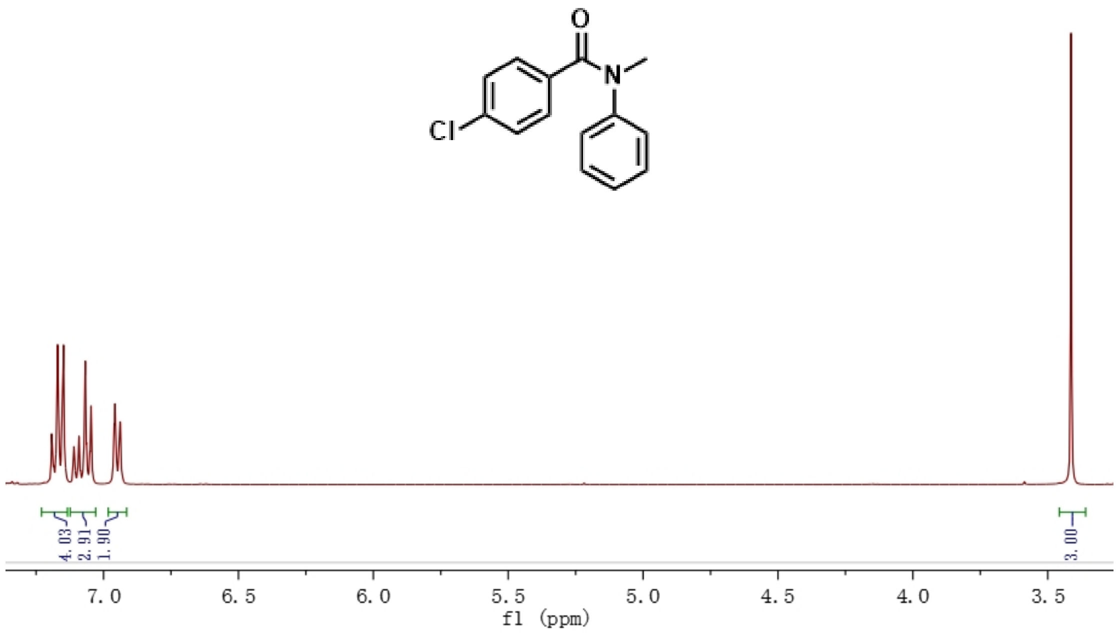

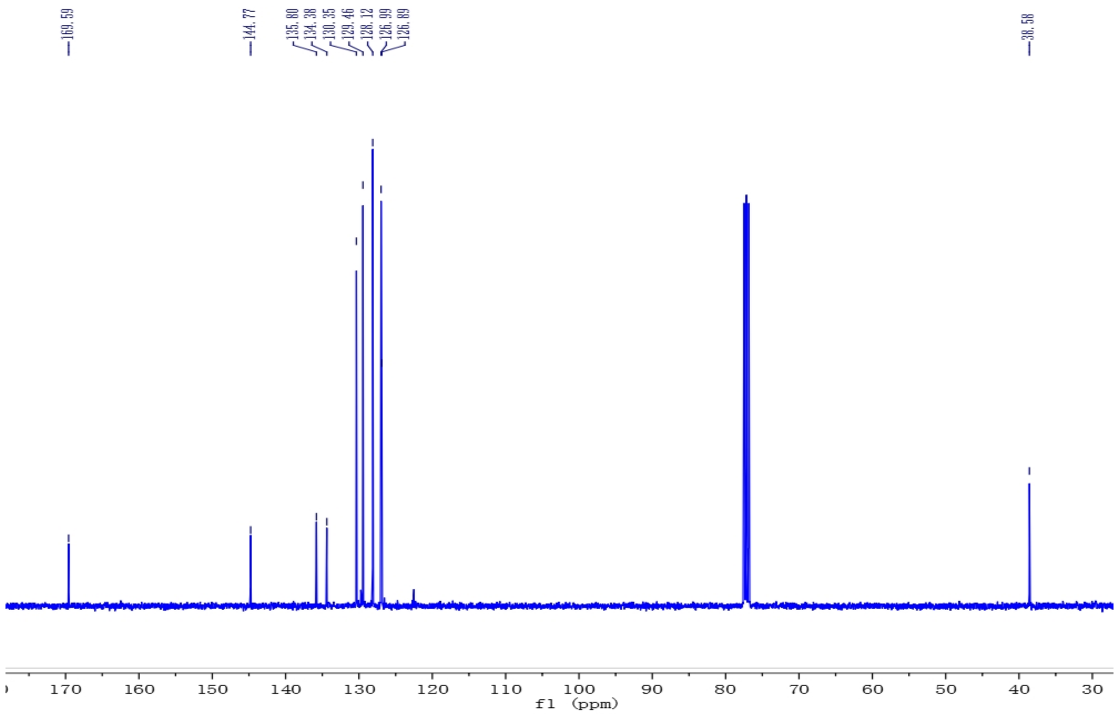

4-bromo-N-methyl-N-phenylbenzamide

^{1}H NMR (400 MHz, CDCl3) δ 7.28 – 7.14 (m, 4H), 7.13 – 7.05 (m, 3H), 6.95 (d, J = 7.4 Hz, 2H), 3.41 (s, 3H). **^{13}C NMR (101 MHz, CDCl3)** δ 169.64, 144.75, 134.87, 131.09, 130.56, 129.49, 127.00, 126.92, 124.22, 38.59. **HRMS (ESI-TOF)**: m/z calculated for $C_{14}H_{12}BrNO$ $[M+H]^{+}$: 290.0175. Found: 290.0175.

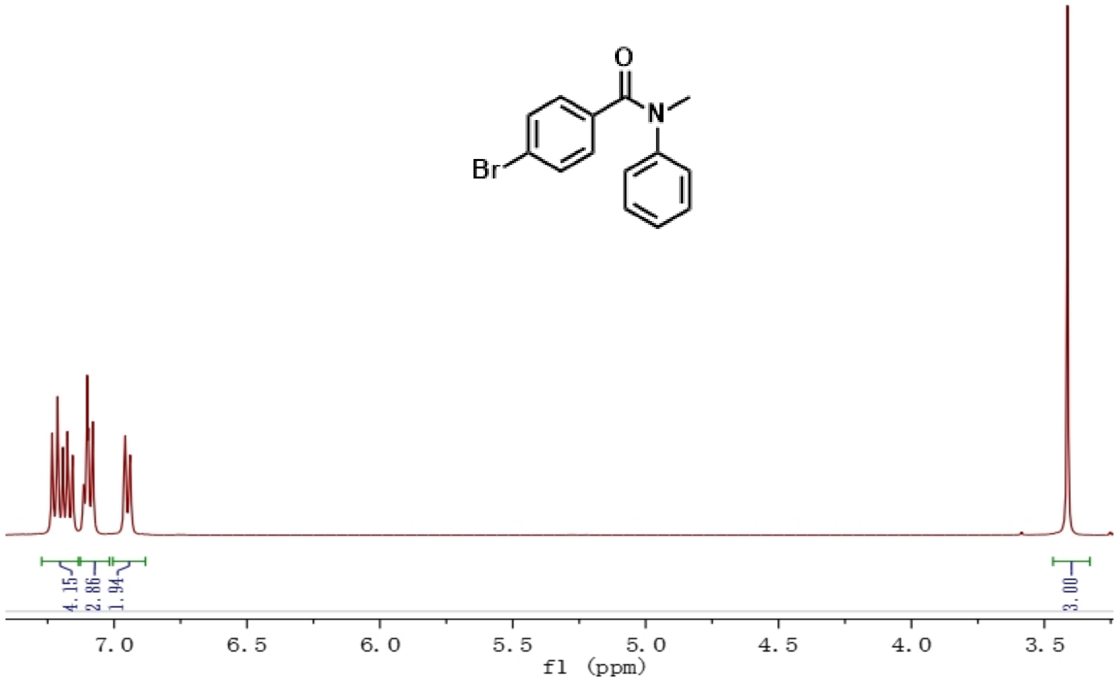

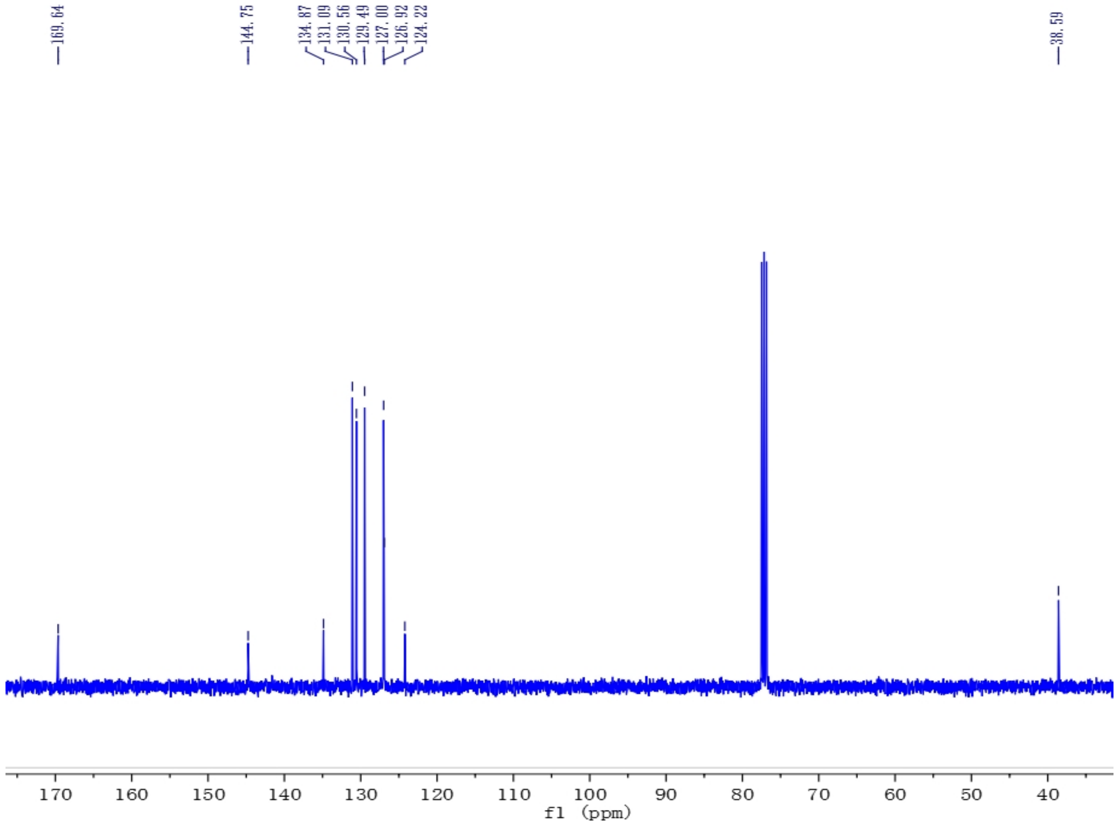

4-(tert-butyl)-N-methyl-N-phenylbenzamide

^{1}H NMR (400 MHz, CDCl3) δ 7.28 – 7.08 (m, 7H), 7.07 – 7.02 (m, 2H), 3.48 (s, 3H), 1.22 (s, 9H). **^{13}C NMR (101 MHz, CDCl3)** δ 170.59, 152.89, 145.14, 132.84, 129.12, 128.70, 126.83, 126.36, 124.62, 38.55, 34.66, 31.11. **HRMS (ESI-TOF)**: m/z calculated for $C_{18}H_{21}NO$ $[M+H]^{+}$: 268.1696. Found: 268.1696.

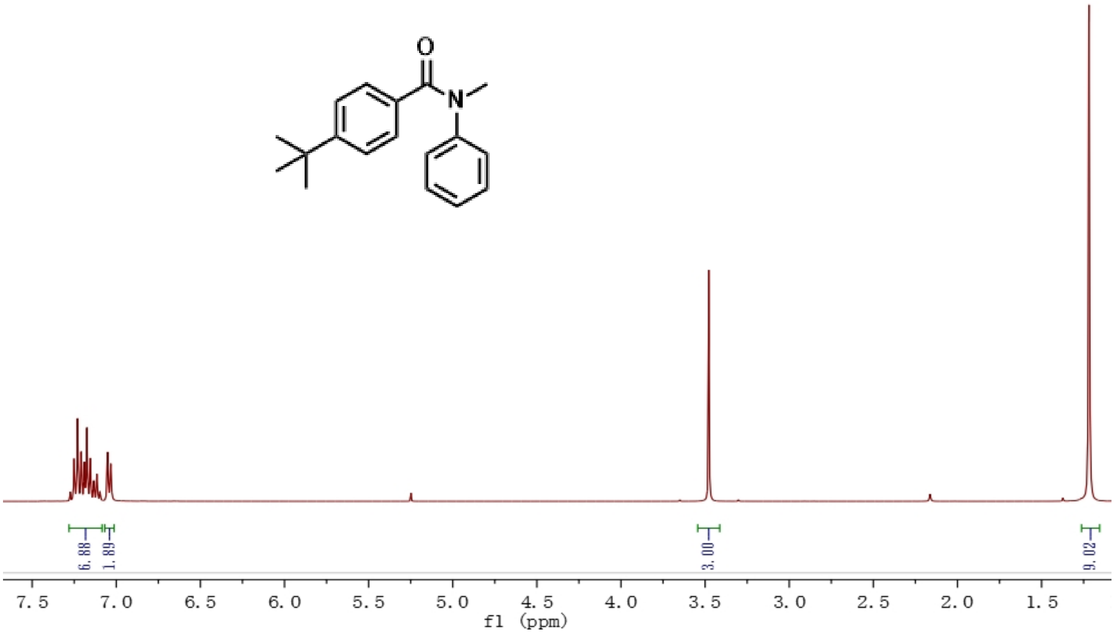

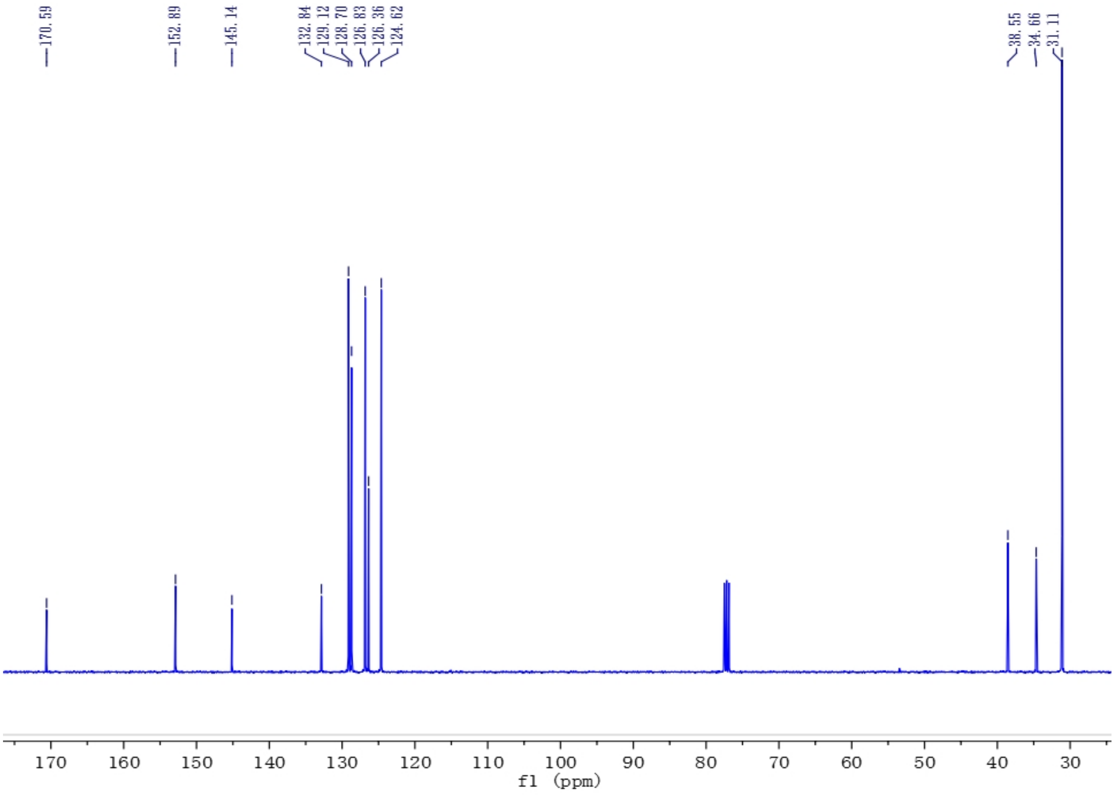

N,3,5-trimethyl-N-phenylbenzamide

^{1}H NMR (400 MHz, CDCl3) δ 7.11 (dd, J = 10.5, 4.8 Hz, 2H), 7.06 – 6.98 (m, 1H), 6.98 – 6.90 (m, 2H), 6.81 (s, 2H), 6.74 (s, 1H), 3.37 (s, 3H), 2.04 (s, 6H). **^{13}C NMR (101 MHz, CDCl3)** δ 170.92, 144.94, 137.10, 135.74, 131.11, 128.95, 126.74, 126.42, 126.30, 38.28, 21.00. **HRMS (ESI-TOF)**: m/z calculated for $C_{16}H_{17}NO$ [M+H]$^+$: 240.1393. Found: 240.1393.

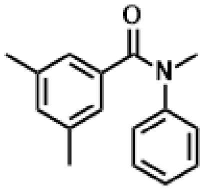

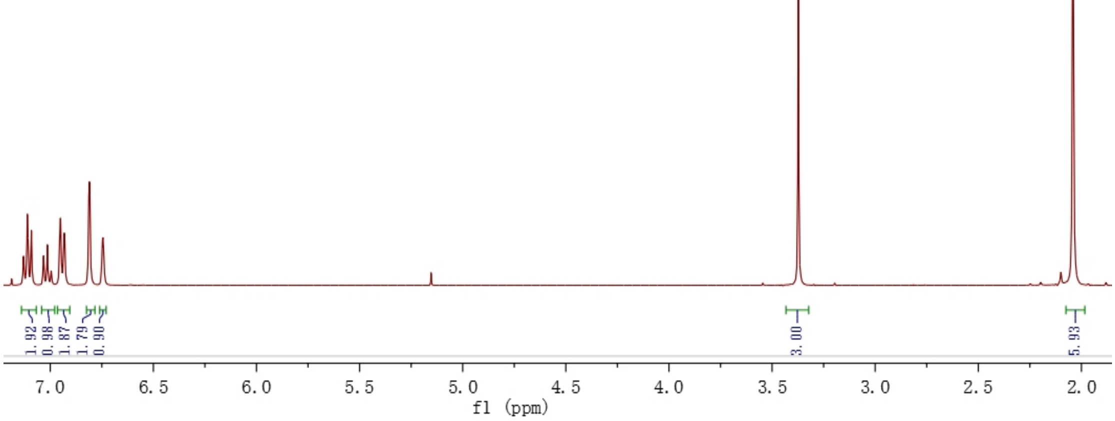

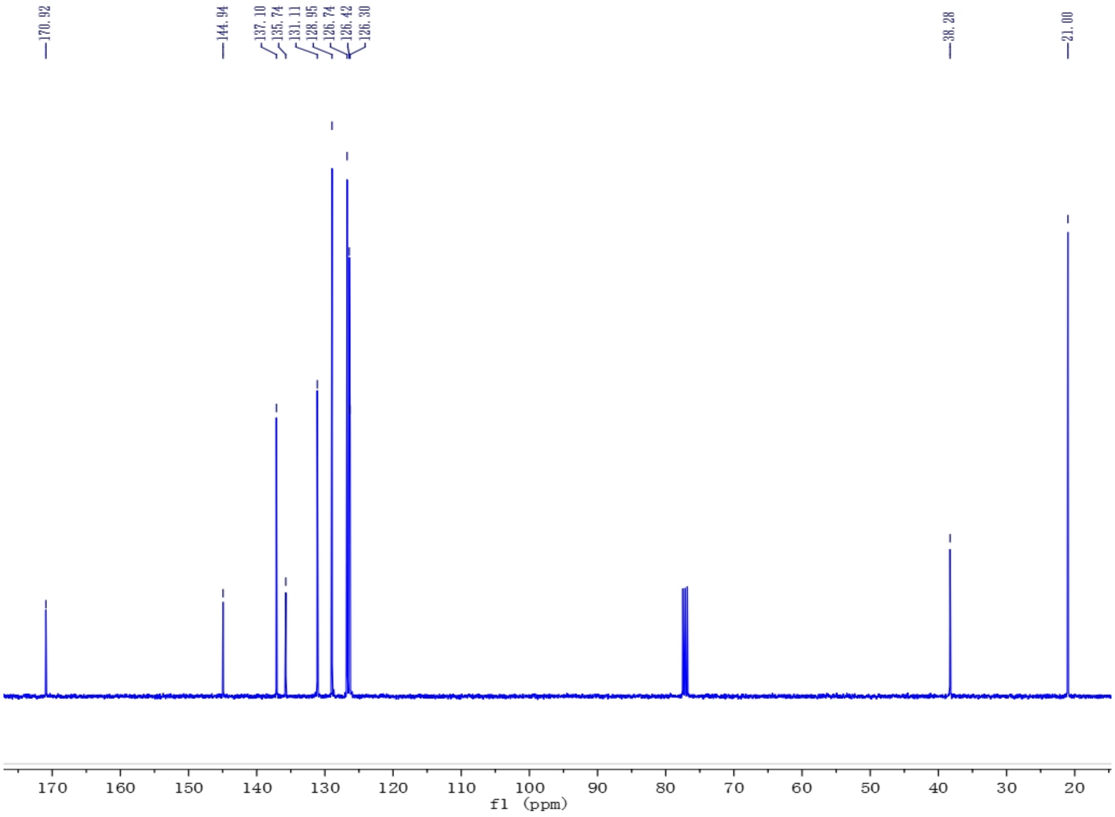

N,2,4-trimethyl-N-phenylbenzamide

^{1}H NMR (400 MHz, CDCl$_3$) δ 7.22 – 7.06 (m, 2H), 7.06 – 6.90 (m, 3H), 6.88 – 6.58 (m, 3H), 3.38 (s, 3H), 2.22 (s, 3H), 2.12 (s, 3H). **^{13}C NMR (101 MHz, CDCl$_3$)** δ 171.43, 144.02, 138.44, 134.68, 133.74, 130.95, 128.90, 127.46, 126.46, 125.83, 115.40, 37.56, 21.16, 19.47. **HRMS (ESI-TOF)**: m/z calculated for $C_{16}H_{17}NO$ $[M+H]^+$: 240.1383. Found: 240.1383.

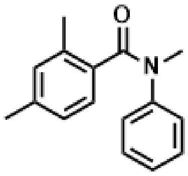

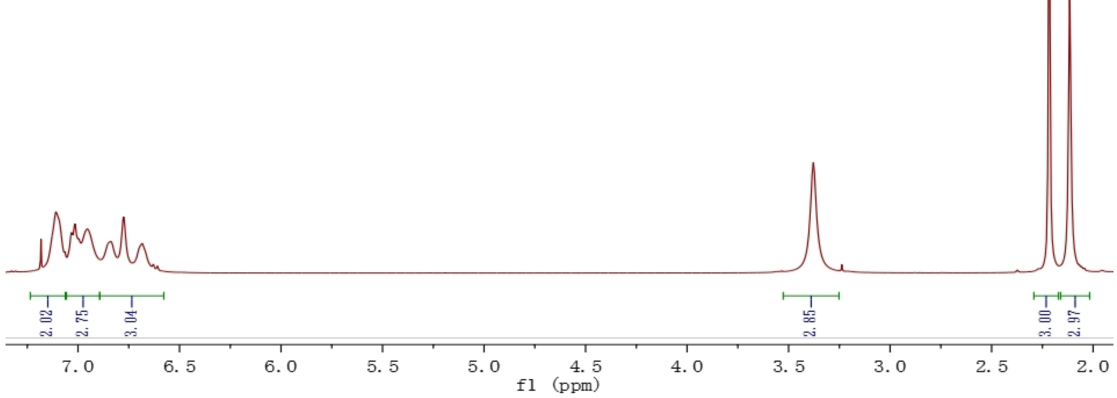

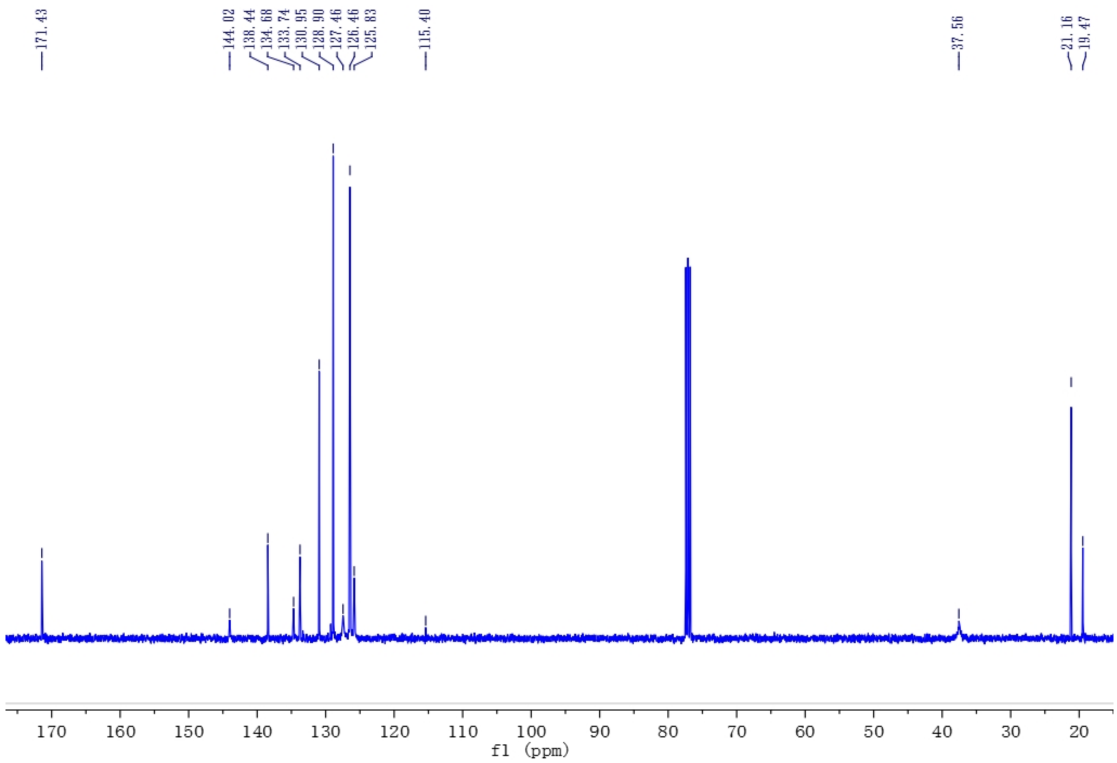

N-methyl-N-phenylfuran-2-carboxamide

^{1}H NMR (400 MHz, CDCl3) δ 7.46 – 7.26 (m, 4H), 7.19 (d, J = 7.2 Hz, 2H), 6.18 (d, J = 1.6 Hz, 1H), 5.81 (d, J = 2.9 Hz, 1H), 3.42 (s, 3H). **^{13}C NMR (101 MHz, CDCl3)** δ 159.36, 147.06, 144.22, 144.14, 129.60, 127.84, 127.38, 116.29, 110.91, 38.44. **HRMS (ESI-TOF)**: m/z calculated for $C_{12}H_{11}NO_2$ $[M+H]^+$: 202.0863. Found: 202.0863.

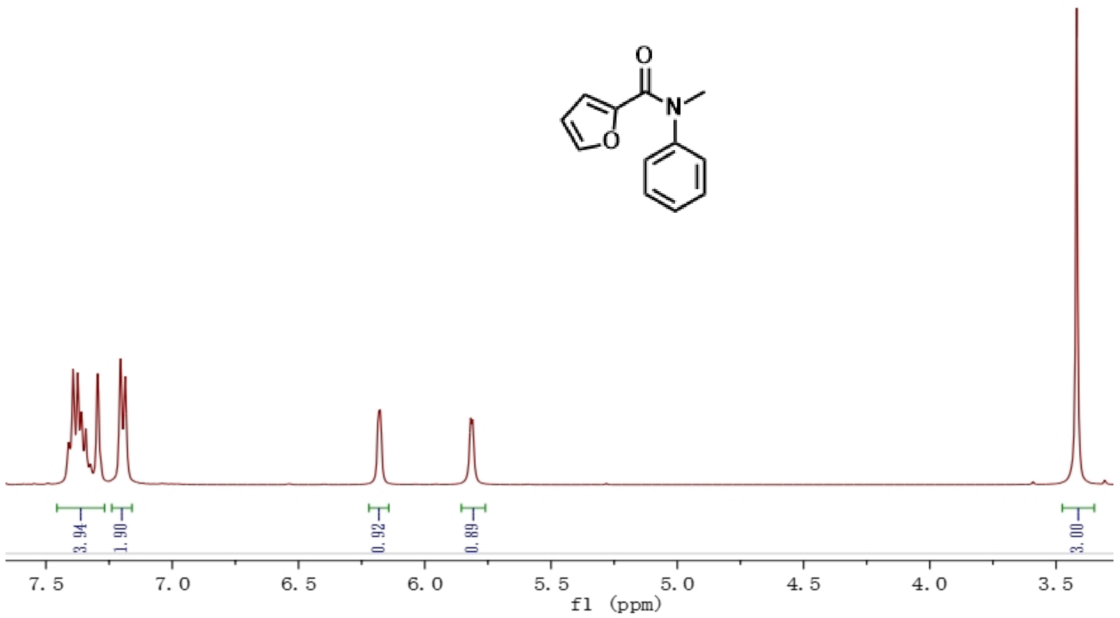

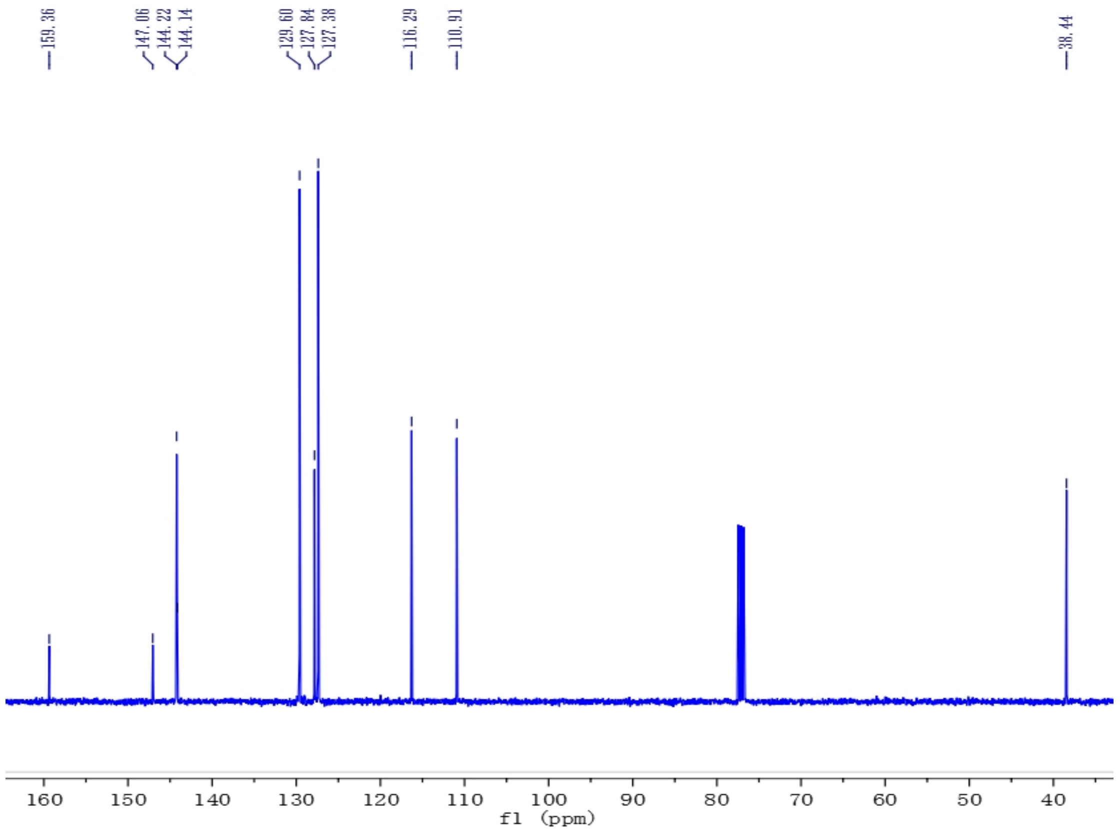

N-methyl-N-phenylthiophene-2-carboxamide

^{1}H NMR (400 MHz, CDCl3) δ 7.36 – 7.25 (m, 3H), 7.22 – 7.12 (m, 3H), 6.71 – 6.65 (m, 1H), 6.64 (dd, J = 3.7, 0.8 Hz, 1H), 3.36 (s, 3H). **^{13}C NMR (101 MHz, CDCl3)** δ 162.72, 144.28, 138.20, 132.06, 130.48, 128.14, 128.05, 126.64, 39.05. **HRMS (ESI-TOF)**: m/z calculated for $C_{12}H_{11}NOS$ $[M+H]^{+}$: 218.0634. Found: 218.0634.

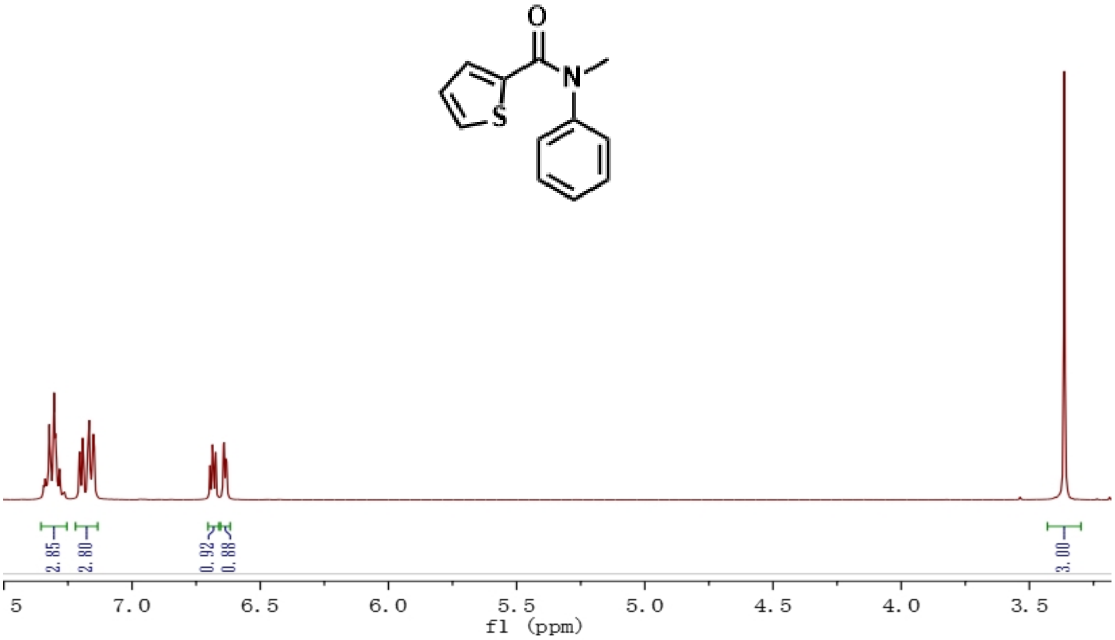

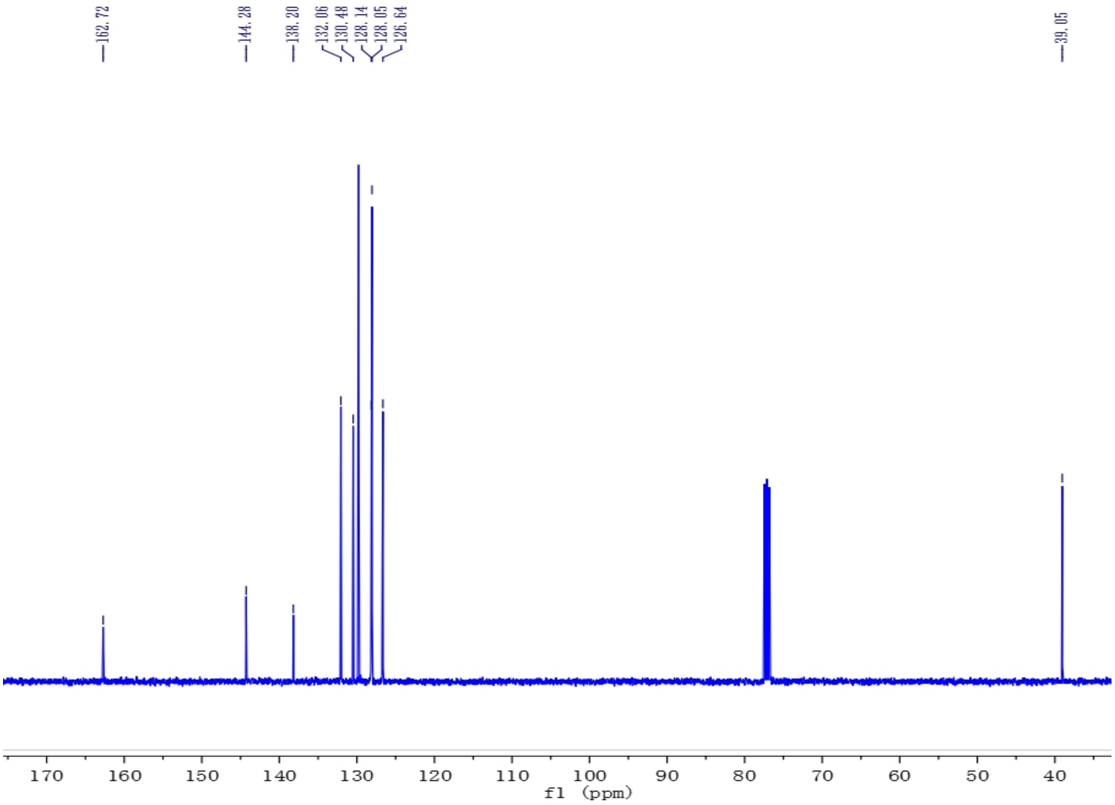

N-methyl-N-phenyl-2-naphthamide

^{1}H NMR (400 MHz, CDCl3) δ 7.86 – 7.79 (m, 1H), 7.69 – 7.60 (m, 2H), 7.55 – 7.48 (m, 1H), 7.42 – 7.32 (m, 2H), 7.28 – 7.22 (m, 1H), 7.16 – 7.08 (m, 2H), 7.07 – 6.99 (m, 3H), 3.49 (s, 3H). **^{13}C NMR (101 MHz, CDCl3)** δ 170.52, 144.96, 133.56, 133.29, 132.42, 129.42, 129.23, 128.63, 127.58, 127.23, 127.11, 126.93, 126.52, 126.30, 125.51, 38.58. **HRMS (ESI-TOF)**: m/z calculated for $C_{18}H_{15}NO$ $[M+H]^{+}$: 262.1226. Found: 262.1223.

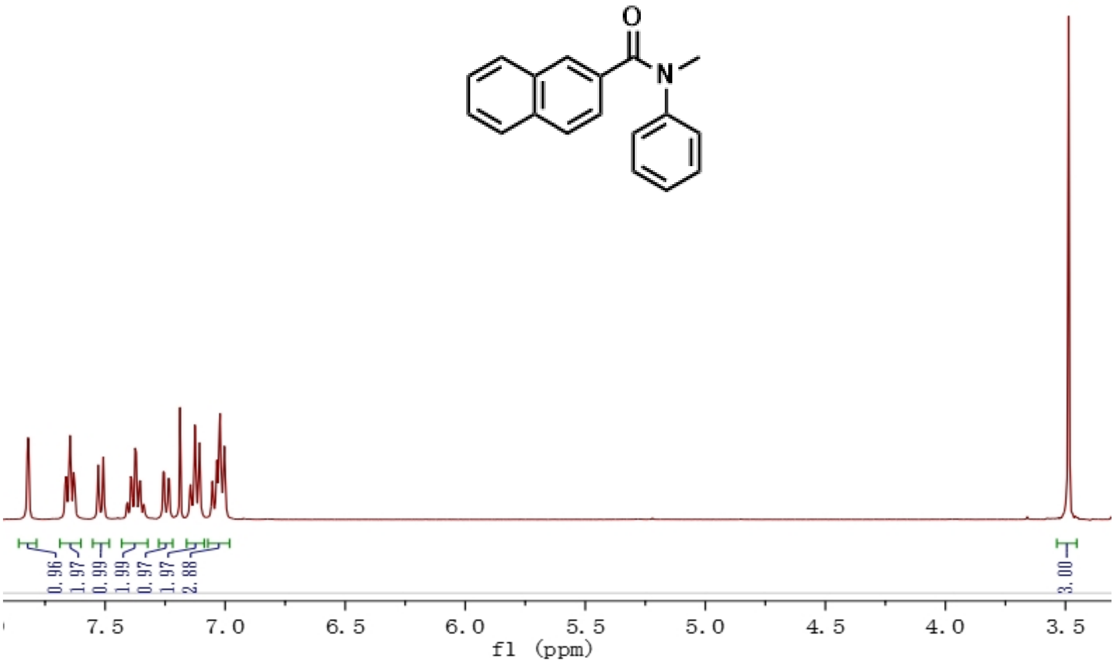

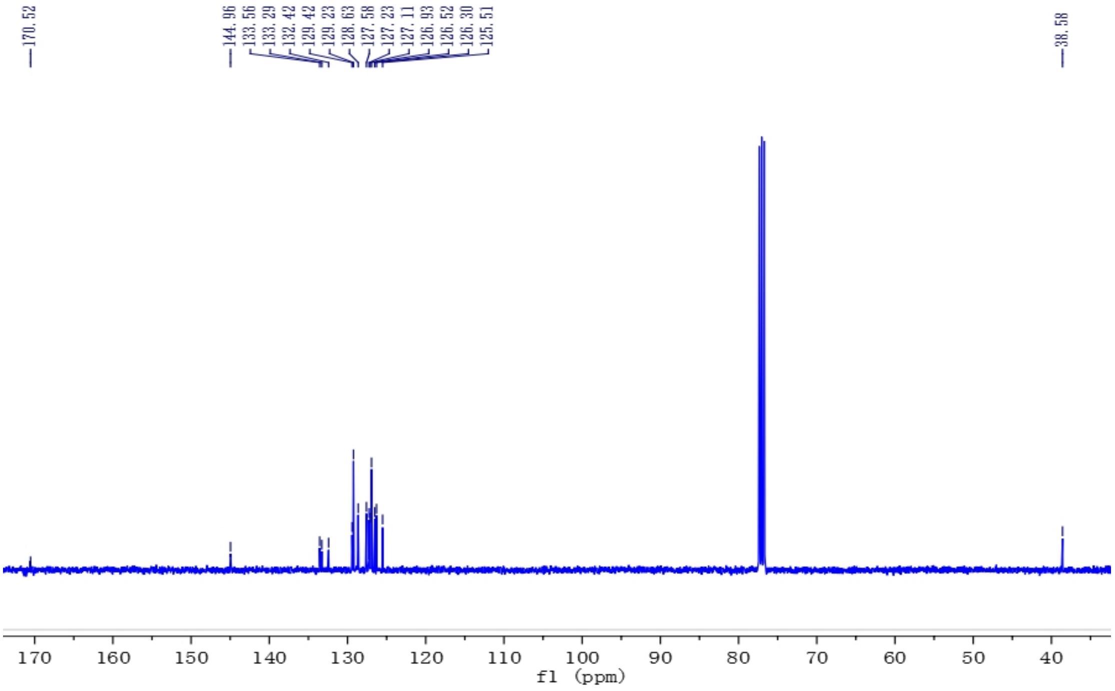

N-methyl-N-phenylacrylamide

^{1}H NMR (400 MHz, CDCl3) δ 7.37 – 7.31 (m, 2H), 7.30 – 7.24 (m, 1H), 7.14 – 7.09 (m, 2H), 6.34 – 6.26 (m, 1H), 6.06 – 5.92 (m, 1H), 5.48 – 5.40 (m, 1H), 3.29 (s, 3H). **^{13}C NMR (101 MHz, CDCl3)** δ 165.78, 143.46, 129.60, 128.53, 127.61, 127.39, 127.31, 37.43. **HRMS (ESI-TOF)**: m/z calculated for $C_{10}H_{11}NO$ $[M+H]^{+}$:162.0913. Found: 162.0913.

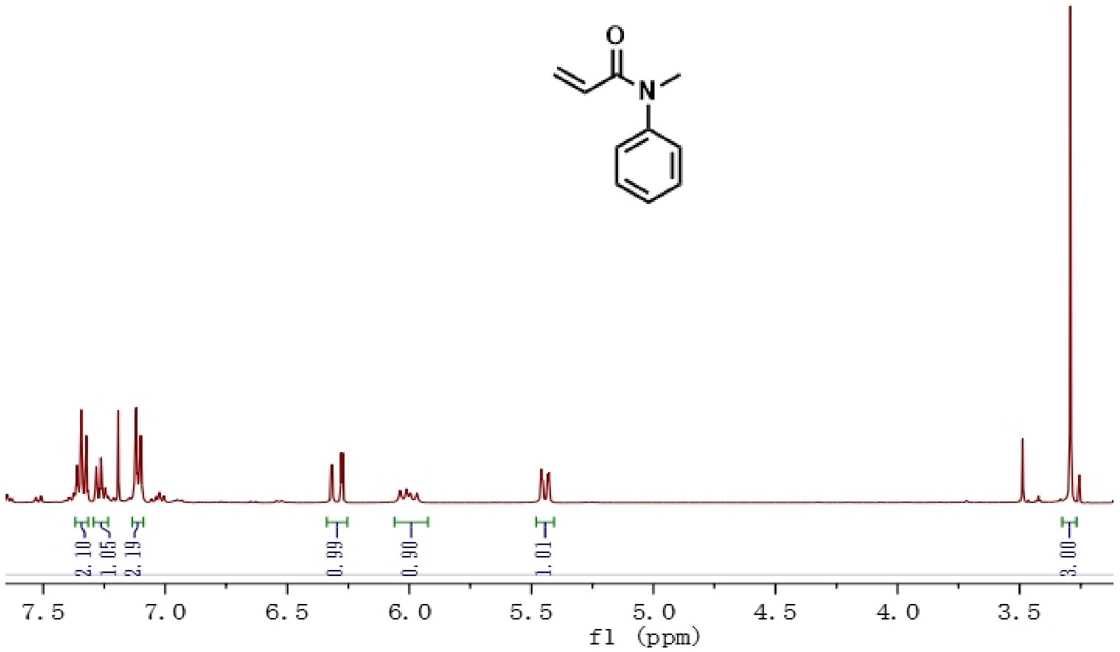

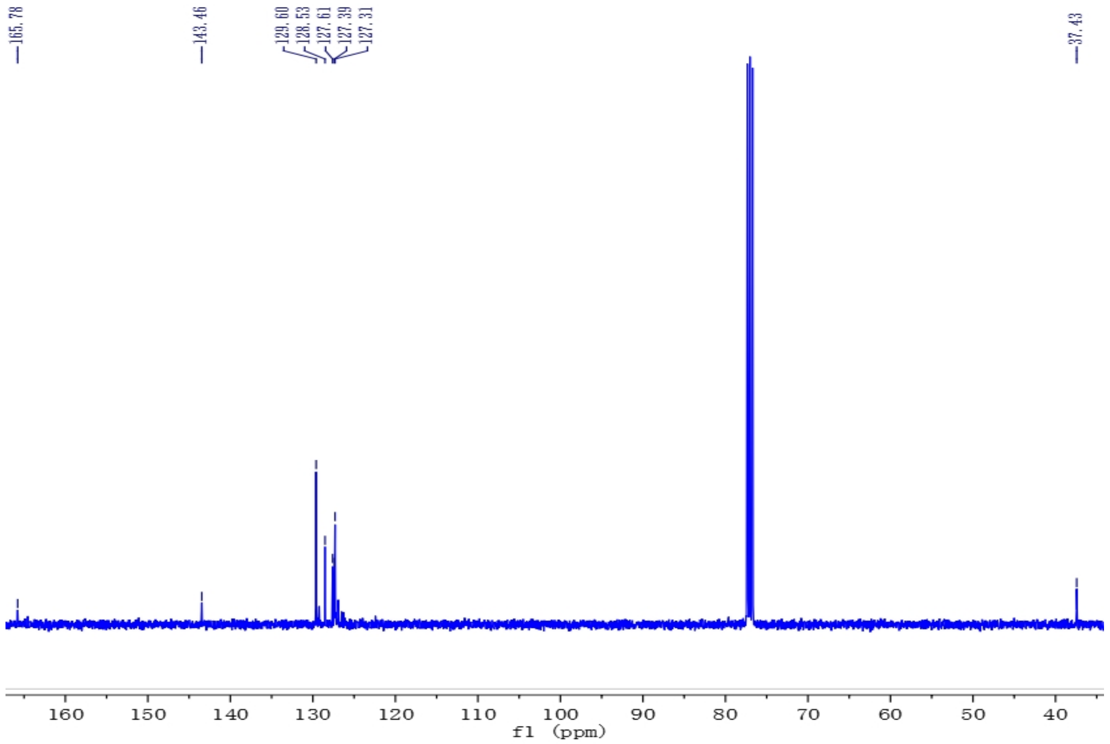

(E)-N-methyl-N-phenylbut-2-enamide

^{1}H NMR (400 MHz, CDCl3) δ 7.37 – 7.30 (m, 2H), 7.25 (s, 1H), 7.12 – 7.07 (m, 2H), 6.90 – 6.79 (m, 1H), 5.72 – 5.64 (m, 1H), 3.26 (s, 3H), 1.65 (dd, J = 6.9, 1.6 Hz, 3H). **^{13}C NMR (101 MHz, CDCl3)** δ 166.19, 143.76, 141.20, 129.52, 127.42, 127.37, 122.73, 37.38, 17.94. **HRMS (ESI-TOF)**: m/z calculated for $C_{11}H_{13}NO$ $[M+H]^{+}$:176.1070. Found: 176.1072.

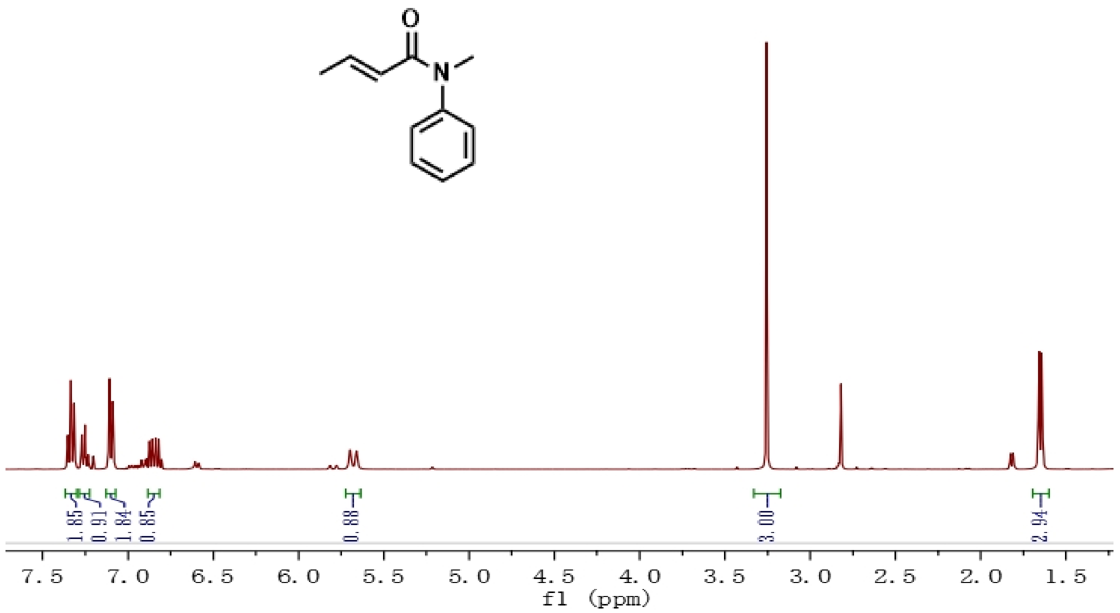

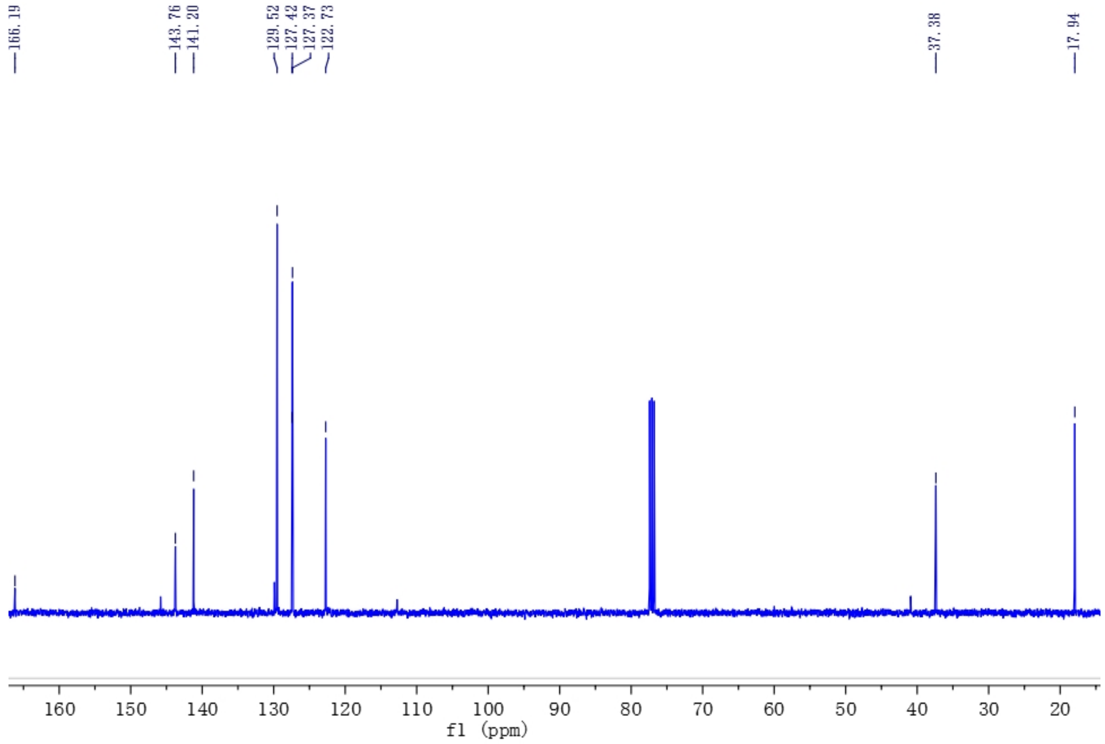

N-methyl-N-phenylcinnamamide

^{1}H NMR (400 MHz, CDCl3) δ 7.58 (s, 1H), 7.35 (d, J = 7.8 Hz, 2H), 7.31 – 7.25 (m, 1H), 7.25 – 7.12 (m, 7H), 6.33 – 6.25 (m, 1H), 3.33 (s, 3H). **^{13}C NMR (101 MHz, CDCl3)** δ 166.17, 143.67, 141.70, 135.23, 129.65, 129.50, 128.69, 127.85, 127.60, 127.35, 118.80, 37.58. **HRMS (ESI-TOF)**: m/z calculated for $C_{16}H_{15}NO$ [M+H]$^{+}$:238.1226. Found: 238.1227.

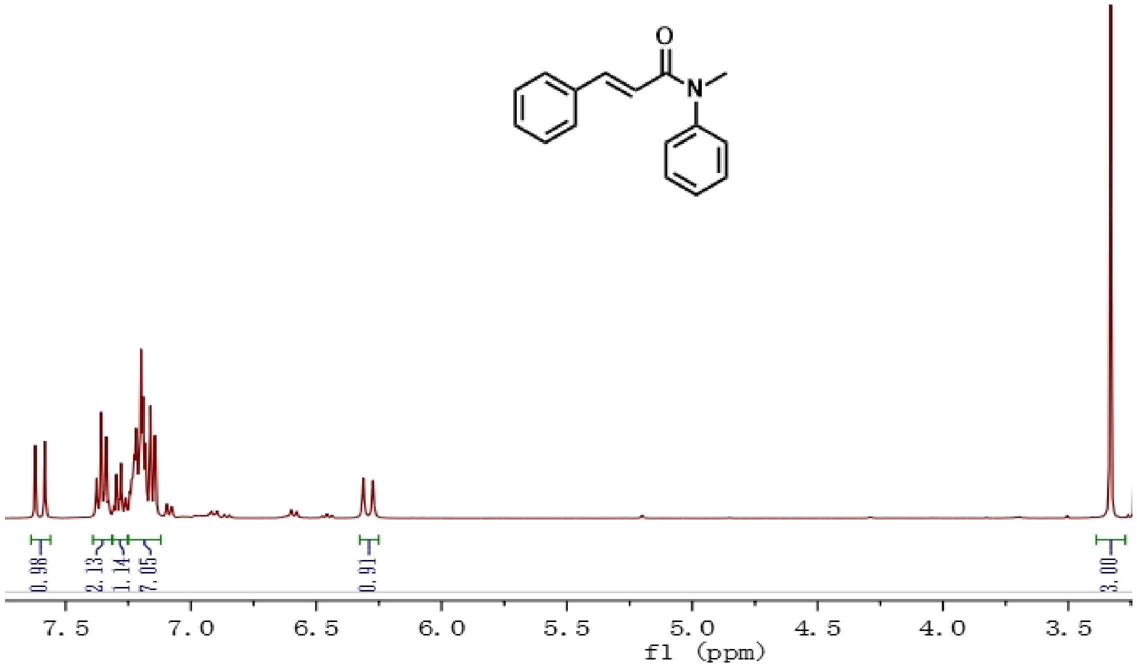

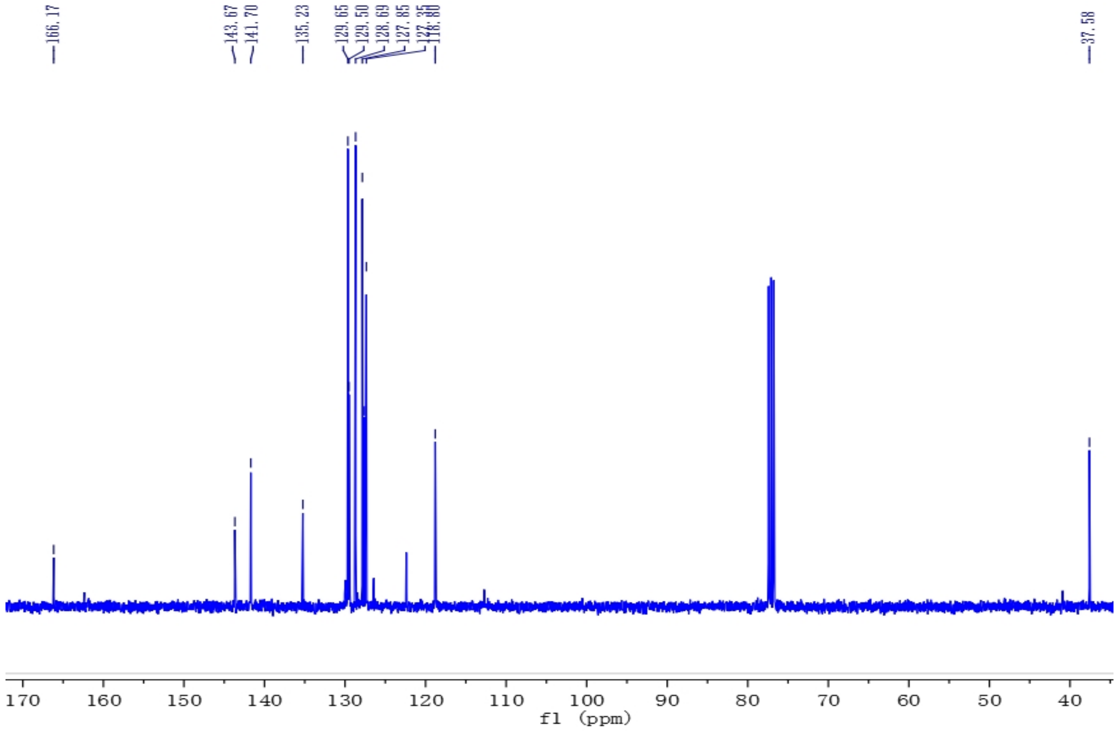

N-methyl-N-phenylbenzenesulfonamide

^{1}H NMR (400 MHz, CDCl3) δ 7.53 – 7.42 (m, 1H), 7.38 (d, J = 7.4 Hz, 1H), 7.20 (t, J = 4.8 Hz, 1H), 7.01 (dd, J = 8.1, 1.5 Hz, 1H), 3.10 (s, 1H). **^{13}C NMR (101 MHz, CDCl3)** δ 170.66, 142.34, 136.28, 136.11, 129.77, 129.47, 128.68, 127.70, 126.68, 38.47, 20.94. **HRMS (ESI-TOF)**: m/z calculated for $C_{13}H_{13}NO_2S$ $[M+H]^+$:248.0740. Found: 248.0740.

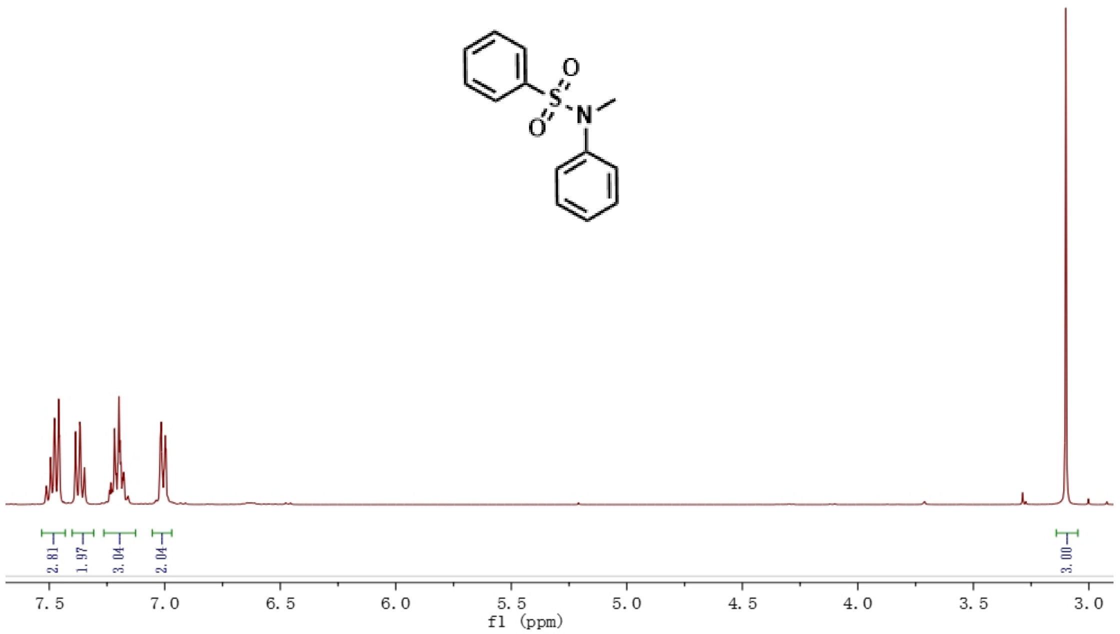

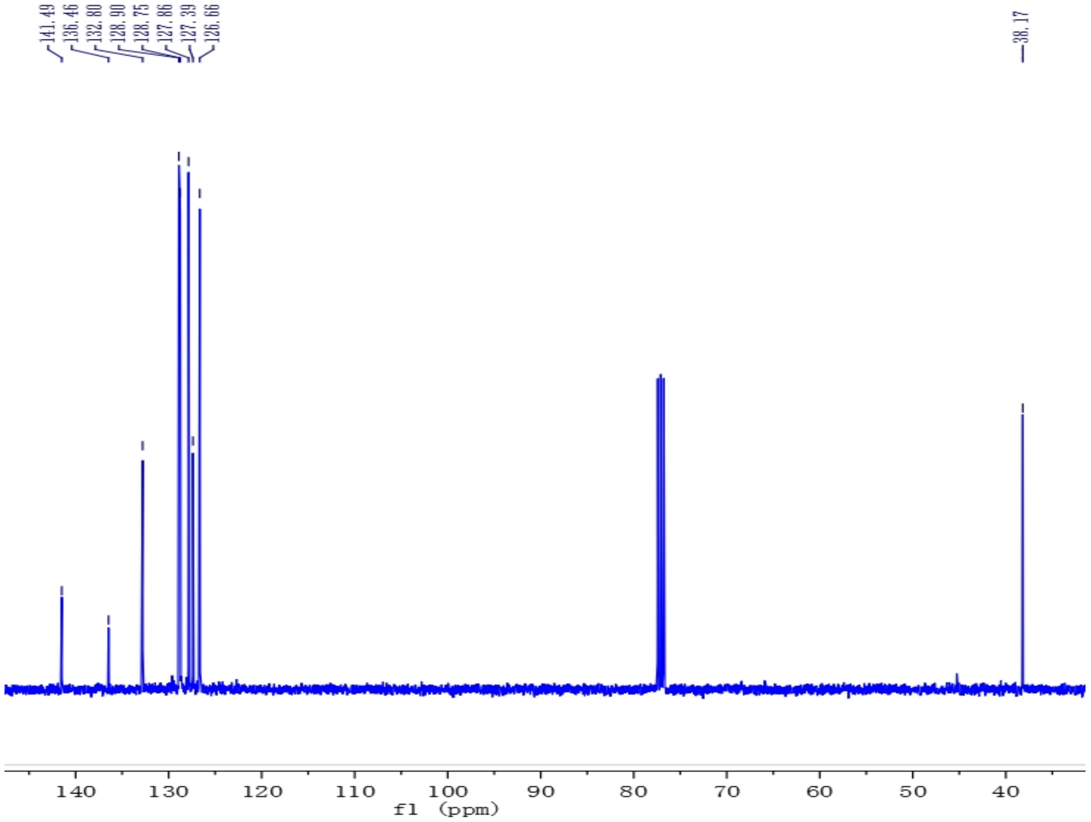

N,4-dimethyl-N-phenylbenzenesulfonamide

^{1}H NMR (400 MHz, CDCl3) δ 7.34 (d, J = 8.3 Hz, 2H), 7.18 (ddd, J = 17.4, 8.9, 7.1 Hz, 5H), 7.01 (dd, J = 8.2, 1.3 Hz, 2H), 3.08 (s, 3H), 2.33 (s, 3H). **^{13}C NMR (101 MHz, CDCl3)** δ 143.58, 141.64, 133.50, 129.36, 128.85, 127.91, 127.28, 126.63, 38.10, 21.56. **HRMS (ESI-TOF)**: m/z calculated for $C_{14}H_{15}NO_2S$ $[M+H]^+$:262.0896. Found: 262.0896.

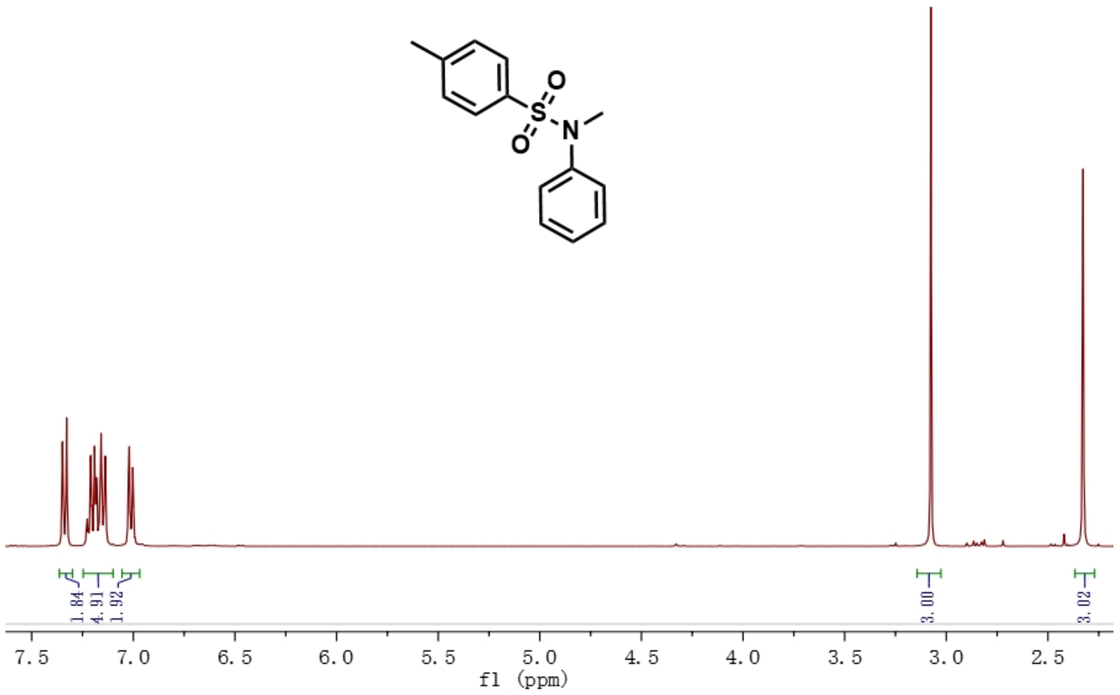

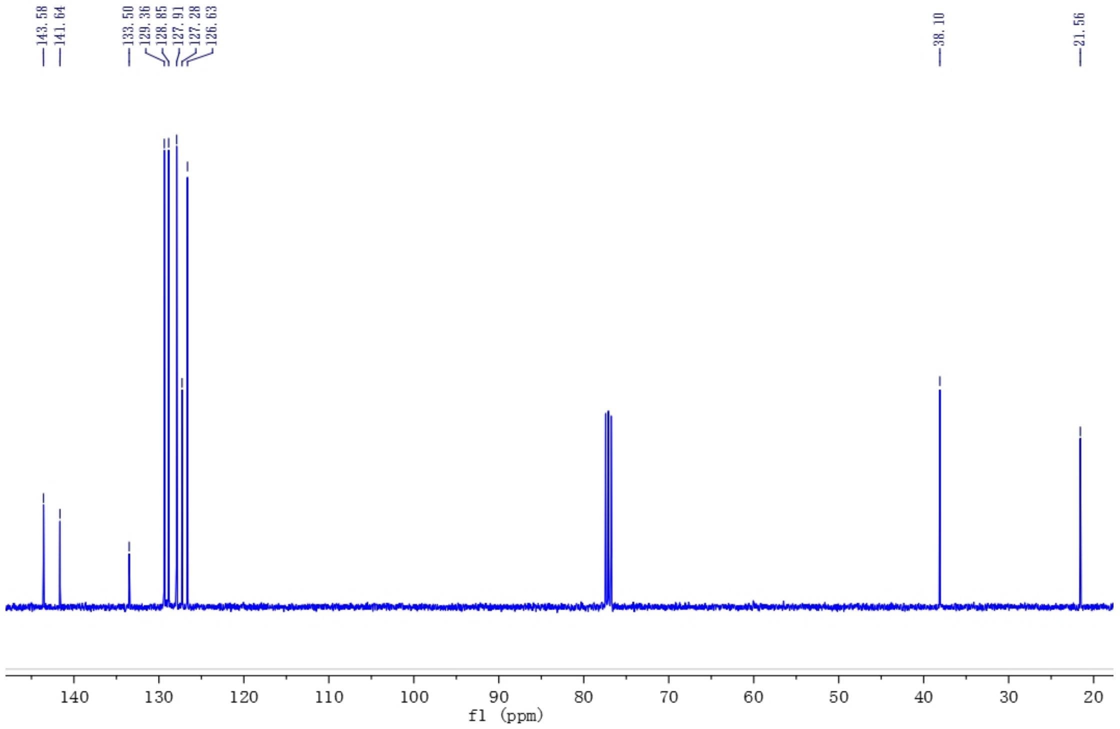

4-Methoxy-N-methyl-N-phenylbenzenesulfonamide

^{1}H NMR (400 MHz, CDCl3) δ 7.50 (d, J = 8.9 Hz, 2H), 7.35 – 7.23 (m, 3H), 7.18 – 7.07 (m, 2H), 6.94 (d, J = 8.9 Hz, 2H), 3.88 (s, 3H), 3.19 (s, 3H). **^{13}C NMR (101 MHz, CDCl3)** δ 163.00, 141.72, 129.98, 128.84, 128.11, 127.25, 126.65, 113.89, 55.60, 38.05. **HRMS (ESI-TOF)**: m/z calculated for $C_{14}H_{15}NO_3S$ $[M+H]^+$:278.0845. Found: 278.0845.

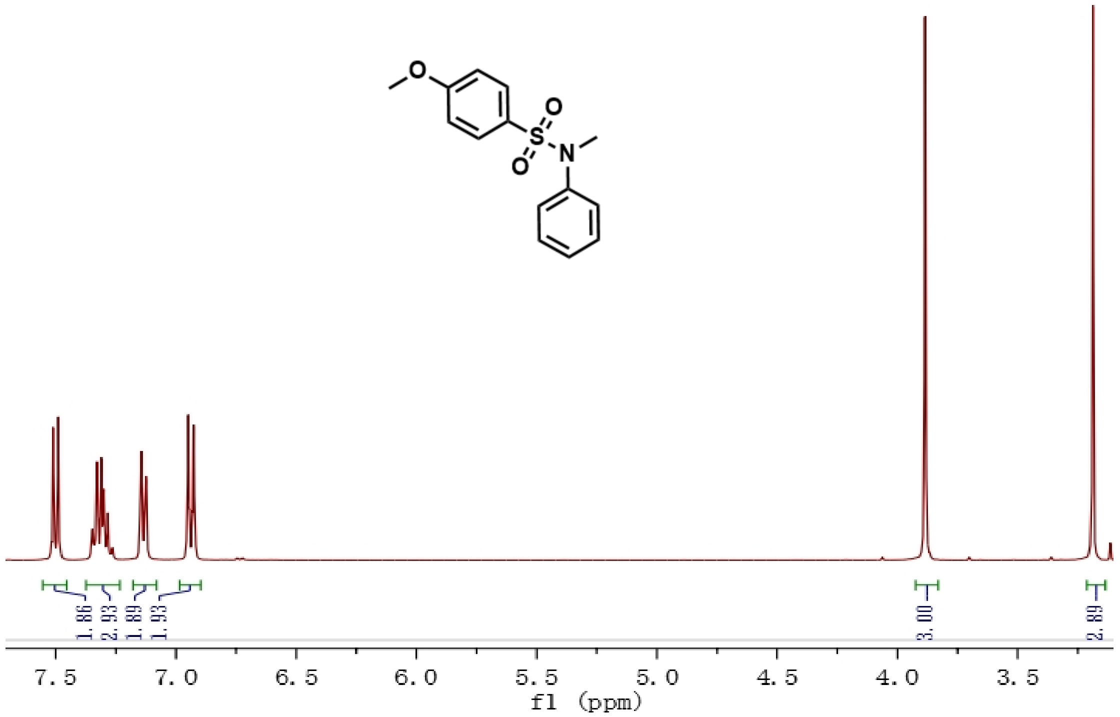

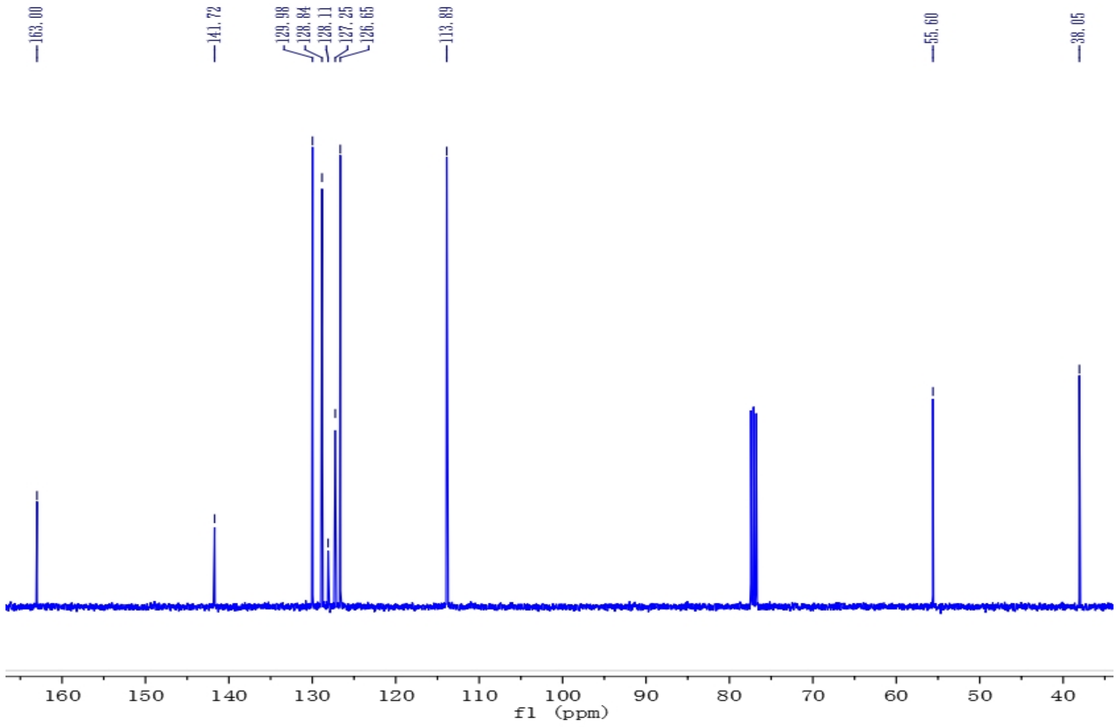

N-methyl-N-(o-tolyl)benzamide

^{1}H NMR (400 MHz, $CDCl_3$) δ 7.19 (d, J = 7.3 Hz, 2H), 7.13 – 7.06 (m, 1H), 7.06 – 6.91 (m, 6H), 3.29 (s, 3H), 2.11 (s, 3H). **^{13}C NMR (101 MHz, $CDCl_3$)** δ 170.77, 143.49, 135.89, 134.73, 131.31, 129.63, 128.62, 128.27, 127.72, 127.54, 126.97, 37.52, 17.72. **HRMS (ESI-TOF)**: m/z calculated for $C_{15}H_{15}NO$ $[M+H]^+$: 226.1266. Found: 226.1266.

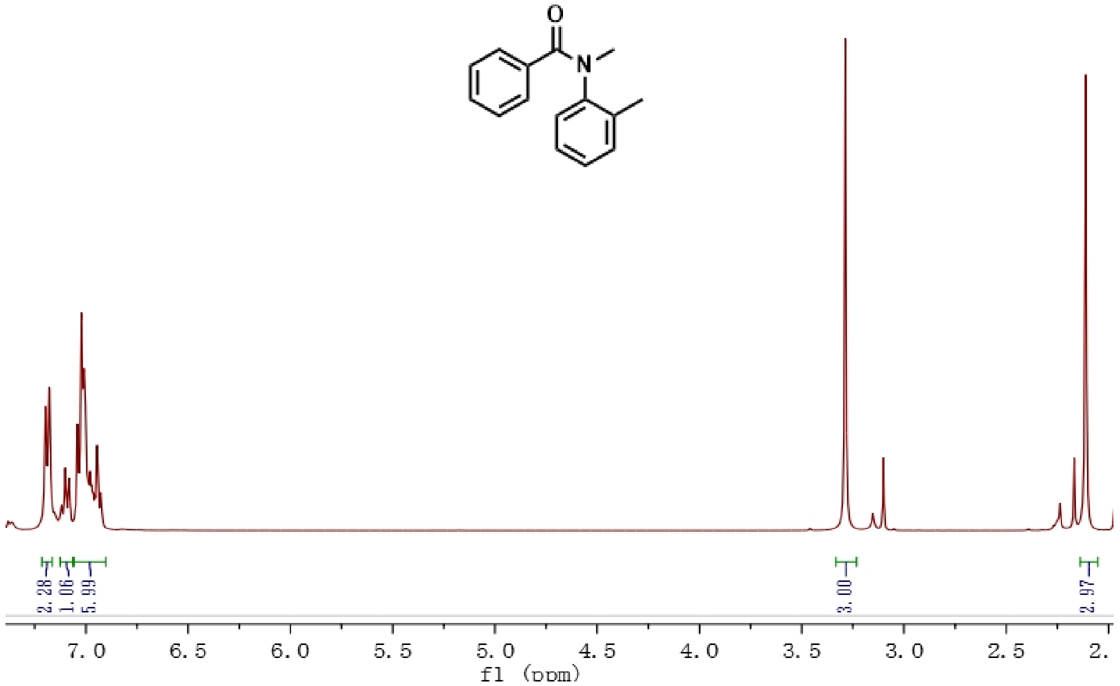

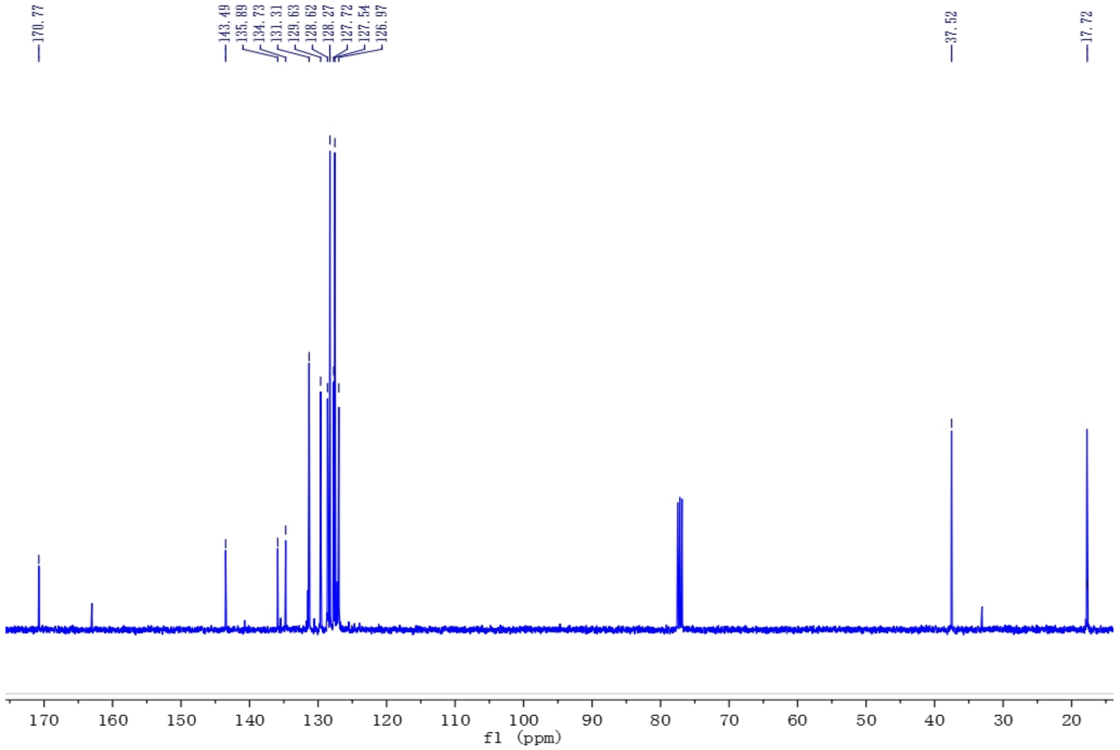

N-(2-chlorophenyl)-N-methylbenzamide

^{1}H NMR (400 MHz, CDCl3) δ 7.33 (s, 3H), 7.25 – 7.18 (m, 1H), 7.14 (s, 5H), 3.39 (s, 3H). **^{13}C NMR (101 MHz, CDCl3)** δ 171.17, 142.19, 135.71, 132.23, 130.50, 129.82, 128.85, 127.96, 127.77, 127.70, 36.95. **HRMS (ESI-TOF)**: m/z calculated for $C_{14}H_{12}ClNO$ $[M+Na]^{+}$: 268.0500. Found: 268.0498.

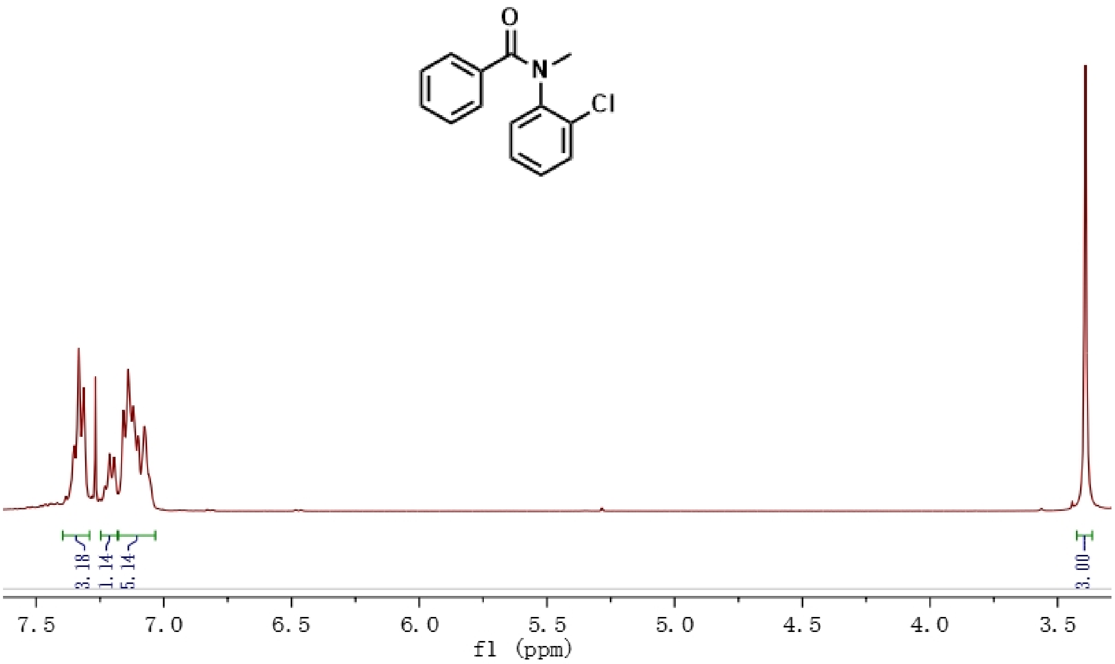

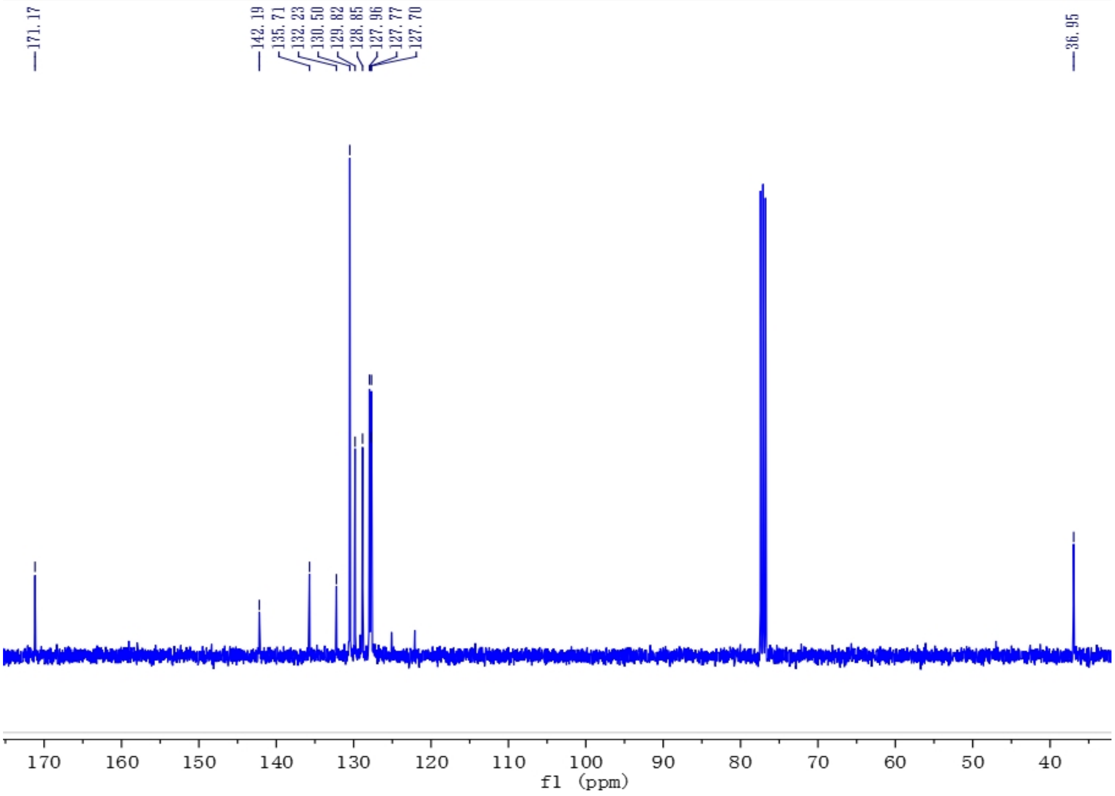

N-(2-bromophenyl)-N-methylbenzamide

^{1}H NMR (400 MHz, CDCl3) δ 7.57 – 7.49 (m, 1H), 7.37 – 7.30 (m, 2H), 7.24 – 7.11 (m, 4H), 7.10 – 6.97 (m, 2H), 3.39 (s, 3H). **^{13}C NMR (101 MHz, CDCl3)** δ 171.02, 162.91, 143.68, 135.71, 133.73, 130.70, 129.81, 129.05, 128.45, 128.08, 127.68, 122.75, 37.13. **HRMS (ESI-TOF)**: m/z calculated for $C_{14}H_{12}BrNO$ [M+H]$^{+}$: 290.0175. Found: 290.0171.

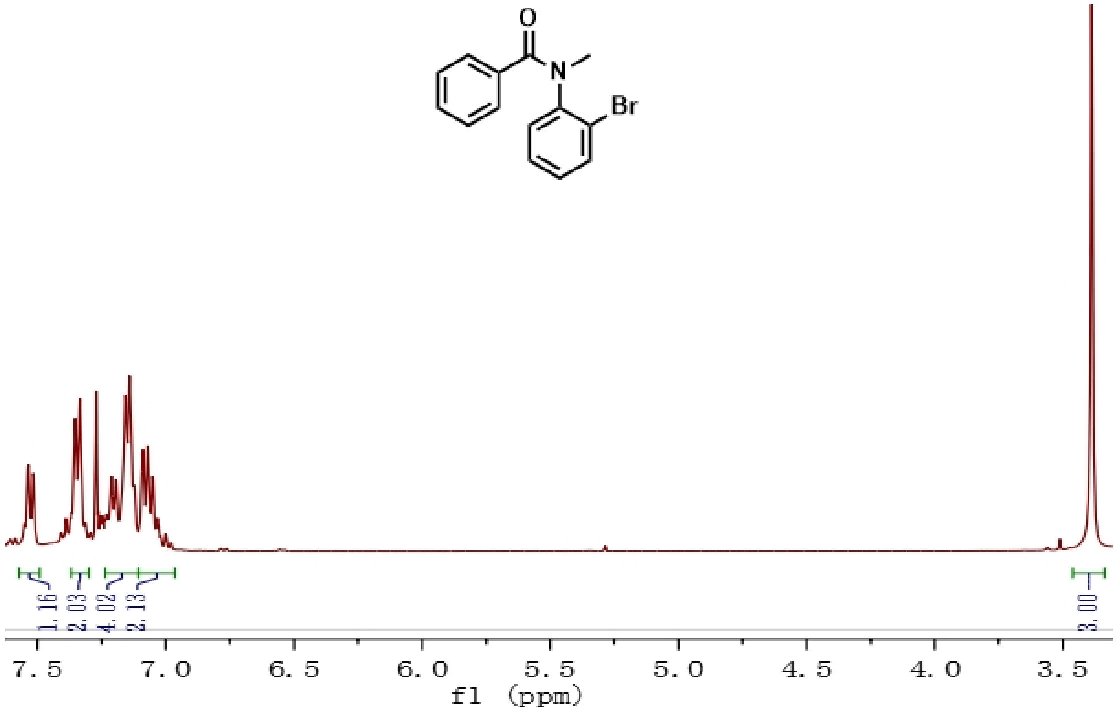

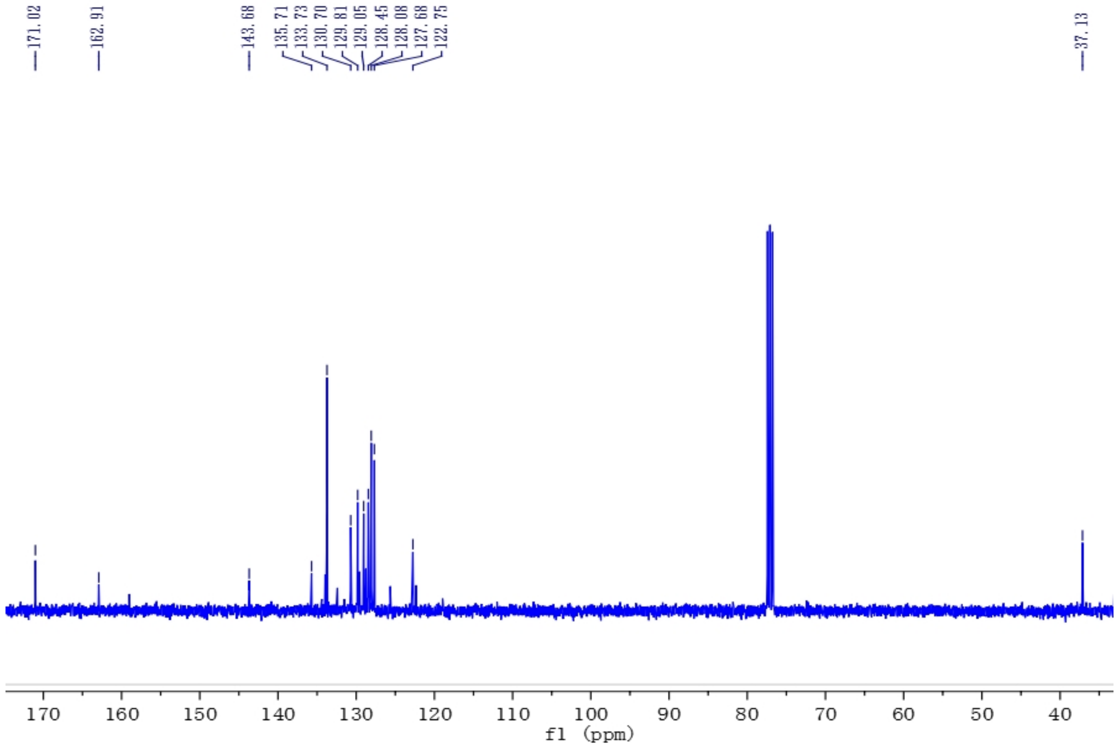

N-methyl-N-(m-tolyl)benzamide

^{1}H NMR (400 MHz, CDCl3) δ 7.33 – 7.24 (m, 2H), 7.24 – 7.09 (m, 3H), 7.05 (t, J = 7.7 Hz, 1H), 6.91 (dd, J = 20.7, 13.1 Hz, 2H), 6.79 (d, J = 7.9 Hz, 1H), 3.46 (s, 3H), 2.22 (s, 3H). **^{13}C NMR (101 MHz, CDCl3)** δ 170.61, 144.80, 139.08, 136.05, 129.52, 128.86, 128.64, 127.67, 127.37, 127.26, 124.04, 38.40, 21.20. **HRMS (ESI-TOF)**: m/z calculated for $C_{15}H_{15}NO$ $[M+H]^{+}$: 226.1266. Found: 226.1267.

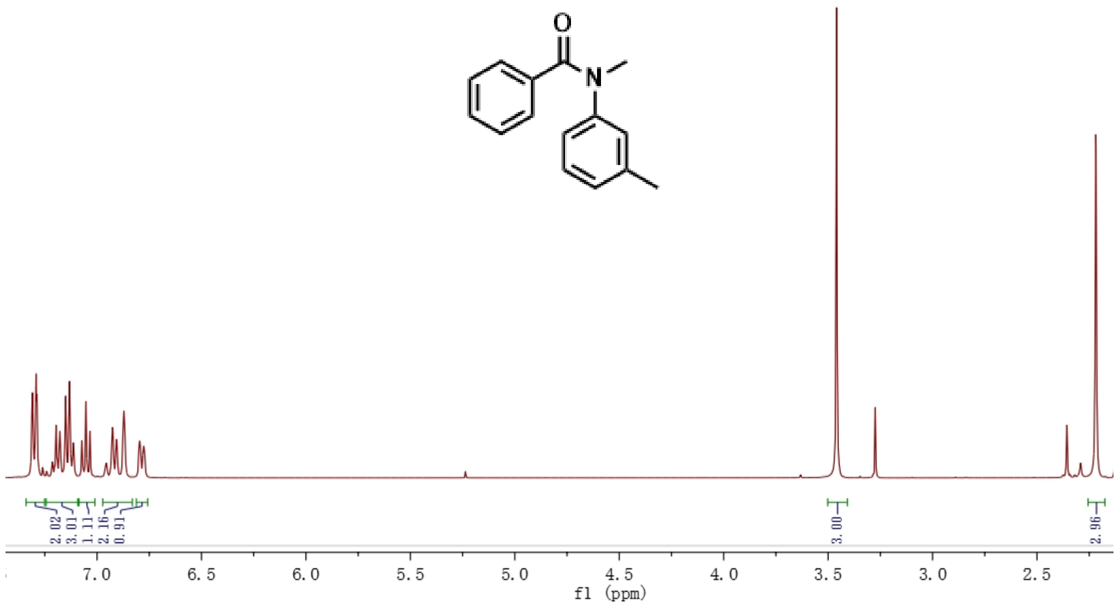

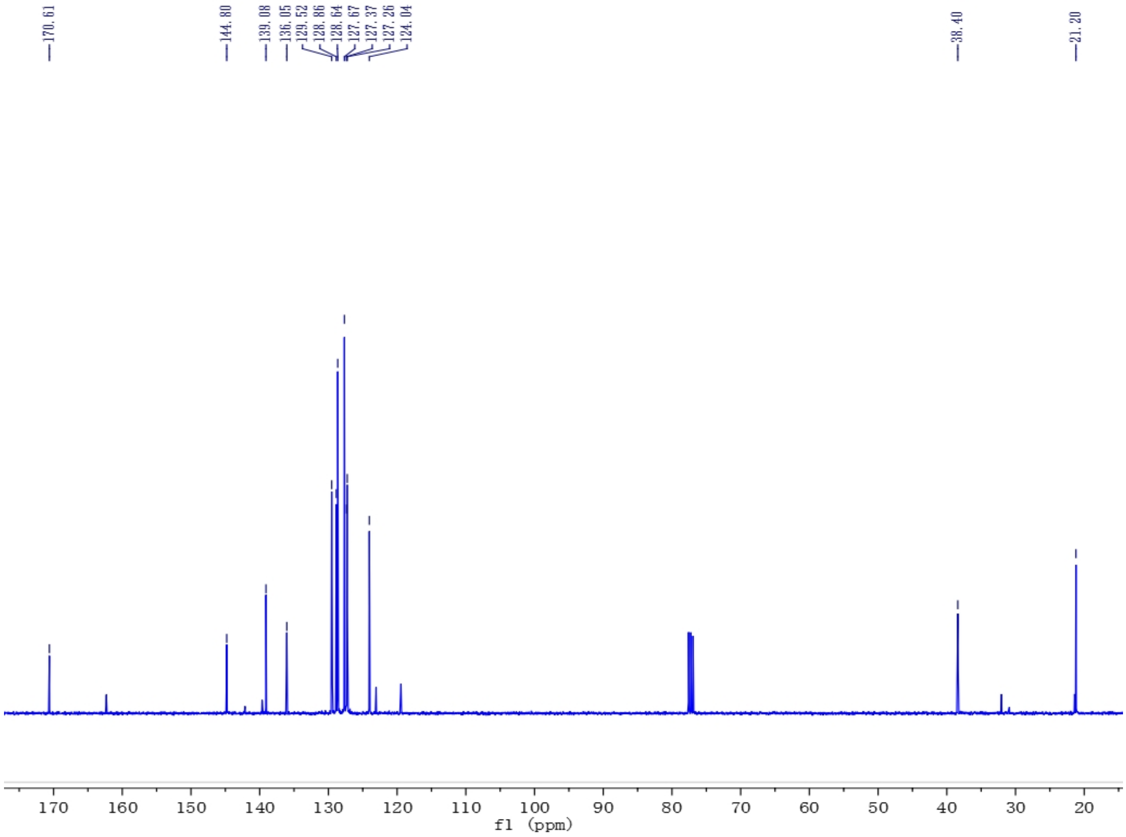

N-(3-bromophenyl)-N-methylbenzamide

^{1}H NMR (400 MHz, CDCl3) δ 7.35 – 7.24 (m, 5H), 7.21 (d, J = 7.6 Hz, 2H), 7.05 (s, 1H), 6.95 – 6.86 (m, 1H), 3.47 (s, 3H). **^{13}C NMR (101 MHz, CDCl3)** δ 170.57, 146.21, 135.44, 130.28, 129.97, 129.69, 129.55, 128.63, 127.96, 125.76, 122.43, 38.37. **HRMS (ESI-TOF)**: m/z calculated for $C_{14}H_{12}BrNO$ $[M+H]^{+}$: 290.0175. Found: 290.0171.

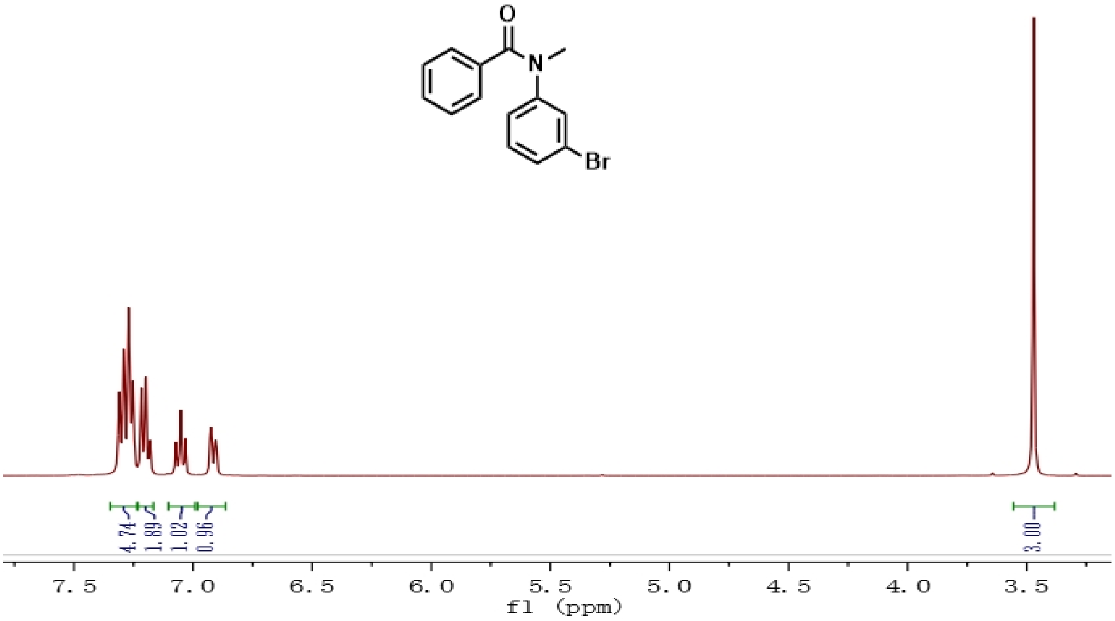

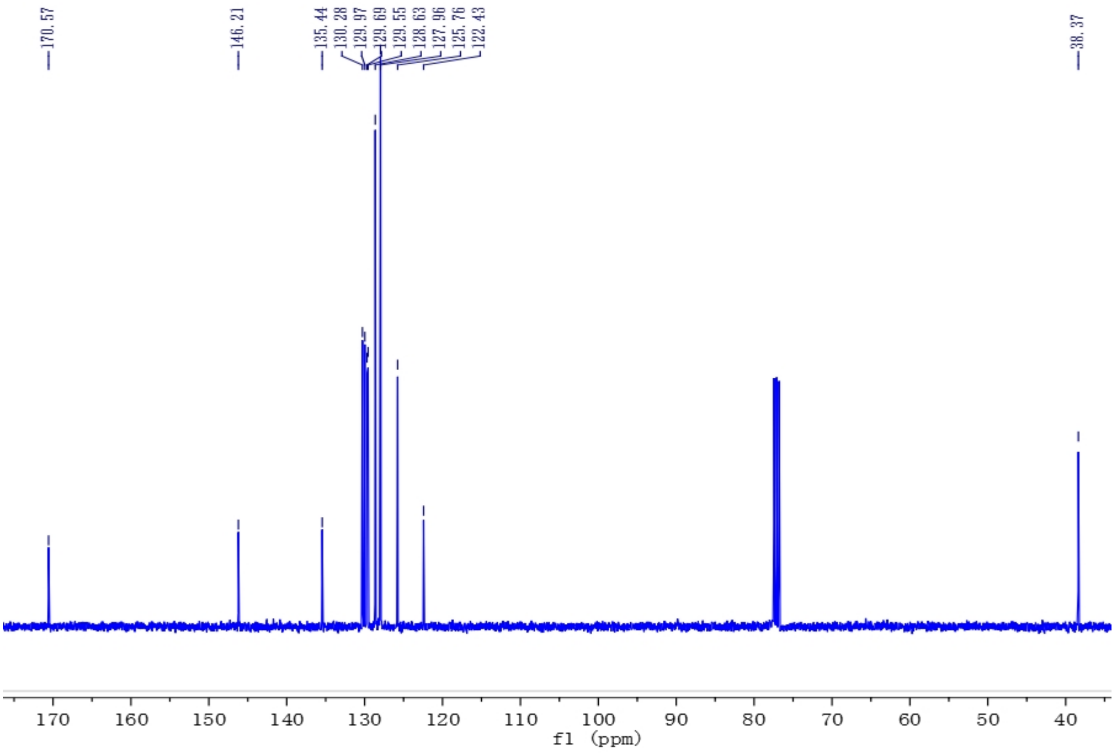

N-(3-methoxyphenyl)-N-methylbenzamide

^{1}H NMR (400 MHz, CDCl3) δ 7.36 – 7.29 (m, 2H), 7.23 (s, 1H), 7.17 (d, J = 7.6 Hz, 2H), 7.09 (d, J = 8.1 Hz, 1H), 6.71 – 6.64 (m, 1H), 6.64 – 6.59 (m, 1H), 6.57 (d, J = 2.1 Hz, 1H), 3.65 (s, 3H), 3.48 (s, 3H). **^{13}C NMR (101 MHz, CDCl3)** δ 170.64, 160.02, 146.01, 136.02, 129.77, 129.64, 128.55, 127.76, 119.20, 112.82, 112.09, 55.30, 38.33. **HRMS (ESI-TOF)**: m/z calculated for $C_{15}H_{15}NO_2$ [M+H]$^+$: 242.1176. Found: 242.1176.

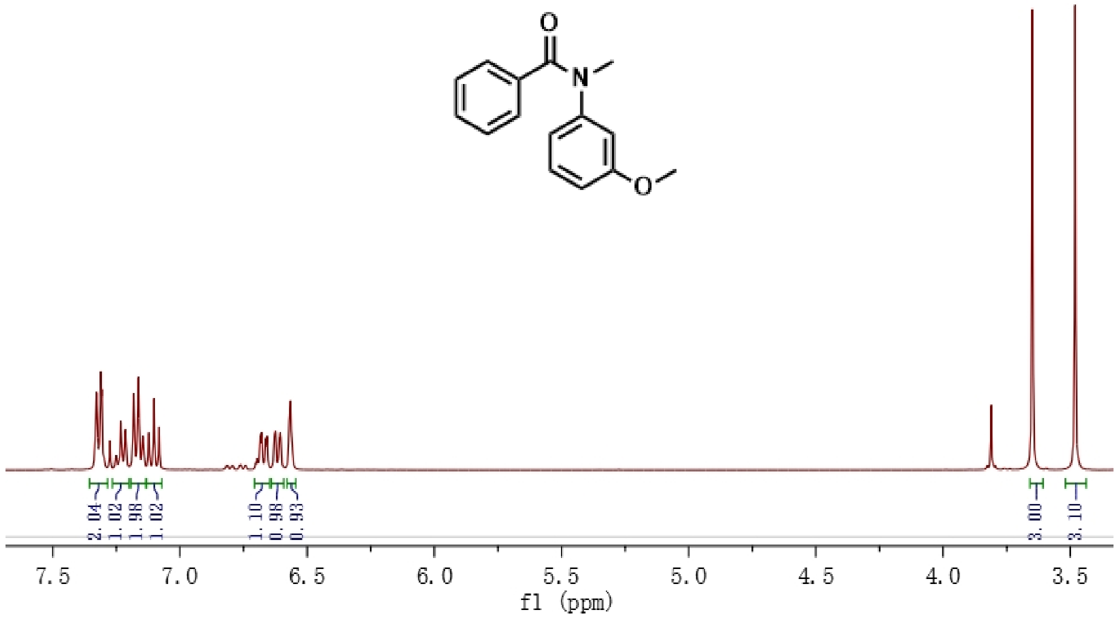

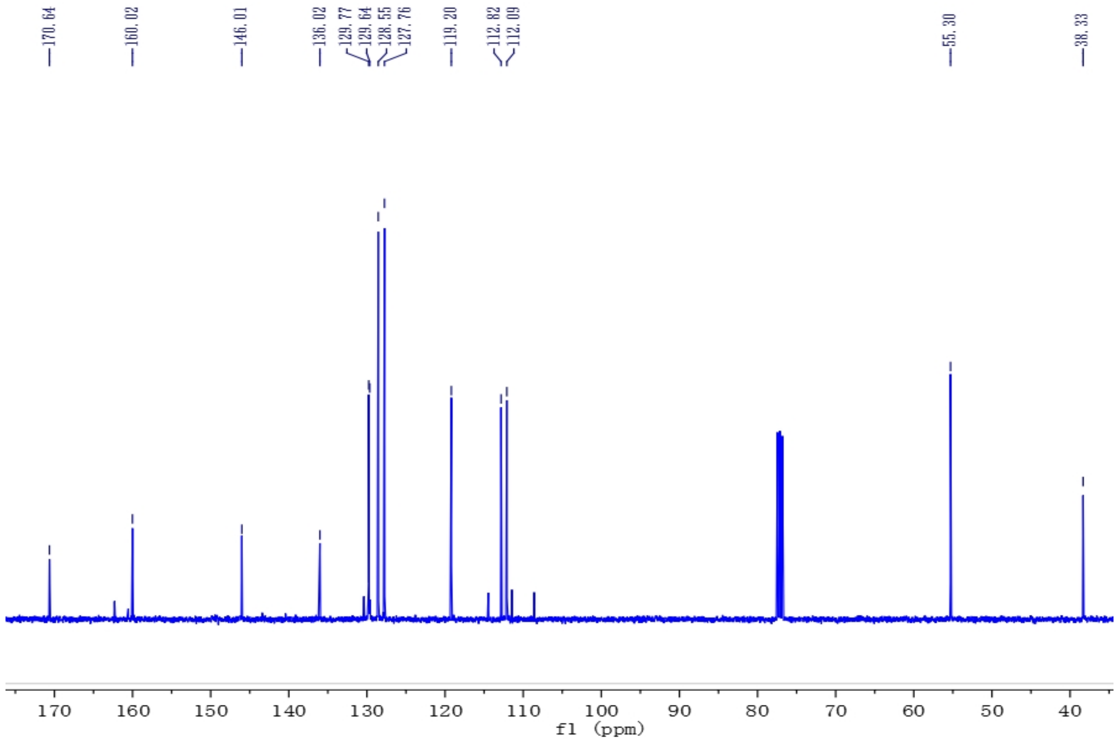

N-methyl-N-(p-tolyl)benzamide

^{1}H NMR (400 MHz, CDCl3) δ 7.25 – 7.16 (m, 2H), 7.08 (dt, J = 14.7, 7.1 Hz, 3H), 6.91 (d, J = 8.1 Hz, 2H), 6.82 (d, J = 8.2 Hz, 2H), 3.37 (s, 3H), 2.16 (s, 3H). **^{13}C NMR (101 MHz, CDCl3)** δ 170.66, 142.34, 136.28, 136.11, 129.77, 129.47, 128.68, 127.70, 126.68, 38.47, 20.94. **HRMS (ESI-TOF)**: m/z calculated for $C_{15}H_{15}NO$ $[M+H]^{+}$: 226.1266. Found: 226.1266.

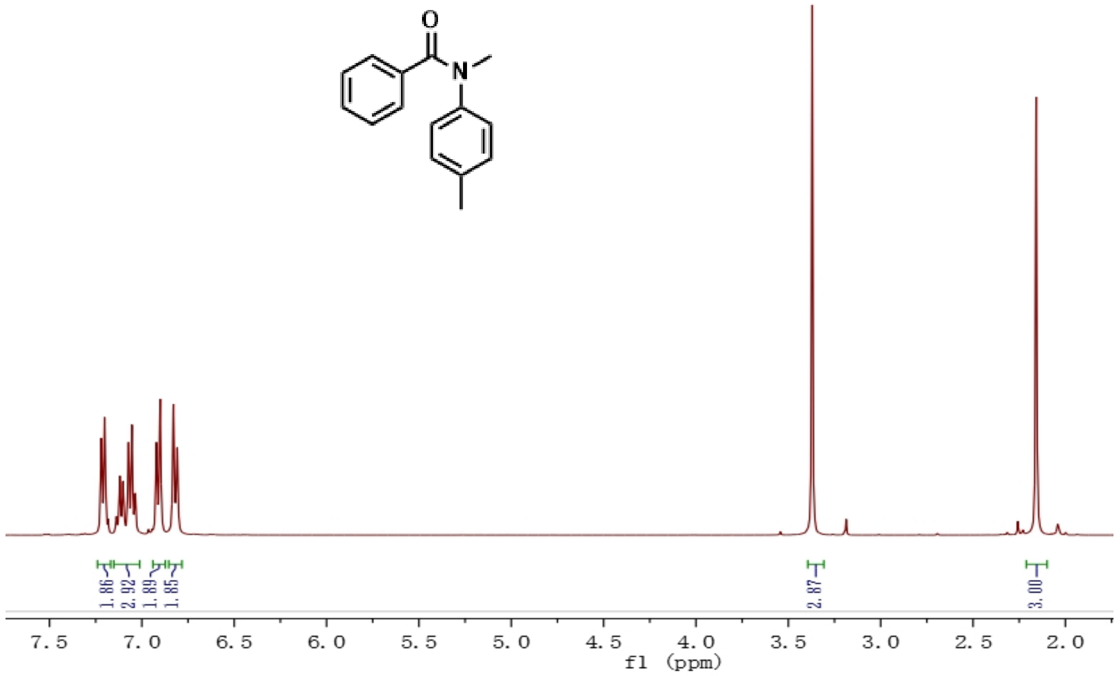

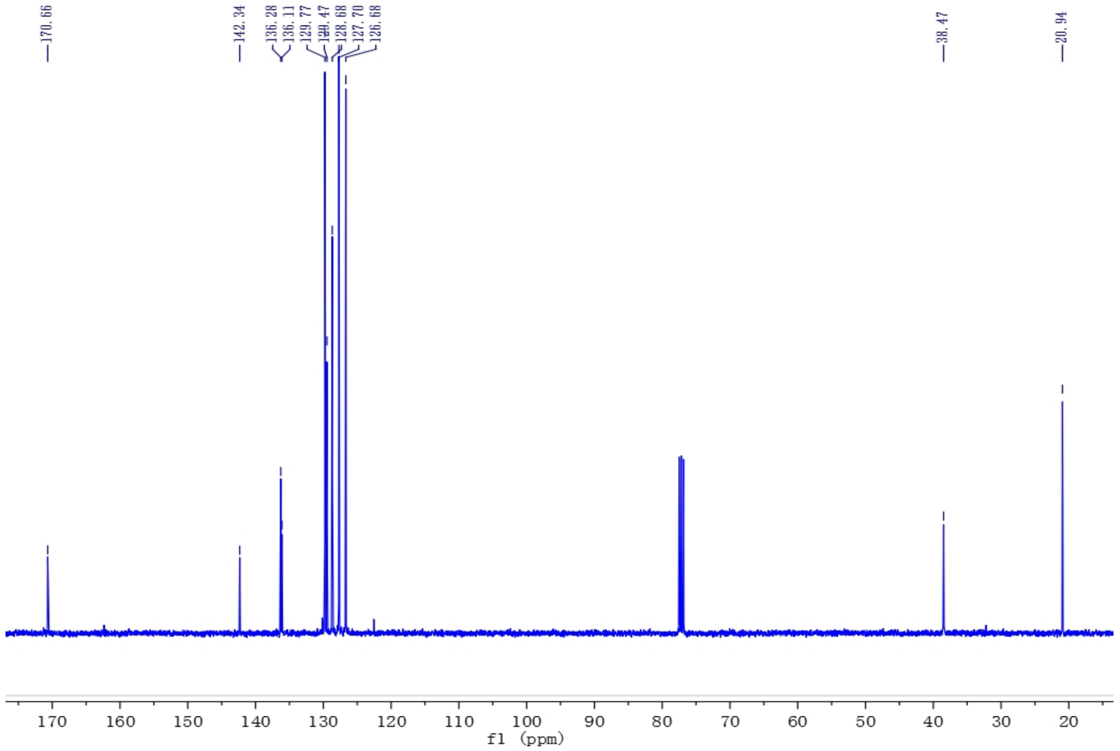

N-(4-chlorophenyl)-N-methylbenzamide

^{1}H NMR (400 MHz, CDCl3) δ 7.28 (dd, J = 7.7, 6.2 Hz, 3H), 7.19 (dd, J = 8.0, 6.1 Hz, 4H), 6.97 (d, J = 8.7 Hz, 2H), 3.46 (s, 3H). **^{13}C NMR (101 MHz, CDCl3)** δ 170.59, 143.48, 135.58, 132.05, 129.86, 129.32, 128.65, 128.08, 127.94, 38.38. **HRMS (ESI-TOF)**: m/z calculated for $C_{14}H_{12}ClNO$ $[M+H]^{+}$: 246.0680. Found: 246.0680.

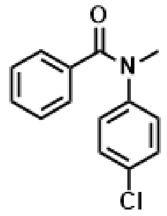

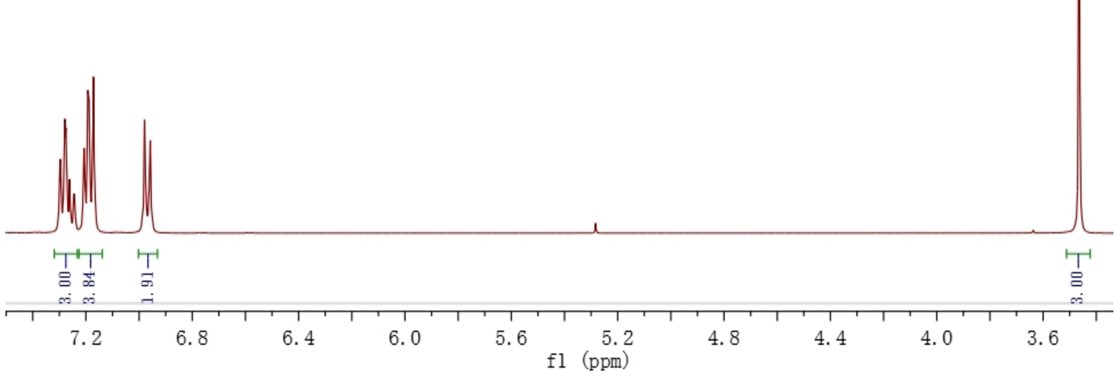

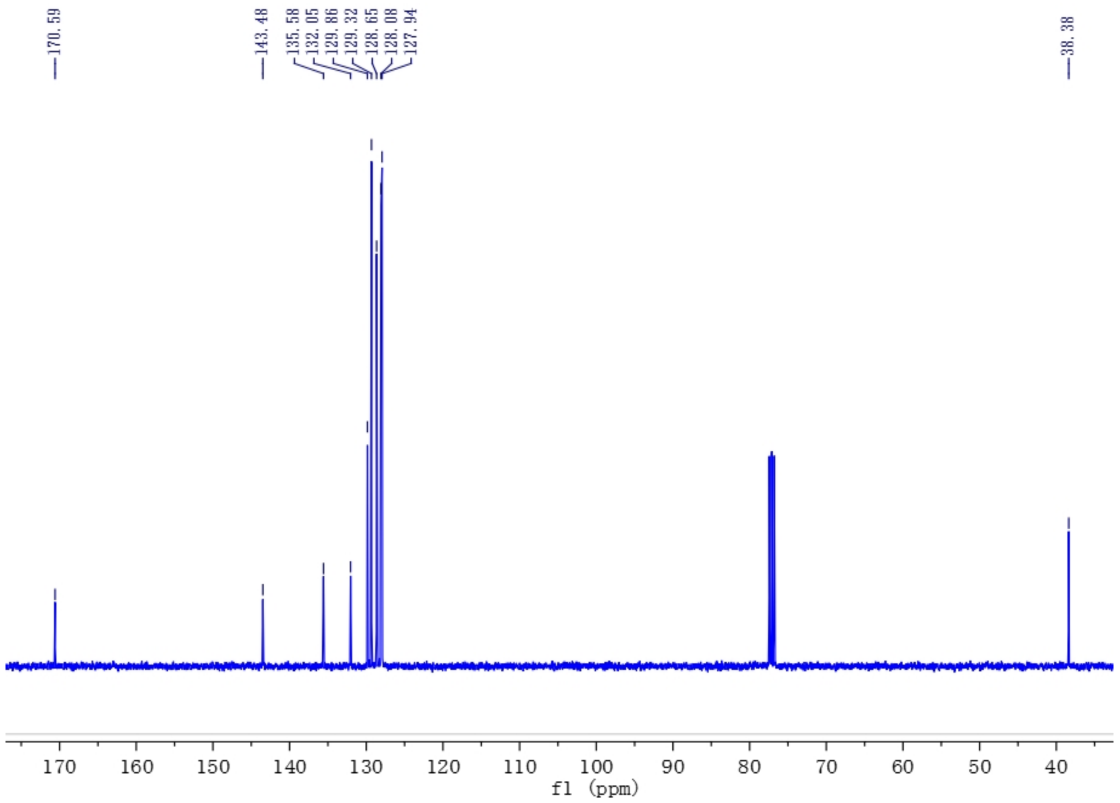

N-(4-bromophenyl)-N-methylbenzamide

^{1}H NMR (400 MHz, CDCl3) δ 7.34 (d, J = 8.7 Hz, 2H), 7.31 – 7.24 (m, 3H), 7.20 (d, J = 7.5 Hz, 2H), 6.91 (d, J = 8.7 Hz, 2H), 3.47 (s, 3H). **^{13}C NMR (101 MHz, CDCl3)** δ 170.56, 144.01, 135.53, 132.31, 129.90, 128.67, 128.40, 127.96, 119.94, 38.35. **HRMS (ESI-TOF)**: m/z calculated for $C_{14}H_{12}BrNO$ $[M+H]^+$: 290.0175. Found: 290.0175.

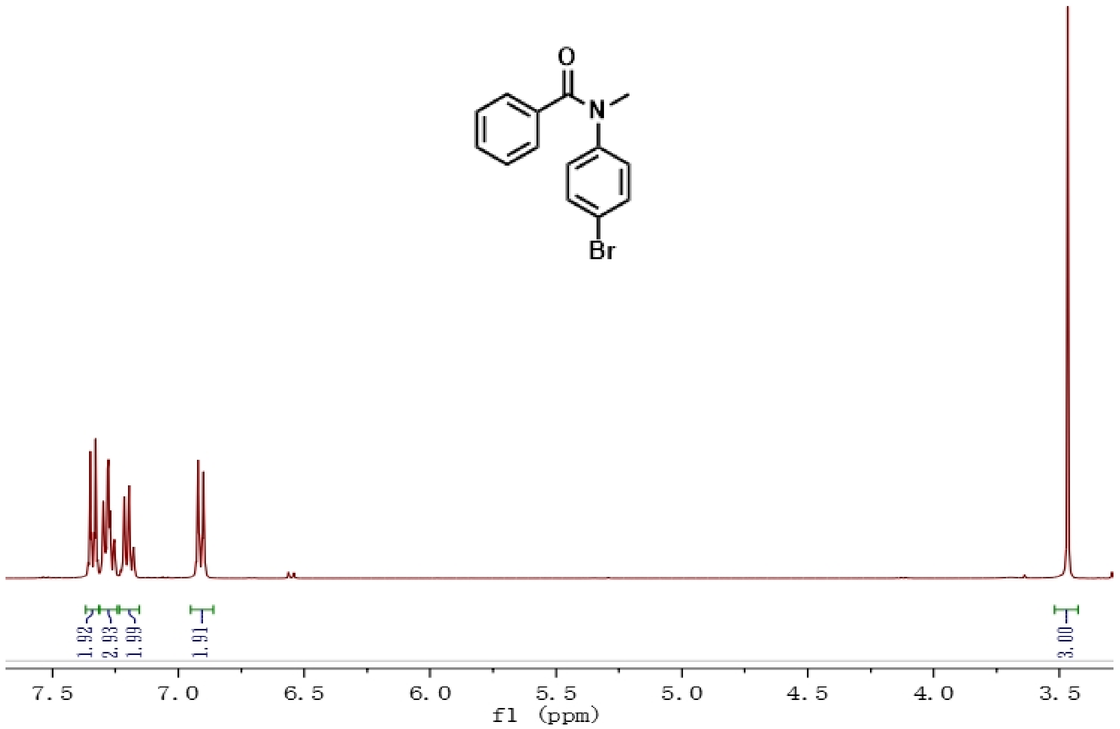

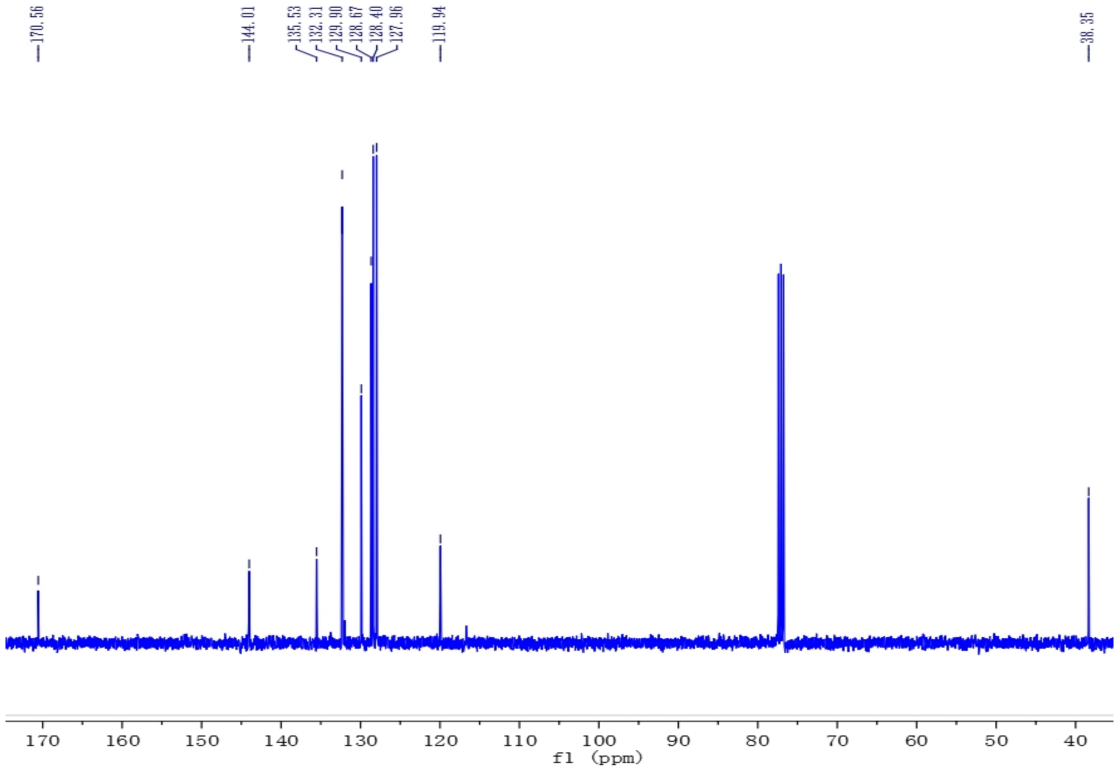

N-(4-methoxyphenyl)-N-methylbenzamide

^{1}H NMR (400 MHz, CDCl3) δ 7.32 – 7.25 (m, 2H), 7.16 (d, J = 7.5 Hz, 3H), 6.95 (d, J = 8.6 Hz, 2H), 6.73 (d, J = 8.8 Hz, 2H), 3.72 (s, 3H), 3.45 (s, 3H). **^{13}C NMR (101 MHz, CDCl3)** δ 170.73, 157.89, 137.80, 136.11, 129.40, 128.62, 128.05, 127.72, 114.33, 55.36, 38.60. **HRMS (ESI-TOF)**: m/z calculated for $C_{15}H_{15}NO_2$ $[M+H]^+$: 242.1176. Found: 242.1176.

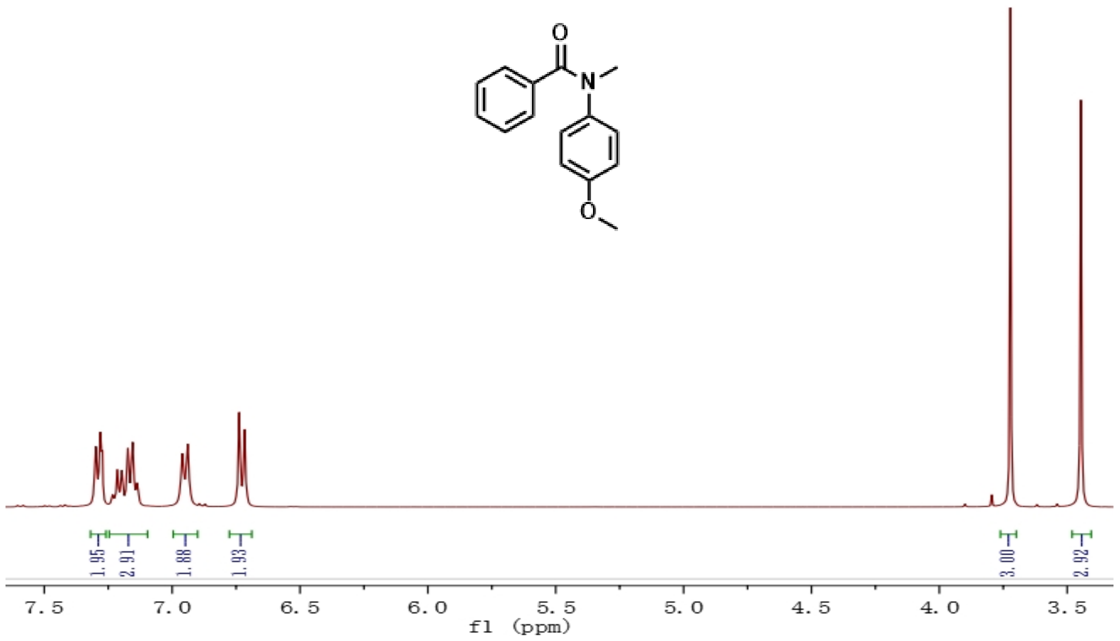

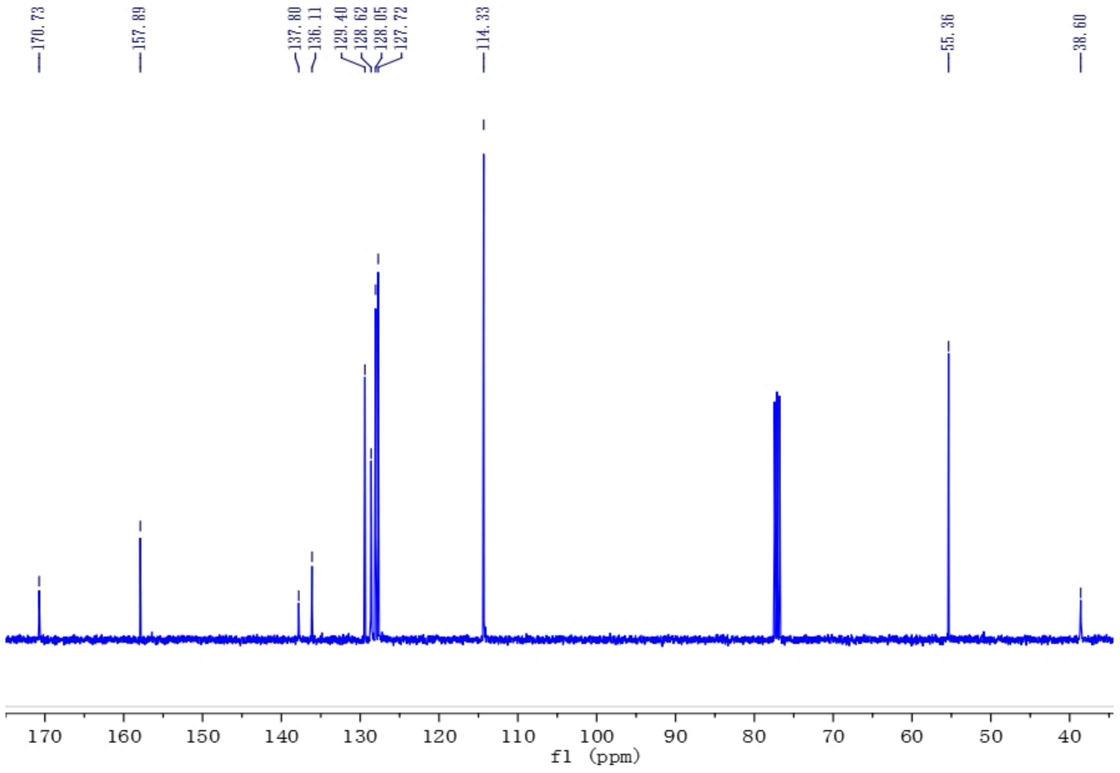

10 BET areas calculation using BETSI[2]

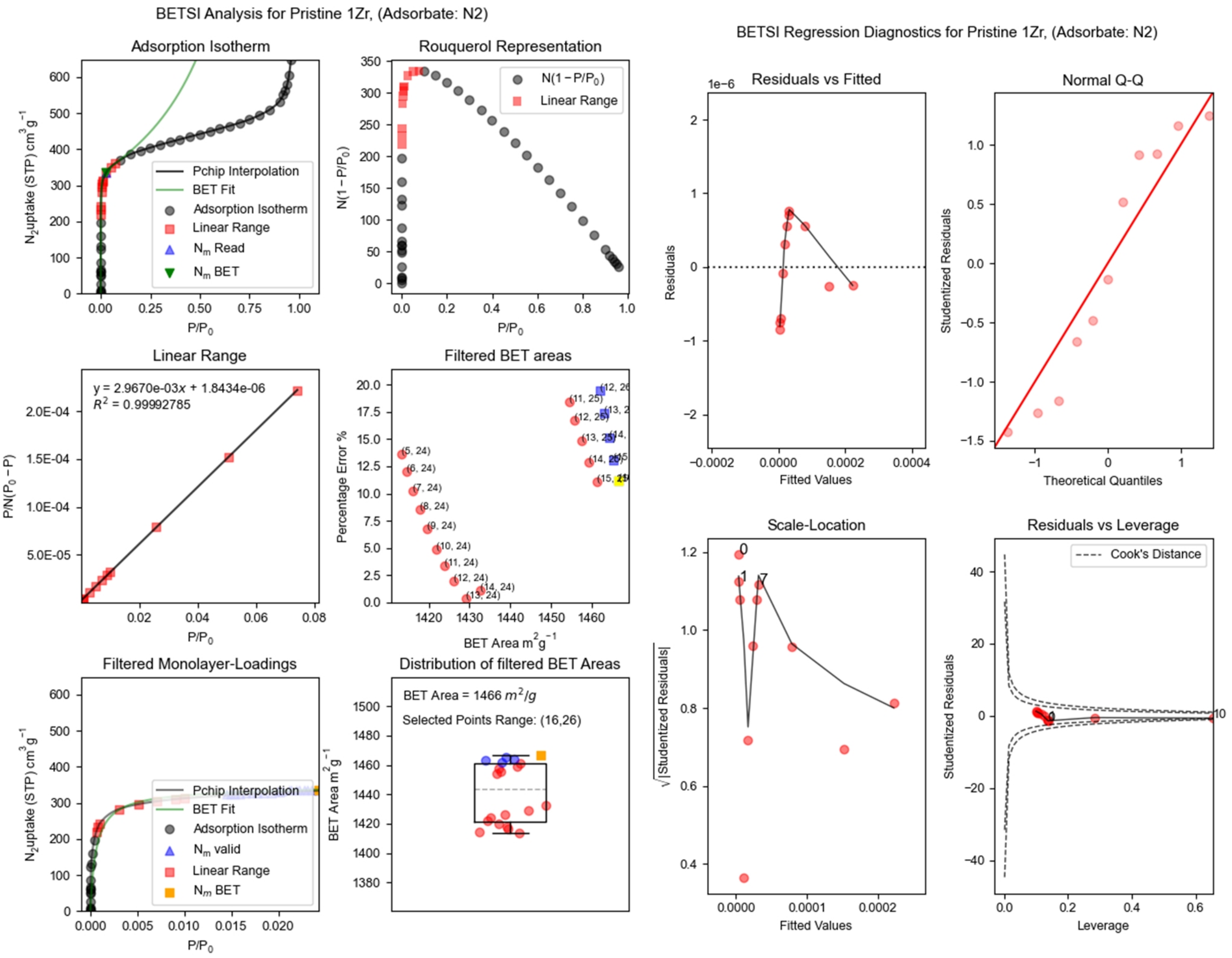

Figure S58 BETSI analysis and the corresponding regression diagnostics for **1^{Zr}**

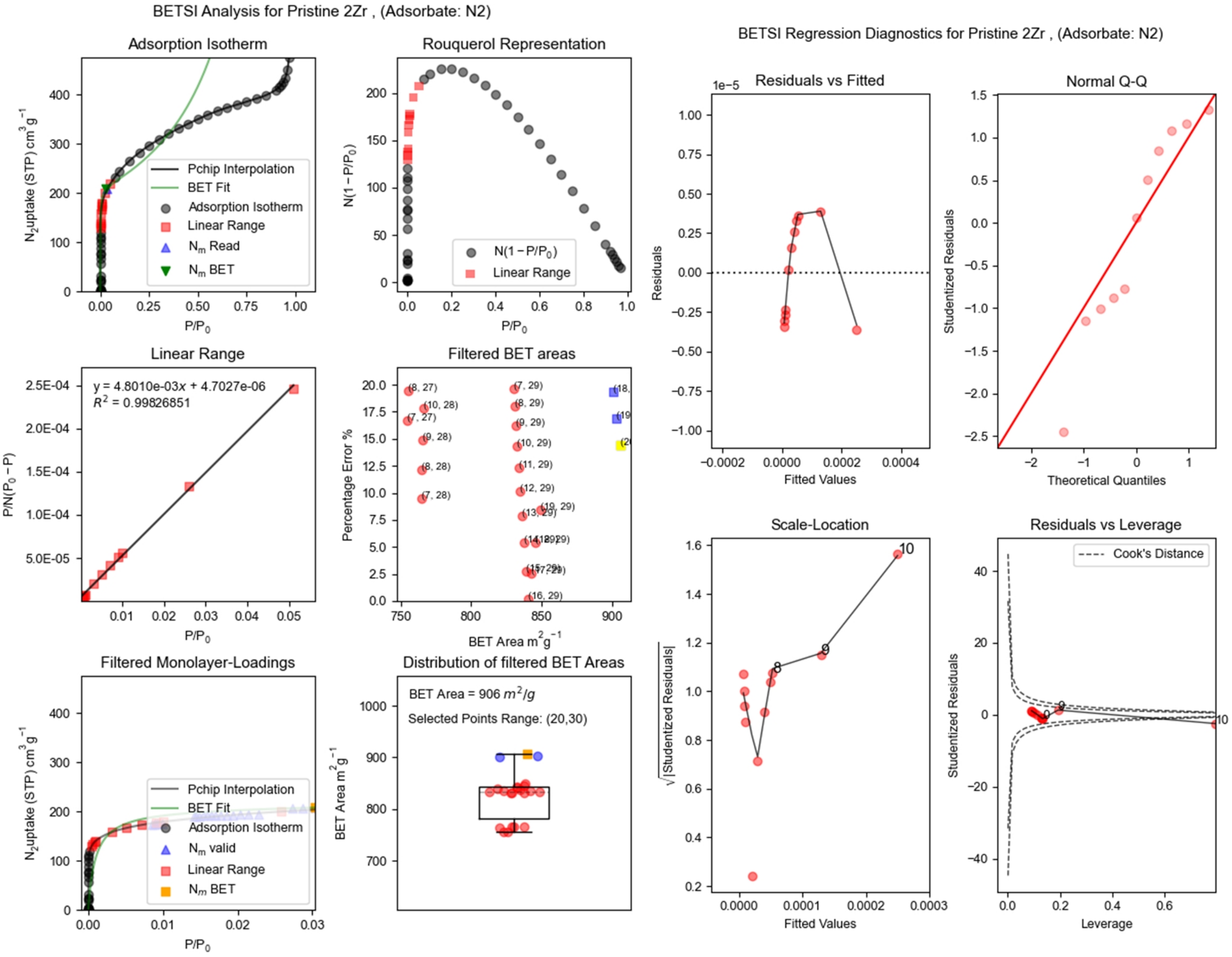

Figure S59. BETSI analysis and the corresponding regression diagnostics for **2^{Zr}**

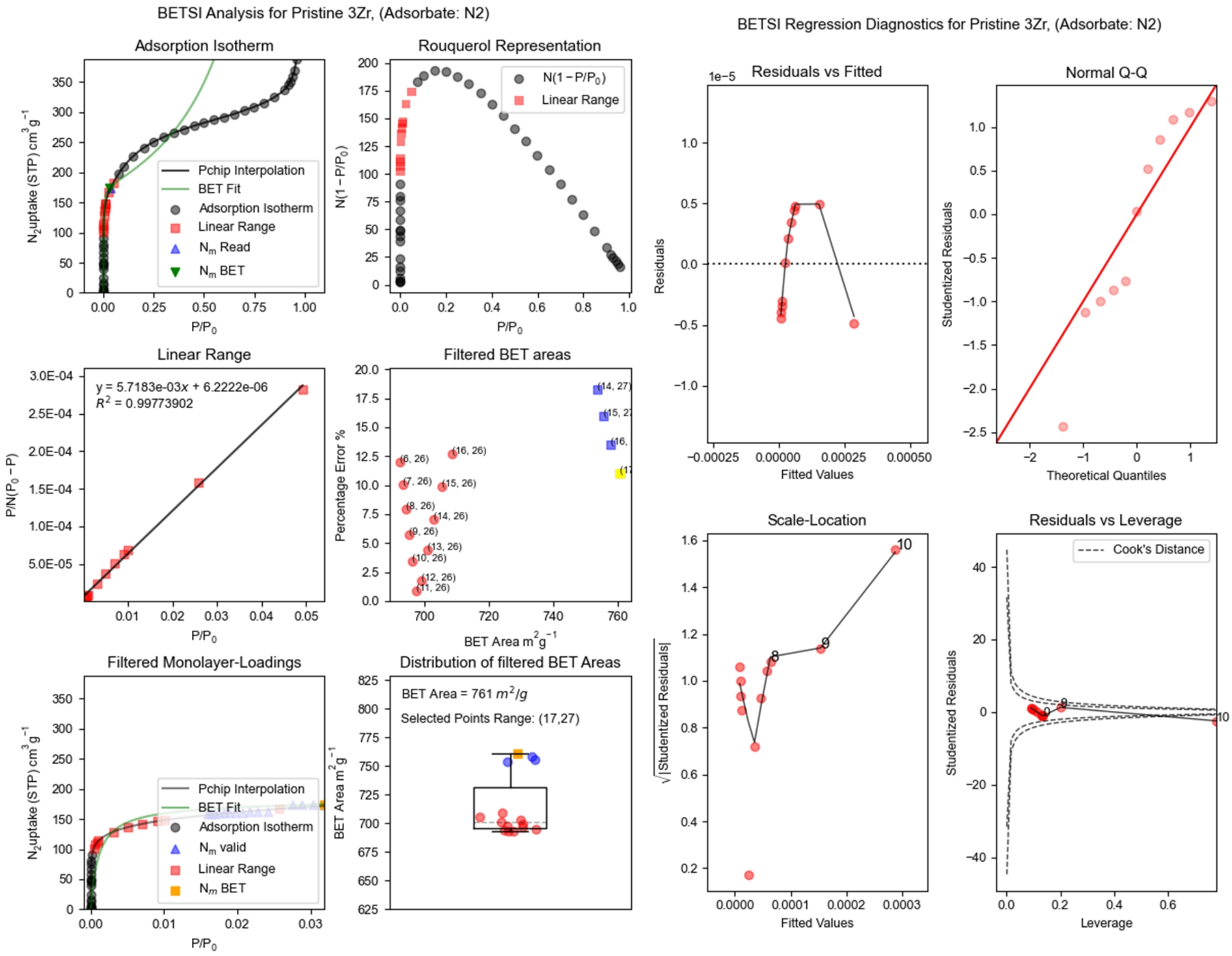

Figure S60. BETSI analysis and the corresponding regression diagnostics for **3**^{Zr}

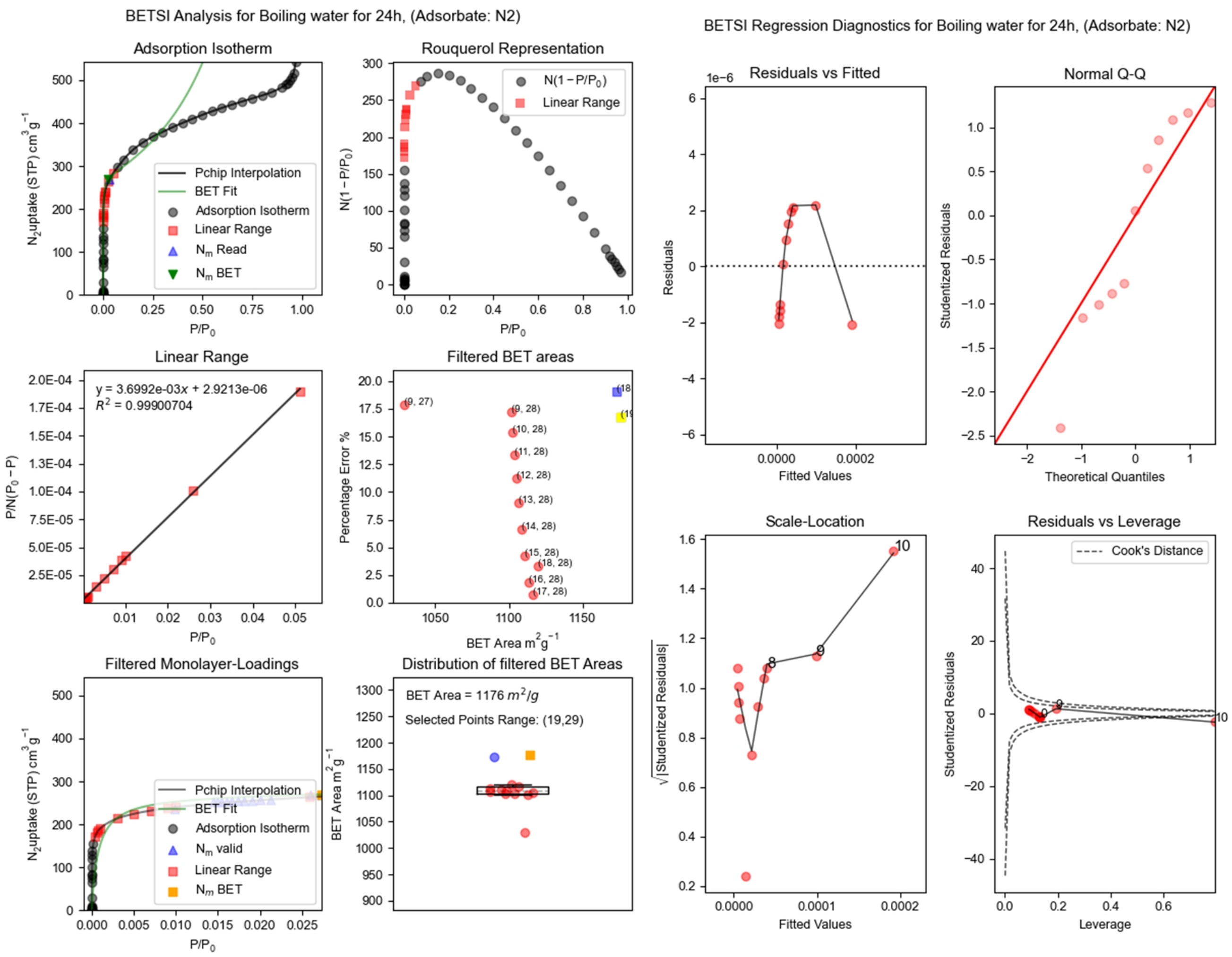

Figure S61. BETSI analysis and the corresponding regression diagnostics for **1^{Zr}** after being treated with boiling water for 24 h

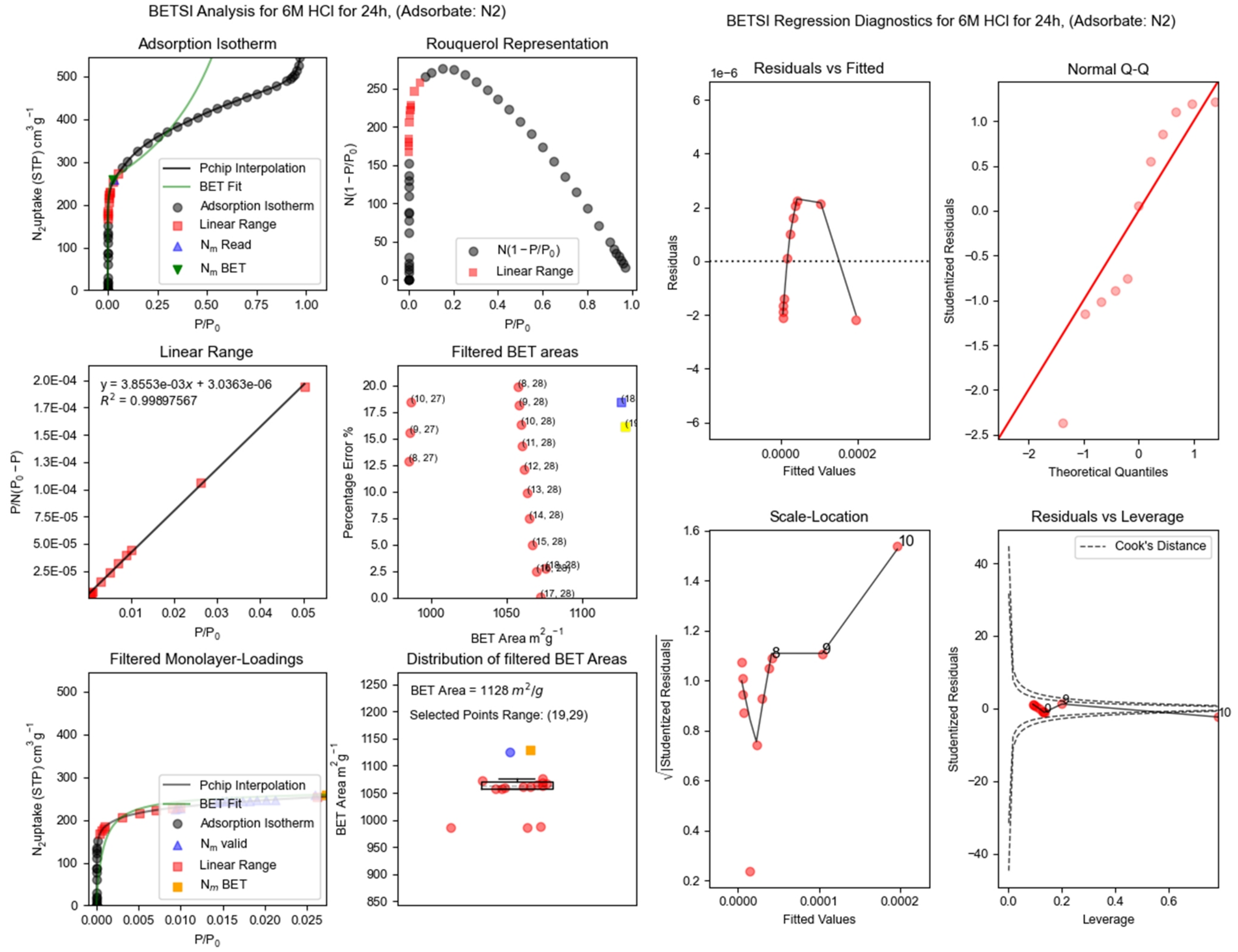

Figure S62. BETSI analysis and the corresponding regression diagnostics for $\mathbf{1^{Zr}}$ after being treated with 6M HCl for 24 h

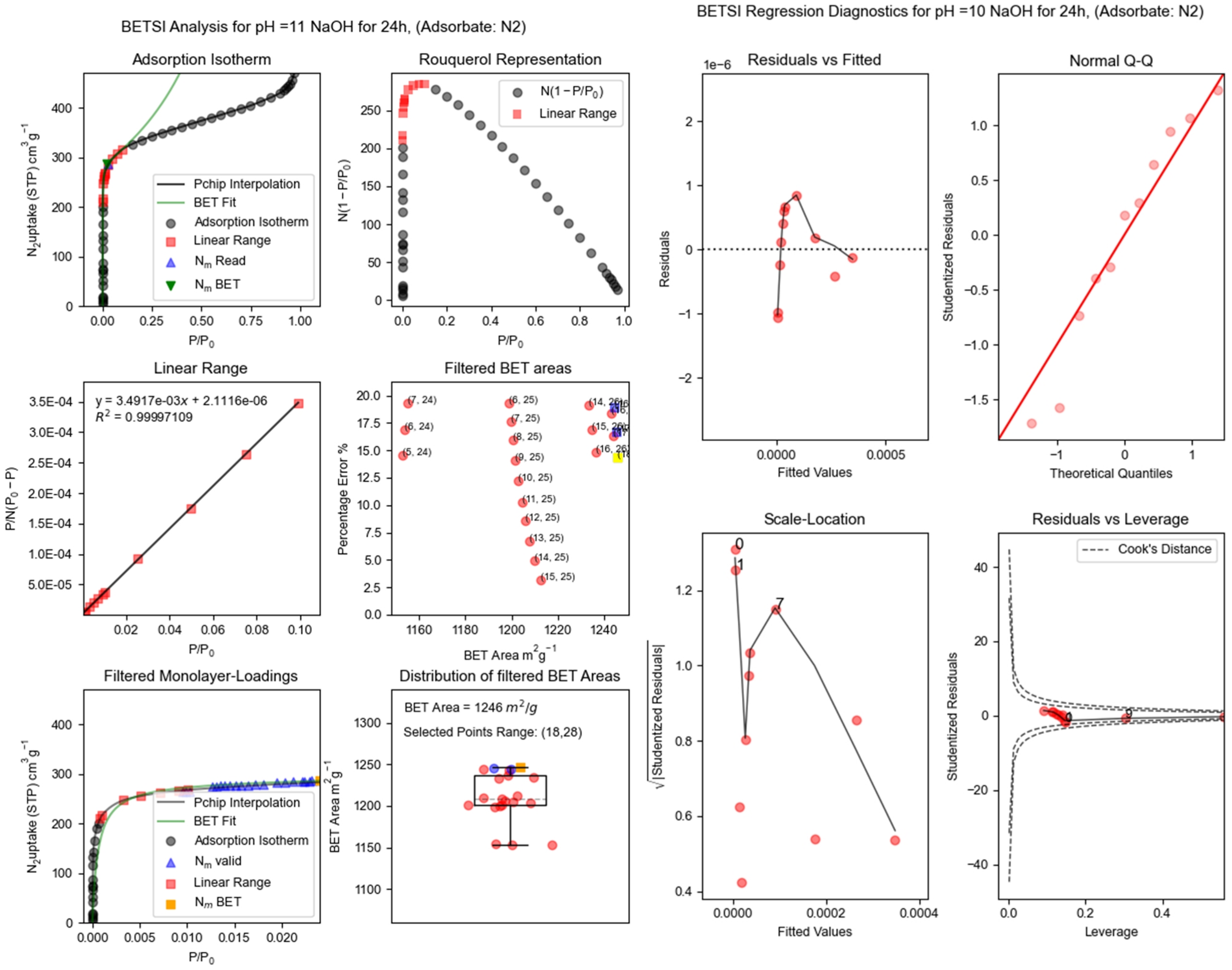

Figure S63. BETSI analysis and the corresponding regression diagnostics for $\mathbf{1^{Zr}}$ after being treated with 11M NaOH for 24 h

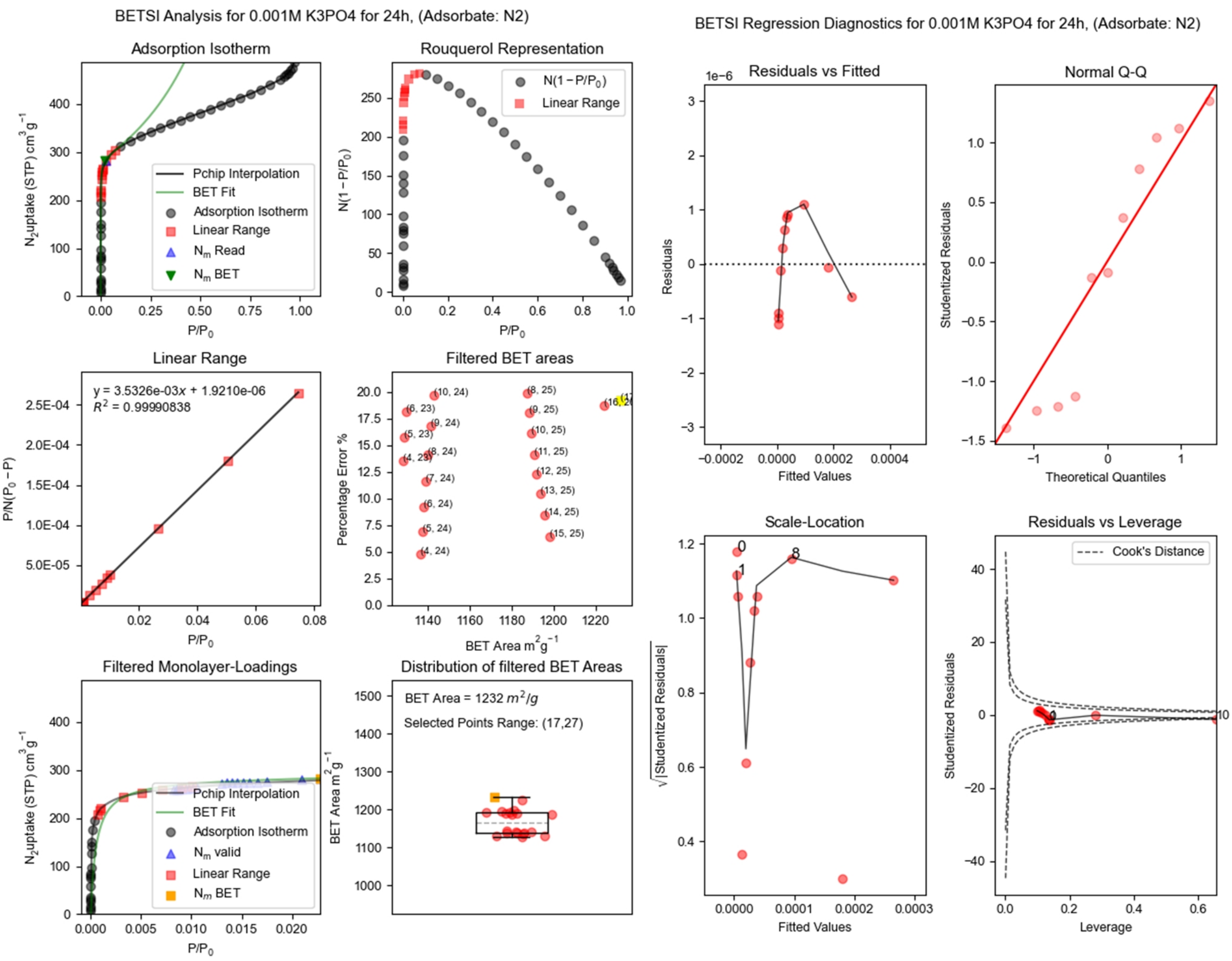

Figure S64. BETSI analysis and the corresponding regression diagnostics for **1^Zr^** after being treated with 0.001M K_3PO_4 for 24 h

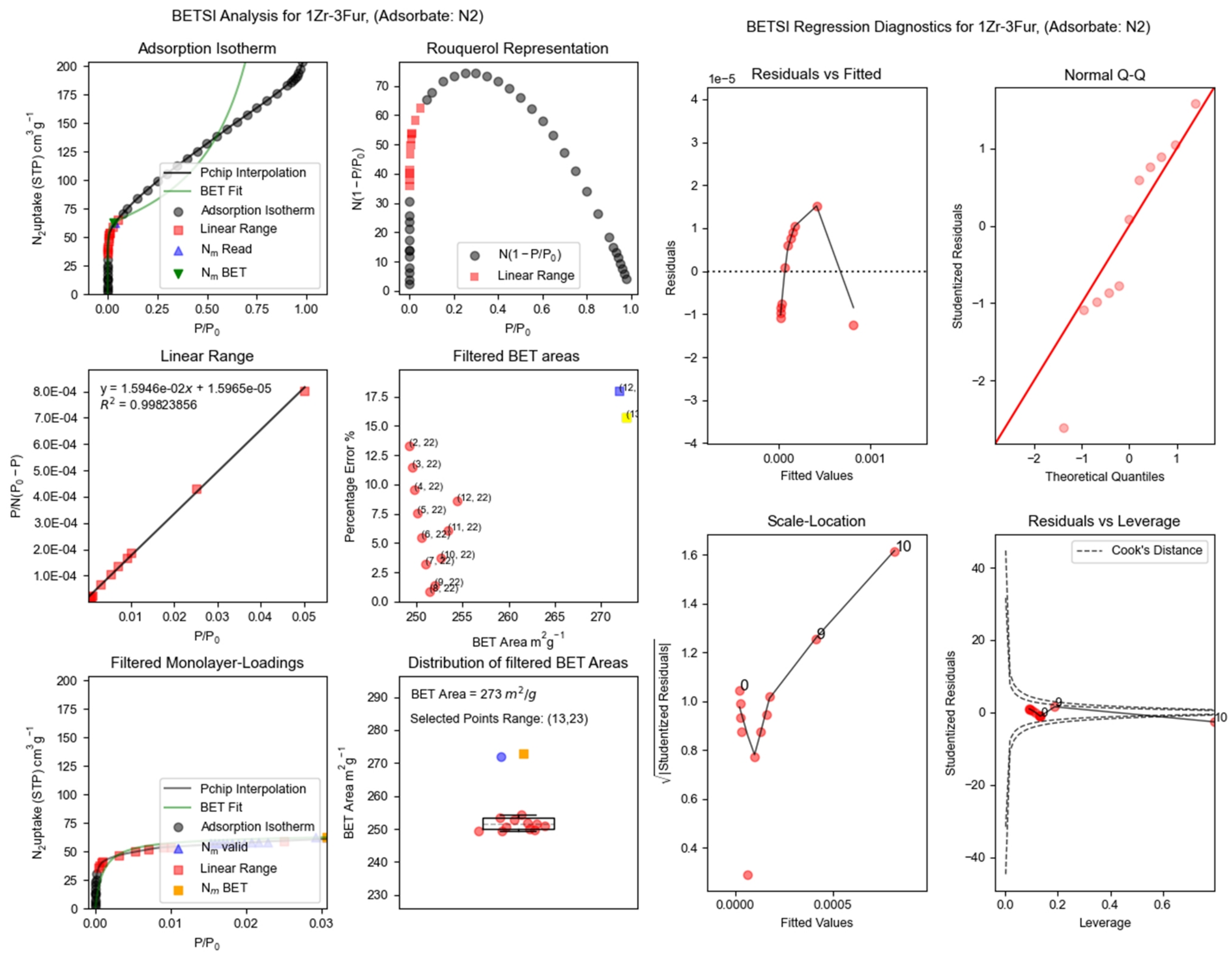

Figure S65. BETSI analysis and the corresponding regression diagnostics for $\mathbf{1}^{\mathbf{Zr\text{-}3Fur}}$

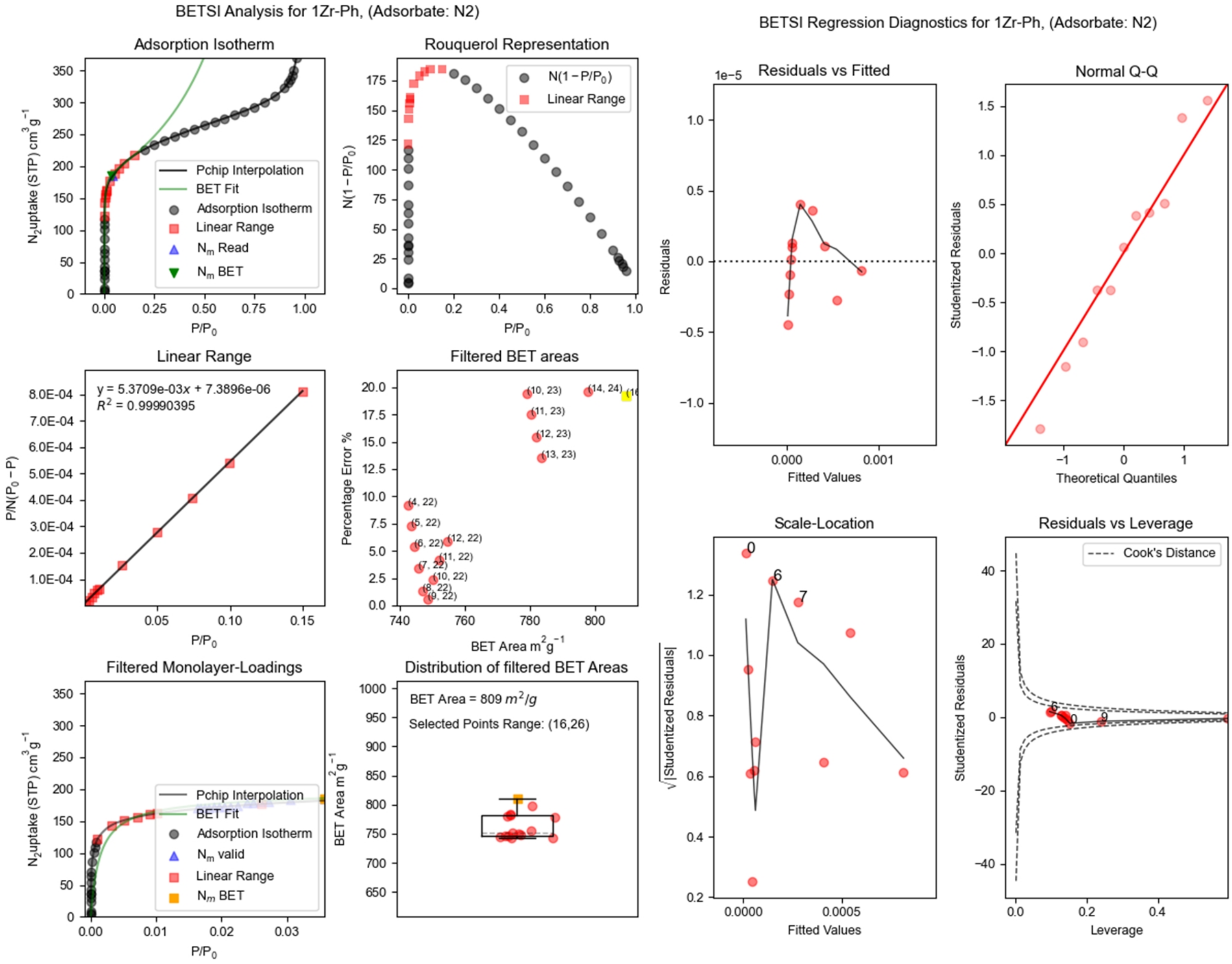

Figure S66. BETSI analysis and the corresponding regression diagnostics for **1**$^{\mathbf{Zr\text{-}Ph}}$

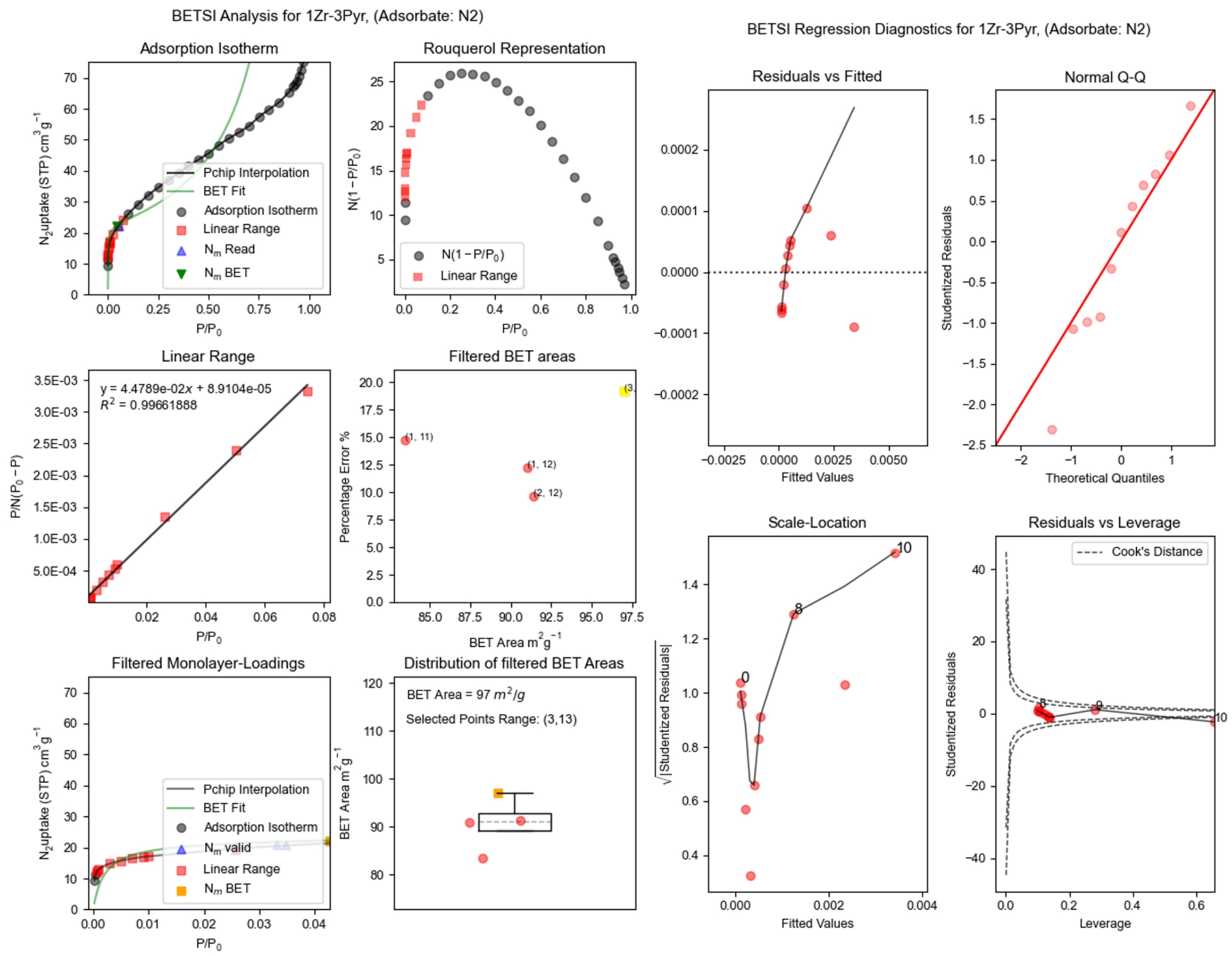

Figure S67. BETSI analysis and the corresponding regression diagnostics for $\mathbf{1}^{\mathbf{Zr\text{-}3Pyr}}$

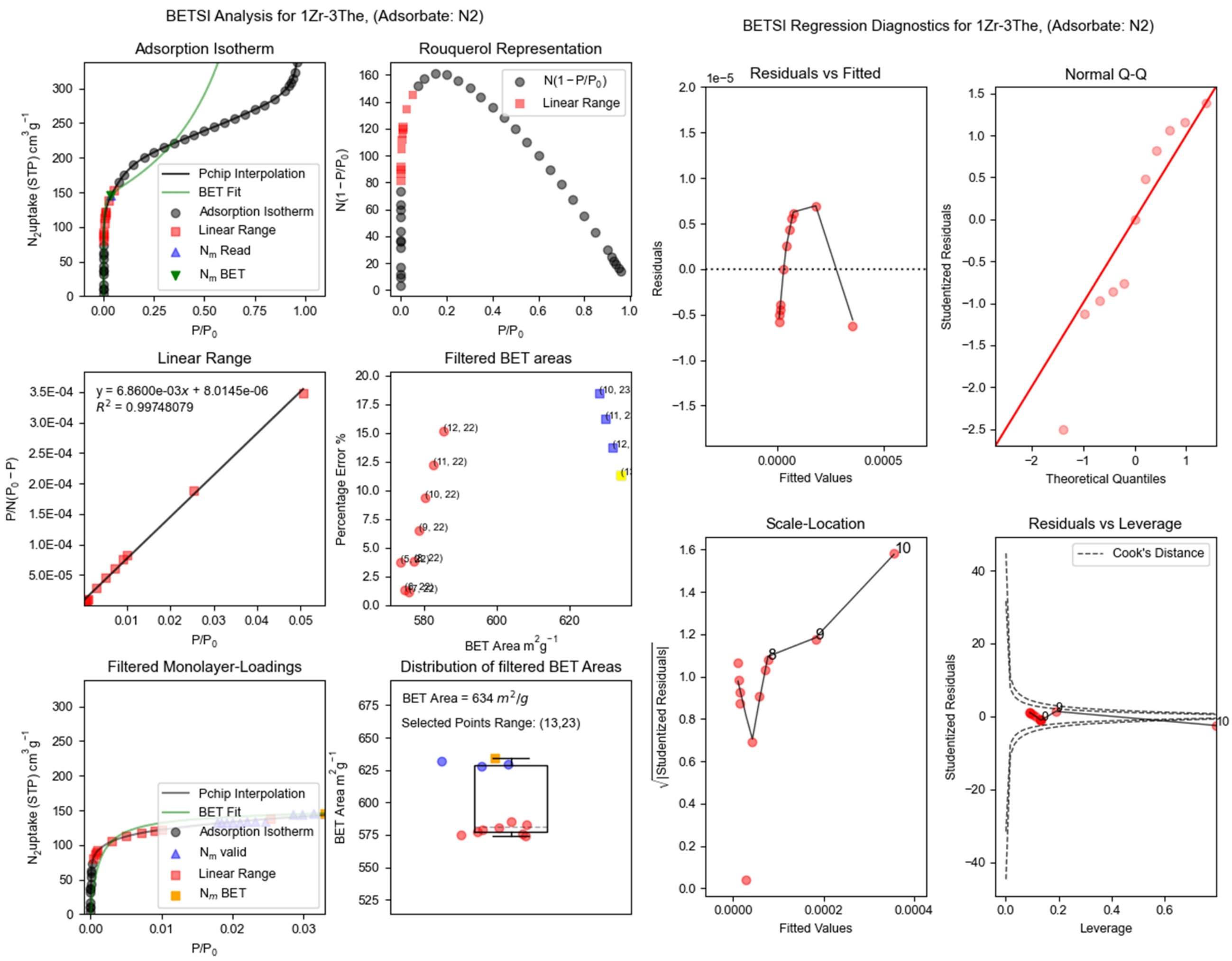

Figure S68. BETSI analysis and the corresponding regression diagnostics for **1**$^{\mathbf{Zr\text{-}3The}}$

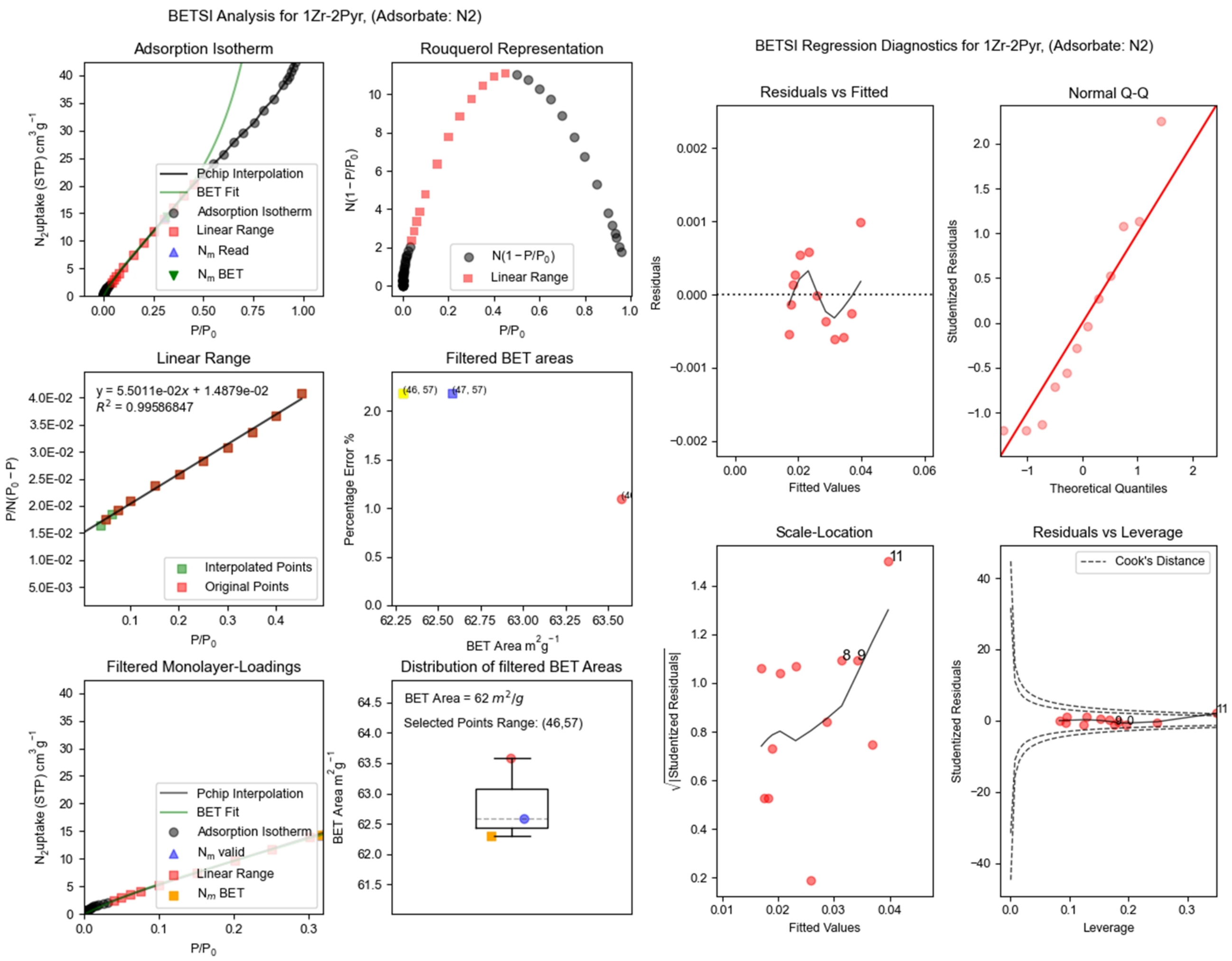

Figure S69. BETSI analysis and the corresponding regression diagnostics for $\mathbf{1}^{\mathbf{Zr\text{-}2Pyr}}$

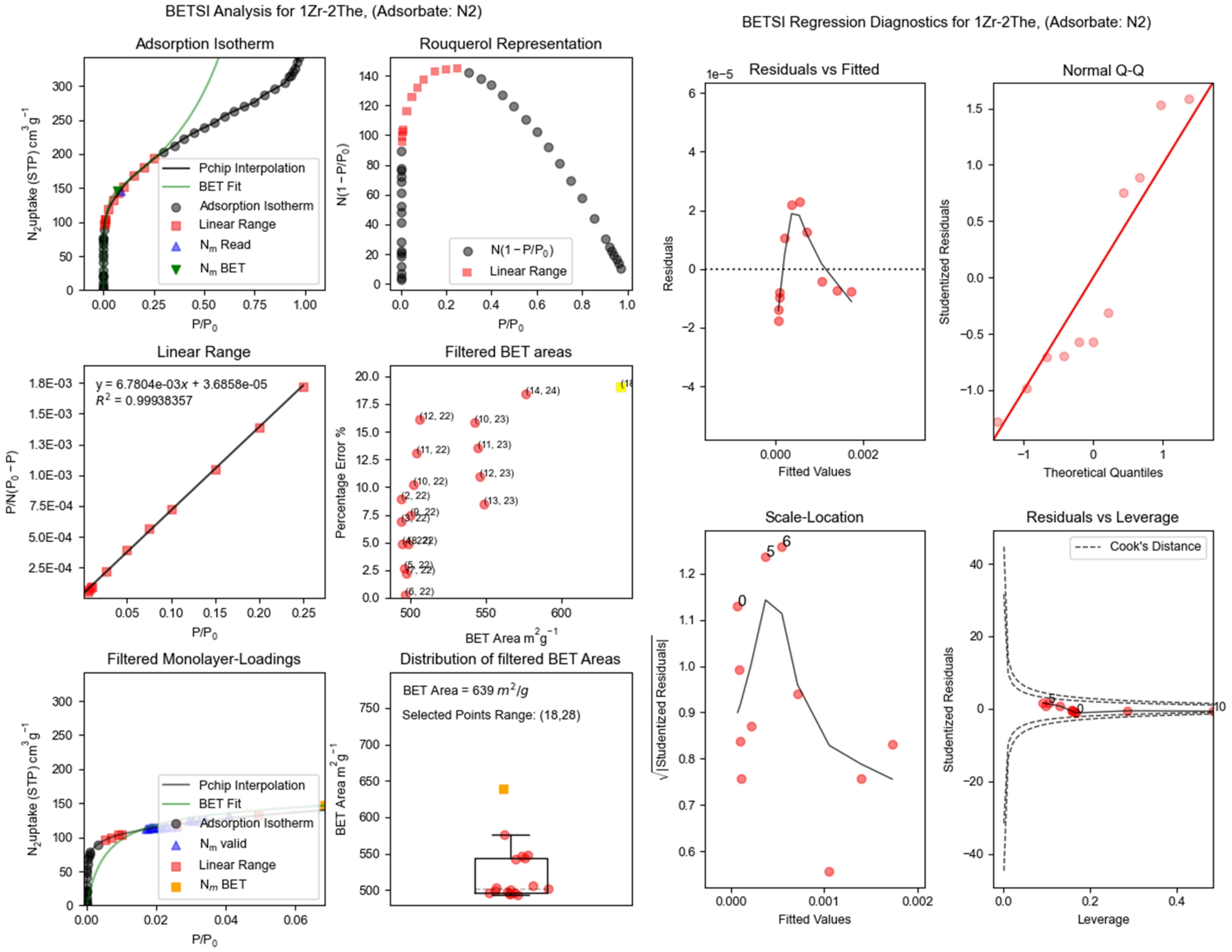

Figure S70. BETSI analysis and the corresponding regression diagnostics for **1**$^{\mathbf{Zr\text{-}2The}}$

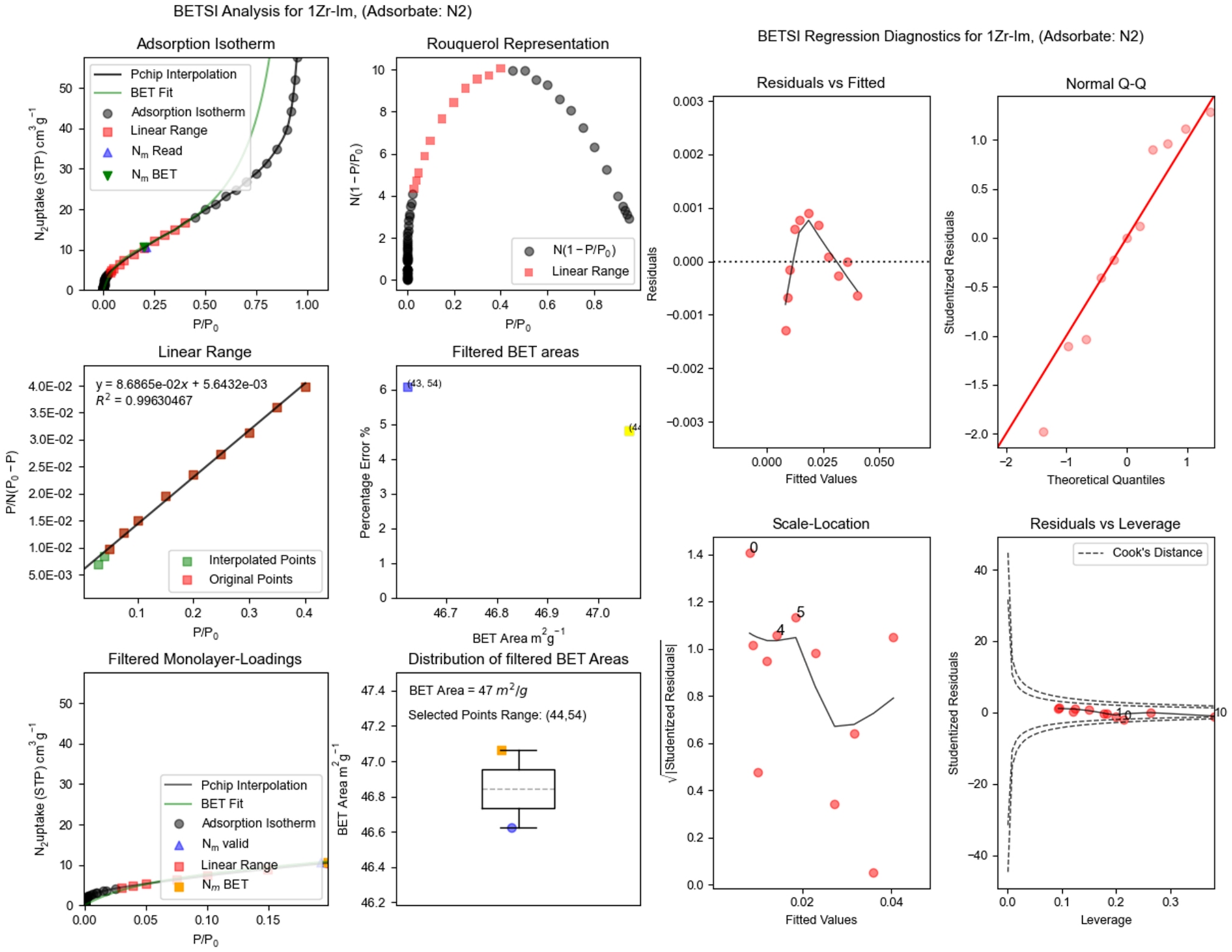

Figure S71. BETSI analysis and the corresponding regression diagnostics for **1**$^{\mathbf{Zr\text{-}3The}}$

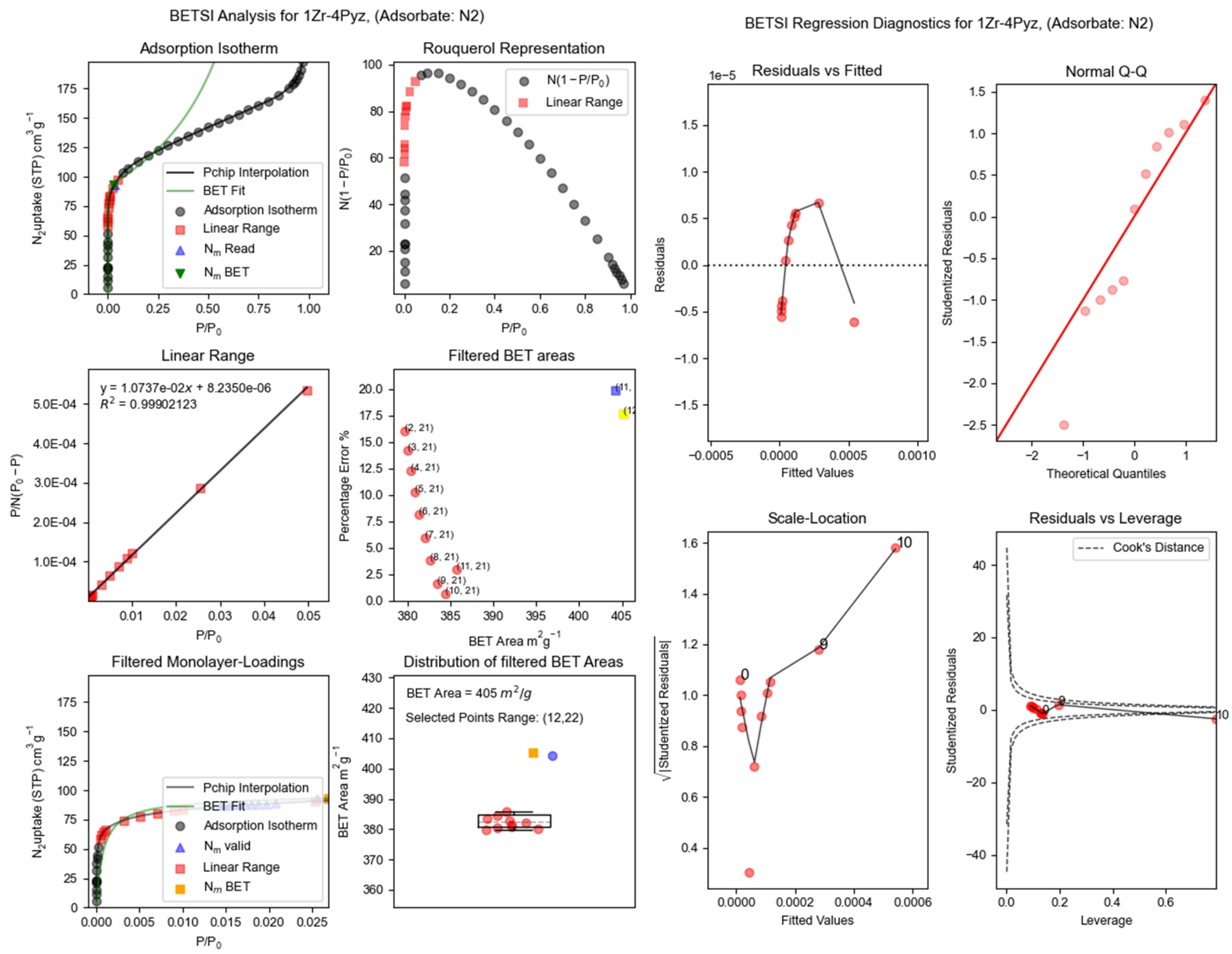

Figure S72. BETSI analysis and the corresponding regression diagnostics for **1**$^{\mathbf{Zr\text{-}4Pyz}}$

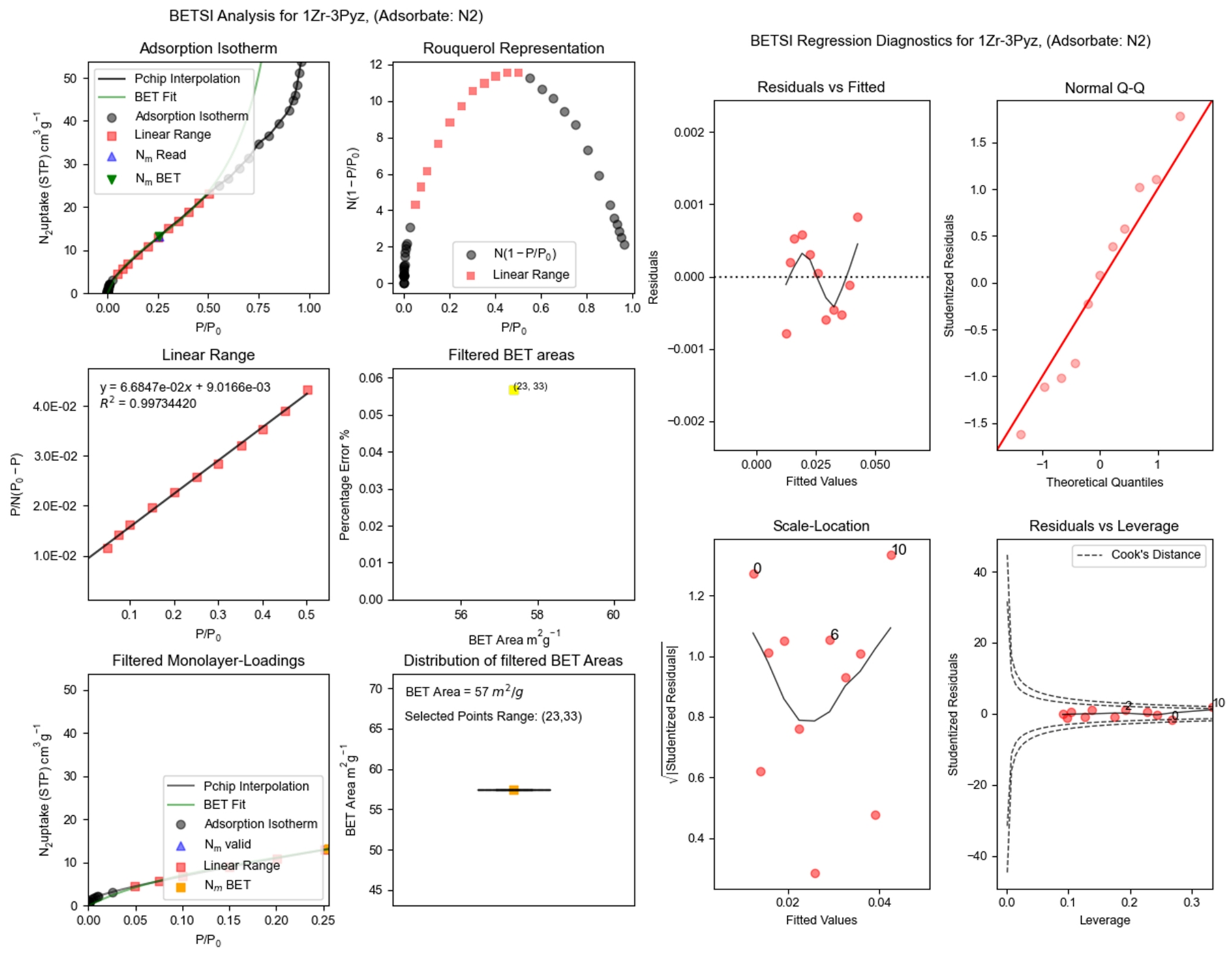

Figure S73. BETSI analysis and the corresponding regression diagnostics for **1**$^{\mathbf{Zr\text{-}3Pyz}}$

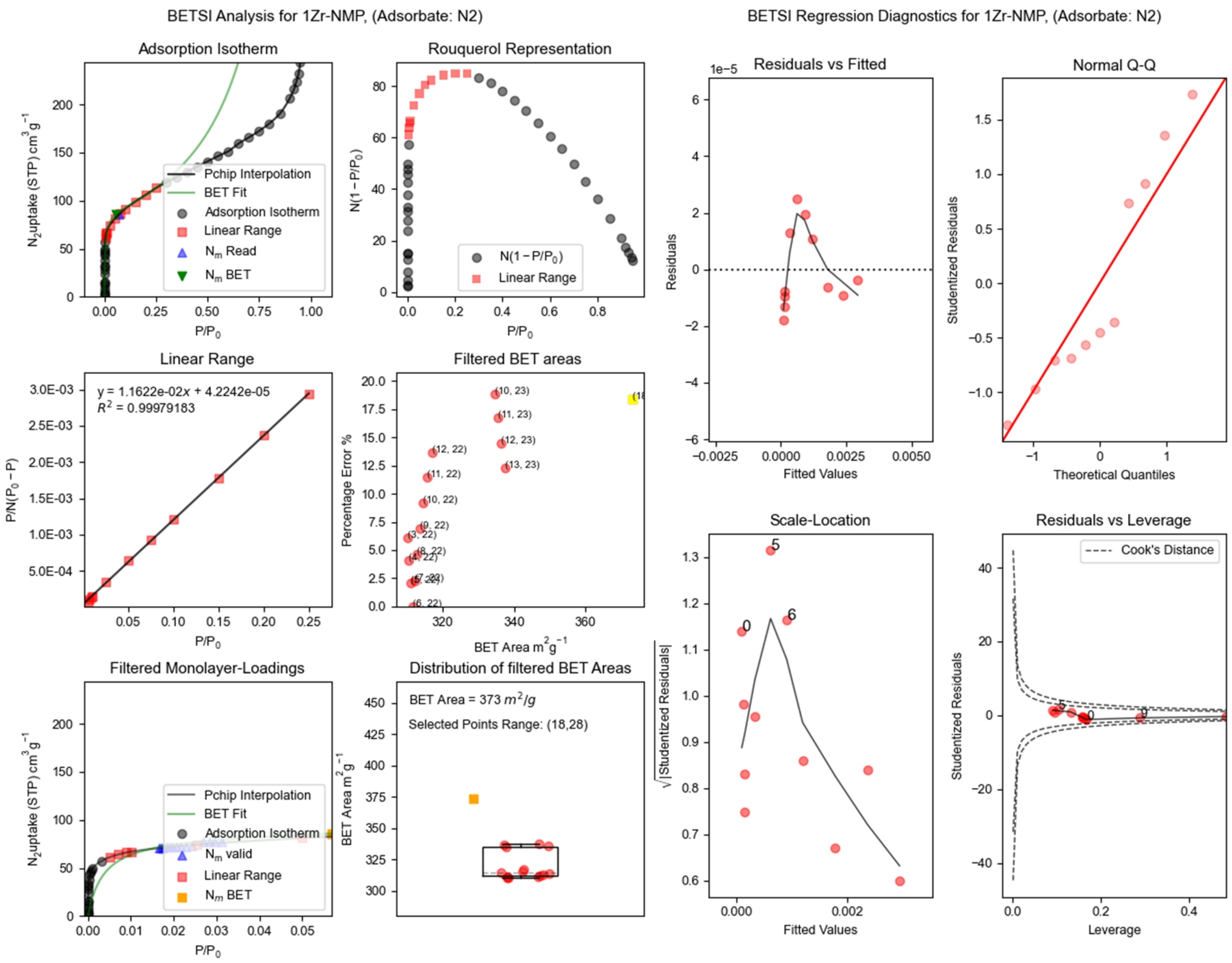

Figure S74. BETSI analysis and the corresponding regression diagnostics for **1**$^{\mathbf{Zr\text{-}NMP}}$

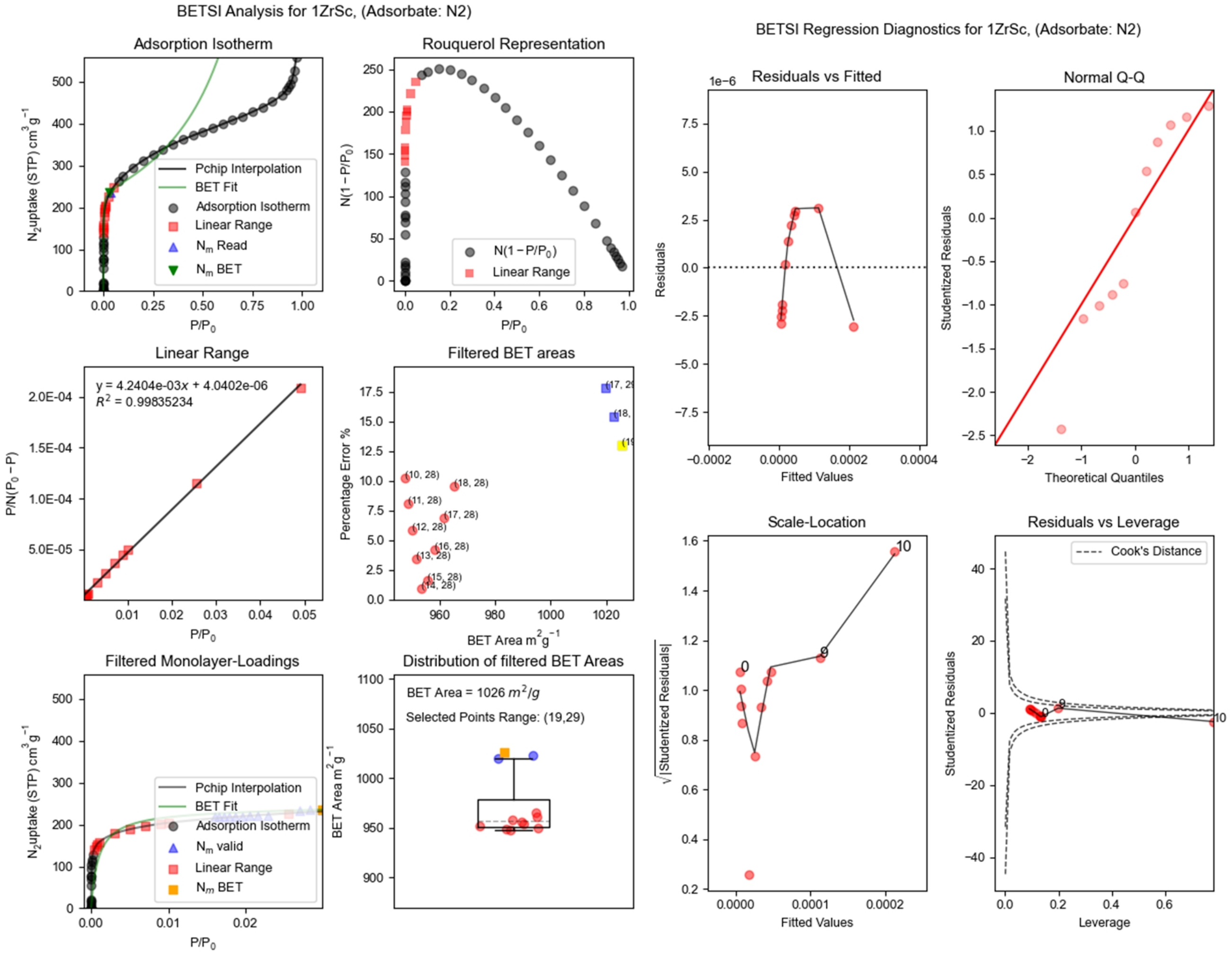

Figure S75. BETSI analysis and the corresponding regression diagnostics for **1**$^{\mathbf{ZrSc}}$

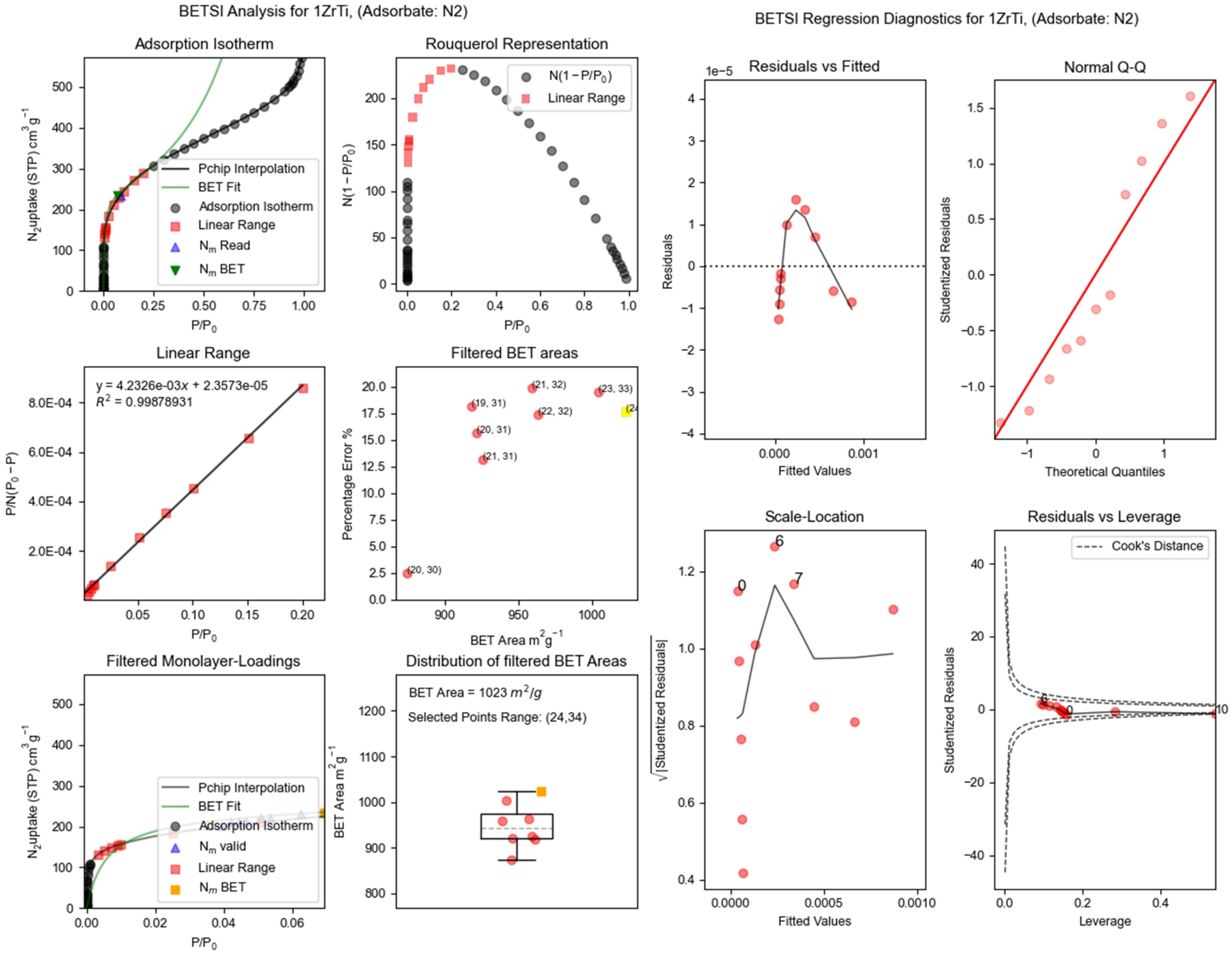

Figure S76. BETSI analysis and the corresponding regression diagnostics for **1**$^{\mathbf{ZrTi}}$

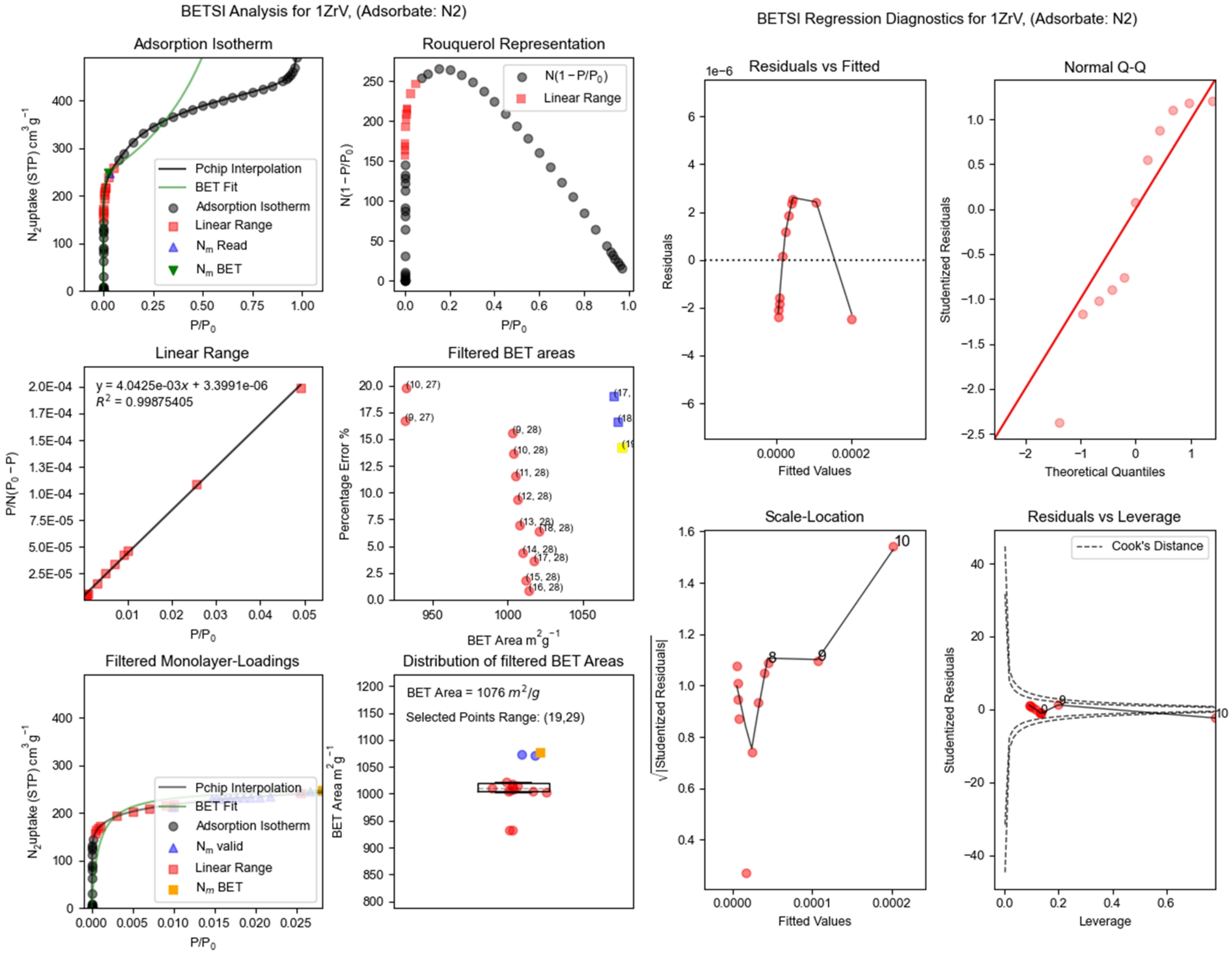

Figure S77. BETSI analysis and the corresponding regression diagnostics for **1**$^{\mathbf{ZrV}}$

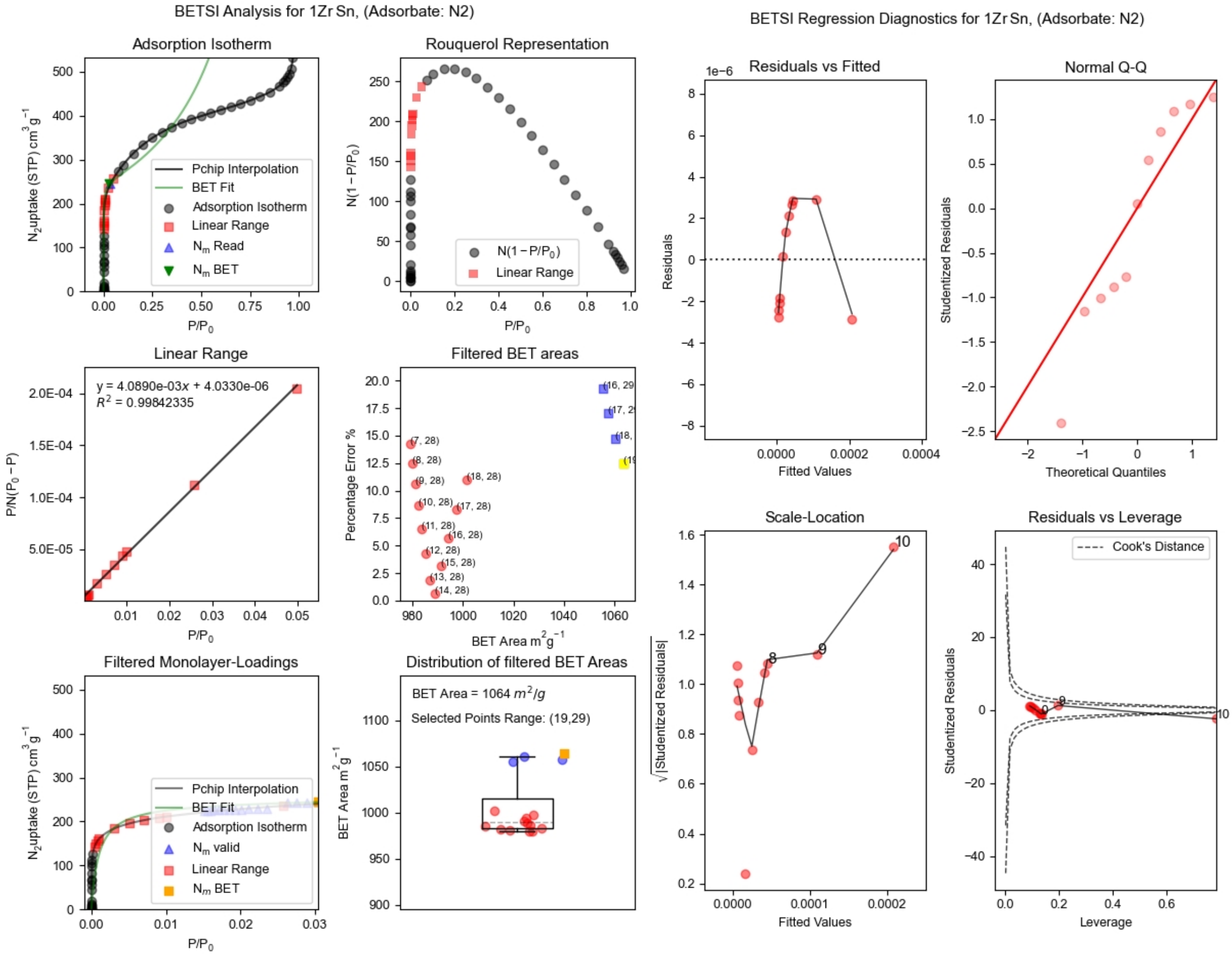

Figure S78. BETSI analysis and the corresponding regression diagnostics for $\mathbf{1}^{\mathbf{ZrSn}}$

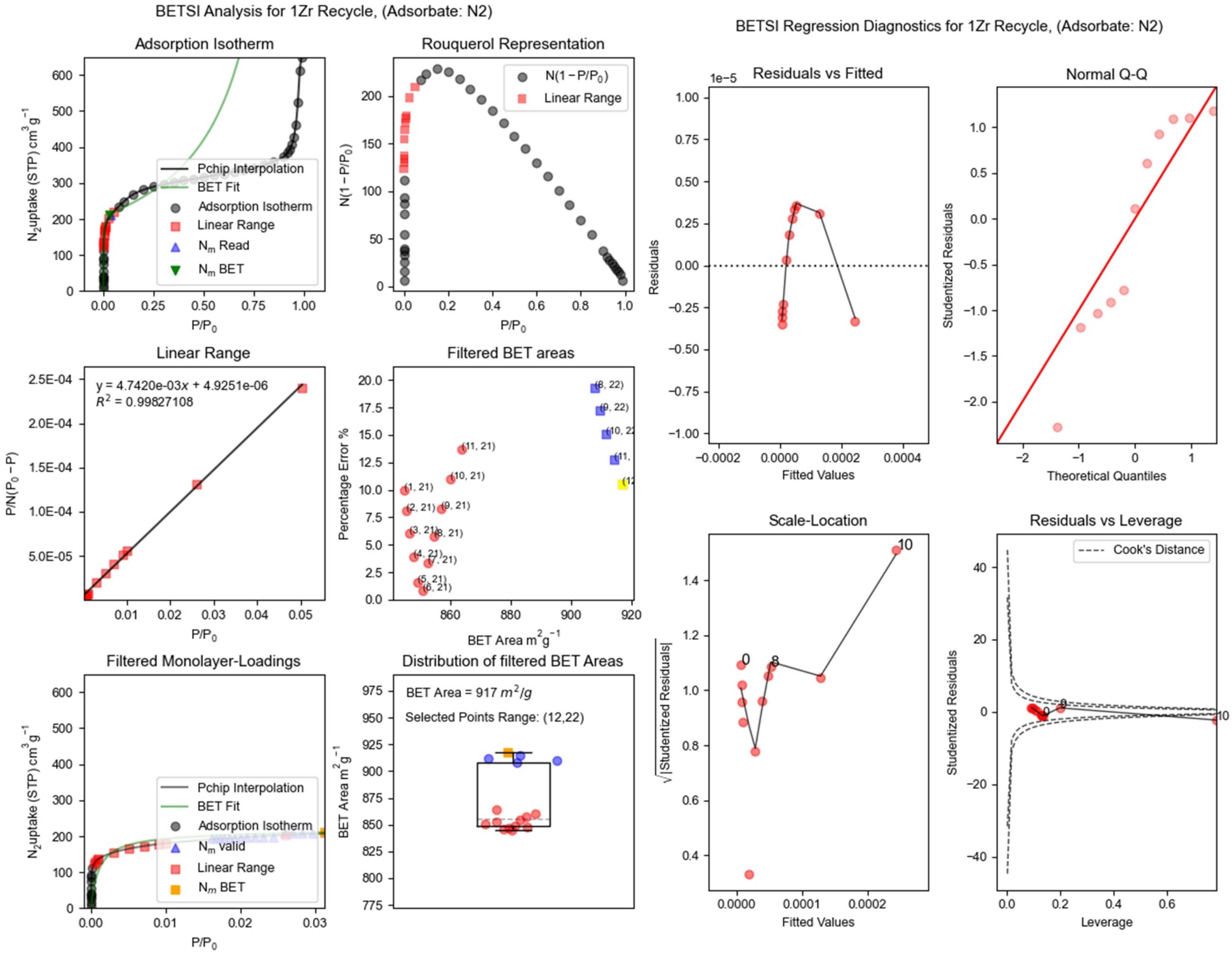

Figure S79. BETSI analysis and the corresponding regression diagnostics for the recycled $\mathbf{1}^{\mathbf{Zr}}$

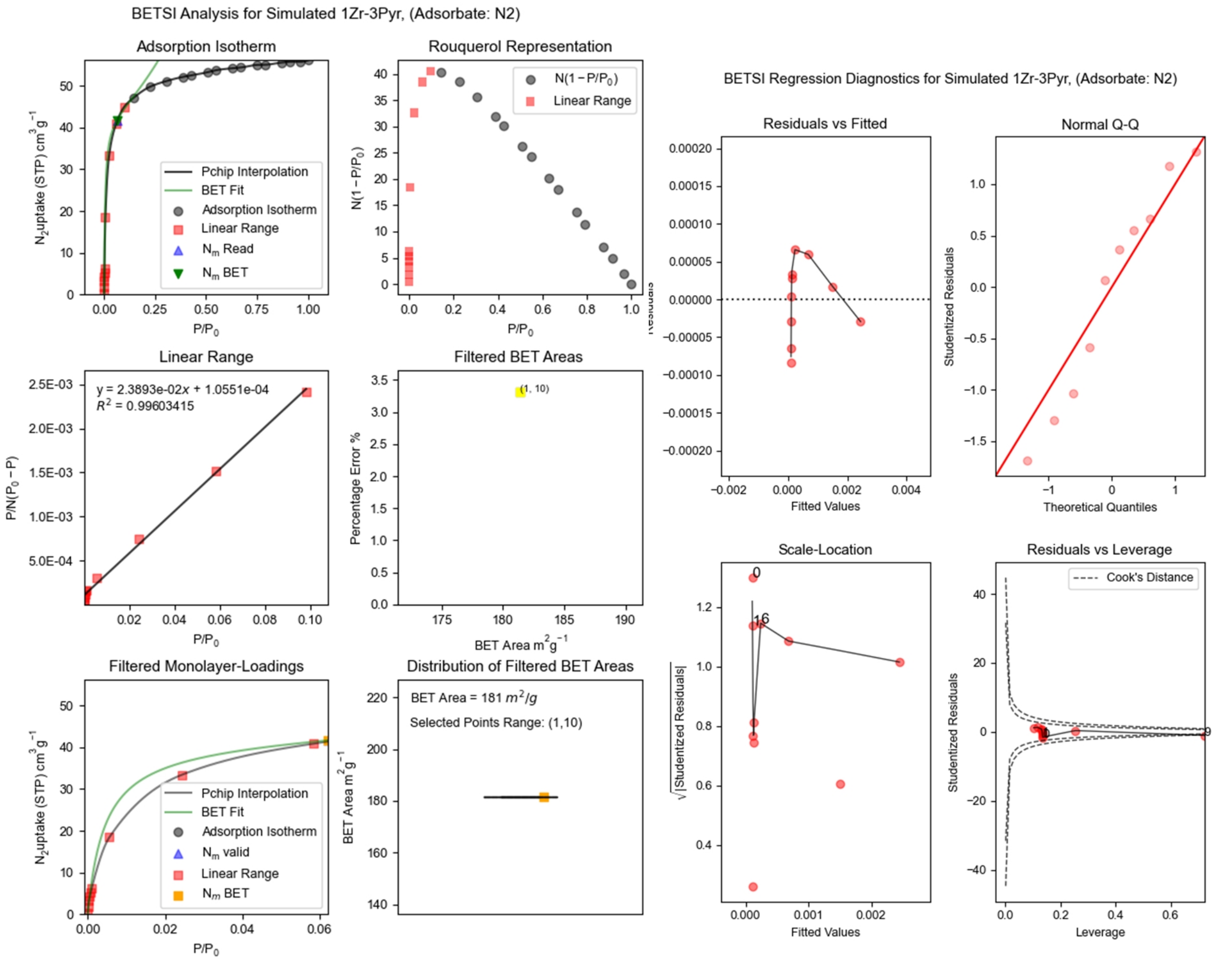

Figure S80. BETSI analysis and the corresponding regression diagnostics for the simulated $\mathbf{1^{Zr\text{-}3Pyr}}$

11 References

1. Komatsu T*, et al.* Development of 2,6-carboxy-substituted boron dipyrromethene (BODIPY) as a novel scaffold of ratiometric fluorescent probes for live cell imaging. *Chem Commun (Camb)*, 7015-7017 (2009).
2. Osterrieth JWM*, et al.* How Reproducible are Surface Areas Calculated from the BET Equation? *Adv Mater* **34**, e2201502 (2022).
3. Giannozzi P*, et al.* QUANTUM ESPRESSO: a modular and open-source software project for quantum simulations of materials. *J Phys Condens Matter* **21**, 395502 (2009).
4. Giannozzi P*, et al.* Advanced capabilities for materials modelling with Quantum ESPRESSO. *J Phys Condens Matter* **29**, 465901 (2017).
5. Perdew JP*, et al.* Restoring the density-gradient expansion for exchange in solids and surfaces. *Phys Rev Lett* **100**, 136406 (2008).
6. Heyd J, Scuseria GE, Ernzerhof M. Hybrid functionals based on a screened Coulomb potential. *The Journal of Chemical Physics* **118**, 8207-8215 (2003).
7. Wu XP, Gagliardi L, Truhlar DG. Metal doping in cerium metal-organic frameworks for visible-response water splitting photocatalysts. *J Chem Phys* **150**, 041701 (2019).
8. Wu XP, Gagliardi L, Truhlar DG. Cerium Metal-Organic Framework for Photocatalysis. *J Am Chem Soc* **140**, 7904-7912 (2018).
9. Grimme S, Antony J, Ehrlich S, Krieg H. A consistent and accurate ab initio parametrization of density functional dispersion correction (DFT-D) for the 94 elements H-Pu. *J Chem Phys* **132**, 154104 (2010).
10. Prandini G, Marrazzo A, Castelli IE, Mounet N, Marzari N. Precision and efficiency in solid-state pseudopotential calculations. *npj Computational Materials* **4**, (2018).
11. Hamann DR. Optimized norm-conserving Vanderbilt pseudopotentials. *Physical Review B* **88**, (2013).
12. Callmer B. An accurate refinement of the β-rhombohedral boron structure. *Acta Crystallographica Section B Structural Crystallography and Crystal Chemistry* **33**, 1951-1954 (1977).
13. BIOVIA, Dassault Systèmes, Materials Studio, 7.0, San Diego: Dassault Systèmes,. (2013).
14. Orellana W. Metal Substitutions in the MOF-5 Metal–Organic Framework: A Hybrid Density Functional Theory Study. *The Journal of Physical Chemistry C* **128**, 6471-6477 (2024).
15. Freysoldt C*, et al.* First-principles calculations for point defects in solids. *Reviews of Modern Physics* **86**, 253-305 (2014).
16. Freysoldt C, Neugebauer J, Van de Walle CG. Fully ab initio finite-size corrections for charged-defect supercell calculations. *Phys Rev Lett* **102**, 016402 (2009).
17. Freysoldt C, Neugebauer J, Van de Walle CG. Electrostatic interactions between charged defects in supercells. *physica status solidi (b)* **248**, 1067-1076 (2010).
18. *Freysoldt, C.; see https://sxrepo.mpie.de/projects/sphinx-add-ons/files for sxdefectalign.*).
19. Colón YJ, Gómez-Gualdrón DA, Snurr RQ. Topologically Guided, Automated Construction of Metal-Organic Frameworks and Their Evaluation for Energy-Related Applications. *Crystal Growth & Design* **17**, 5801-5810 (2017).
20. Rappe AK, Casewit CJ, Colwell KS, Goddard WA, Skiff WM. UFF, a full periodic table force field for molecular mechanics and molecular dynamics simulations. *Journal of the American Chemical Society* **114**, 10024-10035 (2002).
21. Willems TF, Rycroft CH, Kazi M, Meza JC, Haranczyk M. Algorithms and tools for high-throughput geometry-based analysis of crystalline porous materials. *Microporous and Mesoporous Materials* **149**, 134-141 (2012).

22. Dubbeldam D, Calero S, Ellis DE, Snurr RQ. RASPA: molecular simulation software for adsorption and diffusion in flexible nanoporous materials. *Molecular Simulation* **42**, 81-101 (2015).
23. Mayo SL, Olafson BD, Goddard WA. DREIDING: a generic force field for molecular simulations. *The Journal of Physical Chemistry* **94**, 8897-8909 (2002).
24. Martin-Calvo A, Garcia-Perez E, Garcia-Sanchez A, Bueno-Perez R, Hamad S, Calero S. Effect of air humidity on the removal of carbon tetrachloride from air using Cu-BTC metal-organic framework. *Phys Chem Chem Phys* **13**, 11165-11174 (2011).
25. Wilmer CE, Kim KC, Snurr RQ. An Extended Charge Equilibration Method. *J Phys Chem Lett* **3**, 2506-2511 (2012).
